# Establishment of a prognostic signature for lung adenocarcinoma using cuproptosis-related lncRNAs

**DOI:** 10.1186/s12859-023-05192-5

**Published:** 2023-03-06

**Authors:** Saiyidan Yalimaimaiti, Xiaoqiao Liang, Haili Zhao, Hong Dou, Wei Liu, Ying Yang, Li Ning

**Affiliations:** 1 School of Public Health, Xinjiang Medical University, Urumqi, 830011 Xinjiang China; 2 Xinjiang Uygur Autonomous Region Occupational Disease Hospital, Urumqi, 830011 Xinjiang China

**Keywords:** Lung adenocarcinoma, LncRNA, Copper death, Prognostic signature, Biomarker

## Abstract

**Objective:**

To establish a prognostic signature for lung adenocarcinoma (LUAD) based on cuproptosis-related long non-coding RNAs (lncRNAs), and to study the immune-related functions of LUAD.

**Methods:**

First, transcriptome data and clinical data related to LUAD were downloaded from the Cancer Genome Atlas (TCGA), and cuproptosis-related genes were analyzed to identify cuproptosis-related lncRNAs. Univariate COX analysis, least absolute shrinkage and selection operator (LASSO) analysis, and multivariate COX analysis were performed to analyze the cuproptosis-related lncRNAs, and a prognostic signature was established. Second, univariate COX analysis and multivariate COX analysis were performed for independent prognostic analyses. Receiver operating characteristic (ROC) curves, C index, survival curve, nomogram, and principal component analysis (PCA) were performed to evaluate the results of the independent prognostic analyses. Finally, gene enrichment analyses and immune-related function analyses were also carried out.

**Results:**

(1) A total of 1,297 cuproptosis-related lncRNAs were screened. (2) A LUAD prognostic signature containing 13 cuproptosis-related lncRNAs was constructed (NIFK-AS1, AC026355.2, SEPSECS-AS1, AL360270.1, AC010999.2, ABCA9-AS1, AC032011.1, AL162632.3, LINC02518, LINC0059, AL031600.2, AP000346.1, AC012409.4). (3) The area under the multi-indicator ROC curves at 1, 3, and 5 years were AUC1 = 0.742, AUC2 = 0.708, and AUC3 = 0.762, respectively. The risk score of the prognostic signature could be used as an independent prognostic factor that was independent of other clinical indicators. (4) The results of gene enrichment analyses showed that 13 biomarkers were primarily related to amoebiasis, the wnt signaling pathway, hematopoietic cell lineage. The ssGSEA volcano map showed significant differences between high- and low-risk groups in immune-related functions, such as human leukocyte antigen (HLA), Type_II_IFN_Reponse, MHC_class_I, and Parainflammation (*P* < 0.001).

**Conclusions:**

Thirteen cuproptosis-related lncRNAs may be clinical molecular biomarkers for the prognosis of LUAD.

**Supplementary Information:**

The online version contains supplementary material available at 10.1186/s12859-023-05192-5

## Introduction

Lung cancer is a malignant tumor that has posed a serious threat to the health of humans in recent decades. According to the 2020 GLOBALCAN statistics, the incidence of breast cancer is the highest among 36 types of cancer in 186 countries, but lung cancer is the leading cause of cancer-related death [1]. Lung cancer ranks first in incidence and mortality among all cancers, and is the most common cancer in males with the highest fatality rate [2]. Lung adenocarcinoma (LUAD) is a common type of non-small cell lung cancer, accounting for about 50%, and the 5-year survival rate is only 18% [3, 4]. Lung cancer is causing increasingly serious harm to humans, it is extremely important to find more effective methods of diagnosis and treatment.

Recently published articles have proposed the term copper death [5]. Copper death is caused by the direct binding of copper ions to the lipoacylated components of the tricarboxylic acid cycle of the mitochondrial respiratory chain, leading to the aggregation of lipoacylated proteins and the subsequent down-regulation of iron-sulfur clusters, which leads to proteotoxic stress, and ultimately, to cell death [5]. The discovery of copper death has provided a new understanding of the occurrence and development of tumor-related diseases, however, there are few reports relating copper death and LUAD.

Long non-coding RNAs (LncRNAs) are a large group of RNAs that are longer than 200 nt and lack protein-coding ability, and are located in the nucleus or cytoplasm [6]. In 1991, lncRNA was first isolated and identified by the mouse Xist gene and proved to have biological functions [7]. Previously, it was considered as a by-product of gene transcription. Studies have shown that the wrong expression of lncRNA is associated with many diseases, such as leukemia, diabetes, hypertension, coronary heart disease, Alzheimer's disease and tumor diseases. LncRNA has been proved to be widely involved in the diagnosis and treatment of diseases and can be used as a potential marker of diseases [8]. Relevant research shows that MEG3, HOTAIR, MALAT1 and other genes play a role in the occurrence and development of lung cancer [9–11]. AL161431.1 promotes the proliferation and migration of endometrial cancer cells by targeting mir-1252-5p and MAPK signaling pathways [12]. The expression of ABCA9-AS1 is up-regulated in calcium oxalate monohydrate (COM)-induced epithelial-mesenchymal transition (EMT) of renal tubular epithelial cells [13]. The expression of LANCL1-AS1 is down-regulated in non-small cell lung carcinoma (NSCLC) [14]. Studies have shown that lncRNAs are involved in the occurrence and development of LUAD, which can provide ideas for the diagnosis and prognosis of LUAD [15, 16]. Therefore, there is no doubt that identifying cuproptosis-related lncRNAs is of great significance for the prevention and treatment of LUAD.

At present, the role of copper death and its related lncRNAs in LUAD remain unclear. In this study, the Cancer Genome Atlas (TCGA) database was used to construct a prognostic signature of LUAD using bioinformatics methods with cuproptosis-related lncRNAs. To identify prognostic molecular markers of LUAD and provide certain value for clinical diagnosis of LUAD.

## Materials and methods

### Datasets and patients

Transcriptome data and clinical data of LUAD patients were downloaded from the TCGA database **(**https://portal.gdc.cancer.gov), the transcriptome data were extracted in the fragment per kilobase million (FPKM) format that has been normalized. Clinical data included age, grade, stage, T stage, M stage, N stage, survival status, and survival time. Because the grade of all patients was unknown, the grade was excluded from the analysis. Finally, clinical and sample information for 471 patients were randomly divided into training and validation sets according to the ratio of 3:2.The basic information of the training and validation sets is shown in Additional file 1: Table S1. LncRNAs and mRNAs were separated by sorting the downloaded transcriptome data using Perl software. As the clinical information of the patients involved in this study was obtained from the TCGA database and was in strict compliance with the TCGA publication guidelines, ethics committee approval was not required (Additional file 2: Table S2, Additional file 3: Table S3, Additional file 4: Table S4 and Additional file 5: Table S5).

### Identification of cuproptosis-related lncRNAs

R software “limma” package was used to calculate the association coefficient between lncRNAs and cuproptosis-related genes. The screening criteria were |cor|> 0.4, *P*-value < 0.001, and the screening results were visualized using the ggplot package of R software.

### Establishment and validation of the cuproptosis-related lncRNA signature

Univariate Cox regression analysis was used to screen the analysis results and to obtain prognostic cuproptosis-related lncRNAs. Cuproptosis-related lncRNAs with *P* < 0.05 were included in Lasso regression analyses to reduce data overfitting. The R software package glmnet was used for Lasso regression analysis. The results of Lasso screening were included in the multivariate Cox regression model to obtain the key cuproptosis-related lncRNAs for signature construction, and a Risk Score (RS) was calculated. The scoring formula was as follows:

$${\text{Risk}}\;{\text{Score}} = \sum\limits_{{{\text{i}} = 1}}^{{\text{N}}} {({\text{Expi}}\;*\;{\text{Coei}})}$$

N represents the number of prognostic cuproptosis-related lncRNAs in the risk signature, Expi represents the expression value of each lncRNA, and Coei represents the regression coeffificient of each lncRNA in the multivariate Cox regression analysis.

### Assessment of prognostic risk signature

The included subjects were divided into high- and low-risk groups based on the median risk score. The survival analysis method was used to compare the Overall Survival (OS) and Progress Free Survival (PFS) of LUAD patients in the training set, validation set, and among all groups. The R software time ROC package was used to draw the Receiver Operating Characteristic (ROC) curves for 1, 3, and 5 years, while the R software survival, rms and pec packages were used to draw the C-index curves to evaluate the predictive ability of the prognostic signature. The risk score and other clinical traits were then included in univariate COX regression analyses and multivariate COX regression analyses to analyze whether the riskscore was a prognostic factor that was independent of other clinical indicators. Principal Component Analysis (PCA) was used to validate the risk signature and the results were visualized using the R software scatterplot3D package. The PFS of the high- and low-risk groups was analyzed using the R software survival and survminer packages. Patients were divided into stages I-II and III-IV to determine whether the risk signature is appropriate for patients with different stages of LUAD.

### Nomogram and calibration

A nomogram was established using the R software rms, regplot, and survival packages. The nomogram was used to predict the 1-year, 3-year, and 5-year OS rates of LUAD patients. The calibration curve was drawn to evaluate the fitting and predictive ability of the nomogram.

### Enrichment function analysis

The R software clusterprofile package was used to perform Gene Ontology (GO) function and pathway enrichment analyses (Kyoto Encyclopedia of Genes and Genomes) for the selected differentially expressed cuproptosis-related genes [17–19]. KEGG analyses can excavate the biological processes related to the biological problems associated with LUAD. GO enrichment analysis annotates and classifies genes in terms of biology process (BP), molecular function (MF) and cellular component (CC). KEGG pathway enrichment analysis can also be used to understand the possible signaling pathways involved in LUAD.

### Immune-related functional analysis

The expression levels of the 13 cuproptosis-related lncRNAs from the prognostic risk signature and the corresponding sample risk score were integrated. The ssGSEA enrichment analysis was used to evaluate the immune response in the high- and low-risk groups. The R software GSVA, GSEABase, and reshape2 packages were used for immune-related function analysis, and the pheatmap package was used for visualization.

## Results

### Acquisition of cuproptosis-related lncRNAs

**Fig. 1:**
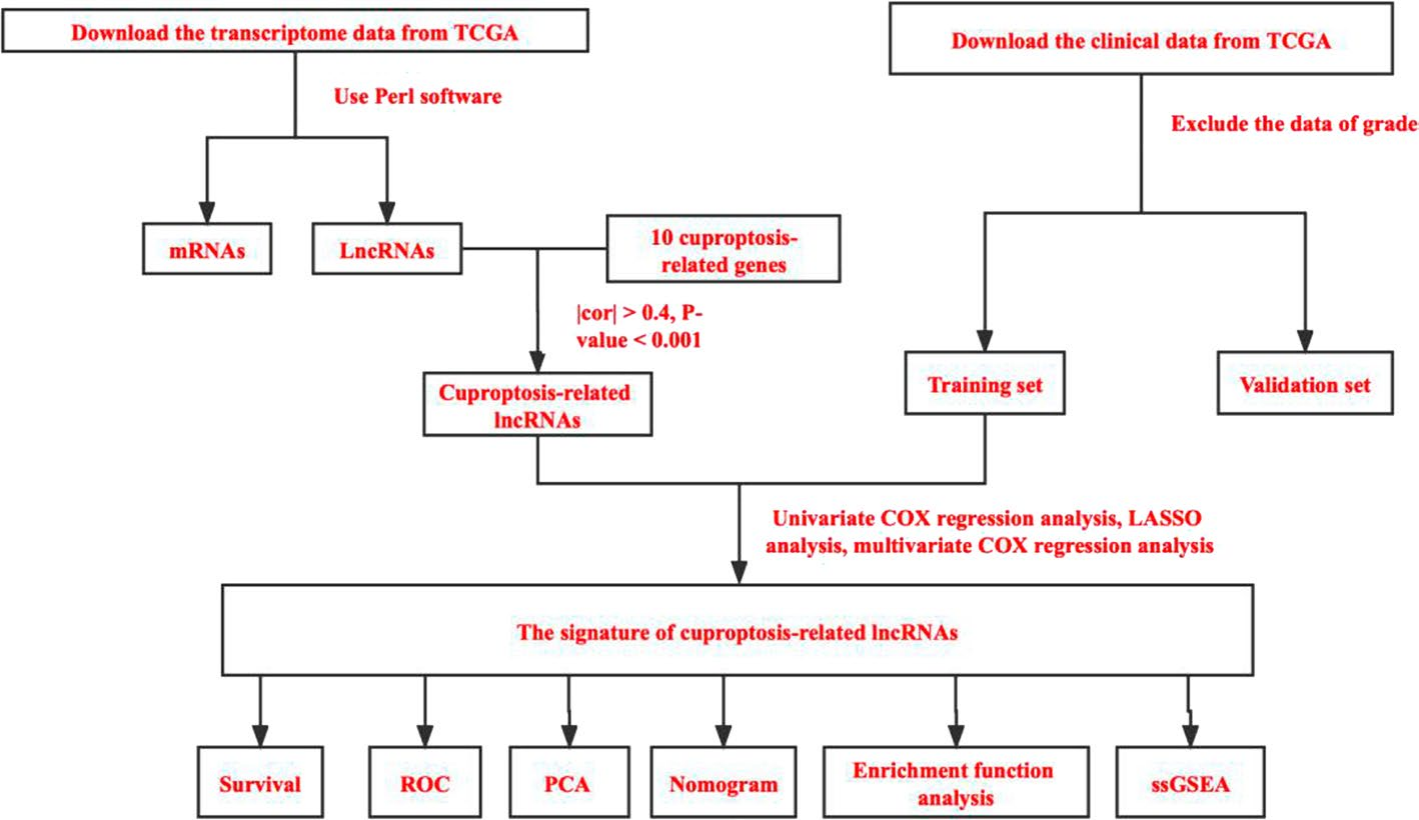
Study flow chart. TCGA, The Cancer Genome Atlas database; lncRNAs, long non-coding RNAs; ROC, Receiver Operating Characteristic; PCA, Principal component analysis; GSEA, Gene Set Enrichment Analysis

**Fig. 2:**
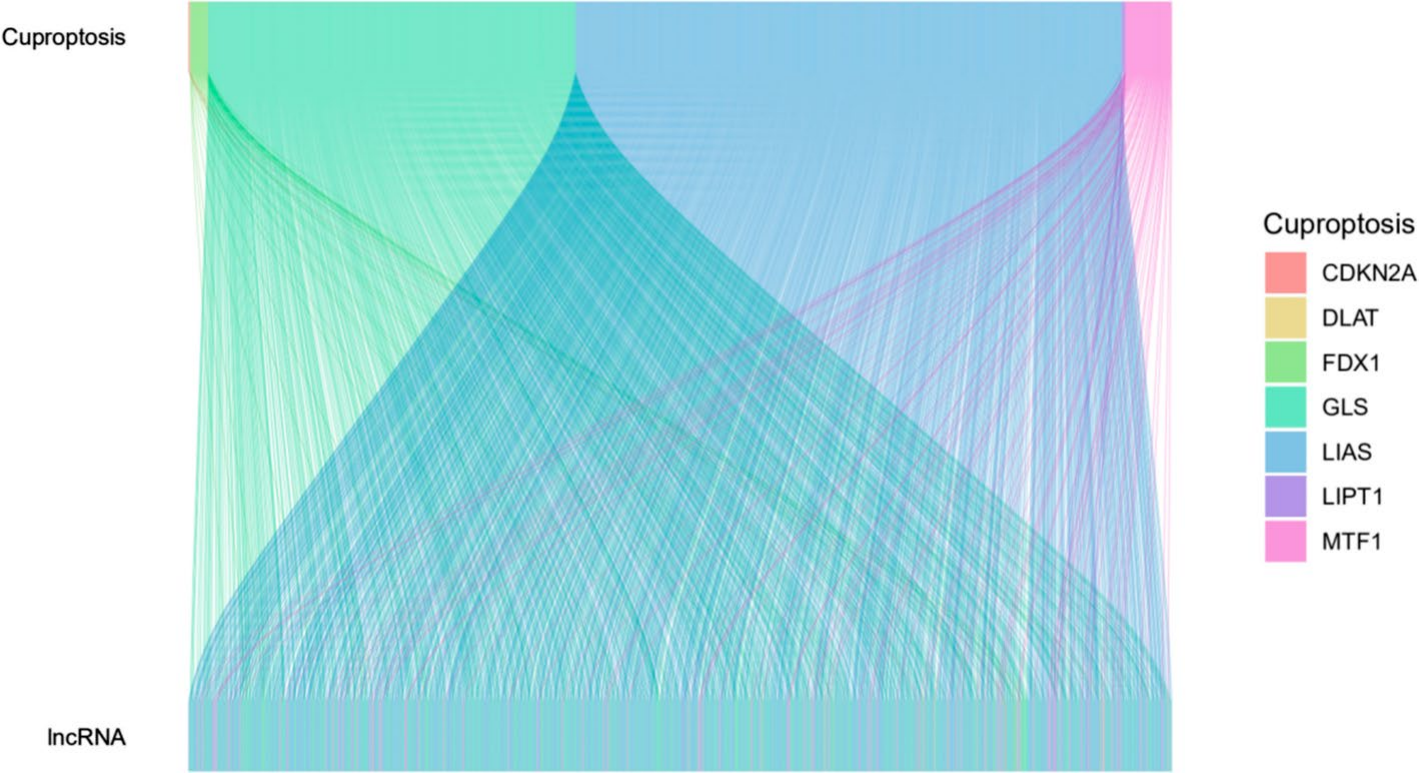
Sankey diagram of cuproptosis-related lncRNAs

Figure 1 shows a flow diagram of the study. A total of 16876 lncRNAs were identified in the TCGA-LUAD database. A total of 10 cuproptosis-related genes (FDX1, LIAS, LIPT1, DLD, DLAT, PDHA1, PDHB, MTF1, GLS, CDKN2A) [5] were obtained, and a cuproptosis-related lncRNA co-expression network was constructed to identify cuproptosis-related lncRNAs. Finally, 1297 cuproptosis-related lncRNAs (|cor|> 0.4, *P*-value < 0.001) were screened (Fig. 2).

### Construction of the cuproptosis-related lncRNA signature

**Fig. 3:**
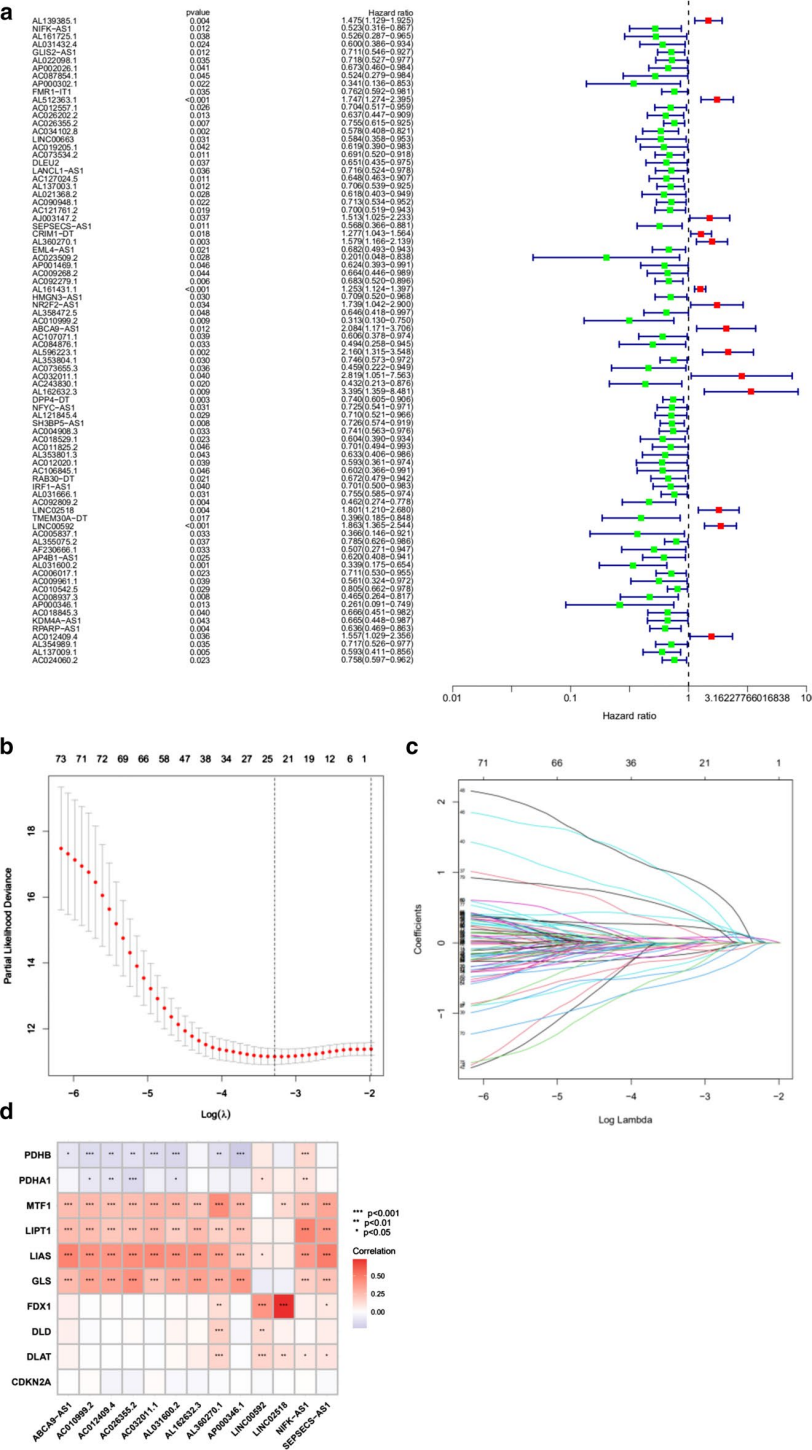
Identification of prognostic cuproptosis-associated lncRNAs signature in LUAD. **a** Univariate Cox regression analysis for identifying the prognostic cuproptosis-related lncRNAs. The red colors in forest map represent high-risk lncRNAs, the green colors in forest map represent low-risk lncRNAs. **b**, **c** Lasso-Cox regression analysis was performed to construct prognostic prediction signature. **d** Correlation heatmap showing the relationship between cuproptosis-related lncRNAs and cuproptosis-related genes for the signature

A total of 82 target lncRNAs related to the prognosis of LUAD patients were preliminarily screened by univariate Cox regression analyses and forest maps were drawn (Fig. 3a). The detail result of univariate Cox regression analysis is shown in Additional file 2: Table S2. Then, the initial screening variables were further screened and analyzed by Lasso regression to reduce the over-fitting of the data. A total of 24 cuproptosis-related lncRNAs with higher prognostic values were then identified, and the Lasso regression coefficient spectrum was drawn (Fig. 3b and c). Then, multivariate Cox regression analysis was performed to identify the 13 cuproptosis-related lncRNAs with prognostic value in LUAD patients. The 13 cuproptosis-related lncRNAs were used to construct the LUAD prognosis signature. Riskscore = -0.513758617008653*NIFK-AS1-0.384776056894586*AC026355.2-0.654434692150166*SEPSECS-AS1 + 0.300148273925387*AL360270.1-0.934043863436313*AC010999.2 + 1.03622089286485*ABCA9-AS1 + 1.63567286520565*AC032011.1 + 1.51230620978365*AL162632.3 + 0.386468780588746*LINC02518 + 0.531265768638627*LINC00592-0.994358590794876*AL031600.2-0.903105986751972*AP000346.1 + 1.01709054082397*AC012409.4. Individuals were then divided into high- and low-risk groups according to their risk score.

Figure 3d showed LINC02518 had a strong positive correlation with FDX1 (*P* < 0.001).

### Survival analysis

**Fig. 4:**
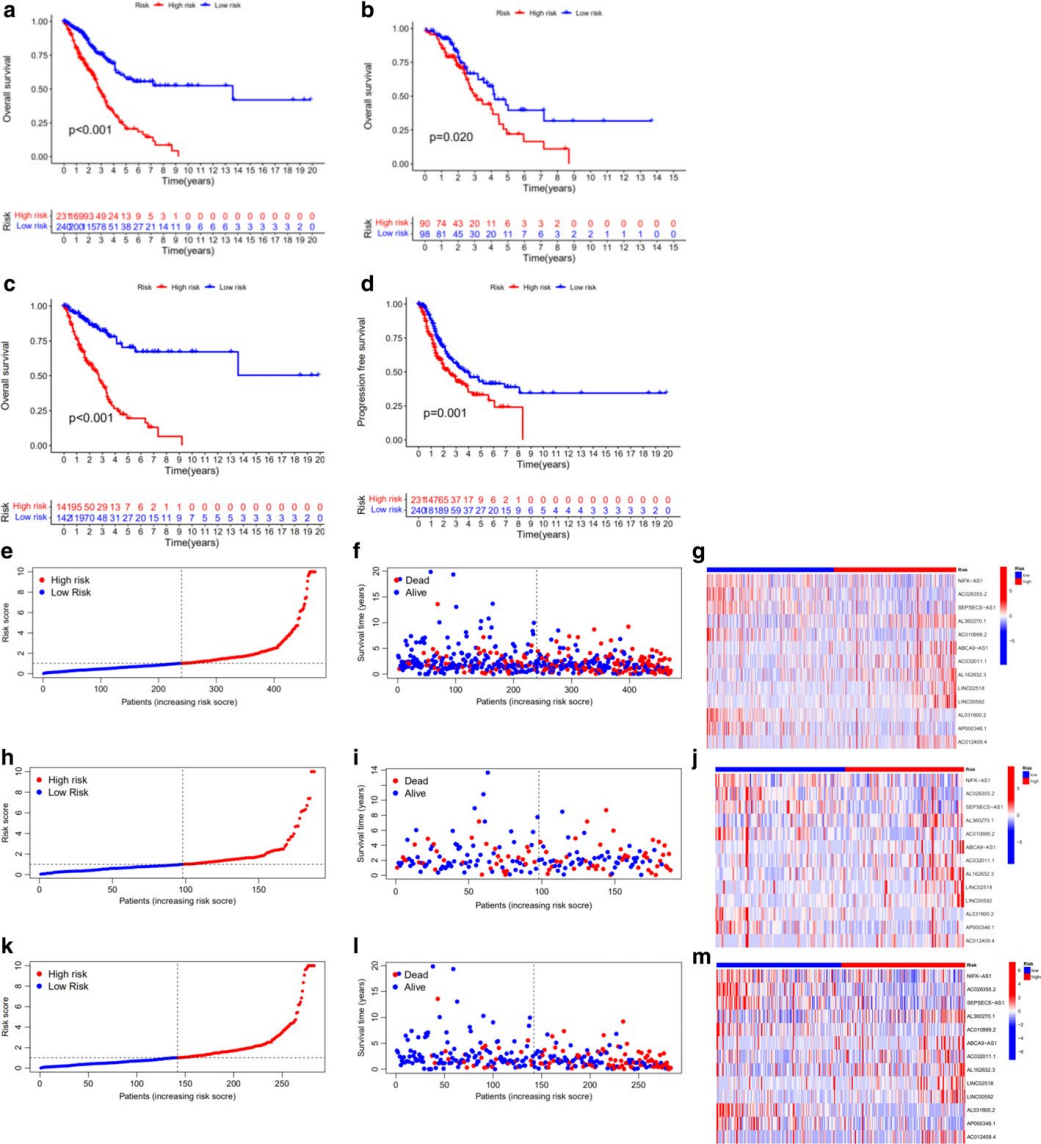
Kaplan–Meier survival analyses of patients in high and low risk groups. **a** OS in all groups. **b** OS in the test group. **c** OS in the training group. **d** PFS in all groups. **e**–**g** Risk score map, survival status map and heat map of the expression of 13 lncRNAs in all groups. **h**–**j** Risk score map, survival status map and heat map of the expression of 13 lncRNAs in the test group. **k**–**m** Risk score map, survival status map and heat map of the expression of 13 lncRNAs in the training group

Patients were divided into high- and low-risk groups based on their median risk score, OS and PFS were compared. In the training set, validation set, and overall set, the high-risk group had lower OS and PFS than the low-risk group (Fig. 4a–d). The risk score map, survival status map, and heat map were also drawn, and revealed that the high-risk group had a higher risk score and a higher mortality than the low-risk group (Fig. 4e–m).

### Independent analysis of prognostic factors

**Fig. 5:**
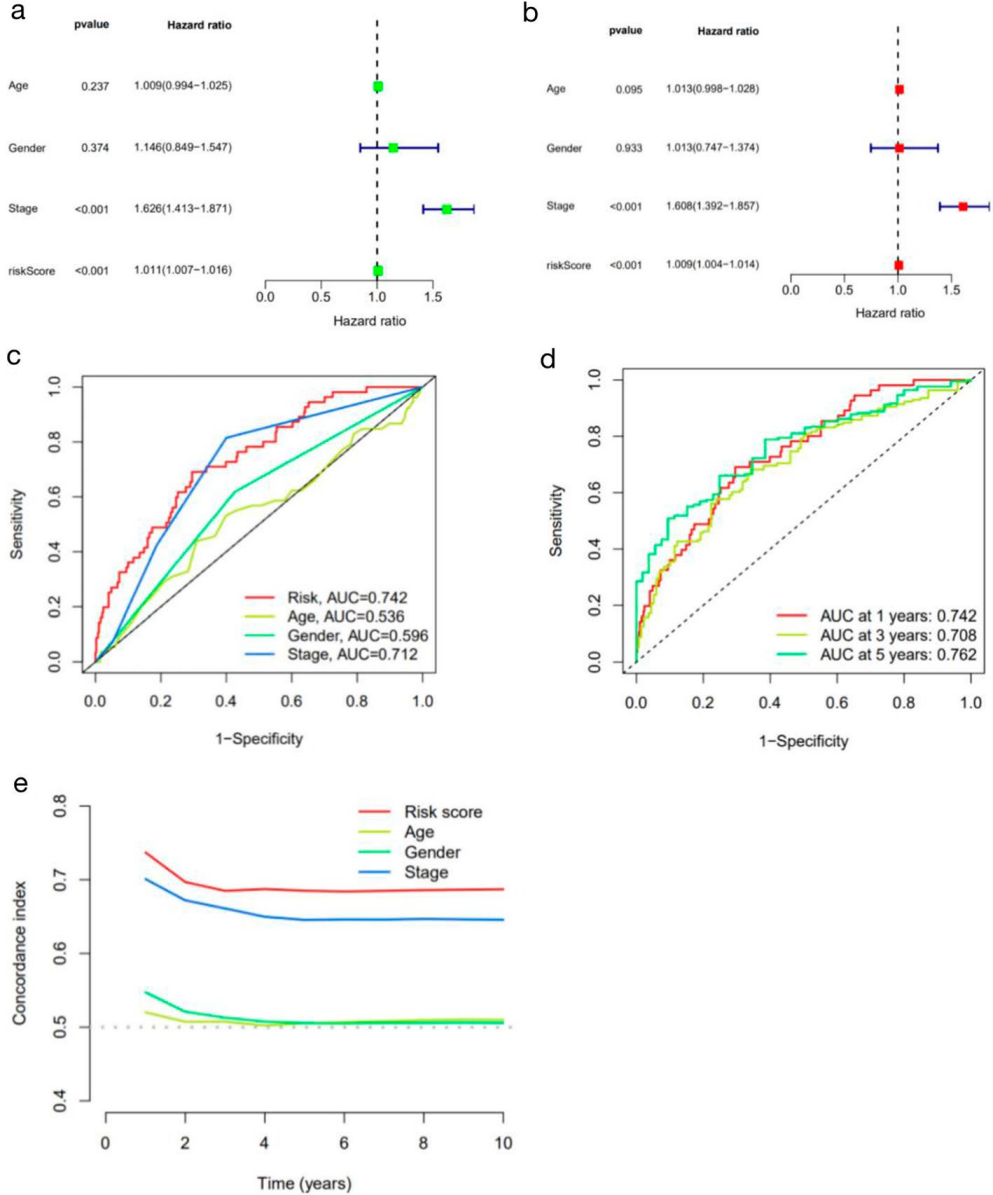
The prognostic value of the signature for LUAD. **a**, **b** Cox regression analysis was used to analyze the hazard ratio (HR) of the riskscore. **c** ROC curves of the riskscore and multiple clinical features. **d** ROC curves of the risk model at different times. **e** C-index curve of the risk mode

The results of univariate and multivariate COX regression analyses showed that the risk score could be used as an independent prognostic indicator for the prognosis of LUAD patients (all *P* < 0.001) (Fig. 5a and b). ROC curve showed the AUC of 1 year, 3 years and 5 years (AUC1 = 0.742, AUC2 = 0.708, AUC3 = 0.762, respectively). ROC curves of risk score and different clinical features were drawn, and it was found that the AUC of the risk score was higher than the AUC score of other clinical features, which indicated that this signature had a relatively accurate predictive ability (Fig. 5c and d). We also found that the C index value of the risk score was higher than the other clinical characteristics, such as age, gender, and disease stage (Fig. 5e).

### Nomogram and PCA

**Fig. 6:**
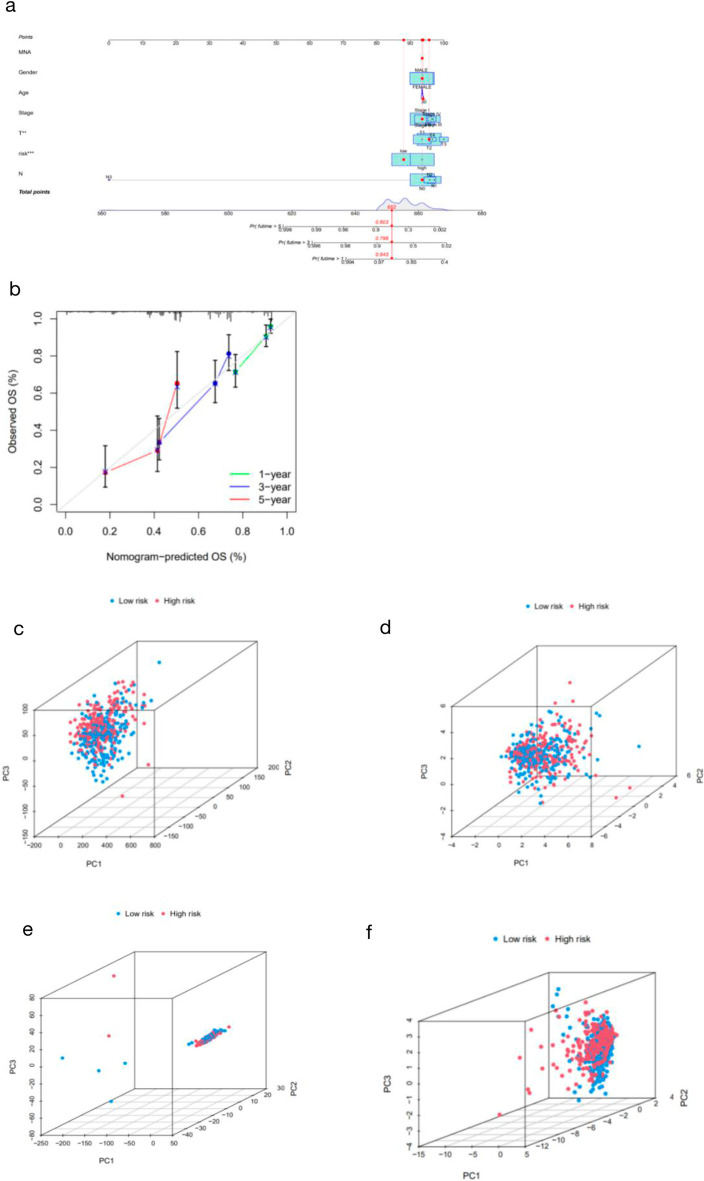
Construction and validation of the nomogram. **a** 1-, 3-, and 5-year survival nomograms of LUAD patients with clinical indicators. **b** Calibration curves used to evaluate the prognostic signature predicting 1-, 3-, and 5-year survival rates in patients with LUAD. Dashed gray lines represent the ideal prediction models. **c** PCA of all genes. **d** PCA of cuproptosis-related genes. **e** PCA of cuproptosis-related lncRNAs. **f** PCA of risk lncRNAs

A nomogram was drawn to further predict the survival rate of patients, and the calibration plot showed that the predicted probability was consistent with the actual probability (Figs. 6a and b). The detail data of nomogram and calibration is shown in Additional file 5: Table S5. We then used PCA to explore the differences between the high- and low-risk groups across four different expression profiles. The results revealed that these lncRNAs could be reliably used to construct the signature (Fig. 6c–f).

### Signature validation of clinical grouping

**Fig. 7:**
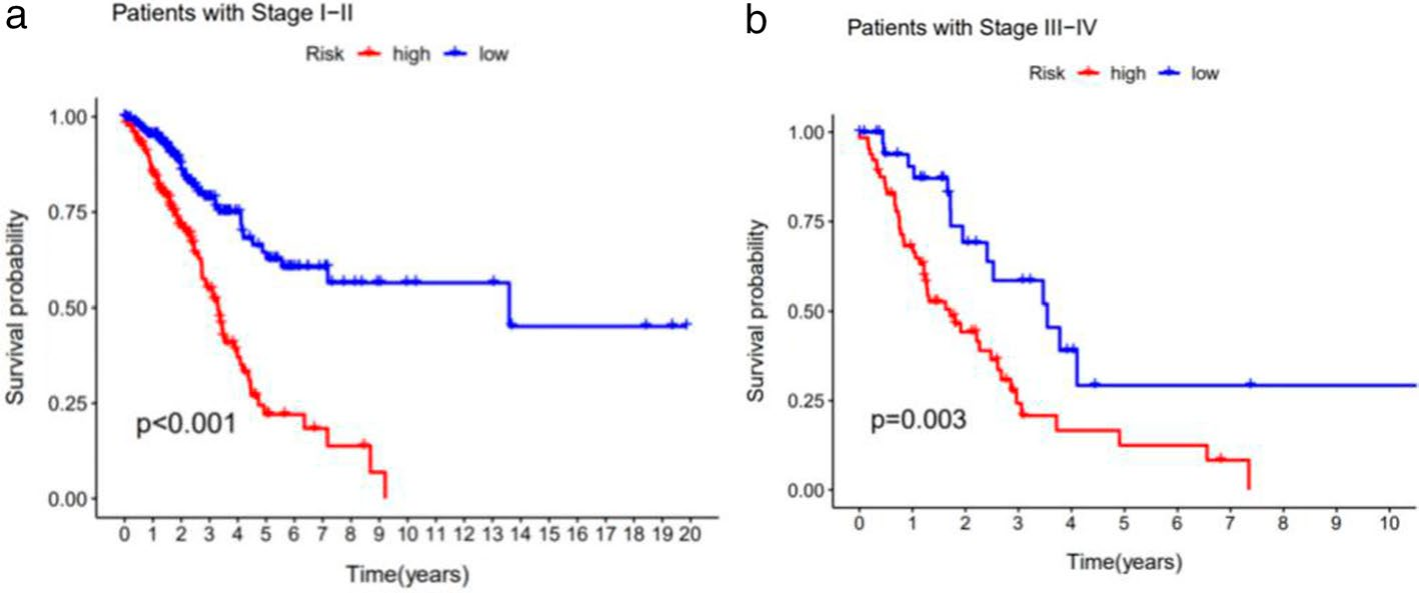
Signature validation results of clinical grouping. **a** Survival curves for early stage patients. **b** Survival curves for advanced patients

The constructed signature is not only applicable to patients in the early stage but also to patients in the late stage (*P* < 0.05) (Figs. 7a and b).

### Gene functional enrichment analysis

**Fig. 8:**
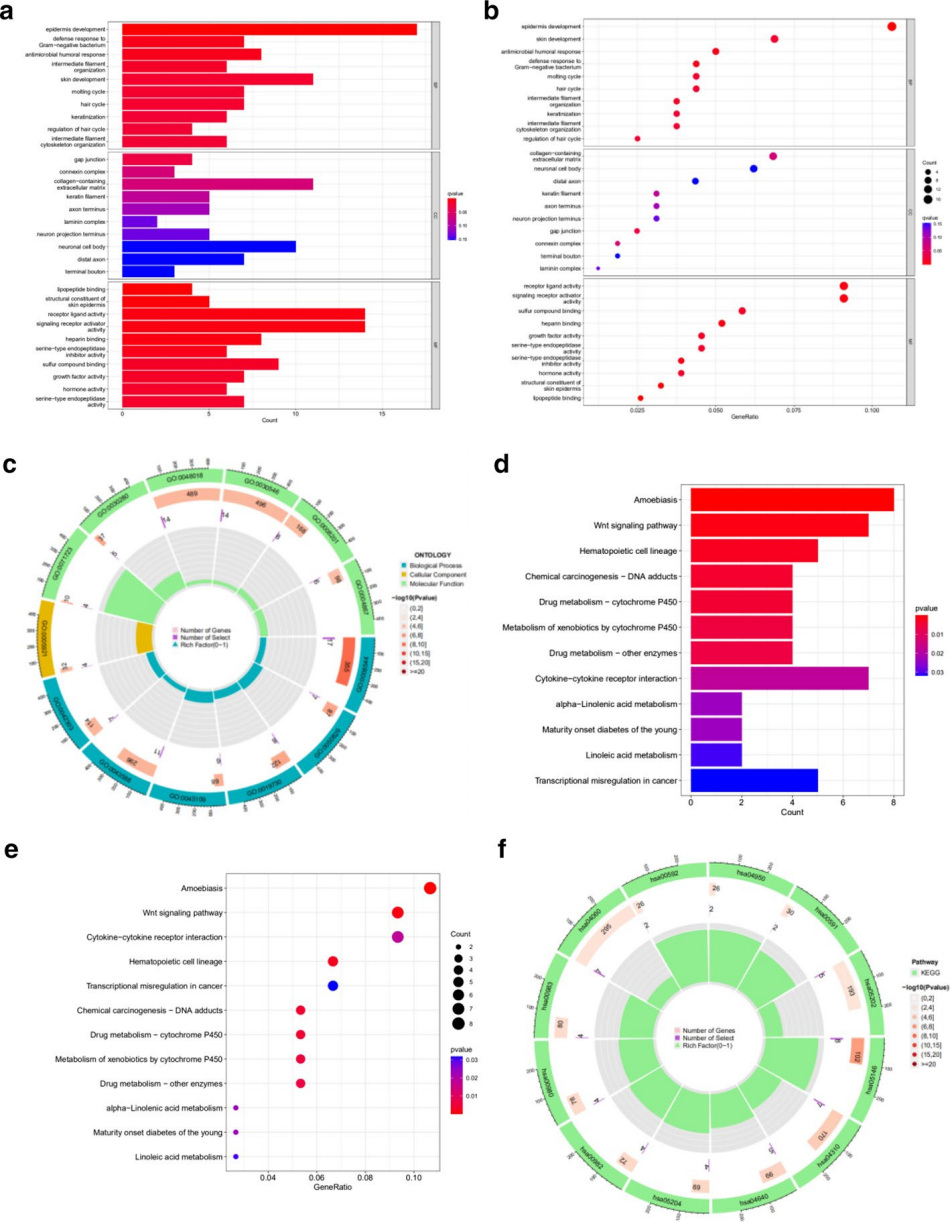
GO and KEGG analyses. **a** Barplot of the top 10 GO enrichment terms. **b** Bubble chart of the top 10 GO enrichment terms. **c** Circle diagram of GO enrichment analysis. **d** Barplot of the top 30 KEGG enrichment terms. **e** Bubble chart of the top 30 KEGG enrichment terms. **f** Circle diagram of KEGG enrichment analysis

GO analyses showed that cuproptosis-related lncRNAs in BP were mainly concentrated in antimicrobial humoral response, defense response to gram-negative bacteria, and epidermis development categories. CC mainly focuses on collagen-containing extracellular matrix, neuronal cell body, and distal axon categories, among others. MF mainly focuses on receptor ligand activity, signaling receptor activator activity, and sulfur compound binding categories (Figs. 8a–c). The detail result of GO enrichment analysis is shown in Additional file 3: Table S3. The KEGG pathway suggested that cuproptosis-related lncRNAs were involved in amoebiasis, wnt signaling pathway, and hematopoietic cell lineage, which means these lncRNAs are involved in the development of tumors (Figs. 8d–f). The detail result of KEGG enrichment analysis is shown in Additional file 4: Table S4.

### Analysis of immune-related function

**Fig. 9:**
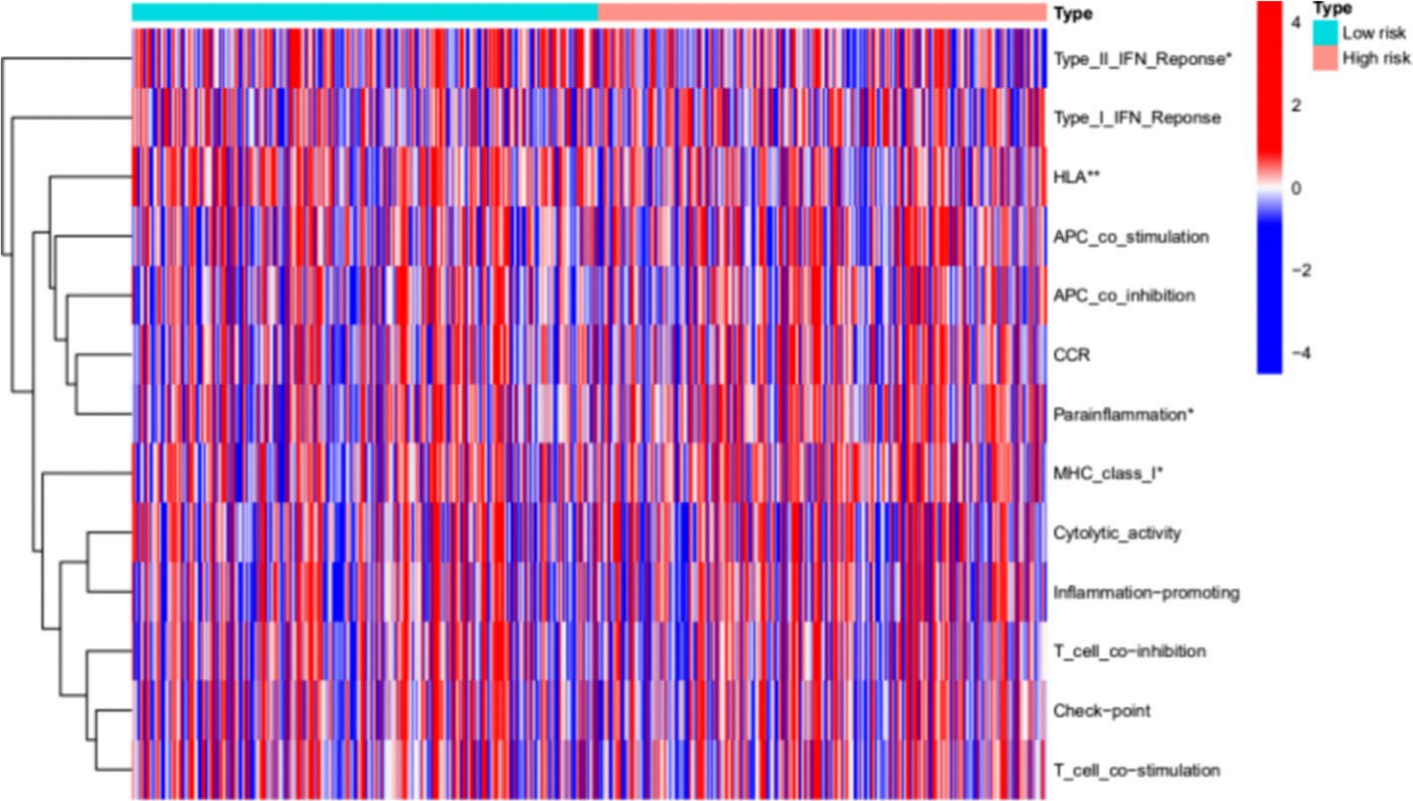
Results of immune-related functional analysis

We explore the relationship between risk score and immune-related function in LUAD, the ssGSEA volcanic map shows that the high- and low-risk groups differed significantly in their immune-related functions, including human leukocyte antigen (HLA), Type_II_IFN_Reponse, MHC_class_I, and Parainflammation (*P* < 0.05) (Figs. 9).

## Discussion

Lung cancer has the characteristics of high incidence, high mortality, and poor prognosis, and therefore, the effective prediction of therapeutic targets and precise treatments are necessary [20–22]. Copper death is a copper- and mitochondria-dependent independent cell death mechanism that was proposed on March 17, 2022[5]. Copper death is a new RCD (regulatory cell death) mode that is different from other cell death mechanisms, such as iron death. Cuproptosis may be an effective way to treat many types of cancer in the future [23]. Studies have confirmed that the abnormal expression of lncRNAs is closely related to cancer and human degenerative diseases [24–27] and lncRNAs play an indispensable role in the diagnosis, treatment, and prognosis of tumors [28–30]. However, there are few researches about cuproptosis-related lncRNAs and LUAD.

In this study, clinical data related to LUAD were downloaded from the TCGA website and cuproptosis-related lncRNAs were identified. Univariate COX analysis, Lasso analysis, and multivariate COX analysis were performed on the cuproptosis-related lncRNAs and a prognostic signature based on 13 cuproptosis-related lncRNAs was established (NIFK-AS1, AC026355.2, SEPSECS-AS1, AL360270.1, AC010999.2, ABCA9-AS1, AC032011.1, AL162632.3, LINC02518, LINC0059, AL031600.2, AP000346.1, AC012409.4). Univariate and multivariate COX analyses showed that risk score could be used as an independent prognostic indicator for patients with LUAD. ROC curves, C index, survival curves, nomograms, and PCA results showed that the signature had a good ability to predict the prognosis of LUAD patients.

The computational models can provide a theoretical basis for the smooth conduct of subsequent experiments, and as an experimental aid may shorten the experimental time and experimental cost. Currently some computational models are widely used in various diseases, Laplacian Regularized Least Squares for LncRNA–Disease Association (LRLSLDA) focuses on the relationship between lncRNAs and diseases, LRLSLDA has better performance compared with previous methods and may be an effective and important biological tool for biomedical research [31]. NCMCMDA is mainly used to predict the correlation between potential miRNA and diseases. NCMCMDA shows reliable and accurate prediction performance when applied to colon cancer, esophageal cancer, breast cancer and other diseases [32]. GCNAT is a useful biomedical research tool for predicting the relationship between potential metabolites and diseases [33]. In the future, we will further explore the relationship between the computational models and prognostic signature identification of LUAD, providing convenience for the development of LUAD related experiments.

There are few studies on the 13 cuproptosis-related lncRNAs in cancer. NIFK-AS1 is highly expressed in hepatocellular carcinoma (HCC) tissues and cells, and its upregulation is caused by METTL3-dependent m6 A methylation. A competitive endogenous RNA (ceRNA) network involving the NIFK-AS1/miR-637/AKT1 axis was identified by Chen et al*.*, and NIFK-AS1 mediates increased MMP-7 and MMP-9 expression in HCC through AKT1 [34]. NIFK-AS1 has been shown to be highly expressed in HCC tissues and promotes the cycle progression of HCC cells by interacting with SRSF10. Thus, those data provide new insights into the therapeutic targets of HCC [35]. NIFK-AS1 was found to potentially participate in dilated cardiomyopathy (DCM) through ceRNA [36]. Tang et al. found NIFK-AS has been included in the prognostic signature to predict the prognosis of colorectal cancer(CRC) patients [37]. All these results indicate that NIFK-AS1 is a novel therapeutic target. AC026355.2 was an important member of the LUAD prognostic model [38–41]. Lu et al. construct a prediction model with seven lncRNAs, including AC010999.2, which provides guidance for the immunotherapy of LUAD patients [38]. Cui et al. showed that LINC02518 was up-regulated in HCC samples compared to the control group, LINC02518 is negatively correlated with the prognosis of HCC[42]. DNA methylation data revealed that LINC00592 was up-regulated in cervical cancer (CCA) patients, suggesting that LINC00592 may be an important CCA-related lncRN [43]. A model including LINC00592 and 10 other lncRNAs related to iron death of LUAD patients was constructed, suggesting that the 10 lncRNAs were involved in immune-related processes [44]. However, there are few reports on SEPSECS-AS1, AL360270.1, AC032011.1, AL162632.3, AL031600.2, AP000346.1, AC012409.4. Thus, it is necessary to carry out relevant researches on these cuproptosis-related lncRNAs in the future.

We further conducted GO enrichment analysis and KEGG pathway analysis, which suggested that cuproptosis-related lncRNAs were involved in immune-related pathways. The high- and low-risk groups differed significantly in their immune-related functions of HLA, Type_II_IFN_Reponse, MHC_class_I, and parainflammation. This suggests that it is involved in immune-related pathways, and further studies on immune microenvironment-related aspects will be done.

In this study, we constructed 13 cuproptosis-related lncRNAs to predict the prognosis of patients with LUAD through bioinformatics analysis, to provide new ideas and directions for prevention, screening and treatment. However, the limitations of this study were as follows: the analysis was only based on a database and lacked qRT-PCR vertification. In the future, we will conduct functional studies and molecular mechanism experiments.

## Conclusions

In conclusion, this study successfully constructed a risk prediction signature for the prognosis of LUAD using bioinformatics methods, providing direction for screening high-risk groups.

## Supplementary Information

**Additional file 1**. Clinical characteristics of the validation set and training set for LUAD [n(%)].

**Additional file 2**. The result of univariate Cox regression analysis.

**Additional file 3**. The detail result of GO enrichment analysis.

**Additional file 4**. The detail result of KEGG enrichment analysis.

**Additional file 5**. The detail data of nomogram and calibration.

## Data Availability

Transcriptome data and clinicTranscriptome data and clinical data of LUAD patients during the current study are available in the TCGA database (https://portal.gdc.cancer.gov; accessed 25 September 2022).
