# Supplementary material for: Establishment of a prognostic signature for lung adenocarcinoma using cuproptosis-related lncRNAs
Source: BMC Bioinformatics. 2023 Mar 6;24:81. doi: 10.1186/s12859-023-05192-5 (PMC9990240; doi:10.1186/s12859-023-05192-5)
Supplement: Supplementary file 1 — Additional file 1. Clinical characteristics of the validation set and training set for LUAD [n(%)]. [file 12859_2023_5192_MOESM1_ESM.pdf]

Table 1 Clinical characteristics of the validation set and training set for LUAD [n (%)]

Covariates	Type	Total	Validation	Train	Pvalue
Age	<=65	225(47.77)	89(47.34)	136(48.06)	0.8078
	>65	236(50.11)	97(51.60)	139(49.12)	
	unknow	10(2.12)	2(1.06)	8(2.83)	
Sex	FEMALE	256(54.35)	100(53.19)	156(55.12)	0.7506
	MALE	215(45.65)	88(46.81)	127(44.88)	
Stage	Stage I	255(54.14)	98(52.13)	157(55.48)	0.866
	Stage II	108(22.93)	46(24.47)	62(21.91)	
	Stage III	75(15.92)	30(15.96)	45(15.90)	
	Stage IV	25(5.31)	11(5.85)	14(4.95)	
T	T1	160(33.97)	3(1.60)	5(1.77)	
	T2	250(53.08)	65(34.57)	95(33.57)	0.238
	T3	39(8.28)	97(51.6)	153(54.06)	
	T4	19(4.03)	19(10.11)	20(7.07)	
	unknow	3(0.64)	4(2.13)	15(5.30)	
M	M0	318(67.52)	3(1.60)	0(0.00)	
	M1	24(5.10)	125(66.49)	193(68.20)	0.9909
	unknow	129(27.39)	10(5.32)	14(4.95)	
N	N0	304(64.54)	53(28.19)	76(26.86)	
	N1	87(18.47)	120(63.83)	184(65.02)	0.6006
	N2	66(14.01)	38(20.21)	49(17.31)	
	N3	2(0.42)	26(13.83)	40(14.13)	
	unknow	12(2.55)	0(0.00)	2(0.71)	
