# Supplementary material for: Establishment of a prognostic signature for lung adenocarcinoma using cuproptosis-related lncRNAs
Source: BMC Bioinformatics. 2023 Mar 6;24:81. doi: 10.1186/s12859-023-05192-5 (PMC9990240; doi:10.1186/s12859-023-05192-5)
Supplement: Supplementary file 2 — Additional file 2. The result of univariate Cox regression analysis. [file 12859_2023_5192_MOESM2_ESM.pdf]

Table S2 the result of univariate Cox regression analysis

id	futime	fustat	AL139385.1	NIFK-AS1	AL161725.1	AL031432.4
TCGA-55-A4DF	1.2054795	1	0.1294809	2.1509495	0.8040536	1.3467584
TCGA-62-A471	3.4136986	0	0.3325074	1.6458861	0.1216786	0.7740807
TCGA-L9-A5IP	0.1589041	1	0.9000263	1.7285301	0.1066163	0.3256167
TCGA-78-8662	9.2082192	1	0.2171066	2.2767051	0.7553149	0.1961034
TCGA-L9-A50W	1.2109589	1	0.0739573	1.9315318	0.7528342	1.3323929
TCGA-75-7025	9.0547945	0	0.2824991	2.3207446	1.3240041	1.8245235
TCGA-62-A46P	1.6273973	1	0.6816741	1.6230709	0	0.754973
TCGA-38-4627	3.1424658	1	0.6255511	1.8482373	0.1642714	0.2289726
TCGA-69-7765	0.4520548	0	0.2910141	1.6966171	0.7553149	0.1996252
TCGA-86-A4P7	1.1369863	0	0.0858325	1.926417	0.9755568	0.7417474
TCGA-97-8177	1.3671233	0	0.1662013	1.8217102	0.3643483	0.9801716
TCGA-50-6595	0.5178082	1	0.8471952	1.5531147	0.1175621	0
TCGA-55-A492	1.6328767	0	0.593306	2.0495959	0.3067289	0.8377011
TCGA-78-7633	4.1863014	1	0.4314625	2.7582819	0.3478924	0.2977197
TCGA-97-A4LX	1.6821918	0	0.4461503	1.6142858	1.1357319	1.4722278
TCGA-78-7220	2.2109589	1	0.8981696	2.2157097	0.458172	0.5693939
TCGA-50-8457	3.0821918	0	0.0931545	1.9480027	0.2995963	0.7201033
TCGA-91-8496	1.3835616	0	0	1.6978628	0.3700527	0.65764
TCGA-55-8092	0.4219178	1	0.5924451	2.1393385	0.579856	0.9804641
TCGA-69-8255	0.3534247	0	0.0654344	1.8317962	0.397803	0.9683488
TCGA-69-8253	1.1671233	0	1.151729	1.9430586	0.2273714	0.5031452
TCGA-86-A4D0	0.3178082	1	0.2947825	1.8528378	0.5628652	0.7217664
TCGA-78-8640	19.347945	0	0.1024566	1.8997943	0.3331947	1.2136272
TCGA-97-8174	0.4493151	1	0.1918153	2.4749551	0.6784325	1.2806
TCGA-49-6744	4.6109589	0	0.2256451	1.7873489	0.6812244	0.4640409
TCGA-44-7671	2.4356164	0	0.3410749	1.6638455	0.2058929	0.2760797
TCGA-86-A4P8	2.2054795	0	0.2075181	2.2345626	1.6520505	1.3137683
TCGA-78-7158	0.490411	1	0.0886844	2.2463169	0.7107899	0.8643347
TCGA-80-5611	7.109589	0	0.7330072	2.7610086	0.3163775	0.7044294
TCGA-44-4112	2.2136986	1	0.3704991	2.2070338	2.5752397	1.16308
TCGA-71-6725	0.7013699	0	0.2380531	1.3074285	0.2108875	0.6574571
TCGA-05-5715	0.169863	0	0.0508847	1.4943644	0.0821576	0.3431235
TCGA-05-4432	2.0849315	0	1.2919571	2.3566509	0.1493894	0.5938796
TCGA-78-7143	13.591781	1	0.978635	1.5716283	0.7920224	1.1819294
TCGA-73-4668	1.2794521	0	0.294312	1.4670186	0.1272371	0.8224446
TCGA-38-4625	8.1452055	0	0.444137	2.5611061	0.2143735	0.4252444
TCGA-91-8499	0.0986301	0	0.1806571	2.945308	0.3467584	2.066537
TCGA-44-6779	1.369863	1	1.1761951	1.8722378	0.1851054	0.1705661
TCGA-67-3773	1.169863	0	0.0939658	2.298629	0.4103411	1.1743425
TCGA-97-A4M2	1.709589	0	0.1505597	1.8169683	1.0535979	1.6866574
TCGA-78-7539	2.1671233	0	0.0863762	2.3810341	0.3524196	0.5242643

TCGA-67-3771	1.6712329	0	3.8253175	2.2606881	0.8330925	0.7069062
TCGA-55-8299	1.2849315	1	1.0303361	1.8804896	0.6820339	0.7303139
TCGA-44-A47A	1.2767123	0	0.1849785	2.1703417	0.7766094	1.5861162
TCGA-05-4402	0.6684932	1	0.4926223	1.8896293	0.2881814	0.458592
TCGA-55-7914	0.5123288	1	0.2605075	2.1938348	0.3020558	0.6998181
TCGA-49-4490	1.0547945	1	0.9483018	2.3033716	0.1589833	1.0107082
TCGA-55-8090	1.6383562	1	0.4941596	1.8163539	0.3911078	0.9887029
TCGA-99-AA5R	1.8027397	0	0.1889073	2.1718793	1.0977456	0.8768018
TCGA-49-4486	6.3506849	1	1.2226809	1.9117683	0.2066432	0.1705661
TCGA-91-6847	2.3068493	0	1.4599037	2.4293214	0.2987756	1.4414304
TCGA-91-A4BC	0.1205479	0	0.6663021	1.4491652	0.5918709	1.3754565
TCGA-55-7570	2.2575342	0	1.7671039	2.7696871	0.3668118	1.021835
TCGA-55-7727	0.3260274	0	0.9169352	1.5096452	0.7235149	1.611975
TCGA-44-2666	0.2657534	1	0.6767789	2.2703495	1.9733664	1.9992183
TCGA-62-8395	3.3315068	0	1.1207501	2.1639173	0.5229597	1.0215508
TCGA-05-4410	0.0027397	0	0.0870554	1.6275601	0.6623878	0.5505067
TCGA-55-6975	0.3232877	1	1.260387	1.7196654	0.2062681	0.2253983
TCGA-73-4662	6.890411	0	0.1696685	2.0787806	0.5662794	0.4028313
TCGA-44-A47G	0.9616438	0	0.267356	1.5695883	0.7199281	0.6014113
TCGA-NJ-A4YG	6.1945205	0	0.3916578	1.7037652	0.413919	1.0282862
TCGA-05-4433	2	0	1.1003722	1.833133	0.4325319	1.8117587
TCGA-55-A4DG	1.6657534	0	0.1235338	2.281075	1.0773797	1.4518046
TCGA-NJ-A7XG	1.690411	0	0.1773438	2.4687397	0.2788174	1.8241161
TCGA-44-6147	2.3150685	0	0.3203884	2.119489	2.4433768	1.8674748
TCGA-49-AARO	10.29863	0	0.434348	1.5228593	0.363115	0.933346
TCGA-44-6774	1.8027397	0	0.2815499	1.9414444	0.675274	0.254715
TCGA-50-8460	2.2712329	0	0.2211966	2.6739182	0.3826119	0.6949248
TCGA-49-AARE	3.3671233	1	0.2980717	1.6417772	0.2613503	0.9896477
TCGA-55-6642	6.709589	0	0.5169229	1.4556494	0.4511188	0.3373115
TCGA-78-7166	0.7068493	1	1.8579412	1.2649567	0.0787464	0.2872359
TCGA-05-4427	2.1671233	0	0.4841897	1.9730234	0.7654499	0.6322682
TCGA-49-4507	0.7342466	1	0.9619196	1.7248685	0.1746621	0.5423076
TCGA-78-7148	1.7150685	1	2.2553799	1.772308	0.1759397	0.2263852
TCGA-55-8514	1.4246575	0	0.03492	1.8412888	0.3993352	0.4494293
TCGA-55-7576	1.8356164	0	0.3095255	1.8586176	0.2803625	0.3222743
TCGA-55-6969	3.3945205	0	0.3449421	1.6006979	0.4630993	0.403595
TCGA-91-6830	0.1643836	0	2.2277103	2.3340247	0.9437338	1.0201999
TCGA-78-7540	3.2794521	1	0.7429548	1.572065	0.144699	0.3458505
TCGA-MP-A4T6	4.9041096	1	0.1745343	2.4852722	1.0130692	2.3085939
TCGA-99-8028	3.0630137	0	0.138028	1.6382129	0.2927817	0.2250281
TCGA-05-4403	1.5835616	0	0.849599	1.3711127	0.0716254	0.2279875
TCGA-78-7167	7.3452055	1	0.2893623	2.9411814	0.7651952	1.1192234
TCGA-55-8615	1.2219178	0	2.102591	1.3901724	0.1016501	0.7963486

TCGA-55-A494	1.3178082	0	0.3345683	2.2154301	0.549817	0.3994446
TCGA-78-7147	1.6054795	1	0.5995082	2.3720606	0.2655569	1.2439128
TCGA-97-A4M0	1.7863014	0	0.0659858	2.1608557	1.1866907	1.4938522
TCGA-64-1679	6.8164384	0	0.6912661	1.629986	0.3078948	0.2981891
TCGA-64-1680	3.0849315	0	0.6204926	2.2335813	0.0724489	0.5580717
TCGA-86-8669	2.569863	0	0.9697491	1.8432201	0.5496199	0.9887756
TCGA-78-7145	2.2630137	1	0.2758414	1.2555007	0.2286032	0.3447149
TCGA-99-8025	2.9041096	0	0.154972	2.3296977	0.2161134	0.6715651
TCGA-L9-A743	1.8191781	0	0.2994791	1.5395807	1.2210728	0.4271771
TCGA-55-8094	1.4821918	0	0.5209502	1.6663476	0.2040155	0.7491481
TCGA-86-A456	2.4547945	0	0.1486088	1.5999366	0.7337882	0.6483734
TCGA-44-3919	2.8109589	1	0.7669767	1.9068137	0.7254358	0.5353563
TCGA-69-7973	0.630137	0	0.2055176	2.2128494	0.4974335	0.4975357
TCGA-35-4122	0.6164384	0	0.5356549	1.3888508	0.0958569	0.2997136
TCGA-35-4123	0.4986301	0	0.5938796	1.9149854	0.2178511	0.7939375
TCGA-55-6982	2.7260274	1	0.1493894	1.3021728	0.340961	0.406972
TCGA-44-A4SU	1.1205479	1	0.417704	2.1147335	0.8644932	1.8909914
TCGA-86-8279	2.6	0	0.4200781	2.6038578	0.2747686	1.245374
TCGA-05-4396	0.830137	1	2.5643538	1.8861209	0.2242873	1.1192898
TCGA-44-6777	2.7041096	1	0.206018	1.2567088	0.514804	0.3358262
TCGA-55-8301	1.4630137	0	0.1405169	1.5856356	0.5384388	0.9550896
TCGA-05-4422	1	0	0.7017708	1.710305	1.0213376	0.9408057
TCGA-05-4417	1.2465753	0	0.1746621	1.3309599	0.3946239	0.4694693
TCGA-75-5146	6.4876712	0	0.3975839	2.4018216	0.5254676	0.7343086
TCGA-97-8552	1.7150685	0	0.1645289	2.1728395	0.6870607	1.2234843
TCGA-MP-A5C7	6.1589041	0	1.0766959	2.3208601	0.813853	1.4528063
TCGA-97-7937	1.5452055	0	0.2343787	2.1149333	0.2471984	0.7755142
TCGA-97-8175	1.509589	0	0.7273542	1.5070081	0.1086248	0.563451
TCGA-97-A4M7	1.7232877	0	0.0976108	1.7589001	0.727267	1.4597988
TCGA-05-4420	2.4986301	0	1.4837255	1.8126214	0.4565959	1.092681
TCGA-91-8497	1.1890411	1	0.4958998	2.1644323	0.6591939	1.1541943
TCGA-38-4632	3.7178082	1	0.0469799	1.6224619	0.1123665	0.3752897
TCGA-78-7163	19.857534	0	0.3119681	2.5607883	0.2072682	0.8049622
TCGA-MP-A4TA	2.6027397	1	0.90589	2.7832471	0.487795	1.3724507
TCGA-50-5931	1.1890411	1	0.7648557	3.0737346	0.2015084	2.0853567
TCGA-55-7725	1.2109589	0	0.1159654	2.2884767	1.2414741	1.000577
TCGA-MP-A4SY	4.1123288	1	0.5547362	1.7973866	0.754973	1.0846766
TCGA-78-7154	1.6246575	1	0.6977738	1.7887275	0.2251515	0.3893465
TCGA-73-4659	1.9479452	1	0.2277411	1.6257381	0.2290957	0.6379348
TCGA-69-7980	1.1260274	0	1.0963968	2.6375407	0.5430999	0.9693808
TCGA-73-7498	3.2575342	0	0.4846022	2.5056369	1.1228052	1.3463045
TCGA-78-7542	0.8794521	1	0.7834147	1.5897156	0.1508196	0.46634
TCGA-05-4250	0.3315068	1	0.2095158	1.9805006	0.1175621	0.2087669

TCGA-38-4630	2.939726	1	0.2636354	2.910771	0.769264	0.9943625
TCGA-05-4418	0.7506849	1	0.3043942	1.21456	0.1042024	0.4010843
TCGA-55-8085	2.4767123	0	0.9465812	2.1519562	0.4742543	1.2286648
TCGA-55-7995	2.4356164	0	0.2954879	1.6830227	1.0608775	1.6027419
TCGA-55-7284	0.6657534	1	0.531369	1.7878921	0.4601659	0.7962655
TCGA-55-6979	0.6493151	1	0.9195308	1.5132886	0.6060631	0.7719701
TCGA-62-A472	2.4931507	0	0.1988713	2.0760802	0.3572705	1.4292678
TCGA-62-8398	1.2164384	1	4.091082	1.7126398	0.6329196	0.272501
TCGA-MP-A4TI	1.1753425	1	0.5731806	1.7083638	0.4610046	1.6189434
TCGA-44-8120	0.7123288	0	0.3551291	2.9218362	0.3571578	0.3721721
TCGA-55-5899	2.5479452	0	0.516116	2.338025	0.3564819	0.4705108
TCGA-MP-A4TK	1.5945205	1	0.2864081	1.587893	0.5574836	1.1131671
TCGA-73-4666	2.1917808	0	0.1914363	1.8062828	0.5597365	0.9347052
TCGA-78-7536	0.6684932	1	1.6734207	2.1740868	0.5831339	0.9038096
TCGA-55-7281	2.3890411	0	0.5941663	1.851759	0.239398	0.220949
TCGA-97-7547	5.3835616	0	0.2638757	1.9694176	0.3903375	0.4433945
TCGA-62-8394	0.3808219	1	0.7639214	1.7466567	0.4272843	0.3610946
TCGA-86-8668	1.1589041	0	0.1541943	2.3610946	0.3853758	1.2986583
TCGA-50-5939	1.260274	1	1.45191	1.4259963	0.2428159	0.3652446
TCGA-38-7271	2.1917808	1	0.1523781	1.5662307	0.5172253	0.2727399
TCGA-49-AARQ	18.443836	0	0.19131	1.8927407	0.4016305	2.0217995
TCGA-64-1678	3.2575342	0	0.0732718	1.9327415	0.1998765	1.0085586
TCGA-49-AAR3	5.1863014	0	0.4975357	1.5274207	0.4430762	1.0389278
TCGA-05-4395	0.0027397	1	0.8854963	1.9119216	0.2123826	0.7521491
TCGA-44-A479	1.3315068	0	0.3228511	1.5309197	0.8368129	0.9429836
TCGA-49-4514	4.6575342	0	0.417488	1.5301706	0.1384213	0.2121335
TCGA-62-A46S	4.5287671	1	0.8119642	1.6932305	0.5257682	0.764516
TCGA-55-A490	0.2712329	1	0.0263043	1.1852323	0.3788445	0.5808208
TCGA-97-7938	0.0493151	1	0.6128714	2.1881477	0.7662135	0.1994996
TCGA-55-8097	1.3041096	0	0.3092926	2.4683748	0.4872805	0.9873209
TCGA-44-3917	3.2410959	0	0.4505382	2.3708896	2.886872	1.4655305
TCGA-05-4415	0.2493151	1	0.1682569	1.7066853	0.3810618	0.4537546
TCGA-44-6145	1.630137	0	2.0450235	1.7376435	0.6608374	0.4954905
TCGA-44-7667	3.0054795	0	0.2309408	2.4742024	0.059909	0.3327366
TCGA-97-7553	5.1232877	0	0.114367	2.0217995	0.6046407	0.5479433
TCGA-78-7150	1.8246575	1	1.5610572	1.8387905	0.0576929	0.2470768
TCGA-69-7763	1.890411	0	0.5386375	1.8263146	0.3467584	0.5130864
TCGA-95-7947	1.3068493	0	0.6636634	1.6869711	0.5022287	1.2738739
TCGA-86-7711	2.8657534	1	0.4117516	1.6314768	0.2741722	0.5061957
TCGA-49-4501	3.8931507	1	0.3278023	2.4113449	0.3278023	0.5705601
TCGA-L9-A8F4	1.3041096	0	0.1129003	2.1841216	0.986957	1.5709972
TCGA-MP-A4T9	3.4657534	1	0.8545543	1.7956007	0.3703875	1.0284277
TCGA-91-6835	0.2164384	0	0.297837	2.5512453	1.5318181	1.2708877

TCGA-50-6590	3.5287671	1	0.9665043	1.8273313	0.2865263	0.3195023
TCGA-95-7039	3.4849315	0	0.4685312	1.2262002	0.3286067	0.6537021
TCGA-75-6206	7.0958904	0	0.555129	2.4241965	0.4043582	0.8552722
TCGA-50-5051	1.309589	1	0.2390314	1.7035437	0.2493847	0.8540755
TCGA-97-7552	5.2931507	0	0.5144	1.8852229	0.8145094	0.8084262
TCGA-44-2657	3.7013699	0	0.1697968	2.297837	0.2468337	1.1660727
TCGA-91-6840	1.0191781	0	0.3070787	2.1673258	0.6888517	1.0029545
TCGA-55-7907	0.939726	1	0.1330374	2.0827026	0.3361691	0.4187836
TCGA-49-4510	2.4547945	1	0.3128975	2.4302317	0.2047668	0.8658397
TCGA-55-7726	1.7863014	0	1.5748766	1.998701	0.0440443	0.7505208
TCGA-35-3615	0.0383562	0	0.2597847	1.9443337	0.3574957	0.8709762
TCGA-97-A4M6	1.5561644	0	0.5093411	2.082975	0.5647196	1.8197499
TCGA-53-7813	1.1616438	0	0.3229665	1.8211387	0.5845777	0.6242413
TCGA-78-8655	6.4657534	0	0.2651968	1.9972563	0.2195867	0.5383395
TCGA-L9-A444	0.8410959	0	0.3123167	1.2918982	0.8088381	0.9889936
TCGA-86-8055	0.339726	1	0.5039593	2.0259147	0.3476657	0.8477564
TCGA-49-6745	1.430137	0	1.2053925	1.4276062	0.3534362	0.2775087
TCGA-MP-A4TH	2.030137	0	0.2346239	2.146753	0.9101186	1.7165081
TCGA-99-7458	2.0465753	0	0.2794119	2.1324452	0.8345494	1.1355349
TCGA-55-1594	3.2273973	0	0.8266807	2.3415019	0.2057678	1.5232106
TCGA-55-7913	1.5369863	1	1.5340616	1.9210554	0.0893626	0.4960021
TCGA-95-7944	1.0328767	0	2.7244975	1.762774	0.7222037	0.8488783
TCGA-05-4398	3.9205479	0	0.5428028	1.4616333	0.6753643	0.6098495
TCGA-78-7161	0.7972603	1	1.1253205	2.0398753	0.2600257	0.7081431
TCGA-50-5933	6.5561644	1	0.4208327	1.4302317	0.4342412	0.1865006
TCGA-75-5147	3.6520548	0	0.236462	1.8878372	0.4872805	1.8395565
TCGA-78-7160	1.909589	1	1.5669125	1.7881428	0.8274939	0.2976023
TCGA-62-8402	4.1041096	1	0.4691566	2.1884326	0.5858279	0.9109629
TCGA-78-8648	3.3123288	1	0.1640139	1.336569	0.4152178	0.4432884
TCGA-44-3918	2.8383562	0	0.2369926	2.3632739	2.7371315	1.5155779
TCGA-55-A57B	1.4958904	0	0.3839393	2.0604279	0.8661563	0.9767302
TCGA-38-6178	1.2273973	0	0.3598585	2.0818509	0.2537473	0.8285503
TCGA-78-7535	2.6	1	0.6852674	2.3312753	0.1182268	0.715542
TCGA-91-6849	0.0958904	0	0.3630028	1.7275284	0.5230601	0.5133897
TCGA-55-7903	1.5534247	0	0.0972062	2.0172449	1.2288495	1.0537369
TCGA-55-A493	0.0767123	0	0.1665869	1.4540179	0.7640064	1.1181604
TCGA-50-5932	3.3835616	1	0.3703875	2.0451284	0.0519985	0.5233612
TCGA-05-5423	0.4136986	0	0.0827026	1.857583	0.0677764	0.3879139
TCGA-62-A46Y	1.1342466	1	0.2602666	2.1198541	0.8087557	1.3830545
TCGA-97-A4M1	1.6465753	0	0.1706942	2.6322449	0.3673711	1.7824505
TCGA-95-7567	1.5561644	0	0.267356	1.8538759	0.1955996	0.2278643
TCGA-55-7910	2.8493151	0	1.9916806	2.0620526	0.6055892	0.697507
TCGA-95-8494	0.230137	0	0.3082444	1.8360049	0.661932	0.817787

TCGA-75-7027	8.3808219	0	2.3969815	2.1639495	0.1747899	0.3286067
TCGA-44-6775	1.9315068	0	0.8435283	2.1837829	2.955567	1.5857478
TCGA-55-1592	1.9205479	1	0.3373115	2.6495005	0.3970362	1.4055023
TCGA-44-2665	3.5643836	0	0.3501577	2.7483539	2.2183937	2.0222613
TCGA-55-A48Z	1.7835616	0	0.2538683	1.5585615	0.7159812	0.9835318
TCGA-86-8281	0.0027397	0	0.0982848	2.1977711	0.6416385	0.7969299
TCGA-J2-A4AG	2.7068493	0	0.1561377	1.8614393	0.5742469	0.3290662
TCGA-78-7152	3.3287671	1	0.2878269	1.5189883	0.4047942	0.3379965
TCGA-93-7348	1.4547945	0	0.23842	1.6117862	0.4500631	0.7696871
TCGA-64-5775	0.169863	1	0	1.357552	0.090718	0.4402081
TCGA-78-7153	9.9589041	0	0.3189242	1.4807304	0.1487389	0.6232114
TCGA-MP-A4T8	0.4410959	1	1.4635701	1.1471113	0.217603	0.5631581
TCGA-44-2661	3.1753425	0	0.063779	1.7868891	0.3589588	1.0728604
TCGA-73-4658	4.3835616	1	0.1806571	1.4639363	0.3343394	0.1106305
TCGA-44-6778	5.1068493	0	0.2624332	1.9769501	1.1871343	1.0751903
TCGA-44-7670	2.4164384	0	0.6779817	3.3186928	0.3319345	0.8617172
TCGA-55-8205	1.6410959	0	1.0510936	1.763794	0.811471	1.1691554
TCGA-55-8512	1.6630137	1	0.9177754	2.0860364	0.4145685	1.5728412
TCGA-55-8203	1.4986301	0	0.5365503	2.0705954	0.4018489	0.9325148
TCGA-44-A47B	0.7863014	0	0.6035017	1.8415706	0.5091384	0.4730076
TCGA-64-5778	3.5753425	0	0.7114068	2.7036766	0.5046712	1.7242574
TCGA-86-7954	1.6575342	0	0.4004286	1.9698596	0.9449334	1.2399479
TCGA-55-6543	1.1917808	0	0.147437	1.5073126	0.4338141	0.6441332
TCGA-62-8399	7.3863014	0	0.1683853	1.83289	0.3626663	0.5176284
TCGA-49-AAR0	13.054795	0	1.1522483	2.3970636	0.3207735	1.6770346
TCGA-38-4626	10.065753	0	0.44297	1.688807	0.5654997	0.6201172
TCGA-95-7562	0.2383562	1	0.9174699	1.6429788	0.1918153	0.1919416
TCGA-69-7760	0.5534247	0	0.2353594	1.3072536	0.153546	0.3389093
TCGA-55-7574	2.7260274	1	0.1840898	1.8181961	1.5830858	1.500904
TCGA-95-7043	1.3780822	1	0.1240634	1.5054337	0.0347792	0.8519189
TCGA-MP-A4T7	0.4575342	1	1.827819	1.859254	0.4666532	0.418136
TCGA-95-A4VN	1.5150685	0	0.2841587	1.3953919	0.7280511	0.5733745
TCGA-93-8067	0.509589	0	1.6285403	1.3550163	0.4022856	0.2823805
TCGA-86-7713	3.169863	0	0.2861715	2.499476	0.3857071	0.7069062
TCGA-50-5044	1.709589	1	1.4594316	1.212009	0	0.3507235
TCGA-64-5774	7.3315068	0	0.5914879	2.5934016	0.2381754	0.3373115
TCGA-55-6712	0.4684932	1	0.8236679	1.360589	0.6768992	0.6554434
TCGA-4B-A93V	0.8219178	1	0.3288365	1.5191896	0.2311866	0.8539956
TCGA-83-5908	2.2575342	0	0.5226584	1.6560844	0.8624316	0.8074374
TCGA-44-A4SS	1.1369863	0	1.7194901	1.4833128	1.0697022	0.7179999
TCGA-L4-A4E5	1.5835616	0	0.1508196	1.5265196	0.4895429	0.8880711
TCGA-97-8171	1.5561644	0	0.0648829	1.963844	1.0020184	1.461057
TCGA-55-6978	0.4821918	1	0.3450557	1.6380738	0.9469554	0.4892346

TCGA-78-7146	0.4739726	1	0.729444	1.3972553	0.254594	0.305562
TCGA-50-5930	0.7726027	1	0.1130337	1.8611614	0.1369788	0.3832757
TCGA-64-5781	4.2712329	0	0.96857	1.3827779	0.1595	0.1469158
TCGA-78-7159	5.4082192	0	1.6726969	2.1281614	0.3191555	0.97717
TCGA-69-8254	1.1205479	0	0.2319239	1.5768607	0.4341345	0.7170348
TCGA-NJ-A4YP	0.1369863	0	0.5522787	1.7774513	0.276318	0.5505067
TCGA-44-8119	0.7808219	0	0.1843438	2.1713349	0.4445611	0.2050171
TCGA-91-6836	1.1424658	0	0.596649	1.9940728	0.3928669	0.1063483
TCGA-49-6743	4.4410959	0	0.5832302	1.4890291	0.2392758	1.0816805
TCGA-44-7662	0.5972603	0	0.5618882	1.7447208	0.5623768	0.4711354
TCGA-44-2655	3.6273973	0	1.3337672	2.1659119	0.2481705	0.3702759
TCGA-86-8673	2.3616438	0	1.0972062	2.2454044	0.1906781	0.6022668
TCGA-MP-A4TF	0.9205479	1	2.493468	2.3037515	0.229957	1.369941
TCGA-55-7911	1.4712329	0	0.2927817	2.8463128	1.0978804	1.3609261
TCGA-75-6214	3.0547945	1	2.4055295	1.2980131	0.2408638	0.4542812
TCGA-05-4244	0.0027397	0	0.1427402	1.9289198	0.421156	0.9505431
TCGA-05-4382	1.6630137	0	0.2053925	2.0128547	0.458382	0.5712399
TCGA-J2-A4AE	2.9561644	0	0.5679348	1.3706107	0.5326661	0.5445843
TCGA-73-4670	0.3589041	0	0.4204016	1.6999957	0.1110313	0.2379308
TCGA-55-8506	0.030137	0	0.450591	1.4785577	0.2983064	0.2757223
TCGA-44-3398	3.1863014	0	0.2513252	1.6972845	0.2078929	0.8140171
TCGA-73-A9RS	0.9315068	1	2.2375637	2.0909889	0.3081278	1.1683853
TCGA-55-8505	1.2054795	0	3.1090262	1.7903133	0.1828194	0.9007992
TCGA-97-A4M5	1.7369863	0	1.4849115	1.8467942	0.6283537	1.1932671
TCGA-75-5125	5.5534247	1	0.3577209	1.5043153	0.4508022	1.2036397
TCGA-62-A46U	5.6630137	0	0.2653169	2.051024	1.2452523	0.6193661
TCGA-91-6829	3.4465753	1	0.5091384	2.0590783	0.4832612	0.7904384

GLIS2-AS1	AL022098.1	AP002026.1	AC087854.1	AP000302.1	FMR1-IT1	AL512363.1	AC012557.1	AC026202.2
1.0906502	0.4648773	2.2169825	0.867343	0.5473511	2.8169887	0	4.1163814	1.0996992
0.6992851	0.0703893	1.1236662	0.3514022	0.3987881	1.5133391	1.1415636	0.9452331	0.7960162
0.2402533	0	0.1571731	0.1637563	0	1.3876934	0.7956838	0.9316831	1.248049
0.3923174	0.8690818	1.1033969	0.2509616	0.5643294	1.9215506	0.446468	1.0310422	0.707083
3.1021374	0.5452765	0.8505594	0.4373343	0.3313613	1.417488	0	1.0880059	0.8898629
2.0673978	0.7277027	1.495337	0.7831633	0.2779847	2.6061816	0.4027222	2.3038392	1.178619
0.872159	0.0626744	0.6578229	0.5449799	0.646347	1.0686708	0	1.1584663	1.422556
0.7077015	0.1394695	0.4196468	0.4790236	0	0.3937457	0	0.9774631	1.4657656
0.9679064	1.0244615	0.7604339	0.6120222	0.1660727	1.8504394	0	1.582556	0.6822137
1.5694911	1.0513025	1.1234013	0.886355	0.1381591	2.0430645	0	2.3052118	1.8829951
2.5433721	1.3728407	1.1350094	0.5758932	0.0930192	1.5562087	0	0.9591766	1.0403314
0	0.0753273	0.3653566	0.5660845	0	0	0.8285503	0.8025655	0.7455385
3.7076021	0.8802545	0.5062973	0.7620511	0.5323668	0.2305719	0	0.980391	1.6833371
3.5362643	1.0383659	1.4159209	0.8724742	0	0.9494976	0.2087669	1.0324535	1.5024834
1.1126334	0.668936	0.6519587	0.9814874	0.4808855	2.7156957	0	1.5021778	1.1218112
0.8312287	0.2476845	0.2775087	0.6414535	0	1.1936456	0.6805944	1.4512243	0.9410312
1.6251302	0.4018489	1.3253864	0.867343	0	1.2813125	0	0.8504794	2.0296649
2.3925922	0.5117716	1.7752191	0.3215818	0	0.6104166	0	1.5469562	1.2291572
1.3145805	0.6034068	0.7481177	0.4540706	0.46425	1.6832024	0	1.6722896	2.3912453
0.7005285	0.2890081	0.5398288	0.2835662	0.1849785	0.9147176	0.5002922	0.761881	1.9769868
1.4811956	0.7031893	0.8379432	0.4556494	0	0.8294437	0	0.5835191	0.9732072
1.0641241	0.2144979	0.962512	0.276318	0.3544522	1.3907227	0	0.9340258	1.0730661
0.3056787	0.4021764	0.9814874	0.3929768	0.1641426	1.2653169	0	0.9406554	1.2102018
1.5191896	1.7324863	1.1204183	0.9162473	0.08882	1.630685	0	1.8042188	1.2539288
0.2674759	1.3691035	0.2333973	0.6648469	0.1427402	1.857782	0.1611783	1.3064372	1.021835
1.1424788	0.2106382	1.527721	0.8012414	0.3000651	0.6767187	0	1.2289726	0.8186052
1.6447331	2.7314439	1.1665869	1.3246954	0.4067543	3.0667609	0	2.427338	1.0538759
1.1502997	0.1557492	1.8847151	0.5015156	0.3909978	2.0785074	0	1.1662013	0.5985558
1.1100291	0.0654344	2.3316766	0.2917214	0	1.444932	0	1.2930173	1.1191569
2.936393	3.3230458	2.5531762	2.796712	0.9260753	2.7438488	0.4513298	4.0929136	3.3843538
0.6146627	0.2136272	0.1563966	0.3876934	0	1.3671474	0	1.0246743	1.0056156
0.758559	0.2219389	0.5870769	0.3203114	0	0	0.4868688	0.2539893	1.0356239
0.5544415	0.293135	0.2654369	0.8658397	0.2380531	1.1118993	0	1.0306892	1.2041407
2.8219551	0.726744	1.5183337	0.9767302	0.6744607	2.9471986	0	2.2135961	0.8694766
0.9926959	0.1925729	0.3493653	0.4904674	0.0710762	1.3056204	0	1.4945692	0.486148
1.423363	0.2856981	0.7337882	0.3284918	0.1470461	1.1018517	0	1.7895623	0.7583032
1.6988852	0.141302	1.7479459	0.3403914	0	1.6901489	0.2463473	0.2370742	1.0953168
0.8545543	0.046002	0.1265765	0.3426685	0	0.8489584	0	0.7438165	0.2684344
1.5572875	3.1264443	0.6669383	0.6858056	0.4120769	1.1915627	0	1.6610199	1.4866114
2.4823578	1.9889573	1.1538053	1.1999393	0.8566272	2.4257547	0.3864799	2.2120401	2.1926045
1.3973101	2.8121901	0.4760187	0.5046712	0	1.8087557	0.1192898	1.2662168	0.7454525

1.7839176	0.3592963	1.8377415	0.8199951	0.187261	1.0541539	0	1.5675941	1.2471376
0.6588284	1.4659745	0.7731524	0.6165459	0	1.8016967	0.741316	1.0521376	1.0522768
2.5205228	0.4195389	1.7657045	0.7009723	0.4886178	2.0464911	0.2050171	1.384713	1.4495878
0.7680784	0.8324445	0.6164518	0.3905576	0.1407787	0.8451086	0	0.6913555	0.4921096
0.7092024	0.7615406	1.3857623	0.7965978	0.1479579	1.1754927	0	0.6679375	1.6514536
1.0230424	0.254594	0.6719274	0.2279875	0	0.7502635	0	1.3401065	0.7623913
1.5988416	0.4171639	0.7475163	0.6422857	0	1.8131552	0	1.269871	0.401412
1.547894	2.1448621	1.0460719	0.8288753	0.1351408	2.2672661	0	2.1804343	1.1164313
4.2161134	1.8543548	0.5625721	0.5162169	0.1416944	0.3790664	0	1.2666366	2.1625968
0.4461503	0.2137516	1.5065512	0.3338817	0.4541759	2.3591557	0.2752455	1.5928278	0.5442875
1.5275708	0.3341106	1.1751094	0.371057	0.3359405	1.8933234	0	1.3357691	1.7012385
1.1420213	0.2744108	1.8773124	0.3027577	0.5977934	1.2604473	0	0.417704	1.2778658
0.8911858	1.6429788	0.812375	0.7323995	0	1.6406209	0	2.1141005	1.7562974
2.7599654	3.4668533	1.668331	2.6034305	0.9092227	2.073249	0.6676651	3.9092963	3.1616083
1.8887335	0.4992719	1.147437	0.9129564	0.4608998	1.8795097	0	2.0982511	2.1233351
0.924328	0.2819059	0.6757256	0.516116	0	1.4749811	0	1.2216915	1.5696369
0.4467856	0.0816123	0.392757	0.4234168	0	1.0562367	0	0.8543947	1.1925729
0.9874664	1.5223069	0.450591	0.7345687	0.1525079	2.2595135	0	2.1183265	1.1111649
1.121546	1.8569857	0.6661203	0.486251	0.1375035	1.817746	0	1.3982409	0.760519
1.6220401	0.3322783	0.9266826	0.6893886	0.8679755	1.2468945	0	1.0199865	1.3326793
0.7554858	0.3512891	0.108491	0.7686713	0.23352	1.4358419	0	1.8028549	0.9033468
2.0788147	1.0601858	2.3102819	1.1375691	0.729879	3.4890805	0	2.6742573	1.2713659
1.7260028	1.3002995	0.6864333	0.7913557	0.6188025	2.9365343	0	1.6863885	0.8016553
2.1321162	3.4451661	2.0069921	2.9110332	1.2345013	3.2398206	0.2340107	3.9686284	2.876422
1.7986312	0.1247252	1.7967223	0.7421787	0.546166	1.6932751	0	1.5266197	1.1726155
1.166137	0.413919	0.7134319	0.5444854	0	2.1005068	0	1.2537473	1.1756204
2.1976453	0.5332643	1.1092937	0.4473147	0.1397314	0.4803685	0.3001823	1.5287213	1.6863436
1.6818091	0.2751263	0.6448715	0.3842709	0.1901723	1.4743062	0	0.6206802	0.7035437
1.213876	0.2946649	0.4518573	0.4595365	0.2539893	1.2066432	0	1.03492	1.9129947
1.3298125	0.1356662	0.9237957	0.1159654	0	1.3222166	0.0771062	0.3370831	1.1664584
0.9151002	0.7126398	1.0319597	0.4936473	0	1.8615584	0.2281106	1.4021218	1.4022856
0.5914879	0.0798389	0.1688987	0.1237986	0	0	0.6128714	0.2255217	1.7480318
0.7281382	0.0977456	0.2371966	0.5092398	0	0.5198438	0	0.8893957	1.3504972
1.4047942	0.2262619	1.7892702	0.4204016	0	2.8210775	0	1.85591	0.5620836
0.2990101	0.2216915	0.3106891	0.3198491	0.0824301	0.9358369	0.3416442	0.9716631	0.6081469
0.5017194	0.2016339	0.4318903	0.4936473	0.1456122	1.0219061	0.0844725	0.7105254	2.2592122
0.4746697	1.0216219	0.886277	1.1692837	0.2617113	2.0318892	0	1.9328927	1.9842612
0.458382	0.4218023	0.2537473	0.5612039	0	0.8991756	0.1949696	1.2128806	0.5275708
3.9870116	0.4821255	1.9682014	1.3585088	0.5375446	3.5477459	0	1.982802	1.48011
0.5687131	2.0650208	0.5570914	0.2872359	0.1677433	1.1357319	0.0977456	0.7985897	1.2663968
1.2035771	0.3931965	0.1844707	0.3083609	0	0.2614706	0.4927249	0.9622158	0.5918709
1.7134759	0.1852323	1.8499592	1.0612923	0	1.9875755	0.1146335	2.2646565	1.287886
0.7199281	0.1195554	1.0943712	0.3630028	0	2.0728261	0	1.0567222	1.3988976

1.7150148	0.1502997	0.6599246	2.5812548	0.1589833	2.7832052	0	2.5428771	1.3879691
0.9396779	1.2439128	0.9632522	0.3261922	0.2479275	2.1564937	0.1460035	1.2812531	1.2591821
1.8875253	1.8608834	0.5043662	0.6180507	0	3.3739544	0	1.8090439	1.0965317
1.220949	0.2789363	0.4460444	0.5757964	0	0.9104257	0	0.9300182	1.0856966
0.9356106	1.5488805	0.4857359	0.4717597	0.3902274	1.3264798	0.2345013	0.8847933	1.3444308
0	0.4778845	0.422233	0.7113187	0	1.9475914	0	1.0859685	1.2977197
0	1.064331	0.1443075	0.2143735	0	0.8965431	0	1.8397984	1.3021143
1.2410469	1.3576083	0.7489764	0.2852246	0.0970714	1.8888893	0.2118844	1.6783875	1.3712243
1.1984313	1.6482814	0.986957	0.5562087	0.2496274	1.7586443	0.1470461	2.169925	0.7270056
1.7620511	0.3645724	1.1153662	0.4119685	0.3843814	0.4803685	0.1578198	0.8289565	1.7740807
1.499425	2.7509066	1.3580023	0.4289999	0.8476763	2.3617964	0.3817264	2.2992447	1.6983074
0.974309	0.9875392	0.5362519	0.5654997	0.276318	1.6716104	0	1.6185206	1.2171066
0.9138755	0.5695883	0.5137939	0.6657566	0	1.2107006	0	1.2593629	1.3592963
0.8569459	0.3822799	0.2585792	0.1338265	0	0.1814206	1.4045762	0.379621	1.2015084
0.2327835	0.1166309	0.6884937	0.3012364	0	0.760519	0.9366662	0.908198	0.7728147
1.0353424	0.9450083	0.4150014	0.323543	0	1.623305	0.6861643	1.2404975	1.162919
1.905582	0.2244108	1.5389354	0.6684823	0.580049	3.3173475	0	1.5742953	1.8543149
1.5154097	0.9117299	0.6305919	0.4431823	0.3070787	2.1799887	0	1.4906215	1.2217534
0	0	0.6571827	0.6316165	0.6858953	2.3044818	0	1.6833371	1.9318343
0	2.2336427	0.4577519	0.4576468	0	0.5041627	0	0.865523	1.598651
0.3574957	0.7927721	0.5515898	0.5368487	0.1935825	0.9823639	0	0.9553128	1.9697123
1.9860832	0.4302853	1.603834	0.8156574	0.5714341	1.8564679	0.0859685	1.4980466	2.0180644
0.9933483	0.3979125	0.3056787	0.5781175	0	0.7962655	0	0.9744559	1.2501127
0.9380223	2.4726437	0.4729036	1.0330177	0.3914378	0.9319856	0	1.6819889	2.0319245
2.6657793	0.803227	1.4651909	1.0199154	0.5603237	2.1557169	0.3444877	1.4909295	1.2696915
3.2642812	0.2210728	3.2116664	0.8626696	0.9758503	2.6531747	0	2.4090379	2.5765705
2.2435777	0.4370146	0.6858056	0.7609448	0.1199537	3.0714366	0	2.1067503	0.8263553
1.1576905	0.2448871	1.256588	0.3664761	0.1091599	0.9374951	0	0.3298699	0.6984852
1.4239008	0.8872913	1.1908677	0.5326661	0.1188913	2.939866	0	1.5226082	0.674551
0.3782897	0.0896337	0.6268596	0.1654296	0.1401242	0.760008	0	0.9183099	1.4161912
1.0374525	0.7071714	0.9316074	1.0641241	0.343351	1.626112	0.2050171	1.9192638	1.5180315
0.3994446	1.2817279	0.4606901	0.3182301	0	0.3963787	0	0.4382929	1.7045622
1.4111008	0.092884	1.2901295	0.3297551	0	1.7938127	0	0.798009	2.5113668
1.0681205	0.0209111	2.0905486	0.2162376	0.1286893	1.2924873	0.3985693	0.7756827	1.1983056
3.076713	0.0404015	1.4934424	0.4144603	0	2.1732554	1.402613	1.2506585	0.6205864
2.8162515	3.7263626	2.482332	1.5101518	0.6933197	1.7639214	0.5533605	2.622087	1.5031452
1.2349304	0.1486088	2.215368	0.7956007	0	2.0464911	0.6074842	1.9588798	0.4249221
0.5838079	0.4851176	0.5720165	0.3355976	0	1.1610492	2.8233214	1.1693478	0.8132784
0.7463988	0.5664742	0.555718	0.3112706	0.0695647	0.6559929	0	1.0547096	1.3324502
1.0565142	0.4287856	0.6349649	0.6042611	0	1.5033996	0	1.4868688	0.3919876
2.3894567	2.4288392	1.8824083	0.7556567	0.2665167	1.9099267	0	2.0343566	1.7424806
1.1088255	0.1729995	0.5056877	0.3101074	0.2885358	1.1952846	0.2237932	0.568324	0.729792
1.417704	0.1814206	0.5782142	0.4242772	0	1.1109645	0.6616584	1.3151604	0.5088343

1.7704485	0.0680516	1.9490493	0.3560311	0.5169229	2.2342867	0.3559184	1.4565959	2.7646434
0.1352722	0.1509495	0.3369689	0.3281471	0.138028	0.5725987	1.6632991	0.4094724	1.5310695
1.9908097	0.3334237	2.2286032	0.5419113	0.4999862	1.770829	0.2102642	1.6000318	2.3517979
2.6768541	0.5065004	1.3074868	0.7527486	0.3759569	2.4918018	0.1171631	1.3910528	0.6006504
1.2493847	1.3753453	0.6113613	0.9740887	0.2095158	1.8395162	0.697418	2.0235393	0.82725
0.3426685	0.9758503	0.69635	0.7113187	0.1851054	0.8458312	0	1.1333005	1.3938555
2.3970636	1.8104842	0.9286546	0.8369744	0.5695883	2.6252705	0	2.1473067	0.3102238
1.7205849	0.2057678	0.7309225	0.4318903	0	1.2187812	1.077243	0.6156988	1.4553864
0.3185772	1.1032626	1.0617071	0.641546	0.58938	2.6754772	0.1933302	1.1618877	0.7005285
0.3961595	0.6849982	1.2179131	0.582845	0	1.3595774	0	1.2133784	1.5452271
0.3515153	0.1222089	0.7770304	0.2609891	0	0.6295665	0	0.2061431	1.7542462
0.9712952	0.3353689	0.9505431	0.4333868	0	1.9472921	0	1.2568899	1.895923
1.8457108	0.1918153	0.4882065	0.3467584	0.0972062	0.8576626	1.0214087	0.6851777	1.1657512
0.7520635	0.0769695	0.4170559	0.5548344	0	1.2365845	0.2126316	1.4196468	1.3742883
0.7134319	0.5130864	0.3567073	0.1454818	0.1491293	1.4280888	0	1.0481664	0.6223682
1.8890841	0.1683853	0.3467584	0.9477036	0.1356662	1.3400495	0	1.3852654	0.4760187
2.089871	0.2578555	1.1011122	0.3060289	0	1.6869263	0.3059122	1.0139981	0.3884651
2.6837863	1.4688439	1.2893033	0.7310094	0	1.0535284	0.8792352	2.0773456	2.1625323
0.3887957	0.5322671	0.2245343	0.6611111	0.2115107	0.3858175	0.2381754	0.6006504	1.0672945
0.3426685	0.7911056	0.2711866	0.8948372	0.1851054	0.845751	0	1.1331689	0.7469147
0.203765	0.0517201	0.932288	0.4080596	0.5504082	1.8898629	0.2338881	0.8151655	1.5826042
1.0019463	0.3346827	1.6128714	0.1695402	0.3288365	1.316725	0	0.3550163	1.6581886
0.8869013	0.0640551	0.8242383	0.4599561	0	2.3010901	0	1.1763866	0.3633393
0.1508196	0.0126402	0.4594316	0.0517201	0	0.2851063	1.0613615	0.5758932	0.8768018
1.0801119	0.3758457	0.4189995	0.8319583	0	1.8387501	0	1.0953844	1.8602477
1.1309309	0.1133004	0.5023306	0.2137516	0	0.4189995	0.6706591	0	0.3311319
2.2656469	0.5299708	0.9165531	0.96857	0.7714631	1.9052354	0	2.0960931	1.154648
0	0.0798389	0.520548	0.3048615	0	1.0390682	0	0.374177	0.6214307
0.9672424	3.2891705	1.0487942	0.6444101	0.2172307	1.8174186	0.2445218	2.7374272	0.8523185
2.0073032	1.4962067	0.9557592	0.9605858	0.1021878	2.6820339	0	2.454676	1.1616297
3.0053461	2.7804886	1.5420847	2.8961556	0.872159	4.0279635	0.1032626	4.7185945	3.618409
0.7775354	0.5055861	0.4397827	0.3067289	0.3139424	1.5299708	2.5445101	1.1323794	0.9734276
0.3379965	0.5089357	0.4300711	0.5527705	0.1824381	0.8358432	0.9402795	0.7944367	0.8490385
0	0.1292171	0.1054099	0.1396005	0	0.4128357	0	1.0163537	0.9486008
0.6736469	1.2430597	0.6480052	0.8483175	0.2660369	1.4697297	0.4279279	1.5786489	0.7677395
0.3111543	0.0275787	0.7458827	0.1631122	0.3169567	0	2.6883818	0.4872805	0.7912723
1.9928409	0.5987463	0.4478436	0.8261925	0	1.606537	0	1.1030612	0.8575033
2.0572423	0.7078781	1.0920044	0.5803385	0.3436921	2.1643036	0	1.0532504	1.5192399
0.160533	0.1547128	0.5506052	0.2806	0.0842004	0.6630258	0	0.5444854	0.674099
2.4790494	0.8009103	0.5665716	0.5666691	0.0799754	0.914488	0	1.242572	1.5458201
1.5265196	0.646347	0.8789999	0.6364506	0.1110313	3.6072474	0	1.5016684	1.8861209
1.6601528	1.6054469	0.622087	0.7895206	0.2875905	2.401412	0.4605853	1.7674429	0.6291001
1.0726546	1.0277203	0.9917531	1.300768	0.1900459	2.1872293	0.7137838	1.25181	0.7636665

0	0.1322478	0.251204	0.4595365	0.1399933	0.672199	0.3005338	0.5303704	0.6833821
0.417596	0.5695883	0.4653999	0.4553338	0.2282338	1.4133774	1.1166974	1.6109835	0.8868232
1.2568295	2.0321714	1.3914928	0.9989898	0.4379734	1.2494453	0	1.2346852	1.7911473
0.3192711	0.950767	1.055751	0.7524061	0	2.5021014	0	1.4081684	1.0894304
0.9231111	1.0243906	0.4838803	1.0216929	0.2424501	0.2359721	0.6994628	0.8463128	2.0924104
1.9277448	1.2991274	0.7499204	0.6458861	0	1.4788165	0	0.6391395	1.5488312
1.0984869	0.2046416	0.6344074	1.2684344	0	2.7348288	0.2877087	1.8239531	1.1076209
1.1632411	0.1036655	0.5773442	0.6144743	0.3965979	1.5075156	0	0.2295879	1.0687396
1.1835183	1.2987756	0.7215039	0.5940708	0.1482183	2.2885062	0	1.8217102	1.6025519
0	0.2696317	0.8984793	0.4267478	0	1.8243605	1.0444641	1.0137124	0.9460571
2.9784153	0.2278643	1.4248146	0.5707544	0.0848807	1.3384529	0	0.9929859	1.322332
1.0920721	0.1252543	1.4892346	1.1626612	0.299948	3.5292712	0.1779816	1.3991711	1.0219771
1.2273098	0.8093321	0.6301259	0.7879339	0.7524917	2.532691	0	1.5561106	1.893168
1.1983056	0.2407417	0.2341334	0.4788165	0	2.1792881	0	1.1768334	1.2079553
1.3103983	0.6563591	0.5930191	0.5301706	0.51783	2.0509892	0	2.1024902	1.0494914
0.7170348	0.2395202	0.6301259	0.5309696	0.3178829	1.0819531	0	0.5338623	1.0976782
0.3484591	0.6091876	0.4561753	0.6404358	0	1.2351756	0.5613017	0.9352334	0.1867541
1.8652854	0.6827531	0.4614238	1.4502215	0.3664761	2.765959	0.1499097	2.4363751	1.8776657
2.8757014	0.8002476	0.8609231	0.8213837	0.5234616	2.191152	0.3765127	1.983933	0.8320393
1.2681349	0.3675947	0.7367347	0.4392508	0.1410403	1.8451488	0	0.9227307	1.5530164
1.5298209	0.2032638	0.4791271	0.4156505	0.0897693	1.6417772	0	0.4330663	1.0134265
0.843019	0.1174291	0.6061579	0.4340277	0.1828194	0.4786095	0.8831516	1.8086734	0.3423272
1.0696334	0.2519311	0.9553128	0.4748773	0.4084945	1.7090259	0	1.7194901	0.8749936
0.9065828	0.7260465	0.9749698	0.7663831	0.1035312	2.7916682	0	1.8133605	1.9546802
0.3743996	0.998051	0.3587338	0.5118728	0	0.6666657	1.8395162	0.4112093	0.9256197
0.9432837	0.5714341	1.8148375	0.4636225	0.4893374	1.8003305	0.5449799	0.9110396	1.6786579
2.0862743	1.0943712	0.7850064	0.6665748	0	0.3702759	0	1.2102642	0.2007554
1.3098747	0	0.41738	0.3164933	0.462157	2.144438	0	0.6310577	1.4574367
0.946132	0.5316684	0.8043014	0.4007565	0.1613073	0.940054	0	1.2739336	1.93104
2.0126759	3.0051964	1.3574582	2.5117379	0.4832956	3.9036039	0.0885487	5.0486692	3.5740813
1.6782973	0.7700256	1.7568098	0.6762674	0.1290851	2.6900147	0.3996633	1.9088897	0.8009103
0.9325904	0.6950139	1.1480881	0.5128842	0	1.7012829	0	1.3317052	0.9407306
1.2087669	0.1651723	0.2972501	0.1669725	0.0724489	1.2630344	0	0.3569326	1.5555708
2.3587901	0.77989	0.953675	0.5425057	0.23352	1.2414741	0	1.2267551	1.0318892
1.9658028	1.5175781	1.1552959	0.2897164	0.1558787	2.1123665	0	1.9018034	1.6021718
1.7469147	0.1548424	0.9406554	0.6172985	0	1.2639358	0.4958998	1.2882995	0.674551
1.4506966	0.5201456	0.3837181	0.4428639	0.1513393	1.4426516	0	1.0601166	1.4615286
3.6595935	2.8813318	0.7057568	0.3937457	0.1952216	0.6440408	0	0.7064642	1.7214602
1.9853911	1.2666966	0.8903299	0.4460444	0.2820246	1.3026992	0.1669725	1.5271705	1.4948764
1.7731524	1.7263953	1.6587827	1.0949792	0.6934981	2.6764931	0	1.7936046	0.4934424
0.7947693	0.8280628	0.276318	0.2908962	0.7006173	0.8873693	0	1.1100959	1.0809305
1.7926472	1.3534362	0.4021764	0.4590119	0.2107629	2.3131878	0	1.245739	2.423363
0.7268312	0.3059122	1.1003722	0.1721034	0	2.9054664	0	1.4969225	0.7336147

0.9162473	0.1527675	0.511468	0.729879	0.090447	1.6487875	0.2875905	1.1823745	1.0186342
1.7672593	3.2849484	2.0351312	2.6777337	1.5953127	3.1976715	0.0689688	4.0851413	3.7404599
0.8835426	0.8884608	1.1116991	0.666393	0.0862403	2.3750951	0	1.6239605	0.7414023
3.0052563	2.5507899	1.8786076	3.2280875	0.384713	3.0697709	0.0792928	4.5419918	3.6529855
1.1991226	0.0628125	0.9628821	0.4416959	0.2497487	3.3471128	0.1471764	1.5643782	1.6139088
3.1925572	2.0448837	1.7463128	0.8900964	0.4291071	1.9480401	0.1354035	1.82725	1.9055049
1.4059379	0.5045695	1.7476022	0.9913178	0	1.06371	0	1.5798077	0.1878944
1.1859934	1.2193389	0.2309408	0.4848084	0.520045	1.425137	0	0.7736588	1.1955996
2.1568173	0.5016175	1.2938413	0.5867888	0.1510795	0.2801249	0	0.6795139	1.1396659
0.5952172	0	0.1729995	0.1359288	0	0.3255015	0	0.5122775	0.9293365
1.40523	0.0418037	0.5675454	0.4590119	0	1.3973649	0	1.827128	1.3120262
0.4693651	0.3355976	1.405121	0.4561753	0	2.1473067	0	1.0494217	0.5717253
1.2931939	1.0733404	0.8237494	0.7771146	0	1.2856981	0.1166309	1.3413027	1.6454711
1.1912468	0.5588554	0.5745375	0.5816887	0	0.8183598	0	0.9999279	1.0278618
0.8660771	1.486457	0.6528765	0.7372541	0.5058909	1.6666203	0	1.9056975	1.6235392
0.5939752	0.4657133	0.6003649	0.3579461	0	0.5896676	0.1355349	0.6479131	0.6792436
0.232538	1.0263752	0.4982509	0.5688104	0.3423272	1.4506966	0.4913914	1.6510861	1.2723816
2.5501619	1.3543393	2.4817123	0.8625109	0	2.292016	0.7407117	2.1197546	1.6885832
0.6247092	0.2310637	0.5865006	0.6052098	0	1.3848787	0	1.4902107	1.6247092
1.2407417	0.4670708	0.4113178	0.6462548	0.158337	2.1303379	0	1.9663566	0.5457707
2.986593	1.0880059	0.6132487	0.4675925	0.3819478	0.4774701	0	1.345737	2.5621569
1.5093918	0.4670708	1.1381591	0.5199444	0.1091599	2.9472173	0.2370742	1.4397827	1.0088454
1.0255958	0.3207735	0.6451483	0.7421787	0	1.7268748	0	2.0093113	1.8560694
0.9949417	0.6679375	1.5140465	0.514097	0.1393385	2.053007	0	2.4383993	1.0740258
1.5955991	1.3569889	1.3653566	0.5024324	0.2481705	1.065848	0	1.220268	2.1205178
1.2876496	2.4924685	0.729705	0.8832298	0.1350094	1.0340044	0.1525079	1.3508367	0.8690028
0.5441886	0.0690147	0.368489	0.2961931	0	0.8131142	0.696261	1.0142839	0.8875253
0.4636225	0.2960756	0.3603081	0.1573025	0.1331689	1.5253673	0	1.2454957	0.5650122
0.4087119	1.4498519	0.6593766	0.8738919	0	2.4710313	0	2.0348496	1.5792767
1.9562054	0.0651587	0.9664305	0.2522945	0.1978654	0.6516832	1.0019463	0.4011936	0.3690476
1.0583858	1.1563966	0.7964316	0.5447821	0.2439128	1.7334411	0.3940751	1.2049545	1.0030265
0.7036323	1.0988238	1.1911836	0.3797318	0	1.9663936	0	1.576377	0.5100505
0.7076131	0.3026408	0.7116711	0.1531568	0.255682	0.4610046	0.1023222	0.8840117	0.4692608
1.410938	0.4334936	0.98448	0.7516351	0.2998308	1.5576307	0	1.5510976	1.2470768
0.6698432	0.3607576	0.2152437	0.1770886	0	0	0	0.865523	0.3774014
1.1842168	0.117695	0.499476	0.2046416	0	0.7054029	0	0.1609202	1.2599052
0.4187836	0.1825652	0.5443865	0.6572741	0	0.7378598	0	0.8070251	1.8302148
1.7923556	0.1067503	0.7023916	0.6060631	0.097476	1.9445961	0	1.1984313	1.3745666
1.531369	0.3053285	0.7208038	0.6918914	0	2.7272888	0.1659441	1.0370307	0.8024
0.3329657	0.1961034	1.4621047	0.4269624	0.3392514	1.6130129	1.4893888	2.2053612	0.6091876
0.6138145	0.117695	1.1705661	0.3489122	0.239398	2.5186862	0	2.3866178	0.7013273
2.9340069	0.2137516	3.0666403	0.3985693	0.1544536	2.3391944	0	0.848638	1.1942762
0.8015726	0.5325663	0.5494227	0.9208268	0.1306673	1.7628165	0.897008	1.7558703	0.8088381

0.5912007	0.1126334	0.5792767	0.24123	0	1.8117176	0.3717261	1.1816751	0.4692608
0.4771592	0.3940751	0.4577519	0.2305719	0	0	0	0.9048887	1.3710012
1.2517494	0.1272371	0.1296127	0.267356	0.1602748	0	0.1809117	0.4691566	0.860208
0.9817796	0.30381	0.418028	0.368489	0	0.9754835	0	1.1331689	1.8338211
1.9124966	0.8848714	0.6122109	0.693052	0.1385524	0.6664839	0.5442875	0.8230971	1.7856761
0.7640913	0.1838358	1.6531518	0.8652062	0.2051422	1.62129	0	1.5841447	0.4893374
0.8198317	0.3366262	0.5274708	0.269512	0.0783366	1.6129658	0	1.0668814	1.2525973
0.3178829	0.578987	0.4881036	0.166844	0	0.5739561	0	0.8662354	0.8058702
0.3407332	0.3771793	0.269512	0.4047942	0.1839628	0.3379965	0	0.3745109	1.3117356
1.1232689	0.2368293	0.5840004	0.5056877	0.2636354	1.1164313	0	1.3391944	0.4828483
1.9975092	1.1543888	0.7081431	0.5636463	0.1547128	1.0696334	0.254594	0.6371001	1.7466137
1.7586443	0.1565261	0.6032168	0.1616942	0.1656869	0.773068	0.3523066	1.5145515	1.360589
0.4224483	0.1843438	0.8252158	0.4605853	0.605115	3.0737346	0	0.8133605	1.0234683
0.3109217	2.9391137	0.6077682	0.5772475	0.3168409	1.857145	0	1.8785684	1.8301742
0.5624745	0.374177	0.5295711	0.178619	0.0852887	1.1254527	2.1564937	0.7905218	0.5081244
2.0601512	2.7969922	0.5251668	0.4432884	0.5955991	2.2642662	0	2.2082363	0.947554
0.9413317	1.4229326	0.808591	0.5835191	0.1296127	1.450591	0.0750534	0.6910874	1.445038
1.6369146	3.2891262	1.8418524	0.683921	0.8966206	2.3738152	0	2.1770886	1.4930324
0.5059925	0.1277653	0.1987456	0.1733833	0	1.0292762	0.0852887	0.5533605	1.2518705
0.6254575	0.0614306	0.5044679	0.4750849	0	1.0537369	0	0.5390347	0.6315234
2.0185986	0.9930584	0.6739182	0.4553338	0.3029916	0.6825733	0.4373343	1.2379308	1.5124292
1.5893321	0.0457225	0.9483766	0.2359721	0	0.4836739	0	1.2274947	0.4922121
1.6451483	0.0304067	0.7857598	0.5418122	0.1837088	1.8625506	0.6935873	1.1269729	1.3105146
2.046526	0.9918982	1.0199154	0.8514392	0.3585088	1.9024981	0.2794119	1.4553864	0.9500204
2.0872252	0.7232527	0.4655044	0.4896457	0.1867541	2.4044944	1.8760945	0.8101552	0.7527486
1.4472089	0.9515878	0.6757256	0.6532435	0.2873541	2.0736489	0	1.9570602	1.5684213
1.5259686	0.1705661	0.9756302	0.8727894	0.2140004	0.9498711	0.2409859	1.1508196	1.6138145

AC026355.2	AC034102.8	LINC00663	AC019205.1	AC073534.2	DLEU2	LANCL1-A	AC127024.5	AL137003.1
1.1228715	1.4572791	0.9154827	1.6434868	2.732356	2.136782	1.1955366	2.9356295	2.5615217
2.0037821	1.4607426	0.4072984	0.8572644	1.0794293	0.6175806	0.2773897	1.9084287	1.7153663
1.1073531	0.1728715	0.3287216	0.5689077	0	0.7777038	0.3319345	0.4855299	1.2677755
2.1504297	0.2864081	1.6588741	1.6532435	1.9218552	1.8374589	0.4672795	1.8197091	2.9910093
2.2057678	0.7745024	1.6025994	1.2916036	1.768629	1.0957894	0.498353	1.4526481	3.2364314
1.7701948	1.4617381	1.6173455	0.9652487	1.7975941	1.2617715	0.8644932	1.743515	2.9674638
0.5665716	0	0.3090597	0.3754009	2.8430392	0.9483018	0.2257685	2.0906163	1.6725611
0.9387751	0	0.3056787	0.7527486	0.1906781	0.3739544	0.133432	0.7646859	2.3604486
1.8039709	1.0053281	1.2440955	0.9156356	0.8899407	1.0960594	0.3701643	1.2672961	2.3133039
2.3189242	0.8714494	1.1603394	1.0356239	1.2660369	1.2883586	2.5697584	2.1621778	3.5489051
2.0355535	0.4508022	0.7536901	0.7589001	0.9500204	0.8695556	0.5386375	1.5465611	2.5554726
0.4722798	0	0.6571827	0.7806461	0.3766238	0.8248086	0.2685542	0.3173041	1.9509909
3.8346404	2.7333977	1.4696255	0.4297499	1.3563692	0.6985741	1.9190349	1.4861995	1.3214086
0.8914192	0.3592963	1.3550727	0.561595	1.2124448	0.5827487	1.623024	1.4738908	1.7307921
0.7426961	0.8612408	0.5717253	1.5778759	0.8987114	1.2339494	0.9024981	2.1903936	2.8063447
0.9280481	0.6298462	0.2917214	1.1263121	0.300768	1.492007	1.6892992	1.878176	3.1591932
1.4671751	1.0099204	1.7179122	0.895923	0.5153088	0.8334973	0.2184712	1.2004416	2.1635309
2.2306334	0	0.8911081	0.5268701	0.5599323	0.6616584	3.1468996	0.4762261	2.5894279
0.8963881	0.376846	0.8004961	1.0955869	0.8900186	0.6022668	1.1742146	1.9607712	2.016318
0.8284691	0.8096614	0.3405053	0.7103491	0.4837771	0.7952682	0.8196682	1.8594528	3.3247962
1.6208679	0.4785059	0.7901047	0.486148	1.535406	0.7514637	0.2140004	1.1610492	1.6042137
0.2757223	1.3406762	0.3779566	0.748633	0.8570255	1.0101353	0.3748447	2.2468945	1.9808296
3.4568456	0.2213203	1.2784606	1.4517518	0.7065527	0.5133897	1.2015084	2.1044709	2.6436253
1.2444	1.0347792	1.1120328	0.9910274	1.0774481	0.9702647	2.4438188	1.6769444	2.0647104
1.6431173	0.6528765	0.625925	0.7391568	1.3981862	0.8832298	1.2950765	1.4052844	1.8337807
0.6084308	0.2775087	1.1656869	0.3942946	0.6218057	0.5268701	1.4293749	1.0778582	1.1329716
0.8970855	0.9232633	1.3035178	1.4561227	1.3325074	1.7957254	3.1459546	1.9325904	3.949124
1.7959331	0.7772829	1.6196008	0.9405051	1.3964335	1.1133004	0.3675947	2.4162993	3.4903519
1.8140171	0.4925198	1.6695257	1.2194009	1.1967329	1.2955467	0.4992719	1.7417905	1.6832473
6.2884084	1.878843	2.0706984	1.2295879	4.9881029	3.9277732	1.8834449	3.2378314	3.7276809
3.4390247	0.1286893	0.754973	0.9733541	0.8714494	0.8028963	0.2172307	1.4641977	1.8681731
1.13888	0.9795864	0.4926223	0.3702759	0.6935873	0.4895429	0.3586213	1.1844707	2.1318199
0.7472585	0.4105582	0.5271705	0.9399788	0.2739336	0.4500631	0.4162993	1.4458856	1.7858853
1.3148705	1.6473145	1.5476966	1.9500951	1.8076022	1.3569889	0.8932069	2.6615672	2.7154761
0.2979544	0.0969365	0.6762674	0.833983	0.1220763	0.7651952	0.2893623	1.4963601	3.0473988
0.6304055	0	0.9438838	0.902035	0.4597464	1.5224073	1.45249	1.0588014	1.1170301
0	1.0889557	0.9868842	1.9292608	0.894682	1.3851549	0.4778845	2.2567088	2.3702759
1.6353829	0	0.251204	0.3367404	0	0.4372277	0.1142337	0.6733755	1.166844
3.801376	0.295723	1.3646285	0.6105111	0.6580972	1.0017302	4.0346735	1.4158668	3.7213945
2.5622302	1.3848787	1.4418022	0.8917302	0.959696	1.1547776	2.5056369	2.3661683	2.4258352
1.2489598	0.8757014	0.651775	1.1772162	1.9199121	0.575506	0.2775087	1.6216182	2.6216182

2.8660178	1.2234843	0.905582	1.0425743	1.14496	0.9819987	0.4725917	0.9993506	2.045443
1.3587338	0.6248963	1.1642714	0.9711481	1.2493847	1.0499792	1.0984196	1.7713785	2.5054591
4.2238318	1.198054	1.5764254	1.291132	1.8414901	1.8594925	0.6694804	2.2851654	3.0529549
1.8079731	0.4359486	0.8126214	0.9446336	0.6208679	0.3840498	0.5162169	1.1740868	2.3816433
0.9859375	0.673285	1.8101963	0.8579014	1.3281471	0.8800978	0.8293625	2.1960089	2.9746945
0.9974731	0.3123167	1.8226486	0.8437427	1.5070588	0.4560702	0.4339209	1.1802115	2.6126592
1.4661833	0.6177686	0.7539468	0.9224262	1.8491586	1.3865903	0.673466	1.3710012	3.0090246
1.3562002	0.6234923	1.5812548	0.8933622	1.347779	1.3822799	2.6726064	0.9935657	2.9642323
2.9064866	2.3754009	0.8800194	0.2195867	1.3914378	0.4592218	3.3260627	1.2709475	1.1229377
0.1439158	0.7105254	1.6794688	2.0295589	1.6331522	1.4517518	0.636265	2.1154994	3.5638049
0.7604339	1.139142	0.7177367	1.2105759	0.6879565	1.4696776	0.4688439	2.1786509	2.5736896
0.5518851	0.646347	0.7198406	1.4372277	0.9240999	1.2078304	0.61664	1.9307752	3.2296494
3.6871727	0.9264549	1.2877678	1.3545086	3.3969541	1.3700527	1.5447327	2.8658792	3.1904884
2.9008443	2.0388927	1.9449709	1.1806571	3.3192181	2.9049786	2.6728402	3.2108564	3.2019527
1.8326875	1.1430015	1.3460775	0.7819891	1.3420427	0.5733745	1.0373822	1.5798077	2.8784507
0.7598376	0	0.8275752	0.9078136	0.6173925	0.4964112	0.4501687	1.9497217	2.0024145
0.7055799	0.3276874	0.4543865	0.6586457	0.9795132	0.7651952	0.7366482	1.0486547	2.4904931
1.0085586	1.2513858	1.1703097	1.3914378	2.4802134	1.0726546	0.6385836	2.2801249	3.7834671
1.5507529	0.7555713	1.156979	0.8506394	1.0351312	1.7670615	0.7480318	1.5941185	2.8628481
1.381228	1.0945063	1.0612923	0.5953127	3.0734261	1.6711122	0.9574689	2.080146	3.1766738
0.4839834	0.5689077	0.3447149	0.3559184	0	0.8907191	1.0375228	1.5973166	2.3115322
1.6965726	1.3277448	1.963733	1.951364	1.4470502	1.0211243	1.3417581	2.7086508	2.7873698
2.5512453	0.9141052	1.3858175	1.3110962	1.7799741	1.2940766	0.1742786	2.9907371	3.2842032
5.1754501	2.2974458	1.8003581	1.2031385	2.423623	2.817596	1.9293239	3.3529986	3.9251291
2.0471195	1.6125884	1.0918691	0.9819257	1.1418251	1.1489341	0.1863738	1.8693187	2.7583671
1.8648101	0.3688242	0.3704991	0.9492735	0	0.736475	0.5088343	1.5013627	2.2223718
1.027791	0.1890338	1.2951352	0.9269103	1.4743062	0.5628652	0.8903299	0.6660294	2.0989248
0.6411761	1.2371966	0.6242413	0.9879029	0.6922486	0.8203219	0.3257318	1.5508022	2.2712165
0.8190959	0.4826418	0.5589533	0.7855087	0.2242873	0.5319678	0.2536263	1.3368546	2.1155327
0.4684269	0.092884	0.4316764	0.32112	1.0687396	0.5930191	0.3666999	1.41738	1.4522791
1.3443172	0.8715283	1.1851688	1.4533859	1.9628081	1.609755	0.444985	2.0150336	2.4409256
0.9985566	0.9976898	0.232538	0.3712801	1.4428108	0.9031926	0.1146335	0.6060631	0.8488783
0.5916794	0.6914448	0.3884651	0.7205411	1.3573831	0.6078629	0.9203695	1.1942762	1.6774406
1.9998557	0.7324863	2.0168528	1.4124022	2.0534589	0.7488905	0.3104565	1.1458079	1.4841381
0.5526722	0.4899539	0.8797058	0.9617715	1.6588741	0.8292001	0.7290089	1.012068	2.3293246
0.3797318	0.5956946	0.5587574	0.8465535	0.3542265	0.8876033	0.2519311	1.9941814	1.757706
1.7999991	1.2439128	1.8098671	1.0547096	1.2573728	1.2068307	1.3725064	2.3259332	2.5102277
0.3660284	0	0.4141355	0.3141745	0.4154342	0.5518851	0.4119685	1.0710075	2.2349304
1.6559471	1.9934208	2.1235669	2.250143	2.5589533	1.1464597	1.3306158	2.6337567	3.3528574
0.8234233	0.3272275	0.9044263	0.7072597	0.9782689	0.8793137	0.2295879	1.563451	2.7007948
0.3677065	0	0.486457	0.2851063	0.6198355	0.3898972	0.8782937	0.5286713	1.2609891
3.1706622	1.2023237	0.8695556	1.1797021	1.924898	1.06067	1.361263	2.666143	2.4853495
0.8070251	1.4150555	0.7602635	0.5764738	1.8083027	0.6479131	0.1216786	0.9652487	1.8597708

0.8426973	1.4668098	0.7489764	0.9079674	1.0134265	1.2306334	0.3019388	2.7468287	2.9271569
0.3995539	0.6002698	1.5929712	1.6390932	1.1023222	1.013498	0.5723076	2.5520819	2.3104565
1.429857	1.2764967	1.0542928	1.5007511	1.8520788	2.147209	0.9735745	2.7645585	3.0625535
1.192131	0.1426095	1.2722621	1.1919416	0.7310963	1.2857573	0.3802862	1.2719634	1.5171749
2.8055814	1.0486547	1.2535658	1.0963968	1.2372578	1.2599052	0.8028963	1.3602519	3.8116868
1.3234277	2.0839962	0.3718376	0.8680546	1.7290524	0.7856761	0.0824301	1.8260298	2.2582777
1.7439458	0.2672361	1.1405824	0.6810444	0	0.701416	0.7732368	1.9152914	2.1298765
0.1096949	1.1040682	1.071694	0.8539158	2.7483754	1.0588014	0.3851549	2.4101783	1.903154
1.1926992	0.8316341	0.834873	1.042084	0.7314439	1.374956	0.779806	1.7287477	2.8270873
1.0278618	0.7656196	0.6728326	0.4121854	2.0085227	0.6349649	0.4921096	1.9758503	1.4518573
1.8958842	0.4506966	1.1021878	0.7034551	1.51783	1.6481893	1.9342523	1.8062828	3.1768015
1.8295655	0.7464848	1.0870554	1.3961595	1.4445081	0.8324445	0.4493237	1.6920701	2.4164344
0.5965536	0.6016965	1.2341947	0.546166	0.609755	0.707083	1.3745109	2.0885487	1.8892009
0.7017708	0.3616561	0.3149865	0.3677065	0.3118518	0.7202785	0.6078629	0.582556	1.2378696
1.0100637	0.4530171	0.5392333	0.9085055	0.5545397	0.9218933	0.1467855	1.419431	2.4905701
2.3938555	0.7968469	0.7999991	0.8635421	1.1692195	1.2811938	0.0994972	1.0214087	2.261952
1.6129186	0.8435819	1.639973	1.0548485	1.287945	0.7316177	0.5794698	2.2997136	3.1218609
1.6152279	1.0621908	1.1072861	1.2756627	1.6833371	0.9465812	0.4637271	2.1063148	2.9059285
1.1050746	0.8778228	0.6712028	0.9101954	0.4379734	1.1795747	0.7211539	1.3001237	1.6943454
1.5025343	0.199374	0.6442255	0.8002476	0.8099906	0.5111644	0.2811938	1.0614997	1.8483175
1.7139157	0.2601461	0.6641187	0.8187688	0.5867888	0.9895024	0.6275135	1.292016	2.4649035
1.5953604	1.9348939	0.6300326	0.8131142	0.462157	0.7690947	2.6569539	1.6687999	2.2792038
0.3640121	0.4308205	0.7485471	0.6820339	1.4690524	0.5053828	0.1388145	1.4009204	1.5806279
1.937156	1.8374185	0.9516624	1.2392758	1.0633649	0.609755	1.2448871	1.699063	2.3820585
1.1450905	1.304219	1.3810064	0.9316074	0.8715283	1.3258469	1.924822	1.2439128	2.763709
4.3677345	2.1722955	2.0473639	0.3997727	2.2753051	0.8190141	0.8257856	1.5363016	2.5805797
2.7661499	1.0521376	0.8686078	0.9388503	1.955722	1.9172026	0.2723816	2.1696044	2.7015491
2.325041	0.40523	0.948227	0.8527979	1.3279747	0.945383	1.025525	2.0603241	2.0328414
1.0513025	1.0455129	1.0675699	1.1547776	1.7668495	1.4618428	1.3080696	2.034427	3.4813894
0.6050202	0.4341345	0.7368213	0.9892844	1.1690912	1.1750455	0.0856966	1.0213376	2.3073994
0.8172958	0.9443337	0.818687	1.2184712	1.402613	0.8473556	1.0142124	1.5541468	2.3868937
1.6856711	0.2917214	0.5364508	0.5426048	0.3613192	0.8487982	0.0909889	0.7690947	1.4826418
1.5471043	0.5521803	1.4357886	0.2430597	1.1274352	0.6209617	0.8028963	0.9833129	2.4094181
2.0251351	0.3297551	0.8617966	1.070458	0.4072984	0.3831651	0.7082314	1.8301742	2.7610299
0.2691529	0.5826523	1.2896574	2.1180607	2.0566876	1.0677764	0.5194413	3.3017486	3.816999
0	0.8868232	1.1381591	0.6081469	1.6586457	2.1023894	0.9289577	2.2793227	3.9857099
2.8881685	1.7558703	1.1514043	0.9113464	0.8522386	1.3899522	0.757194	1.6592396	3.2190291
1.1081564	0.4333868	0.6840108	0.6034068	0.2899525	0.7954345	0.7758512	2.3845196	1.9158268
1.423363	0.2678354	0.5934016	0.69073	0.4281424	0.7567671	1.4938522	1.5476966	1.8557904
1.3329084	1.7118473	1.3771237	1.1038669	2.8191777	1.3731192	0.7589001	2.3143195	2.7044515
3.0379796	1.5167212	1.283507	0.8214653	2.026446	0.8078495	1.0572076	2.0080924	2.9230731
0.5045695	0.1396005	0.5840966	0.4852207	0.1750455	0.8190141	0.2707083	0.8885387	1.9145263
0.8274939	0.3043942	0.7834986	0.8904856	0	0.8484777	0.3861488	1.4448261	2.4058835

0.0973411	0.5094425	1.918806	1.7725191	0.6210555	1.9705224	0.895923	2.1573348	2.413919
0.4239546	0.1866273	0.3002995	0.4619476	0	0.3800645	0.239398	1.2909551	1.6046407
1.70982	1.6711122	0.6822137	0.7386382	0.9747496	1.1860568	0.8255414	1.7604765	2.4668359
4.9093363	1.5587574	0.8667102	1.5323668	0.9341768	0.9756302	0.7518921	2.4038131	2.4105039
0.1224739	0.5160151	0.8987114	0.6667566	0.1847246	0.8938281	2.2275255	1.3011194	2.1452862
2.5776101	0.2488991	0.897163	1.0022344	0.3095255	0.9380976	0.523562	1.571968	2.2434863
3.3309742	1.4143521	1.8494789	1.2085797	2.1325768	2.9373256	0.7457967	3.5195545	2.8529577
1.9564656	0.9565771	0.6647559	0.6243349	0.2273714	0.4762261	0.3919876	1.3497616	1.4555968
2.239123	0.7606893	0.7636665	1.2476845	0.7324863	1.75446	0.9690123	2.0953168	2.5604704
1.1307332	0.4146768	1.4318903	1.1725515	0.9905193	1.1906781	0.9277448	2.1282604	3.4525559
0.213876	0	0.4520682	1.1857397	0	0.7248249	0.153935	2.280511	3.8609529
2.639209	0.4904674	0.9852089	1.2094534	2.0306539	1.2096405	0.5939752	1.5750218	2.7470651
1.4118601	0.4685312	0.6288202	0.5302705	0.1658155	0.803723	0.3854863	1.4373876	2.565719
1.4138107	1.1696685	0.215368	0.7908554	0.7930219	0.7201033	0.6906406	1.384713	1.2025744
2.0312187	0.2013829	0.5209502	0.8682917	2.1456775	1.2141248	0.3344539	1.4468385	1.8362473
0.5335633	0.9633261	0.5555217	0.3732863	0.4272843	0.9699701	0.055751	1.7111865	2.5772234
0.6686638	0.8937505	1.071694	1.1794474	1.2947237	1.6697978	0.6611111	1.6310577	2.2765562
0.2744108	0.3266524	1.3880793	1.9127265	0	0.9560567	4.0565142	1.5176788	3.5906499
0.7796379	0	0.848638	0.4027222	0.3516283	0.5286713	1.0067648	0.5413166	1.4100697
1.0613615	0	0.6680283	0.5132886	0.5641342	0.4185678	1.1031283	0.839234	1.640991
2.2561955	1.2539893	0.6626613	1.5407217	2.1759716	1.6944791	0.580049	2.2946649	1.276318
1.3769571	1.9326659	0.5187365	0.4681141	2.1229046	1.322332	0.2058929	1.7648557	1.6550312
1.9211697	0.6749126	0.5952172	0.8415706	0.8137709	0.9392266	0.230449	1.8906802	2.2142492
2.213565	0	0.6671201	0.4571215	0	0.4025039	0.0941009	1.0945063	1.023894
0.147437	0.3344539	0.6082415	0.8214653	1.4971269	1.3783451	0.5024324	1.6087619	2.5425057
0.7564256	1.2102018	0.1806571	0.2007554	1.1464597	0.871134	0.0492125	1.7013273	0.374177
1.2653769	1.2251515	0.5610084	0.3516283	2.1869125	1.3430667	1.1608557	1.6626157	2.2430292
0.9445586	0.169412	0.4307135	0.6677559	0.9632522	0.7996676	0	0.9363647	1.0655723
1.5985081	1.0828388	1.4050666	1.0830431	2.3153633	1.3092344	1.4266405	1.8387905	3.4169748
1.5439907	1.1456122	1.4349351	1.8598106	1.9349693	1.590626	1.2678953	2.6042137	4.2105525
5.0359406	2.3682096	1.6807969	2.1003049	4.3867075	3.9436588	2.9177182	4.2710969	3.4625432
0.3514022	0.2230518	0.7092024	1.0433445	0	1.4676447	1.6036916	1.460428	1.2063306
1.7195339	0.245374	0.5619859	1.0095622	0.771125	0.6440408	0.6710216	0.8294437	1.797096
0.2553194	0.1601457	0.6205864	2.1840898	0.3765127	2.2442173	0.6278869	1.8409264	1.1432628
1.1788102	0.8766446	1.0082717	1.1957885	0.7723924	1.0222613	2.3117937	1.0157116	2.428866
0.504061	0.4201859	0.4221253	0.8840899	0.2807188	0.7256103	1.0220482	0.9150237	1.7317915
1.6436253	0	0.9658397	0.7600932	3.0285868	0.6621143	1.3197335	1.9515878	1.9699333
0.3842709	0.6933197	0.9331949	2.1053764	1.1725515	0.7503492	1.0776532	1.7341785	1.8050448
0.9668734	0.2208252	0.5385382	0.5939752	0.1441769	0.429964	0.418028	0.9656919	0.7869309
1.761966	0.3047447	0.8024828	0.5033487	1.5809655	0.9766569	0.6288202	0.9285788	2.4865599
1.7871399	1.0711448	0.8572644	1.5844334	0.6410836	1.1005068	0.6557182	1.5087329	2.407244
2.1305356	0.8146735	0.9628821	1.0824301	1.5499156	0.956131	1.7939791	2.0075543	2.979751
2.273307	0.4724878	1.1166974	1.4640409	0.3174199	0.9932758	1.0488639	2.0867158	3.1609686

0.8554316	0	0.6592852	0.6874191	0.43957	0.6992851	0.217603	1.0201999	1.3509498
1.6501212	0.3054453	1.1619522	0.6487415	0.924936	1.2282954	0.6301259	1.6884937	2.3982956
1.1423481	1.6764931	0.9863745	0.6462548	0.9486756	0.8080143	2.2279875	1.7229468	2.1058457
2.7525131	2.1063818	0.4327457	0.3674829	1.2180372	0.9236436	0.6375639	1.9008378	2.1860251
0.2721427	0.4622618	0.9471051	0.7898544	0.2140004	0.7352622	1.3421565	0.7307486	2.1870076
0.6601072	0	1.1745982	0.9837506	1.281075	1.0377336	0.6526012	1.0229004	2.7986312
0.2873541	1.0489336	1.4960021	1.2091414	1.6026469	1.3153923	0.7641763	2.2985997	2.4008658
1.953079	0.725174	1.0662614	0.8451889	0.6351507	1.2316782	0.2504766	2.5891642	2.9340446
1.0308304	1.5894759	0.4050121	0.7146632	1.0938982	0.8539956	0.6630258	1.5216538	2.242633
1.1845342	0	0.628447	1.2158028	0.4080596	1.5273206	0.7418337	2.1712709	2.710305
4.7970234	1.0000721	1.1918153	0.4709273	1.5388858	0.8893178	0.3678183	1.1521185	1.7855924
2.3025238	2.488335	0.8329305	1.2311866	2.2743809	1.2913678	0.8357624	1.7364317	3.0753957
1.0619144	0.4614238	0.4634132	1.2971914	1.5435454	0.8659188	0.2855798	3.0539107	2.359746
3.0222258	0	1.1662013	1.3846577	1.4505382	1.0873271	0.6728326	1.8560694	3.3432799
2.8075816	0.9193019	0.9845529	0.851839	0.9585829	0.6028369	0.4276062	1.8660376	2.376346
1.7756827	0.1545832	0.8645725	0.9065058	0.780898	0.5832302	0.5776343	2.2240712	2.4946716
0.5605193	0.2532632	0.6189904	0.6659385	0.3147545	1.0490034	1.6404358	1.3832757	2.3188953
2.7413375	1.2180992	0.8117998	1.0008654	0.7432995	1.0984869	0.6577315	2.290041	2.6809769
0.581303	1.0588706	1.579856	1.3312466	0.9391513	1.0777899	1.8140171	2.139011	2.8838358
0.4325319	1.1840263	0.3607576	1.0198442	0.780898	0.9072753	0.8316341	1.9311157	2.5489051
0.4482667	0.5280712	0.3632272	0.6271399	0.7431272	0.4669664	0.1741507	1.6461166	1.5384885
0.7567671	0.9481523	1.0270833	0.7411434	0.9593992	0.6018866	0.6966171	0.4745658	1.1639495
1.6666657	0.7720546	1.0698396	0.96377	0.7953514	2.4306064	0.3351402	1.5296211	2.8787253
1.4268551	0.779722	0.9199121	1.8794705	2.1316224	0.7086728	0.4402081	1.9621788	0.894682
1.2467121	0	0.5508022	0.6846392	0	0.651775	0.1646576	1.8547138	2.8934981
1.9921158	0.6375639	0.9948693	1.4066999	0.4361618	0.4897484	0.147437	1.5560615	2.5887806
1.5262692	0.2717842	1.1848515	0.7744181	1.0380849	0.5255678	1.0590091	1.2036397	2.0017302
0.7773671	1.7010611	0.3674829	0.9902288	1.5753123	1.856189	0.380397	2.4687658	2.7611789
0.4881036	0.217603	0.6942116	0.7009723	0.8694766	0.716069	0.8506394	0.595981	2.0038181
5.1001984	3.2416521	1.0528796	1.7794699	4.7697541	4.0142839	1.8955224	3.5547648	4.0082658
1.6917575	1.1106305	1.4545444	1.340961	2.2016966	1.4684269	0.8954577	1.4617381	3.8591049
1.3668118	0.7121996	1.4762261	1.0248161	0.8567069	0.6633902	0.4549128	1.2249664	2.2551381
1.8125393	1.0447438	0.212009	0.7923556	1.9563541	1.7795959	0.0582472	1.3968719	1.4979444
2.4814023	0.9759236	0.6479131	0.3046279	1.5071603	0.7512923	0.3569326	0.5912007	1.7246503
0.4735272	1.8125804	1.5846258	0.9815604	1.4831063	1.678117	0.9690123	3.5678496	3.1499909
1.1560082	0.7330072	0.7832471	0.7238643	0.2753647	1.9157886	0.3655806	1.7023916	2.1197546
0.3230818	0.685985	1.0020904	0.4430762	1.7312701	0.7834986	0.4830547	1.283981	2.6244753
3.0980152	2.1981798	1.0487942	0.6782522	0.3257318	0.4309275	2.4328793	1.1720394	3.2790701
0.5751187	1.1296787	1.428464	1.1666512	1.6206802	1.7397617	0.962438	2.14496	3.3227935
0.951364	0.772139	1.6697071	1.825175	1.6589198	1.1721034	2.1140338	2.5950023	3.3701503
2.2292187	0	1.0538759	0.3727293	1.2754839	1.0416635	0.2015084	1.0576929	2.2249972
1.0500489	1.6617952	0.4716557	1.015997	1.4057201	2.0743684	0.3941848	2.2792038	1.8806855
1.6260185	1.7227283	1.0559592	0.90589	2.0754642	2.4730595	0.3416442	2.0472592	2.6472454

1.0712135	0.3415304	0.3875831	0.7451943	1.3046863	1.2954292	0.7191396	1.5187365	1.9709641
3.9980902	2.1038557	1.3373115	2.0865913	3.0443125	3.2883832	2.062375	3.8975147	3.7079738
0.9327415	1.1838993	1.2467121	1.6198355	0.2814312	0.6613848	1.4453559	1.3779011	2.588013
5.2143152	3.2206782	1.8834449	1.616687	3.2532178	4.79253	2.6241009	3.7091472	4.2268591
1.0513025	1.0289227	1.1600166	1.5731806	1.1086917	0.8949923	0.7803101	2.3850997	3.1025742
4.3263288	1.5393325	1.1874511	0.6288202	0.932288	0.4713436	1.2108252	1.9055049	2.1026581
1.6914895	0.2548359	1.5135918	1.328779	1.7882263	1.3818371	0.6423781	1.9385493	2.8677581
2.1528	0	0.951364	0.5694911	2.1420539	0.7076131	0.2306949	0.7009723	1.4968202
0.4601659	0.541614	0.7027462	0.9527063	1.7291394	0.8393147	0.2874723	1.3735647	2.5944769
0.198997	0.2380531	0.263395	1.4996291	0.2961931	0.7214164	0.3338817	0.9557592	2.5753849
0.5103543	0.9266067	0.6941224	0.3427823	1.1005068	0.4532278	0.0529723	1.529721	1.0563754
3.0422241	0.8562288	0.5972213	1.0316775	1.0209822	0.5517867	0.1088924	1.6425167	1.8380643
0.6673926	0.3848235	0.7893537	0.9448584	1.1144337	1.0360461	1.1460686	1.437281	2.4151367
0.2000021	0	0.8362473	0.3884651	0.2976023	0.9649531	0.3940751	1.4330663	2.1502022
0.398022	0.6580058	0.809826	1.0868517	0.7942703	1.2100147	2.4819705	1.4938522	2.1308979
0.3729521	0.1625968	0.7130799	0.883699	1.494774	0.9127265	0.8451889	2.1288543	3.5528443
0.3828332	0.8952251	0.7767778	0.8723166	0.2091414	2.1090596	1.4767964	1.729618	2.8728091
1.4549655	0.9817066	1.7863873	0.9620677	1.4066455	1.1166309	2.151729	1.8290783	2.7242355
0.2488991	0.2967804	0.8405237	0.5373458	1.4546497	1.1941501	0.4131608	1.4618952	2.5120246
2.0559938	0.3996633	0.985719	1.343351	3.2806891	1.8187688	0.4053389	3.119572	3.4411382
1.1768334	0.8750723	0.7904384	1.1774714	1.3725621	1.6122109	0.718789	2.071694	2.8218326
2.2993326	1.6107945	0.7029235	1.7107018	0.4977401	1.3933613	0.4937498	2.2782524	2.4580145
1.2325994	0	0.8417316	0.524164	1.2548963	0.8851058	0.7541179	1.0999011	2.0674322
5.1265393	0	0.8560694	1.3797873	1.3757345	0.455439	1.5578757	1.2995377	2.4230672
1.9933121	1.4215869	1.0375931	0.9991341	2.4408193	0.8479168	2.2248429	0.9612529	2.5056877
1.8091675	0.3448285	0.7275284	0.9820718	0.8933622	0.8318772	2.6261354	1.3930867	2.3041605
0.4371212	0.2804813	0.8502394	0.6656656	1.063779	0.5096452	0.0443242	0.5356549	1.4856844
2.622743	1.3771793	0.8383467	0.642563	1.8224446	0.9483766	0.158337	1.0812033	2.3683772
1.2189051	0.9411814	1.0558204	1.2345013	0.6639366	1.6340356	1.7096435	2.3495917	2.9138564
2.0600128	0.2657969	0.6667566	0.3467584	0.3299847	2.2960756	0.1602748	1.9882666	1.1094274
2.0840302	1.09815	0.8075198	0.7890197	1.1942132	0.8231787	1.2171687	1.4469443	2.6259484
2.2516888	0.3722835	1.2110745	1.0161397	1.0853567	1.8134836	0.6765382	2.0834857	2.4791012
0.6660294	0.3411888	0.9879757	0.3701643	0.8407653	1.0094188	1.1124333	1.993457	1.2575538
0.432425	1.0423642	0.8982471	0.6455172	0.6214307	0.9514386	0.6640277	2.8512193	2.2069245
0.8919635	0	0.3799536	0.4693651	0.8407653	0.7863454	0.6804144	0.9028068	0.9634001
0.8951475	0.5329652	0.4673838	1.0635029	1.4346683	0.754802	0.1196882	1.7402368	1.9090434
0	0	1.1815479	0.5568953	0.6790634	0.6956375	0.2703495	0.5808208	2.326336
0.9046575	0.899485	0.9912452	0.6260185	1.4383461	0.5417131	0.0783366	0.9310022	0.7782927
1.3683214	0.6692989	0.9308509	1.0984869	0.9540474	2.0261981	0.4705108	1.6618864	2.868588
2.7146192	0.6296597	0.9537495	1.1091599	0.3006509	0.8869013	0.7556567	1.950879	2.3318772
1.6076262	1.7480747	1.4285176	1.3223897	1.6090457	1.7120234	0.7527486	3.0043576	2.0110662
1.1071522	1.2154301	1.1478928	1.1084241	0.6714745	0.8311476	1.3031086	1.7496201	1.5352069
1.1265765	0	0.7146632	1.0867838	0.5823633	1.3965431	0.2954879	1.9006833	3.3964609

2.6843475	0.7754299	1.1710787	0.80719	0.7468287	1.41765	0.2709475	1.851799	1.8798626
0.5401265	0	0.5471537	0.5946441	0	0.6528765	0.1108977	1.1068843	1.5571895
2.359999	0.4042492	0.3662523	0.3780676	0.269512	0.3886855	0.0662614	1.3361691	1.345964
0.7463988	0.8653646	0.4184598	0.8775872	1.5429514	1.3721163	0.3543393	1.8410472	2.2670563
1.0210533	0.4295356	1.3587901	0.5860201	0.9776096	0.6112668	0.9904467	1.0124256	1.8018622
0.70982	0.4333868	0.6804144	0.7102609	2.0719686	0.830093	0.4501687	1.7750927	1.6862988
0.4645637	0.6205864	0.4973313	1.0696334	0.6647559	0.9620677	0.7580473	1.9914992	2.3128104
0.3623296	0.899717	0.4514353	0.843019	0.9105792	1.1760674	0.587557	2.5150564	1.8645328
5.0348452	0.6427478	0.5456719	1.217727	0.5611061	0.4577519	0.2836848	2.1790333	1.8499592
0.4638317	0.5458696	0.6742799	1.4731635	0.6637545	0.9846258	0.1807844	1.995376	2.4476585
4.3316049	1.1673579	0.6383983	0.5475485	0.4809889	0.9061979	1.6647559	0.7942703	1.9619566
3.1489666	0.4167317	0.966209	0.9947245	0.888227	0.9350071	0	1.2650768	1.1040682
2.3048906	0.3091762	1.2065182	0.7347421	1.146981	0.674551	1.1241296	2.3941025	2.8107104
4.2564748	1.2337654	1.2608687	1.1561377	1.9224262	2.6513158	1.1226727	2.2380531	3.0045014
0.5688104	0.3233124	0.7264825	1.1815479	0.7121996	1.1674864	0.1964811	1.1558787	2.224349
1.6444101	0.9543452	1.1741507	1.4411116	0.674099	1.3025238	0.9144114	2.8846565	3.782765
3.7405175	0.929791	1.1173626	0.9177754	1.2103889	0.6205864	0.2018847	2.064538	2.8531374
1.6780268	1.2228046	1.7155859	2.2028252	1.9352334	1.2171066	2.2063306	2.6506727	3.0518419
1.0243906	0.2884176	0.3469852	0.3173041	0.3573831	1.0260918	0.1467855	0.946656	1.3249834
0.8366513	0	0.7124637	0.70982	0.5697827	1.1812934	0	0.8470348	1.3982409
3.0439569	0.6191782	1.054779	0.8192594	0.7492339	0.9889936	1.7640489	1.1604685	2.8093939
0.9492735	1.0902437	0.2877087	0.907506	0.7799741	0.8556708	0.5769574	0.9161709	2.8780779
1.4300711	0.8052099	0.5031452	0.5950262	1.2774492	0.5746344	0.8589358	1.4655044	2.5474005
0.4005379	0.4732154	1.2726205	0.8693977	1.7930219	1.1050075	1.2794713	1.9934932	2.240528
3.4847053	1.8730257	0.9521099	1.2580968	0.3122005	1.3548471	0.5278711	2.2588505	2.4203207
2.5852029	1.2416572	1.0824301	1.5057893	0.9731337	1.6949693	0.4815056	2.7136518	3.3901862
1.2937825	0.2869994	0.7763567	1.9765103	1.0829069	0.6140973	0.4682184	2.1565261	2.9477036

AL021368.2	AC090948.1	AC121761.2	AJ003147.2	SEPSECS-A	CRIM1-DT	AL360270.1	EML4-AS1	AC023509.2
1.5475979	2.2980131	2.0432745	0.2215678	1.6397878	3.384713	0.6050202	1.3908327	0.0899049
0.7766936	1.9383234	3.5061195	2.2103577	1.5109113	3.1252047	0.2747686	0.2914857	0.042084
0.2578555	1.0855606	0.851839	0.9500951	0.8864331	3.4941724	1.7777038	0.4753962	0
2.7565964	1.5536554	0.5486339	0	0.8904856	5.3185229	0.0635029	0.292075	0
0.8334164	0.6060631	1.3205424	0	0.6946574	4.3630098	1.7576207	0.4469972	0.6113613
1.4050666	2.2572822	1.7346121	0	1.1739589	4.1184843	0.7990044	1.2599052	0.5002922
0.525668	1.9687175	2.9689202	0	1.2525973	3.8190448	1.0546401	0.1367164	0
0.3579461	0.3719491	0.8328495	0	0.6263924	3.999991	0.6300326	1.3090015	0
0.8758587	1.8140582	0.9586571	0	1.4710313	1.4731115	2.6165224	0.5992226	0.1839628
1.7116711	2.0168528	1.0484455	0	0.3170725	3.559039	0.8433407	1.5398784	0.3561438
1.0024505	1.7063758	1.3842156	0.6775308	0.230449	3.8897753	0.8094144	1.6792436	0.2681948
0.1729995	1.0252415	0.580049	0.4525954	0.5809173	3.5310196	1.4765891	1.2871176	0
0.7368213	2.0869875	1.5774893	0.0897693	1.2827363	3.0826685	0.5396303	0.4612142	0.5583656
0.8353582	1.8385081	1.7011498	0	1.0702519	1.8045905	0.3674829	0	0.1315895
1.1495195	2.6227196	1.8404431	0	1.2848102	2.3615719	1.1379625	2.5530655	0.1508196
0.7735744	1.7709981	2.5846499	2.0191326	1.8459918	3.9294028	2.5246404	0.3559184	0
1.5882769	1.1320503	1.0205555	0	1.400374	2.7356955	0.7987556	1.2520523	0.0954519
1.2328449	0.5787938	0.6082415	0	0.8045492	4.0700544	0.4271771	2.643025	0.154972
0.989357	1.7800161	3.1815956	0	1.502076	3.1846453	1.4650864	0.25181	0.1252543
0.268075	1.0745739	1.3709454	0.6989296	1.3331375	3.6184501	1.8219143	0.972178	0.0361868
0.5055861	1.2965455	0.9556104	0	0.9590282	2.0429595	1.8920412	0.4875892	0.0227584
0.6256446	1.0096338	2.899195	0.7381193	1.6521423	3.132511	0.0736832	1.1210154	0
0.8071076	0.6642097	2.343891	0.3823906	0.651775	1.4615809	2.2863489	0.9502444	0.0938306
1.7932717	2.0827707	1.7631566	0.0326651	1.4936473	3.0895998	0.795102	0.6605636	0.213254
0.661932	1.4676968	1.0741629	0.0529723	1.1324452	2.0760117	0.7693486	1.4232017	0.0812033
0.2761989	1.5208999	1.6255978	0	0.751378	2.2588505	0.5211513	0.1484786	0.0606008
1.7676548	2.9725824	2.6673699	0.0553345	1.219153	4.1372166	0.605115	1.0489336	0.2771516
1.9020736	1.7620086	0.4866629	0	1.1412366	3.6459322	0.9758503	1.1160986	0.3984598
0.4903648	0.9235676	1.7165959	0	1.2085797	3.8260908	1.0310422	1.2961343	0
3.0722602	4.2012026	2.3622454	0.0485152	2.1200699	2.0603068	2.3212354	1.9871389	0.4681141
0.3637878	0.8658397	0	0	0.988921	2.6564048	1.7126398	0.1314578	0.0795659
0.337654	0	0.8901743	0	0.7441611	1.5499156	1.1352065	1.0824982	0.1354035
0.46425	1.0112809	2.2147776	0.0607392	0.902035	3.9106271	1.4537019	1.0659858	0
1.1578198	2.2941355	2.192857	0.7166837	1.6388616	2.5291463	1.8580208	1.2273714	0.1800205
0.366588	0.8455904	0.7518065	0	0.3188085	2.0449536	1.5837598	0.1461338	0.0596322
0.6548021	0.6653928	1.7148829	0.7282253	0.8901743	3.4798772	0.5254676	0.8420536	0
1.7330506	0.261591	1.6858056	0	1.6225087	2.1945914	0.3533233	1.3207157	0
0.1070183	1.1708224	0.9979066	1.0280033	0.5588554	4.3852239	2.0514765	0.5213523	0
0.8332544	2.2661569	1.621712	0.0833836	1.4158668	5.745353	0.9159415	1.5736169	0.3520805
1.5664742	2.8095791	2.2982477	0	1.5087836	3.2265856	0.6410836	1.9919707	0.0357647
0.8572644	2.1388472	0.5976027	0.0388576	1.5018722	2.6102512	0.4018489	1.1890971	0.0886844

0.7121115	1.3295829	2.1646254	0.13711	0.6911768	3.6555923	1.8722378	0.7505208	0
0.8154935	1.2832699	1.5846739	0.0501882	1.1406478	4.7154486	1.1347467	1.0279325	0.2864081
0.9026525	2.2963987	1.8982858	0.3674829	0.692695	3.5853592	0.5412174	1.9743825	0.5722106
0.3205424	0.4575418	1.0291348	0.0522768	0.7701948	3.3687683	0.9781224	1.130865	0.1745343
0.8317151	1.4349884	1.5259686	0	0.9199121	3.8048073	1.5765705	1.8715677	0.0843364
0.2590615	0.8893178	0.4218023	0.1717192	1.3241769	3.8504394	0.8426973	0.1680001	0.1347467
0.7105254	1.0700458	1.3586776	0	0.6991962	2.3898421	1.1653653	1.1565908	0.1482183
1.3925922	1.9484513	1.9441838	0.0985543	1.6678467	3.7936878	0.5840966	1.963881	0.2529
0.2696317	1.3231971	1.533663	0.0265876	1.073409	3.1362898	0.5083272	0.1491293	0.1382902
1.2447044	1.0854926	1.653427	0	1.1844707	2.9191875	0.956131	0	0.0367495
0.3713916	1.8255007	1.4272843	0.1300083	0.486251	2.169412	0.5052812	0.9879029	0
0.6318028	1.2450697	2.4766409	0.2445218	1.7121555	1.6970176	1.0715568	0.2829735	0.0802483
1.6277936	2.9783787	2.32386	0	1.5855875	2.7979883	1.2729787	1.2514464	0.3140584
2.3617496	4.0115255	3.050937	0.0144981	1.4187477	2.7058084	1.5097634	2.0517085	0.4907413
0.9300939	2.3530692	1.5559143	0	1.4057201	2.5345348	1.8278597	0.6938549	0.2747686
0.4648773	0.946132	0.7977601	0.2202061	0.9165531	1.2570106	1.4690524	0.7177367	0.117828
0.1574318	1.2319239	1.0529723	0.5525738	0.5672534	3.517704	1.7233401	0.1767057	0.072586
0.9942176	2.1751733	2.2215678	0.1112985	1.5945008	2.2488688	1.0164963	1.1475021	0.1285574
0.8252972	2.0550221	1.293135	0	0.8443053	2.374956	0.9710745	1.5004452	0
1.6447331	1.7785871	1.2432425	0.2775087	1.3090597	2.2812235	0.4500631	1.6951921	0.0918014
0.6084308	2.0691522	1.2377473	0	0.78668	3.0057772	1.0641931	1.3117356	0.1976138
1.3466449	2.0687396	1.7371676	0	1.654298	2.8461322	1.0809305	0.6936765	0.2798873
1.0378039	1.2806	1.7127718	0	1.4946204	3.2885653	0.5693939	0.3799536	0.3432372
2.0882095	4.2060597	2.0004087	0.0759661	1.87883	2.5473264	2.4046761	2.1511553	0.5514586
1.0818168	1.8686078	2.1164313	0.5413166	0.8552722	3.9490773	1.4270698	1.5164691	0
0.5074141	0.5980793	0.6905512	0	0.7482036	3.0564968	0.6209617	0.674099	0
0.4644592	1.3109799	1.2490812	0	1.8027722	3.3322496	0.4577519	0.7786291	0.3916578
0.2859348	0.8911858	1.72927	0.9163238	0.8370552	4.3015072	2.1249236	0.2000021	0.1857397
0.5171245	1.116032	1.2602666	0	0.8435819	1.6329196	0.4363751	0.686254	0
0.1943392	0.8169683	1.8537562	0.024887	0.9544197	3.7621362	0.7263953	0.3077782	0
0.8076022	0.7919391	1.2968979	0	1.1956626	1.245739	1.3879139	1.486457	0.0593553
0.1074201	1.4634132	1.3222166	1.3049782	0.7623063	4.4653934	1.2379308	0.628447	0
0.3381106	0.962438	1.198997	0.1115655	0.4422269	3.9854549	1.3116775	0.2105136	0
1.0542928	0.7898544	0.9480775	0	0.5812066	3.2187347	0.5934972	0.4201859	0.0475384
0.3702759	0.8354391	1.3087104	0	0.4806787	3.7564042	1.0206978	1.2158649	0.0465609
0.5086315	1.0666748	2.2149019	0.1062143	1.1434587	1.9263031	0.6909981	0.9785617	0.0626744
1.1777265	0.9746027	1.1066833	0.1929516	0.4805753	4.6988574	0.9667996	0.8790784	0.2219389
0.1921942	0.623305	1.1572378	0	1.0361868	2.7133879	1.8428582	0.3431235	0.0253123
2.1166974	1.8594925	1.3124909	0	1.5590023	1.9233774	1.8170502	1.4065367	1.0226164
0.484396	1.8251343	1.6258316	0.0930192	0.6979517	2.5422333	0.6388616	1.2816092	0.0724489
0.1891604	0.3199647	0.3188085	0.5462648	0.7355222	3.0138195	0.8436623	0.7517208	0.0405418
1.8863941	2.6539772	2.2829735	0	1.7710827	1.7003953	1.285343	0.2685542	0.2903064
0.7528342	1.2239168	1.5956469	0.0560285	0.4186757	3.6112786	1.104538	0.4721758	0.0436245

0.9900109	1.9888119	0	0	0.5839041	0.8861989	1.1273691	0.4091465	0.2954879
0.9334971	1.3741214	1.785132	0	2.0441843	3.7859062	1.3717819	0.6110779	0.0371713
1.2890081	2.1646576	1.7362152	0	1.3278598	2.8199542	0.8304987	2.1872293	0.2533842
0.2822619	0.7146632	0.7283995	0.1837088	0.8076022	2.5036286	1.7601358	0.6636634	0.0299829
0.6597419	0.646347	1.3226781	0	0.9096579	2.4651909	1.0976782	0.4089292	0.1760674
1.031254	1.0503972	2.1420213	0	1.2047042	2.0749506	0.5835191	0.8701871	0.1137005
0.4128357	1.5723076	0.6525094	0.1458731	0.9963887	2.4077334	1.5870769	1.2340721	0.1142337
0.4477379	1.1259155	1.9611047	0.0356239	0.574925	1.8719619	1.7646434	0.574925	0
0.509037	1.9501324	1.9373067	0.458172	0.398022	3.2092506	0.9309265	0.9973285	0.0739573
0.354565	0.7995847	1.6616584	0.9916806	1.4797997	4.5256617	2.1805935	0.0997665	0
0.5984605	1.8426169	1.1105637	0	1.4172179	2.9615863	1.2582174	0.8824474	0.3333092
0.7658742	1.2933116	1.9723986	0	0.9040409	2.4543865	0.875151	1.5748766	0.258338
0.4189995	1.0027385	1.8550329	0.6982185	0.9237957	2.231371	1.1021878	0.6125884	0.1160986
0.2601461	0.1159654	0.3413027	0.1036655	0.4311415	2.7063095	1.0234683	2.5494967	0
0.4510132	0.8146735	0.8983245	0.0457225	0.4955928	2.2936648	0.7494914	1.5599323	0
0.3155662	0.722466	1.5356051	0	0.7968469	3.1375527	1.5688104	1.5896676	0
1.5905781	1.6328266	1.5427038	0	1.176961	3.0363451	1.0176369	0.810731	0.2152437
1.4551233	0.9703383	0.6884042	0	1.96857	3.6780043	1.5786972	0.5854433	0.4819189
0.6834719	2.8387098	2.6543668	0	1.2975436	4.1081982	1.0222613	0.1924467	0.2941943
0.2803625	1.0324535	1.2349304	0	0.3561438	2.4617119	0.8887725	1.8685682	0
0.5504082	1.817787	1.7179999	0.0367495	0.7860945	3.3971869	0.7211539	1.3589588	0.1110313
0.7473444	1.3992258	1.4169478	0.1079556	1.5354558	0.8368129	0.2971327	0.9630302	0.3627785
0.3419857	1.2513858	1.6219932	0	0.6059684	2.599889	1.2367681	0.9178518	0.0499095
1.6388616	0.4627853	2.1092268	0	2.0598744	3.8121696	0.5695883	0.5245652	0.0612923
0.9814874	1.8443857	1.316725	0.0603241	1.6001746	3.3004752	0.4874863	1.1165644	0.5811102
1.3460775	2.1061473	3.0689115	0.0448837	2.7864709	1.7651104	1.4128357	0.3885753	0.4253519
0.8976277	1.7734478	0.587653	0	1.6418234	3.3206724	1.7752191	0.0854247	0.2253983
0.4349884	0.8175414	1.1959145	0	0.6121166	1.8937505	0.151729	1.6574571	0.031254
1.5795181	1.8503594	2.4985317	0	0.7054029	3.2629292	0.4731115	1.3958854	0.2235461
0.9272138	0.7492339	2.7057568	0	0.6574571	1.2015711	1.3938555	0.1000357	0
1.1723595	2.0906502	1.995195	0	1.4464151	3.3283913	0.5030434	1.5308698	0.247563
0.2661569	0.4803685	2.4762261	0.0821576	0.6531518	3.9559265	0.974823	1.4423862	0
0.2378084	0.6929628	1.9199502	0	1.4518573	4.741111	0.3640121	0.1624679	0
0.2458607	1.2378084	1.7026575	0.3432372	1.4768483	3.548116	2.1369788	0.4093638	0
1.0873949	0.2860531	1.2618918	0.0908534	1.2688535	4.2550475	1.5046204	0.1726155	0.0709388
1.2980131	3.5209753	2.5778034	0.102994	0.8868232	4.0307686	1.9860104	1.5262692	0.5444854
1.1306673	1.4204555	2.0416986	0	0.6768992	1.8135247	0.7228157	0.9191494	0.1326426
0.2702299	0.6941224	1.8418524	0.2428159	1.0770379	4.2880558	0.655535	0.4416959	0
0.4137024	0.7306617	1.3653566	0.2141248	0.5981746	3.0965486	0.4166236	0.6053047	0.0583858
1.5704144	1.8397984	2.194749	0	2.2825288	3.5366995	0.8304176	1.3820032	0.0919367
1.6458861	2.3040437	1.9547919	0	1.0243906	2.7089376	0.8342258	0.4651909	0.4214792
0.4514353	0.7020369	1.8639385	0.0746424	1.0193461	1.7151905	1.6248028	1.0272956	0
0.4670708	0.2715452	0.8678174	0	0.6782522	2.5004707	0.9194545	0.7313571	0

1.1342209	0.9787814	1.8313098	0.1224739	1.3837181	3.0692038	0.4670708	0.5189379	0
0.1935825	0.5724046	1.4318369	1.2681349	0.6628435	2.4766668	1.0345679	1.0663303	0
0.631989	1.0034583	1.7086287	0.0353424	1.3631711	0.7031007	0.9670948	1.0482362	0.2065182
1.0332292	2.1179942	2.6443639	0.0381552	0.8902521	3.4265197	0.8127036	1.271067	0.1423481
1.1663941	1.7525345	1.952259	0	1.3920976	3.7253703	1.9264929	0.7776196	0.1204847
0.4044672	1.5813512	1.5775376	0	0.5668639	2.6393248	1.5134402	1.8475961	0.1060803
1.1208164	1.7437735	1.6349649	0	1.1025238	3.1583855	1.3238888	1.6053995	0.1825652
0.369271	1.2037023	2.0737517	0	0.8410875	3.4763817	1.7650679	0.811882	0.0387171
0.6170163	2.4430231	1.9732072	0	0.6586457	2.4225829	0.7759355	1.8370148	0.0980152
0.7420062	1.7006617	1.772688	0	1.1749177	2.8408258	1.0497702	0.5398288	0.1536757
0.3368546	0.5971259	1.3973101	0	0.5805314	2.2765562	0.8759373	1.0633649	0
1.0524159	1.8067365	2.7001068	0	0.8195047	3.1167141	0.9976898	1.2918982	0
0.4109923	0.8840899	0.7431272	0.0706641	0.7774513	1.4729036	0.9150237	0.9858647	0.0817487
0.4117516	1.518233	1.0568609	0.0357647	1.1765143	2.6350114	1.4826934	0.7125518	0.081885
0.2172307	1.2144357	1.3664201	0	0.9375704	2.0597706	1.411155	1.5429019	0.0430645
0.5155106	0.6763577	0.8935952	0	1.1014484	3.4180415	0.940054	1.1687704	0.1142337
0.5891882	0.3243498	1.2668765	0.1040682	0.9776096	2.5648171	1.2503553	0.72927	0
1.1932671	1.0840643	1.279709	0	1.0710762	3.1256346	1.4979955	1.6814942	0.3881344
0.2106382	0.2529	0.3843814	0.0797024	0.8061178	2.6258082	1.842295	1.0776532	0.0621217
0.328262	1.4115347	0.6126828	0	0.5462648	3.0825323	1.0308304	2.0950467	0
0.1947175	2.100103	1.9976898	0.2229282	1.1577552	3.1056613	0.2590615	0.4078422	0.3567073
0.5160151	1.2614105	3.9401574	0.127105	0.4966157	4.7215805	0.5196425	0.3448285	0
0.3848235	1.9928409	1.563451	0.2091414	0.5279711	1.960697	0.9849174	1.8240346	0
0.1712068	0.4235244	0.7246503	0.028852	0.5247658	5.1947293	1.5071096	0.212009	0
0.4117516	1.910771	1.9413693	0.1844707	1.2044538	4.0466133	1.3366262	1.5525738	0
0.0758749	1.5336132	1.8482373	0	1.0061185	4.2953704	0.5988416	0.5736654	0.0344975
0.6525094	2.5685186	2.1431322	0.0889557	0.9901562	3.3684611	0.5101518	0.697418	0.3458505
1.0809987	0.2865263	0.9729132	0	0.686254	0.4108838	0.5711428	0.2521734	0
1.3315906	2.6149689	1.6051624	0	1.5052812	3.9533677	2.0586628	0.6521423	0.0639171
1.7745446	2.9411814	1.6126828	0.0375931	1.0781999	4.0559071	1.3414165	0.6006504	0.1134338
1.3688521	4.2473883	3.4906921	0	1.7080989	2.5073507	2.587509	2.9362893	0.5665716
0.0640551	0.5283713	0.3043942	1.0657102	0.7063758	3.2667116	2.4935961	0.2276179	0.0480967
0.5387368	1.7174297	1.6248028	0	0.9919707	2.5616927	0.5797594	0.6494775	0.0531113
0.1456122	0.8701871	1.2136894	0.206018	1.1628545	0.949647	0.9908823	0.5654997	0
1.0592168	1.4435536	1.3035178	0	1.6421008	3.4529775	1.0209822	2.7589001	0.1904252
0.2052674	0.2003788	1.9449709	2.8721787	1.3343394	3.9572089	2.35834	0.1195554	0
1.0850167	1.4349351	1.3694944	0	0.6030269	2.5602503	1.0174231	0.6563591	0.0462815
0.9541218	1.5106582	0.9005674	0.090176	1.2785201	3.5164691	1.403595	0.7054914	0.0356239
0.3353689	0.7277898	0.8586176	0.1759397	0.8694766	4.5629628	2.0313599	0.3260771	0
0.4969225	0.6349649	0.5265697	0	0.6310577	2.8099494	0.6293799	0.9720309	0.1313261
0.65764	1.817173	0.888149	0.1573025	0.6885832	2.5006746	1.165108	1.3005338	0.0318186
0.8883049	2.1193894	1.254594	0.162339	1.0677075	3.2061587	0.9974731	1.5930191	0.4529117
1.5057893	3.2541405	0.8613202	0	0.175301	2.9851725	1.3050366	1.8378222	0.1090262

0.2200823	1.2386035	0.7565964	0.4888235	0.5378427	2.6303123	1.1428708	0.9497964	0
0.6363578	1.805086	0.8704239	0	0.7268312	1.5215031	1.3494219	0.1641426	0.193204
1.0878701	2.0044295	2.4022037	0	2.2372884	1.8407653	0.7663831	0.1695402	0.4806787
0.4622618	1.8511194	2.7274195	0.5854433	0.8880711	2.1542916	0.9997114	0.439038	0.0982848
0.808591	1.1512094	1.1042024	0.0467006	1.1667797	3.6035373	1.5672047	1.383331	0.1401242
0.7437304	1.3855967	1.1284914	0	1.2100147	2.181357	0.2998308	1.1048734	0.0405418
0.592158	1.844707	0.6445024	0.097476	0.8494389	3.4457002	0.7763567	0.9679801	0
0.5774409	1.5456225	0.5447821	0	0.725174	1.1054099	1.8078907	0.6381202	0.0622599
0.7540323	2.2043286	1.578504	0	0.8823692	2.9798607	0.4013028	0.0538064	0.0844725
0.8028963	0.9315318	1.0597014	0	0.4255667	3.0850167	1.2781632	0.1782366	0.0732718
0.740539	1.3465315	1.9735378	0	0.8890062	2.2131917	1.2120712	0.4682184	0.1826923
1.3358834	2.6614988	1.5355056	0	1.7816115	4.4612273	0.4072984	1.600127	0.6298462
0.7788814	2.4112906	2.1935194	0	0.8777443	2.9501884	0.5112656	0.4701984	0.0540844
1.0054719	1.5871249	1.0827707	0	1.0434145	3.4492312	0.683921	1.1124333	0.0648829
1.817746	2.3389663	1.5251167	0.1079556	0.7168592	3.3270262	0.6022668	1.3752897	0.2021356
0.4841897	0.2623129	0.4824353	0.4062101	0.5383395	2.8340235	1.1716552	1.0287106	0
0.1200865	0.5922537	1.5956946	0	0.5550308	2.6031694	1.7154541	2.1160986	0.0550568
1.2359721	2.563451	1.7352189	0	1.0941009	2.1724875	0.4938522	1.6946574	0.3123167
1.1428708	2.2369212	1.2343787	0	2.153935	2.8172958	1.0611541	1.5086822	0.1342209
0.5586595	0.6987519	2.5764012	0.151729	1.6915788	1.6116918	1.3757345	1.8482373	0
0.5960764	1.1679359	1.1241958	0.0651587	0.6139088	2.7994396	1.7159812	0.3460775	0.0754642
0.6366362	1.8890841	1.431623	0.1015156	0.2584586	3.732356	1.4815573	0.8914192	0
0.8595721	1.093763	0.7466567	0.4542812	0.514905	1.2905424	1.2140626	1.4205632	0.1430015
1.1759397	1.4410585	3.2785498	0.0381552	0.7409707	5.3461166	0.6864333	0.1436546	0.0870554
0.4005379	0.9393018	0.5247658	0	1.3502709	2.1054099	0.9871753	1.7400641	0
0.7685019	1.0436245	1.0292055	0.0344975	0.8987114	3.8552024	0.6853571	1.8302553	0.0268709
0.6263924	1.9724721	1.2965455	0.6248963	1.3467584	3.1171464	1.5639879	0.8857305	0.0593553
0.3226204	0.9381729	1.9707432	0	1.4190534	3.8817625	0.8042188	2.2106694	0
0.5423076	1.0031704	0.7536045	0	0	2.5729382	0.1377658	0.6620232	0.0468403
1.745854	4.2095235	1.8160808	0.0500489	1.6948208	2.0151883	2.3353975	3.2050484	1.2113238
1.1825652	1.7591132	2.2077679	0	1.1669082	4.1997352	2.1144337	1.0835197	0.3042774
0.8313098	1.0878023	1.2297725	0	0.9690123	3.4205902	1.287295	0.7246503	0
0.1658155	1.1289532	1.4020673	0	1.6075315	3.4382929	1.417974	0.0513025	0.0408223
0.3109217	1.7417043	0.5939752	0	0.9156356	2.8224854	0.920522	0.1680001	0.0690147
1.2580968	2.5128084	1.9099651	0	0.3552418	2.973299	1.5041627	0.6432097	0.2899525
0.4268551	2.3045987	1.3286642	0.2826177	0.5089357	1.6731041	1.4678533	1.6392785	0.0935602
0.4266405	0.955536	0.8060353	0	1.2030132	2.1446011	1.1693478	0.6271399	0
0.9940004	0.4346683	1.0844045	0.0732718	1.5496199	3.4960916	0.7616257	1.7847971	0.6464392
1.177854	1.5674968	1.6739182	0	0.7311832	3.0811351	1.3698294	1.7777038	0.1245928
1.4478965	2.0856286	1.9199883	0	1.4489538	4.4935577	1.0324535	1.0601858	0.5445843
0.4981487	1.0960594	0.5690049	0	0.6004601	1.2420232	1.0906502	0.841168	0
0.4765372	1.539928	1.7138278	0.1547128	0.6125884	2.1486413	1.6870607	0.4133774	0
0.6996405	0.6857159	2.5075663	0.311038	0.8212204	1.6459783	0.6474527	1.0413832	0

0.6072948	1.4881551	3.3584385	0.0657102	1.3744553	4.4796445	1.3101074	0.3482324	0.0257376
2.1976243	4.3217213	2.1795747	0.2660769	2.4214702	2.456666	2.1233903	2.3715124	0.6577924
1.1300083	0.9237197	1.3497616	0.4614238	1.1957885	3.8535566	1.9589911	1.2016966	0.1857397
2.452121	4.6799107	3.1369706	0	2.8668486	2.5188372	1.6344306	1.8515991	0.621712
1.0580394	1.5603237	2.0053641	0.5617905	1.2200203	2.8463128	1.271605	0.8461523	0.1096949
0.651775	1.7273542	1.9744559	0	1.7007948	2.7719912	1.1521185	0.6304987	0.1644001
1.9826925	2.1394695	1.839234	0	1.0518593	1.8296466	1.0497005	1.4213714	0
0.4340277	1.6350578	0.2747686	0	0.3395935	2.0562367	1.2313095	0.8181143	0.0847446
0.2537473	1.8789215	0.5127831	0	0.7441611	3.4649688	0.7532622	0.3901174	0.4532278
0.2760797	0.9626601	0.1712068	0.248049	0.5443865	2.1884959	1.1228052	0.9386998	0
0.2744108	1.7088052	1.1798295	0.047678	0.9587313	2.2460128	0.4812989	0.0919367	0.0732718
0.5619859	1.2801843	1.1591125	0.9128798	0.705049	2.4501159	1.2708877	0	0.0768327
1.117961	0.8548734	1.9763636	0.0378742	0.8511194	3.6386184	0.6837414	1.849639	0.2204538
0.4933399	0.8439035	0.5913922	0	0.2635152	2.9846987	1.6703418	1.5535571	0
0.9038867	2.2946355	1.780688	0	1.3933064	2.8307015	0.9914629	2.018741	0.0551957
0.2028878	0.5065004	1.5901469	0.3569326	0.9368923	2.7731313	1.1997509	0.6867022	0.0344975
0.6475448	1.131787	1.4002647	0	0.8302553	2.24373	0.7270927	2.4810664	0.0354832
1.061638	1.8392743	1.6638	0.0600474	1.278639	2.3454532	0.8745216	0.6611111	0.0919367
0.4560702	1.5590023	2.2782227	0	0.7457106	3.1786509	0.3922075	0.8922744	0.0437644
1.288831	2.2198036	1.6462548	0	0.915865	2.5494967	1.3255591	0.6516832	0.0903115
0.9203695	1.1541295	2.2945472	0	1.6325474	3.1840263	0.6770797	1.3173041	0.0790196
0.998051	1.162919	1.535406	0	1.9301696	2.44151	0.7359554	1.4398359	0.1211481
0.3923174	1.6251302	1.8810381	0.067088	0.2655569	4.2691529	0.9508416	0.7231653	0.102591
0.309991	1.3779011	1.7450221	0.6296597	1.7534334	3.098268	2.9390761	0.439889	0
0.7760197	1.6613848	1.9645835	0	0.4723838	3.6953814	0.7560839	0.9927684	0.2102642
0.500904	0.8742855	1.2188432	0.0743684	0.9316831	3.0251706	0.5566992	0.4641455	0.2690331
0.1078218	0.7330072	1.6370537	0	0.6636634	3.2712613	1.1154994	1.3106891	0
0.2395202	1.7927304	2.2163928	0	0.6971511	2.3854863	0.3170725	0.4938522	0.075738
0.96857	3.1552959	0.9779027	0	1.0341453	2.0150693	1.1551663	0.8663146	0.0657102
0.4304994	0.6183326	0.6493855	0	0.6434868	2.8917691	0.6983963	0.2711866	0
0.6641187	1.9236056	2.0413832	0	0.9795132	3.4225021	1.1005068	1.0260918	0.1741507
0.6891202	2.3284918	1.2290957	0	0.782073	1.8972792	0.4138107	1.1883376	0.0423642
0.3669236	0.7704485	1.6144743	0.1587248	0.9104257	1.8896293	1.1404515	0.7886022	0.0257376
1.3077782	2.5955514	1.7891867	0	0.4978423	1.7578767	1.0310422	0.2172307	0.0900404
0.153935	0.7923556	0.9067367	0.217603	0.3524196	2.1021878	0.1733833	0	0
0.3441467	0.3365119	2.1883693	0.0551957	0.921284	0	1.0061903	0.4653999	0
0.4475263	1.6255978	0.7353489	0	0.5065004	1.9319477	2.5848423	1.1434587	0.067501
0.2097653	1.2875314	2.1076209	0	0.9159415	4.7719595	0.4592218	0.1983684	0
0.827006	1.9493482	1.0977456	0.0546401	0.7417474	2.9325715	1.8250529	2.32112	0
0.8545543	2.2321081	0.9235676	0.1314578	1.3902274	2.6005076	1.5402257	0.878843	0.0522768
0.9500951	1.8146325	1.9554244	0	1.1286233	2.5670343	1.877705	0.7638365	0.1382902
1.1710787	1.1596938	0.2851063	0.0289934	0.8646517	3.6220401	0.5005981	0.3541136	0.3776235
0.8116354	1.9110396	1.5928756	0	0.7865127	3.504989	1.4821771	2.4857875	0

0.5041627	0.5564049	0.5848663	0.4564908	0.9772433	1.7453664	0.9355352	0.628167	0
0.231801	0.7892702	0.6601985	1.328779	0.9622899	3.3366833	1.165301	0.6442255	0
0.3701643	1.0980152	2.2817279	0.0597706	0.605115	3.2235152	1.2185952	0.4990677	0
0.8520788	1.0612232	1.548042	0	1.4107753	4.4481609	1.4700943	0.4329594	0.0779266
0.4933399	1.2668765	1.1172961	0	0.9331194	2.9267585	0.4541759	1.3155083	0.1536757
1.1108309	1.7694756	1.2794713	0.0261627	0.5062973	1.6467617	2.5112909	0.4792306	0.0601858
0.4810922	1.3922075	2.8269044	0.111432	0.6205864	4.0297797	0.7731524	0.7030121	0
0.4602708	1.3371402	1.7793018	0.1837088	1.1200201	0.2974849	1.562621	0.5285713	0
0.3836075	0.9926234	1.2903654	2.0120323	0.9371937	3.2720979	1.912305	0.7393297	0
0.4143521	1.345226	1.1130337	0.5189379	0.6245221	2.9655996	1.3383388	0.9907371	0.0784732
0.6250834	0.8116354	0.9486008	0	0.8448677	3.3309025	0.580435	1.1071522	0.1095612
0.22231	0.6694804	0.1604039	0.9074291	1.1796384	2.1836771	1.1615008	0.3297551	0.1834548
1.4254056	1.4252444	3.0681893	0.7003509	1.9012241	3.6660976	2.3558902	0	0.1955996
1.0913952	2.4513298	1.2776277	0	1.009992	1.3585088	0.8734983	1.4924172	0.0954519
0.2684344	0.4532278	0.3719491	0.1499097	0.4950811	3.7324971	2.09815	0.1742786	0
1.0720372	1.6644828	1.6128243	0	1.2591218	2.4168668	0.8621141	1.5145515	0.1307991
0.4212637	1.0585243	1.3470987	0.1613073	1.0194884	2.635708	1.23444	1.0173518	0.0187766
1.9912452	2.5120499	1.1696044	0.0549179	1.3306731	3.1117658	0.7651104	0.5405233	0.2751263
0.4080596	0.2571313	0.9114998	0	0.3171883	2.3218704	1.4544918	0.5004961	0
0.5170237	0.7417474	0.7394161	0	0.851839	2.8682719	3.0418212	1.1804662	0.0546401
0.9048887	0.3600833	2.0043216	0	0.85591	3.4492576	0.4811956	1.4025039	0.0309716
0.2983064	0.8044666	2.1278974	0.1027253	1.0025945	4.6589198	2.1239641	0.1944653	0
0.3451693	1.4044672	2.2249664	0.5739561	0.7879339	3.6282603	1.9253918	0.1315895	0
1.200881	1.7142675	2.0949116	0	1.1140338	3.0630197	0.9279723	1.511721	0
0.6327335	1.4205632	0.7382058	0	0.3972553	1.1748538	1.0653655	1.4074615	0.2067682
1.0203422	1.9785251	1.9936381	0	1.9626601	2.588061	1.0627434	3.6403317	0.167101
0.376846	0.6618408	1.9772433	0	2.0546749	3.7871817	2.3908052	0.419431	0.1231364

AP001469.1	AC009268.2	AC092279.1	AL161431.1	HMGN3-A	NR2F2-AS1	AL358472.5	AC010999.2	ABCA9-AS
0.6324544	0.8800978	3.2184247	0.0429244	1.6257381	0.4496406	1.3309599	0.4716557	0.0723116
0	0.5499156	0.953675	4.1481288	2.2013516	0.6588284	0.6903724	0	0.6912661
0.220949	0.1824381	0.6595593	4.1971577	1.2886539	0.1600166	0.6181447	0	0.1142337
0.3622174	0.5509991	0.9616974	0.6061579	1.8322824	1.2546545	0.8786861	0	0.5479433
0.6922486	0	1.3276874	0.016211	0.7985897	0.5711428	1.0915983	0.3656925	0.0274372
0.8157394	0.6954594	1.5062973	0.0395596	1.3523631	0.9281239	0.7400209	0.212009	0
0.3251561	0	1.0061903	3.6425514	3.9515225	0.1589833	0.1998765	0	0
0.1327742	0.5568953	0.5568953	0.0597706	1.0551262	0.7142235	0.2778658	0	0.0257376
0.4476321	0	1.8530375	0.2530211	1.6686184	0.511468	0.4013028	0.1843438	0
1.136257	0.9095811	1.7076131	0	1.4941596	0.4669664	1.5101518	0	0
0.4410585	0.8293625	1.2769135	0.5046712	1.1893502	0.4064278	0.1441769	0.4565959	0.3074285
0	1.1658798	0.7394161	5.2840921	1.642563	0.4746697	0.123931	0	0
0	0.3336527	1.7707022	0.7161569	2.8550927	0.1399933	0.5550308	0.3776235	0.261591
0.1133004	1.069221	2.7384652	1.4776255	0.9009537	0.4625759	0.3652446	0.2055176	0.6598332
1.340961	1.3704433	1.260387	0	1.5138949	0.3229665	1.1684495	0	0
0.3963787	0.4714476	1.0490731	2.4401017	2.7751348	1.0004327	0.6393248	0.3743996	0.9154062
0.4240621	0.1625968	1.5493242	0.0457225	1.6957266	0.4957975	0.640991	0.2424501	0
0.4043582	0.2602666	1.0812714	0.0989585	0.963918	0.2809563	0.7151905	0	0
0.1754288	0.7750927	1.4605853	1.662479	2.2888015	1.5690536	1.116897	0.2409859	0.7864291
0.3156822	1.2195248	1.6928289	0.0170667	0.883699	0.5794698	0.588037	0.2675957	0.1261799
0.690283	0.5848663	1.7936462	3.7723185	1.8980148	0.1203519	0.5368487	0.2530211	0.1565261
0.2885358	0.6239605	2.4261305	0	2.6622055	0.090447	0.9463566	0.2719037	0
0.3644604	1.0381552	2.2254292	0.0226164	1.5662794	0.2429378	1.0096338	0.1823109	0
0.5775376	0.7206287	1.7574073	0.5433969	1.5967444	0.9368169	0.8755442	0.3605328	0.059909
0.0867838	1.0431345	1.7948525	0.0576929	2.0115672	0.3894567	0.2852246	0.1585956	0
0.1268407	0.5352567	1.455439	0.4576468	1.0924781	0.3096419	0.4050121	0	0
1.458382	0.2118844	2.0846766	0.2272482	1.3345111	1.1407132	1.4695214	0.1651723	0.0681893
0.6948356	1.3289513	2.0269771	0	1.5007001	0.2519311	0.9868114	0	0
0.5268701	1.0547096	0.9276689	4.8410221	2.4886692	0.3680419	2.5973405	1.1086917	0.0467006
2.3040291	3.2575161	5.2954659	0.127105	4.7477687	1.8838749	1.8068602	1.073786	1.0924781
0.4476321	1.3678183	1.0809987	0.2357271	1.5739077	0.0666748	0.3618806	0.1054099	0
0	0.3303289	0.3331947	0.0329472	1.5242142	0.0938306	0.2456174	0	0
0.1466552	0.3351402	1.6763577	0	1.5257682	0.1406478	0.7850064	0.0932897	0.0565835
0.1918153	1.4527536	1.3554674	0.2092662	1.9573946	0.1450905	1.211573	0.4850146	0
0.2025118	0.5997939	0.7881845	0.0561673	1.2933116	0.2994791	0.8022346	0.154324	0.0321008
0.2916036	0.7688407	0.6854468	1.4920583	1.2963106	0.4905701	0.5091384	0.1633699	0
0.7517208	0	0.9868842	0.0900404	1.353662	0.2970153	0.3315906	0	0.4487953
0.1673579	0	0.4786095	5.6669639	1.0836559	0.305095	0.2832106	0	0
0	1.6637089	1.4953882	0	1.1100959	0.5263693	0.6053995	0	0
1.3242346	1.2097029	1.8544346	0.2057678	1.6216651	0.718526	1.2177891	0.1384213	0.111432
1.0285692	0.826762	2.4297766	0.0284277	3.6364158	0.2184712	0.8775087	0.1172961	0.2251515

0.1145003	1.2714257	3.8552223	0.4077334	3.1224077	0.4252444	1.2088294	0.2077679	0.0863762
0.6665748	1.444932	1.1353379	4.3691593	0.8670266	0.6084308	0.8291189	0.1506896	0
0.7833309	1.4659745	3.2300031	0.0251706	3.7204536	0.2785796	1.8813122	0	0
0.0855606	0.5343605	0.9817796	0.0936954	1.5423076	0.3702759	0.4333868	0	0
0.9754835	0.8377011	1.4303923	0.0201999	1.2435472	0.2281106	0.2270017	0	0
0.2744108	0	0.8201585	0.888227	1.3940202	0.3669236	0.2445218	0	0.0553345
0.1580785	0.8868232	1.2842772	0.0243197	1.6559929	0.2193389	0.5620836	0.1949696	0
0.9095043	0.65306	1.3725621	0.2387869	1.0153548	0.7204536	0.8273313	0	0
0.0437644	0.7456246	1.1578845	0.3527585	1.5073634	0.1307991	0.7232527	0.0809305	0.0164963
0.5813995	0.1824381	1.1890338	0.0847446	1.4407928	0.2553194	1.297837	0.1419559	0
0.1430015	0.1729995	1.2657969	0.0163537	1.8431799	0.3528715	1.1636919	0.3707223	0.4539653
0.844707	0.924404	1.6182856	0.5430999	1.8007446	0.108491	0.9393018	0.7557422	0.0326651
1.5612528	0.7100846	2.5972928	0.0406821	2.5072365	0.514804	0.8501593	0	0
2.676305	2.0639631	4.7243247	0.1706942	2.4484429	2.7668566	1.7273977	0.9962682	0.9535757
1.3196179	1.4998841	2.7588788	2.4508549	1.8056226	0.4912887	0.841007	0	0.0784732
0.2414741	1.0794293	0.8671057	0	1.8301742	0.5316684	0.4018489	0.2273714	0
0	0.623024	0.7881845	3.7334086	1.2776277	0.6984852	0.3698294	0.2715452	0
0.3018217	1.0957894	2.2314631	0.0415234	2.8755442	0.3473255	0.8170502	0.2471984	0.0526941
0.3766238	1.1152996	1.2270633	0.0373119	1.1366508	0.3041605	1.0368901	0.1528973	0.0318186
0.9363647	0.8944492	1.7471296	0.0652966	2.3813664	0.2010065	0.6393248	0.1784916	0
0.6075789	0.8221183	1.4297499	0.0328061	1.5192399	0.2636354	0.4534386	0	0
0.5439907	1.1887174	1.336112	0.1937087	2.390255	1.0411028	1.0274372	0.166844	0
0.9215887	0.6999069	2.1871977	0.0594937	1.8248493	0.1837088	1.4271771	0.3096419	0
2.8994141	2.3133233	4.1660084	0.2246989	2.6152044	1.7009723	1.8660771	1.3179215	0.7831353
0.5657921	0.587845	1.1561377	0.8344685	1.250052	0.4715517	1.328779	0.470615	0
0.4633086	0.3879139	1.6532435	0.0394192	1.5193406	0.3122005	0.2899525	0	0.1909309
0.24123	0.1994996	1.5908176	0.0190614	1.6074368	0.3088268	0.6180507	0	0.032383
0.2751263	0.5977934	0.5541468	0	1.82725	0.1527675	0.7045179	0.2110122	0
0.2980717	0.5066019	1.628587	0	1.0316069	0.1423481	0.3823906	0.1473067	0.030548
0.3325074	0.5082258	1.5122775	2.5089103	1.5085808	0.4022856	0.7747554	0.1478277	0
0.7815695	2.1026245	2.406836	0	3.4573185	0.2753647	0.904195	0	0
0.0439044	0.8714494	0.5566011	0.0097771	1.5794698	0.2110122	0.574925	0.1578198	0.1272371
0.1804025	0.4066455	2.1382575	1.1749177	1.4818672	0.5181322	0.6121166	0.1696685	0.3769571
1.4434475	0.2327835	2.2798873	0.230326	2.239398	0.2423281	1.2944884	0.4884122	0
0.2753647	0	1.3137683	0.0110662	2.4950811	0.2465905	0.1681285	0.2594232	0.1774714
0.3967075	0.2075181	2.1565584	0	1.539928	0.1606621	0.6653018	0.3070787	0.0336521
0.6716557	0.3671474	2.913416	5.7780351	2.5718709	1.1196218	0.7024802	0.5305702	0.1800205
0.0536674	0.4552286	0.4515408	0.0931545	1.1430668	0.133432	0.3035762	0.0990932	0
1.196607	0.729357	2.1095612	0.0169241	1.0001443	0.2073931	2.5344601	0.5892841	0.9895024
0.2875905	1.0554733	1.4613714	0.0230424	1.1818023	0.5121763	0.5398288	0.186247	0
0	1.1330374	1.3426685	0.3577209	1.7973036	0.1925729	0.147437	0	0
1.4513298	1.0426443	2.1860568	0.0271541	2.8110599	0.3261922	1.0763539	0.1127668	1.1044709
0.5455731	0.4015213	1.5007001	0.0206267	1.4157046	0.0878701	0.9016489	0.1673579	0

0.8191777	0.8869013	3.6547678	0	3.2212584	0.0727232	1.072243	0	0.2408638
0.291132	0.4954905	1.7104814	0.0176369	1.6702965	0.3283769	0.5295711	0.1437852	0.2735756
1.3257893	1.5065512	2.8179506	0.063641	1.804384	0.4709273	1.2922517	0	0.0364682
0.2389091	0.4115347	1.6690721	0.6355222	1.5418122	0.241108	0.4822287	0.116897	0.047678
0.1277653	0.294312	2.0256313	0.0575542	2.4393838	0.235482	0.2182232	0	0
0.2332745	1.0512329	2.6771023	0	3.9942901	0.1531568	0.6243349	0	0.1777265
0.3386811	0.897008	0.7107018	0.054779	2.1189909	0.2133784	0.3906677	0	0
0.4137024	0.2665167	1.4486896	0.0517201	2.2029192	0.4871776	0.7615406	0.2086421	0
0.8385888	0.4986594	0.8583789	0.3885753	1.4372277	0.316725	1.0303361	0	0
0	1.2231754	1.722772	0.8580606	1.7622638	0.0093472	0.8063653	0.4234168	0.3725064
1.0235393	1.23842	2.1176286	0.1206174	1.9151767	0.3925372	0.8241568	0	0.0832474
0.417488	0.2691529	1.4154342	0.1983684	1.4674882	0.2585792	0.4924172	0	0.0662614
0.2919571	0.1500397	3.1238648	0.055751	1.555129	0.2748878	0.8406042	0.1164978	0.2026371
0	0.3805078	0.4955928	0	1.0864441	0.3758457	0.1011122	0.3002995	0
0.1461338	1.1191569	0.9449334	0	0.9631782	0.3726178	0.3043942	0.3780676	0
0.3999914	0.8326065	1.2461648	2.6625701	1.6874191	0.3468718	0.2482919	0	0
0.6691175	0.444243	1.1809117	0.1895399	1.3596898	0.2740529	1.4511188	0.2440346	0.0263043
1.1234676	0.9425333	1.9343278	1.3638439	2.3887131	0.2994791	0.9052739	0.3392514	0
0.1647863	0.9161709	1.91219	4.8460319	3.5067796	0.7828279	0.61664	0	0.9958824
0.2542312	0.3939653	0.7441611	0	0.7290959	0.3639	0.6473606	0.1640139	0
0.1742786	0.7028348	1.3025238	0.013498	1.061638	0.399007	0.6470842	0	0.0229004
0.215368	0.5579737	1.3016462	2.6197886	3.0657619	0.704872	1.1323136	0.5682267	0.8038057
0	0.4527009	1.4809372	0	1.5005981	0.2046416	0.3401634	0	0
0	0.9325148	2.1803707	0.1401242	2.1433934	0.5308698	0.5768607	0	0
0.8304176	0.6029319	1.4433945	0.0221902	1.9322502	0.2577348	1.0428544	0	0
1.8350752	2.0264814	2.3529562	0.5462648	4.3860936	0.4981487	1.4131608	0.1351408	0.0554733
0.986957	0.590051	2.9947788	0.3215818	1.197488	0.3626663	0.864097	0.2554403	0.0275787
0.7677395	0.1563966	1.3428392	0.0439044	1.9680908	0.5831339	1.0521376	0	0.0250288
0.7363884	0.3223897	0.9809027	0.078883	1.4787648	0.7210664	1.2630945	0.1322478	0.0272956
0.0432045	0.4560702	1.2868811	0.0096338	1.3675388	0.1920679	0.3427823	0.3618806	0.0483757
1.1900459	1.3596336	1.9160944	0.276318	2.4500895	0.485839	0.7097318	0.2017593	0.0425043
0	0	0.4286784	0	0.7334411	0.1145003	0.1184926	0.628447	0
0.3248106	1.8450283	1.1192234	0.0159257	1.7300095	0.0457225	0.3914378	0.2501127	0
0.2898345	0	1.4852722	3.1780453	1.6464392	0.7128159	1.2168584	0.5029416	0.3450557
0.5172253	0.8402819	2.5882529	0.0989585	2.7400857	0.9040409	1.0978804	0.4896457	1.061638
1.072929	1.1368476	2.6834045	0	0.9485261	0.7911056	0.7213289	0.7565964	0
0.4977401	0.5884208	1.4466268	0.0216219	1.8390727	1.2016339	1.104538	0	0.0721744
0.6148512	0.6384909	0.8237494	0.3969267	1.2864672	0.3034593	0.7392433	0	0.5676428
0.1982427	0.3652446	1.4106125	1.8509594	1.1048063	0.3263072	0.5534588	0.287945	0.015783
0.4100154	1.1028597	2.3882447	0.0147838	1.8690423	0.9213602	0.768756	0.6925165	0.6428402
2.1982112	0.9161709	3.3892639	0	2.0423642	0.7165959	1.0960594	0.3810618	0.0216219
0.4814023	0.281075	0.6480972	4.6402565	1.7597098	0.3518544	0.3019388	0	0
0	0.8042188	1.499425	2.2183162	1.9028454	0.3878037	0.1821838	0	0.0538064

0.9393018	1.0541539	1.5234616	0.0230424	1.126246	0.1137005	2.1990598	0	0.0581086
0.1240634	0.2861715	0.5932103	1.7563829	0.7684173	0.6707497	0.3968171	0.1534162	0.0319597
1.1332347	2.1765781	2.3640401	4.6319482	1.9055049	0.3612069	1.4739947	0.3879139	0.3350258
2.0379444	0.90134	1.2179131	0.0139267	2.7378382	0.3912178	0.9553128	0.5017194	0.2618317
0.6406209	1.242633	1.3259044	0.0436245	1.5744406	0.3864799	0.5378427	0.6076736	0
0.4075159	0.2624332	0.7726458	0.0507455	1.8476763	0.1984942	0.3649086	0.2053925	0
0.6574571	0.7701948	3.262809	0	1.5162673	0.7576207	1.1010449	1.0579008	0.1476974
0.3020558	0.8906412	1.373509	3.6329312	0.9792937	1.0332292	0.8650478	0.1493894	0.0614306
0.3793992	0.6336637	0.9775364	2.6173455	1.0980826	0.4603756	1.514299	0	0.078883
0.3109217	0.3046279	2.0655723	0.0450235	2.1584986	1.1554254	0.374956	0.2391536	0
0.1163648	0.8648894	1.4144062	0	2.0869196	0.3013535	1.3109217	0	0
0.3700527	0.8934399	0.8620347	0.0366088	1.8808031	0.5885168	0.7400209	0	0
0.2723816	0.4919044	0.9318343	3.6299744	1.4170559	0.1163648	0.5257682	0.1082233	0.0221902
0.2729787	0.6877774	3.463714	0	2.1514692	0.0625362	0.7944367	0.108491	0
0.1760674	0.2123826	1.1439158	0	2.2070807	0.1952216	0.0802483	0	0
0.4972291	0.6551686	2.0279325	0.0184917	1.4883093	0.2095158	0.0728604	0	0.0313952
0.6956375	0.8133605	0.7624764	0.1131671	2.6028132	0.2281106	0.6788832	0.3013535	0
1.2148087	0.3438058	1.0258793	0	1.4390912	0.5426048	1.3521935	0.2705887	0
0.3589588	0.5462648	0.4266405	4.8431548	0.9737215	0.5166203	0.2215678	0	0
0.1131671	0.8462325	1.0101353	0.0255958	0.8760159	0.3036931	0.5884208	0	0
0.7979261	0	1.7620511	0.0144981	2.5523525	0.3053285	0.8264366	1.2002532	0
0.2048919	0.9504684	2.4984551	0.0475384	1.0499792	0.068602	1.1000357	0.8938281	0.1183597
0.4271771	0.8815863	0.9712216	5.3338995	1.5170741	0.3189242	0.2961931	0.4038131	0.0457225
0.0475384	0.2190911	0.7303139	2.1951901	1.3149865	0.4401017	0.406319	0.2492633	1.5630605
0.5382402	0	2.5636219	0.0353424	2.0754299	0.1004395	0.6304987	0.1452209	0
0	1.2577951	0.5861162	0	1.0829069	0.531868	0.1851054	0	0.1074201
0.797179	1.1104969	1.2047042	3.1710787	2.4371212	0.2172307	0.9128031	0.2600257	0.0281448
0.3447149	1.2936648	3.3301998	0.0170667	1.5580717	0.0727232	0.4655044	0	0.028852
0.6612023	1.5334637	1.9353089	0.3034593	1.4276598	0.8007446	0.4240621	0	0
1.3651326	0.9730602	2.6727648	0.0137838	3.0187054	0.3501577	0.9166295	0.5775376	0
3.2332439	2.8045286	6.3763442	0.0763539	3.0406908	0.9383234	2.5450911	1.7122876	0.597698
0.4460444	1.0444641	1.4016851	3.1264113	1.0696334	1.1185591	0.4681141	0	0
0.5676428	1.7469576	1.349988	1.6564048	1.9836412	0.4514353	0.2779847	0	0
0.1397314	0.5823633	2.8938281	0	2.61584	0.1052758	0.4891319	0.2516888	0.2509616
0.5086315	0.7973451	1.3255591	1.08882	1.7077456	0.6343145	0.8888504	0.294312	0
0	0.2376861	0.8771945	0.9266067	1.818687	0.6919808	0.175301	0.1856129	0.2529
0.1885275	0.4234168	2.1349437	1.7217226	1.2801843	0.5014137	0.5166203	0	0
0.458697	0.6067265	0.8990209	0	1.2000649	0.5962673	1.5288213	0.2635152	0
0.193204	1.3155083	0.3053285	0	1.2211966	0.4213714	0.2503553	0.0938306	0
0.0947766	1.5813995	0.6317096	0.0528332	2.1832642	0.4372277	0.5050779	0	0.0182069
0.7381193	0.7652801	1.4721758	0.197488	1.6399267	0.3607576	0.8131963	0.237319	0
0.7290959	1.0875986	1.3909428	0.0611541	1.2608085	0.6410836	0.6597419	0	0.0691522
0.8793921	0.4961044	2.815104	0	1.9143732	0.576377	1.2668165	0	0.044604

0.1654296	0.5315686	0.5521803	6.0705589	0.7966808	0.0990932	0.2149951	0	0
0.2681948	0.3215818	1.2632748	0.4661311	1.8616378	0.3343394	0	0.252779	0
0.1450905	1.1968588	1.9342145	2.4848342	1.6622967	0.3574957	0.5520819	0	0
0.2026371	0.4527009	2.306758	0.1147668	2.4487953	0.1843438	0.9490493	0	0
0.284751	0.3410749	0.901031	0.2087669	1.2545336	0.4058835	0.700706	0.4946716	0
0.2423281	0.6746414	0.7386382	0.4733194	1.3367975	0.3327366	0.516116	0	0
0.6590112	0.3595212	2.2174479	0.1060803	1.8264773	0.8185234	0.4940571	0	0
0.1300083	0.5470549	1.1135005	0.0295589	1.4977912	0.6977738	0.2219389	0	0.0982848
0.4379734	0	1.6382129	1.4288392	1.1991226	0.3341106	0.4833644	0.1646576	0.0343566
0	1.8972404	0.6698432	1.3599709	1.4280888	0.2649567	0.5766672	0.2736949	0.6242413
0.4447731	0.5273707	2.1985884	1.0718999	1.3720048	0.4528063	0.5632558	0.4205094	0
0.5678375	1.4243847	0.9961718	0.0430645	2.9078136	0.6589198	1.2612901	0.1751733	0.1072861
0.4077334	0.8466337	2.6802118	0	2.4727737	0.1655583	1.3286642	0.5450788	0
0	1.4252982	2.9579332	0	1.8539557	0.27453	0.9019578	0.4534386	0
0.1746621	0.3946239	1.5903864	0	1.397803	0.2856981	1.3076034	0	0
0.3161457	0.3097583	1.3948434	0.0607392	1.6490636	0.8331735	0.473735	0	0.0771062
0.1152996	0.2669964	1.1675506	1.5265196	1.1971105	0.4833644	0	0	0
1.5054337	0.9879029	1.7610725	0.2803625	2.010386	0.520045	1.3334237	0.516822	0
0.6054943	1.0142124	1.5556199	0.0487942	3.2149796	0.6569997	0.3515153	0.3709454	0.0278618
0.514703	0.3778456	3.8968628	0	1.4912374	0.0821576	0.2821432	0.4269624	0.2143735
0.756511	0.8817429	1.8425766	0.0240359	1.5658896	0.2329063	0.8829951	0.1935825	0.8717648
0.4029404	0.8379432	0.5532622	2.5988892	1.3887957	0.7927721	1.1422174	0.2028878	0.0427844
0.4162993	0.4944668	1.5637439	0	1.9929497	0.2068932	0.9330438	0.1433934	0.0149265
0.2891262	0.406319	1.5746344	0.0139267	2.3940476	0.1790014	1.0671568	0.1152996	0.0926134
0.1245928	0.2874723	0.8802545	3.4094181	2.0033864	0.5745375	0.4829515	0	0
0.7843365	1.2468337	1.8406042	0	2.3047739	0.6168282	0.9673162	0.104605	0.4592218
0.5352567	1.0689459	2.1689629	0.3064956	2.2049232	0.5062973	0.7763567	0	0.047678
0.4596415	0.9389256	3.0075723	0	1.2934293	0.1527675	0.6739182	0.1897929	0.1164978
0	0.2294649	0.7005285	0.0866479	0.9922609	0.4750849	0.2467121	0.3383388	0
2.0832247	2.850306	5.3675889	0.1114765	4.4339409	1.3754936	1.7970683	1.4157226	0.8759897
1.4782988	0.7502635	1.6210086	0.1023222	1.7162008	0.9894297	1.3644043	0.2740529	0
0.1868808	0.7451082	1.2417182	0.0850167	0.5576797	0.3555801	0	0	0.0727232
0.206018	1.1380936	1.0913952	0.6536104	1.8924687	0.0867838	0.2171066	0.0806577	0
0.6075789	0.3288365	1.4998331	5.2886502	0.7781245	0.3162616	0.6360793	0.4777809	0
0.6892097	1.7348722	2.728443	0.0425043	1.8809989	0.5275708	1.4540706	0.4673838	0
0.3639	0.6096604	2.8454298	0.3242346	0.7943535	0.3841604	1.0531113	0.1819294	0.0381552
0.0922074	0.8527979	2.2547754	0.1582077	1.644087	0.7726458	0.1587248	0.1681285	0.1028597
0.1195554	0.7079665	0.9673162	0.0536674	1.6457939	0.3718376	0.2045164	0	0.0458623
0.254594	0.2107629	1.9932033	0.0201999	1.6651653	0.4963089	0.6995516	0.1642714	0.0342157
1.1146335	0.3782897	2.1509495	1.6010309	2.2532027	0.5682267	0.623586	0.1570437	0.2428159
0	0.9344788	0.9460571	0.1135672	1.5401265	0.2319239	0.2596642	0.097476	0.0199154
0.7280511	1.248049	2.2617715	0	3.2092038	0.083792	0.8024	0.4345615	0.2752455
0.6763577	0.7915224	1.5012608	0.0618453	1.4333868	0.9586571	1.213005	0.458067	0

0.3004166	1.4810922	1.2584586	0.2465905	1.4698339	0.2936059	0.9140287	0.1948435	0.8304176
2.3621893	2.6213056	4.2494201	1.3134394	3.3497946	1.8368263	1.8398387	0.9937106	1.3224858
0.4121854	0.7036323	0.9486008	0.1751733	2.6729231	0.5314688	1.2190911	0.3509498	0.2701103
2.5727927	3.1099205	4.9531977	0.2222481	3.9426318	1.8018622	1.7180218	1.7849855	0.9145263
0.7452804	1.071694	1.4020127	0.0177794	2.5746344	0.1787465	0.6746414	0	0.088413
0.7408844	1.0947766	2.1303379	0.8610025	1.9406178	0.3623296	0.6287269	0.3677065	0.0275787
0.5043662	0.8625902	2.2657369	0.1023222	2.6625929	0.2471984	1.1728075	0	0.0874628
0.1755565	0.3963787	0.6807744	0.9959548	1.5181322	0.077243	0	0	0
0	0.2152437	0.7428686	0	1.0517201	0.1978654	0.9473295	0	0
0.1079556	0.2509616	1.1880844	3.4862381	1.0839962	0.5159143	0	0.3688242	0
0.0783366	0.6277002	1.8736951	2.9983399	2.030901	0.0831112	0.3728407	0.2738142	0
0.0820213	0.7782927	0.849599	1.0199865	1.6046407	0.239398	1.7975111	0	0.031254
0.3390233	0.4046852	0.7326599	0	0.6189904	0.3634515	0.7987556	0.220949	0.0236102
0.2095158	0.8187688	1.5776826	0.141302	1.0880059	0.3776235	0.186247	0.1971105	0
0.8084262	0.2678354	1.8756621	0.1505597	1.1184262	0.4288928	0.7331808	0	0.0873271
0.1419559	0.7062874	1.7550585	3.530283	2.0950467	0.3124328	0.4483724	0.133432	0.0275787
0.9694544	0.4753962	2.0838261	2.059113	2.0097771	0.4034859	0.8080143	0.2624332	0.0563061
1.4646683	0.7555713	1.2528395	2.9808296	1.3645164	0.5721136	1.5782625	0.1787465	0.0374525
0.178874	0.6456094	2.8997943	0.0207689	1.3997727	0.1760674	1.0739573	0.3188085	0.0176369
0.9252399	0.4201859	2.9134543	0.021764	2.8297481	0.3049782	1.1099623	0.175812	0
0	0.1981169	1.1118993	0	2.4042219	0.3868109	1.0440443	1.0901082	0
0.5075156	0.2974849	1.2369518	0.0147838	2.3260483	0.2133784	1.1359945	0.2333973	0.1890338
0.1094274	0	1.9590653	0.1424788	0.3771793	0.3427823	0.4301782	0.1987456	0
0.380951	0.373509	3.2430445	0.1628545	1.9070445	0.8353582	0.6655747	0.1548424	0.0322419
0.4768483	0.348799	1.8117176	0.1352722	1.9262272	0.2657969	0.8786861	0.8789215	0.1969847
1.0376634	0.6525094	1.52682	0.4431823	1.1576258	0.4899539	0.6949248	0.0769695	0
0.1884009	0.7500062	0.8366513	0	1.1365852	0.1294809	0.5368487	0.231801	0.0492125
0	0.5089357	1.73773	0.0182069	1.1250559	0.1510795	0.3268824	0.1480881	0.0308304
1.4823837	0.9814874	2.0724489	0	1.6398804	0.6529683	1.2129428	0	0
0	0.8931292	1.5393822	0	2.9351768	0.2006299	1.1106305	0.2194628	0
1.434348	0.7408844	1.5195419	4.0774994	2.4479759	1.1319845	0.8664728	0.5990321	0.1674864
0.8199951	0.6991962	1.7536045	0.1531568	1.1115655	0.2091414	1.1248575	0.1628545	0
0.3446013	1.153546	1.3740101	0.1059462	0.9194545	0.203765	0.4220177	0.1947175	0.0206267
0.1861202	1.6979072	3.8365604	1.1373068	1.9852454	0.1723595	0.8555911	0	0.0957219
0.1241958	1.6772151	0.3368546	4.8192237	1.1105637	0.2053925	0.1100959	0	0
0.1751733	0.2113861	1.5932103	0.0203422	1.3459073	0.0391384	1.4962067	0.4466797	0.3116194
0	0.3226204	1.3742883	3.9600854	1.5574836	0.3352546	0.3460775	0	0
0.0589399	0	3.1067503	1.6719727	1.8251343	0.0807941	0.8834644	0.3036931	0.8678174
0.0893626	0.3924273	1.6949693	2.2129739	1.8061591	0.3624418	1.3664761	0.1632411	0.0673634
0.3962691	0.4712395	0.8376204	0.4482667	2.6561989	0.418136	1.2590615	0	0.5581697
1.1267086	1.3662523	1.4256204	0.0501882	1.1662655	0.2214441	1.7934381	0	0
1.231371	0.7302269	1.7278769	0	1.5442381	0.2508403	1.6226961	0.088413	0
0.1545832	0.976217	1.2816092	0.1036655	1.4294821	0.9365908	0.7207162	0.1453514	0

0.3001823	0.810402	0.9272897	0.0480967	2.6329661	0.7629015	0.4220177	0.3662523	1.6730136
0	0.3691593	0.5000882	1.4483724	1.4430762	0.2666366	0.5066019	0.291132	0.1231364
0	1.9031926	0.4421208	0.4695734	1.0992952	0.5842891	0.1681285	0	0
0.2363395	0.7868472	1.4373343	0.0371713	2.2157718	0.2035144	0.5566992	0.6379348	0
0.3108054	0.4513298	1.1906149	0.1099623	0.9603634	0.6846392	0.4551233	0.0790196	0
0.8394759	1.0278618	1.4199703	4.5161097	1.8301742	0.1710787	1.2582174	0	0.032383
0.3779566	0.3149865	1.554245	0.0517201	1.3371402	0.1820566	0.4350952	0.3915478	0.0354832
0.4595365	0.2429378	0.940054	0.0236102	2.249415	0.0564448	1.3341678	0	0.3876934
0.3140584	1.4769001	0.6305919	0	1.6133902	0.2401311	0.514905	0.3828332	0.4473147
0.5639391	0.5725017	1.8524783	0.1554902	2.1910256	0.5520819	0.6435792	0	0
0.3447149	0.8677384	1.0474686	0.2086421	2.1259155	0.3274574	1.0294175	0.088413	0
0	1.0455129	0.5950262	2.5349829	2.1680001	0.1378969	0.9226546	0	0.3765127
0.4999862	1.3326793	1.8973953	2.5856356	1.5086822	0.6329196	1.9714424	0	1.3708338
0.7303139	1.4800583	2.8363281	0	2.884637	0.9225785	1.1547776	0	0
0.6604723	0.2356045	2.9450458	5.2056154	1.5624745	0.6195539	0.2532632	0.0949116	0.6123053
1.3355404	0.5816887	1.3791219	0.0160684	1.5862603	0.5662794	1.6753643	0.2514464	0
0.258338	0.8099083	2.6030506	0.026446	2.0435195	0.5239633	0.9911726	0.5058909	0
1.0647449	0.7030121	1.3829439	3.075892	1.7803941	0.5163178	1.2030758	0	0
0.2531422	1.1189577	2.1659441	3.6755563	1.475915	0.3393655	0.4503799	0.3779566	0.0170667
0.9325148	0.2653169	1.7233401	0	0.7291829	0	1.0650897	0	0
0.1281614	0.6485575	1.5388858	0.0576929	1.5544415	0.7112306	0.2188432	0.5212518	0.1200865
0.7939375	0.3772904	1.0122111	3.7245957	1.0144981	0.2727399	1.3881344	0.5443865	0
0.8556708	0.6722896	2.0381201	2.6588513	1.8880321	0.6959048	0.3623296	0	0
1.1225402	1.5451776	1.4870747	1.7813177	2.494134	0.7985068	1.1606621	0.1441769	0.1973622
0.3188085	0.6820339	1.8412888	0.3258469	1.523562	0.3860384	0.8474357	0	0
0.8386695	0.8503194	1.0275787	0	3.2126627	0.5608128	2.0853227	0.3176514	0
0.7376868	0.3022898	1.3900073	0.8941387	2.0641241	0.9882666	0.9447085	0.4408459	0.714839

AC107071.1	AC084876.1	AL596223.1	AL353804.1	AC073655.3	AC032011.1	AC243830.1	AL162632.3	DPP4-DT
0.6114557	0.4430762	0	2.901591	0.4460444	0.2611095	0.044604	0.4627853	0.4534386
1.7312701	0.8252972	1.0932897	1.992007	0.1519887	0.064331	0	0.0614306	0
0.6015064	0.5661819	2.1304038	1.6083835	0.133432	0.0563061	0	0	0.0597706
1.3455099	0.2213203	0.077243	1.7468287	0	0.2699906	0	0.6071054	0.1016501
0.5236623	0.5331646	0.3070787	1.407244	0.5367492	0.1991226	0.6653018	0.0984196	2.1457427
0.7208913	0.9131862	0.2274947	2.4311415	0.6721085	0.2045164	0.3002995	0.3012364	2.0163537
1.2849878	0.1976138	0.068602	0.8755442	0	0.2415962	0.3440331	0.0812033	0.4040311
0.0581086	0.2239168	0.1156992	1.6545272	0.2256451	0.3925372	0	0.1367164	0.7034551
0.9945797	0.325962	0.1713349	1.5762803	0.4681141	0.2747686	0.5509991	0.3785116	2.5079722
1.5052812	0.6058736	0.2722621	2.6305686	0.2754839	0.3811726	0.3175356	0.1680001	1.9616974
0.8312287	0.2722621	0.1567849	2.1834865	0.0973411	0.193204	0.3473255	0.2189671	2.4939035
0.1200865	0	0.0824301	1.4669142	0	0	0	0.1889073	0
0.9317587	0.3541136	0.2063932	0.7304008	0.3565946	0.343351	0.1654296	0.1969847	0.4077334
0.1881477	0.1920679	0.0336521	2.1504947	0	0	0.1037997	0.4188916	1.5572385
0.487795	1.4578044	0	2.1240303	0.5000882	0.117695	0.3742883	0.5319678	2.6743477
1.2316167	0.186247	0.9014945	2.1139671	0	0.0799754	0	0.0762854	0.4477379
0.4146768	0.5112656	0.0401209	2.2472288	0.1186255	0.09815	0.1525079	0	1.9292229
0.1863738	0.3582837	0.065848	1.6795589	0	0.1591125	0.0522768	0.0780633	1.4195928
0.6590112	0.2932528	0	2.0988238	0	0.4075159	0	0.3410749	0.2001277
1.636497	0.3066122	0.044604	1.7176051	0.0672257	0.1088924	0.0869196	0.0529723	0.2225573
0.7634966	0.1619522	0.1863738	1.5146525	0.1631122	0.1672295	0.0871913	0.0661236	0.5819778
1.3003581	0.366588	0.1321162	1.5454742	0	0.1107641	0	0.4248146	0.6730136
0.6335707	0.3223897	0	2.7040309	0.7056683	0.2716647	0	0	0.2208252
0.4724878	0.4114262	0.031254	2.2484741	0.2624332	0.2851063	0.0729976	0.108491	0.9042721
0.6612936	0.4050121	0	2.1799568	0	0.2910141	0.0402612	0.117961	1.2213822
0.3495917	0	0.0746424	0.9338747	0	0	0	0.2503553	0.5441886
0.7332676	1.2349917	0.0529723	2.9512707	0.5408208	0.1891604	0.1613073	0	1.1322478
0.2761989	0.4042492	0.1470461	2.7040752	0.2836848	0.3920976	0.3904476	0.2266318	2.0959244
0.5370475	0.5468574	0	0.7268312	1.5149554	0.0889557	0.8195864	0.1650436	0.1830736
1.4960277	1.8485578	0.6962164	5.040888	1.4003193	0.2683146	1.4908525	0.6592852	2.3212643
0.4272843	0.1901723	0.0977456	1.0007933	0.1915627	0.232538	0.1273691	0.2895984	1.1232689
0.4426516	0	0	0.3343394	0	0	0.1943392	0	1.9054664
0.6297529	0.169027	0	1.5133391	0.2484134	0.1071522	0.217603	0	0.4961044
0.6279803	0.1685137	0.1676148	2.5678131	0.1696685	0.329181	0.5079215	0.2574934	2.6899253
0.7381193	0.275007	0.024887	1.6955039	0.4545971	0.0309716	0.2707083	0.2211966	2.0101353
0.6535187	0.0783366	0.1518588	1.0216929	0	0.1273691	0.2331518	0	0.316725
0.1154328	0	0	1.7737854	0.2284801	0.1901723	0.3920976	0	0.2015084
0.3947336	0	0.3609822	1.3353117	0	0	0	0.2253983	0
1.0652966	0	0.0799754	2.3646285	0.8996397	0.3609822	0.3449421	0	2.8175414
1.3801753	0.643025	0	2.6602441	0	0.3460775	0.1350094	0.0522768	1.3123747
1.4515408	0.4792306	0	1.7923973	1.0231844	0.2178511	0.9167823	0.2467121	3.0075723

1.4655566	0.3651326	1.0020904	1.5070081	0	0.0835197	0.8113066	0.0797024	0
0.8885387	0.4950811	0.267356	2.119489	0.2705887	0.1172961	0.1815479	0.1119661	1.4631516
1.8735376	1.342441	0.1277653	2.3693548	0	0.2305719	0.2423281	0.1506896	4.1521915
0.5550308	0.1460035	0.0745054	2.3036931	0.5155106	0.0923428	0.1706942	0.2241638	1.055751
1.0311128	0.153546	0.3760681	2.5718709	0	0.8017381	0.1979912	0.0625362	1.3402774
0	0.6299394	0.0847446	0.8304176	0.7955176	0.1050075	0.1318528	0.2821432	0.7850064
0.729705	0.3438058	0.2368293	0.7391568	0.1834548	0.1880211	0.3182301	0.2131295	1.3107473
0.6773504	0.8610819	0	2.339451	0.3879139	0.1170301	0.4865599	0.0569996	0.4898512
0.2108875	0	0.0504669	1.1986827	0.0758749	0.0316775	0.1352722	0.5718224	1.3414165
0.5091384	0.3654686	0	1.2423891	0	0.523562	0.0359054	0.1054099	0.0597706
0.7746711	0.9320612	0.0427844	1.4888749	0	0.1540647	1.1307332	0.0507455	0.2629142
1.5257682	0.1462642	1.7223349	1.4158127	0	0.3885753	0.2578555	0.0594937	0.1901723
1.2208871	0.5393325	0.2020102	1.5523771	0.753091	0	1.0490731	0.4410585	2.9973466
1.8095791	0.9840424	0.7754018	4.4124361	0.9294123	0.9602646	0.7472871	0.879758	1.5000372
0.8199951	0.7239517	0.0610158	2.3436068	0.7283995	0.075738	0.7512066	0.4435006	1.6692989
0.7646859	0	0	1.8903299	0.4005379	0.0918014	0.2220626	0	2.1224408
0.7307486	0	1.2657969	1.2438518	0	0	0	0.2040155	0
0.4821255	0.7612853	0.0274372	3.5074268	0.494262	0.067501	0.3825013	0.2697513	3.6170869
0.9755568	0.5016175	0.1419559	2.1845025	0	0.1190241	0.25181	0.1673579	3.4742413
0.6774406	0.4514353	0.1126334	1.7811079	0	0.0713508	0.1742786	0.3122005	3.0400157
0.7005285	0	0.3127813	1.4935449	0	0.2028878	0	0.1003049	0.4871776
0.6396026	0.6508564	0.0535284	1.8451889	0.8515991	0.1910572	1.2476845	0.6226493	3.878176
0.6771699	0.4156505	0.1027253	3.3910803	0.1534162	0.1867541	0.1592417	0.2862898	1.6580515
1.3829807	1.7468144	0.5994448	5.1089022	0.8697398	0.4695387	0.8706343	0.587237	1.5472359
1.1175621	0.9681276	0.2608687	2.4375474	0	0.1996252	0.7563402	0.4617381	3.0280387
0.8101552	0.2868811	0	1.6728326	0.2888901	0.24123	0	0	0.904195
1.0962619	0.2766753	0.2330291	1.4869717	0.5121763	0	0.255682	0.1700532	3.5829413
0.5515898	0.5252671	0.3135941	1.6233987	0.1027253	0.2035144	0.0543623	0.3660284	0.1749177
0.3713916	0	0.0471195	1.2756627	0	0.0585243	0.1091599	0.1095612	0.2340107
0.8562288	0.537644	0.3406192	0.1923204	0	0.169412	0	0.187261	0.0623981
0.7567671	0.7016821	0.0729976	2.9406178	0.2113861	0.0905825	0	0.1680001	3.1131837
0.3958306	0.2157407	0.959696	0.9387751	0	0.0931545	0.1170301	0.531369	0.0989585
0.5955991	0.1584663	0.7918557	1.3130717	0	0.0676387	0.0432045	0.1264443	0.2683146
1.2661569	0.3215818	0.1688987	1.6459783	0	0	0.1774714	0.1355349	2.3723114
0.3092926	0.0855606	0.057277	0.6849085	0.3175356	0.0360461	0.3289513	0	1.9448584
0.8555911	0.6840108	0.0518593	1.3156822	0.2895984	0.064331	0.0207689	0	0.1013811
1.0787464	0.4990677	3.6373204	2.1777265	0.8742855	0	0.6741894	0.4932374	0.7889362
0.133695	0.4120769	0.0919367	1.6170633	0.1805298	0.439038	0	0	0.0816123
0.4019581	0.6442255	0.1691554	2.4681662	0.5585615	0.7311832	1.2489598	0.1518588	2.8303567
0.2486563	0.6568167	0.2524157	0.9340258	0.3314759	0.0377336	0.1818023	0.1389455	2.3234277
0.0731347	0.0747794	0.0251706	0.7485471	0.0753273	0	0	0.0299829	0.1596292
1.2437909	0.8120464	0.0705267	1.6044035	0.2045164	0.0444641	0.2606279	0.4724878	0.3778456
0.7414023	0.4256741	0.2038902	0.9823639	0.2993619	0.3607576	0	0.063641	1.2387869

0.5005981	1.0633649	0.7506922	2.3099038	0.5735684	0.0705267	2.0612232	0.3089433	3.9392924
0.415326	0.4746697	0.0458623	2.0162824	0.6681191	0.2156165	0.8613996	0.2062681	1.051024
1.1920047	0.5630605	0.1618232	3.4380266	0.6793337	0	0.1700532	0.5566011	3.225568
0.3863695	0.3937457	0.0731347	1.4771074	0.2117598	0.1340895	0.0581086	0.0439044	0.6021718
0.9019578	0.4034859	0	2.1506896	0.2173548	0	0.0597706	0.0890913	2.4151367
0.8057877	0.5446832	0.0713508	1.3340533	0.6927843	0	0.4010843	0.1642714	1.230326
0.4604805	0.2062681	0.0717627	1.9409184	0.3893465	0.0889557	0.1116991	0.3132459	2.6345236
1.2724413	0.520045	0.1633699	1.5623279	0.3691593	0.3441467	0.4029404	0.1184926	0.8448677
1.1058792	0.9131096	0.0461418	1.4187297	0.3748447	0.0574156	0.5488312	0.054779	1.961364
0.2713061	0	0.0977456	2.5002922	0	0.0617071	0	0.115566	0.9836047
1.2766158	0.6358008	0.3510629	1.9088513	0.3551291	0.0803848	0.4077334	0.0766959	3.5292463
0.8082615	0.2860531	0.0344975	1.7162008	0.1982427	0.0429244	0.3857071	0.0809305	1.7438165
0.6540689	0.1086248	0.0368901	2.187356	0.1094274	0.1335635	0.8795489	0.4212637	2.0821576
0.1895399	0	0.067088	0.5941663	0.3663642	0	0.3792883	0.1547128	1.5332643
0.4945692	0.8763303	0.0862403	0.8414096	0.8062828	0.0543623	0.2856981	0.3292958	2.2758116
0.9992063	0.9470302	0.1674864	1.5090877	0.2473199	0.2061431	0.2564069	0.6650289	2.4577256
0.5051796	0.5985558	0.0404015	2.1409095	0.4281424	0.3996633	0.2655569	0.1393385	1.8341044
0.7855087	0.4099068	0	1.9706328	0.2210728	0.422233	0.5803385	0.2950177	2.7080327
1.2893623	0	0	1.998701	0	0.3353689	0	0.1152996	0.8760159
0.1499097	0.153027	0	1.2820246	0.2934882	0	0.0416635	0	2.0371713
0.3696061	0.2913678	0	1.430874	0.104605	0.1680001	0.0278618	0.1220763	1.5864045
1.5467587	0.5348585	0.1765781	1.5428523	0.1545832	0.0654344	0.5563068	0.4135941	0.2895984
0.1745343	0.3366262	0	0.951364	0	0	0.3510629	0.1423481	1.6772151
0.7083197	0.2164859	0	2.3626382	0	0	0.1175621	0.0893626	0.6511321
0.5082258	0.4539653	0.2184712	2.3305871	0.3201958	0.1401242	0.4726957	0.0684645	0.7236022
1.7327468	0.2419622	0.2408638	2.8796862	0.4522791	0.2497487	0.516822	0.2389091	0.4022856
1.44297	0.623305	0.1237986	1.4137024	0.3474389	0.2005044	0.7113187	0.0990932	1.9614752
0.7814856	0.1131671	0.3145225	1.3918227	0.1141005	0.2662768	0.7704485	0.1330374	1.4269624
0.8012414	1.1697968	0.042084	1.8228117	0.3447149	0.0522768	0.4087119	0.0499095	1.1237324
0.5008021	0.2128806	0	1.9213982	0.2144979	0	0.5850587	0.2231754	0.1890338
1.1117658	0.1885275	0	2.0614652	0	0.1576905	0.3702759	0.0773797	0.5768607
0.6594679	0.2255217	0	1.139011	0.7501777	0.1890338	1.6856262	0.1807844	1.9374574
0.5595408	0.1216786	0	1.2485956	0.529671	0.0515809	0.1273691	0.0492125	0.1076879
0.8531973	0.3683772	0.7443333	1.8552323	0.5739561	0.1112985	0.5179307	0.582267	0.2790552
1.0724489	0.6447792	0	3.2135495	0	0.3905576	0.1355349	0.1031283	0.1146335
1.23842	0.8912636	0.8878372	3.782052	0.516822	0.4369081	0	0.116764	2.9052739
0.6650289	0.78073	0.2125071	2.9641029	0.3117356	0.2607483	0.7834986	0.3056787	1.6599702
0.539134	0.3386811	0.2332745	1.0896337	0.3410749	0.2856981	0.0966667	0.2734562	0.5517867
0.5130864	0.3296403	0.0483757	1.4214253	0	0.0600474	0.3740657	0.1392075	0.8409264
0.9417824	0.7264825	0.1835818	3.2464385	0.4981487	0.2256451	0.3787336	0.0458623	0.6892097
0.8681336	0.3573831	0.0332292	2.5346095	0.2779847	0.0413832	0.2439128	0.3529845	2.1385196
0.7765252	0.1066163	0.0716254	0.9862289	0	0	0	0.4785059	0.1828194
0.1765781	0.3402774	0.0418037	2.1790651	0.2371966	0.0519985	0.1585956	0.0976108	1.7740807

1.932288	0.2532632	0.1997509	1.9999279	0.4030495	0.2126316	0.4170559	0.7054914	0.4794375
0.3278023	0.0732718	0.23008	1.1762589	0	0	0.0196308	0.1678717	0.0961269
0.7063758	0.3639	0.1924467	1.7850902	0	0.0832474	0.4603756	0.2606279	0.0448837
0.6830227	0.7621362	0.1740228	2.598389	0.3031086	0	0.5319678	0.3149865	0.0950467
0.7782086	0.3144065	0.1821838	2.2652568	0.2185952	0.1385524	0.0600474	0.0894982	0.3627785
0.7015934	0.5125809	0.066537	1.8590154	0.3636757	0	0.2464689	0.0787464	1.7021699
1.445091	0.587845	0.115566	1.4788165	2.0149622	0.1428708	0.8928184	0.5811102	4.9441041
0.5329652	0.3833863	1.1667797	2.1574965	0.1405169	0.1163648	0	0.3105728	0.3425547
1.344374	0.4784024	0.1202192	1.2041407	0	0.2174789	0.0958569	0.3317052	0.9861561
0.9884847	0.587653	0	2.7478385	0.5914879	0.4662356	0.4576468	0.0923428	1.4703546
0.5157125	0.7922723	0	0.6996405	0	0	0	0.4291071	0.1749177
0.8856525	1.0136409	0	0.7859271	0.1410403	0.9149472	0.2471984	0.3117356	1.9017261
0.19131	0.3670355	0.2241638	1.4593792	0	0.0839282	0.2038902	0.2970153	2.9795132
0.2358496	0.2841587	0	2.1047057	0.1969847	0.1244605	0.2279875	0.5721136	0.0893626
0.2885358	0.7472585	0	2.0754984	0	0.1895399	0.0831112	0.1232689	1.8436221
0.2636354	0.1406478	0	2.6419852	0	0.1174291	0.1471764	0	1.3152184
0.3972553	0.1479579	0.0507455	1.7888945	0	0	0.3595212	0.635708	1.6283537
0.8081791	0	0	2.2682547	0.4716557	0.2126316	0.5385382	0.2033891	2.789145
0.4017397	0.219215	0.5750218	0.7651952	0	0.2674759	0.0607392	0.7200157	0.100574
0.2734562	0.1918153	0.1909309	1.4951323	0	0	0.0528332	0.2244108	1.0455828
1.044604	0.6444101	0.3411888	1.5004452	0.2168584	0.0930192	0.7438165	0	2.7292265
0.8325255	0.3401634	0.0622599	0.6480052	0.1814206	0.6324544	0.049352	0.2747686	0.4342412
0.9672424	0.6786128	0.8452692	1.0475384	0	0.169027	0.3057955	0.1615653	1.4428639
0.3304437	0.369941	0.6612936	1.1674222	0.0825664	0.1011122	0.0219061	0.403595	0.0366088
1.5965536	1.3088851	0.1770886	2.2140626	0.4824353	0.4508022	0.3892364	0	1.6031694
0.0622599	0.1245928	0.0425043	0.4693651	0.1255189	0.0528332	0.130272	0.4006472	0.3082444
1.3632272	0.6331987	0.3494785	2.2973088	0.1277653	0.1056781	0	0.1952216	0.5705601
0.6766285	0	0.0873271	1.5220557	0.2504766	0	0.1677433	0.1032626	1.2429378
1.3145805	0	0.3561438	3.3729382	0.888227	0.27453	0.2884176	0.0930192	3.1138672
1.1338923	0.5436938	0.0712135	2.7998126	0.3869213	0.1305356	0.3333092	0	3.2926934
2.0577622	2.0192393	0.3352546	5.0982785	1.7419631	0.6164048	0.2604473	0.8292813	2.403445
0.4549128	0.3248106	1.3261922	1.2843365	0	0.0735461	0	0.4838803	0.2225573
1.1141005	0.5058909	1.230449	1.8426973	0	0.2311866	0	0.1512094	0.3875831
0.3336527	0	0	1.8153295	0	0	0.4062101	0.3157981	0.3034593
0.4976379	0.2754839	0.0973411	2.6051387	0.2775087	0.5214528	0.0773797	0	0.4548076
0.857583	0	1.3832757	1.8237901	0	0.2765562	0	0.1384213	0.078883
0.4404207	0.4486896	0.0569996	1.5366	0	0	0	0.0676387	0.3422134
0.2963106	0.2464689	0.0439044	1.5129853	0.1294809	0.3447149	0.6203049	0.3718376	0.1674864
0.6035017	0.1697968	0.5415148	0.7739963	0.249506	0.2078929	0.2586998	0	0.2202061
0.0812033	0.082975	0	2.2331518	0.2375637	0.03492	0.4067543	0.5528689	3.624756
1.301295	0.5015156	0	2.2212894	0.1160986	0.1416944	0.5434959	0	0
0.880568	0.2977197	0.3402774	1.8503994	0	0.0668125	0.2018847	0.1248575	1.7532622
1.0522768	0.1969847	0.0683269	3.1199703	0.1984942	0.0847446	0.4277134	0.230326	1.9388503

0.2083924	0	0.0978804	1.6353365	0.1462642	0.0618453	0	0.369941	1.3170146
0.3350258	0	0.2352369	1.596649	0.7805621	0	0.2468337	0.0978804	2.7319435
0.5369481	0	0	1.6300792	0	0.1060803	0.3120843	0.3682655	0.9202171
1.0204844	1.0363978	0	1.926379	0.1793837	0	0.0487942	0.2085173	0.4904674
0.3017047	0.4649819	0.0447438	1.3903375	0.4681141	0.1609202	0.0701145	0	0.4166236
0.3917677	0.2779847	0	2.5441886	0.1467855	0	0	0.0592168	1.1481532
0.9173936	0.2651968	0	2.812108	0.6872399	0.1156992	0.3384529	0.4833644	0.2359721
0.9746761	0.7290089	0.0765591	2.0133551	0.5820742	0.1839628	0.175301	0.1759397	0.8447874
0.2539893	0.2247812	0	2.4094724	0.0794293	0.7029235	0.2704691	0.0625362	0.531369
0.7358687	0	0.2549568	2.3623016	0	0.2151194	0	0	0.2278643
0.7194901	0.249506	0.3463045	1.5693453	0.3261922	0.1430015	0.1360601	0.2311866	0.2218152
1.1210154	0.5658896	0.213254	1.8317557	0.1647863	0.1367164	0.359746	0.0668125	2.0787123
1.7214164	0.6500292	0.1910572	2.8211183	0	0.0825664	0.3345683	0.153546	1.4940059
0.8797842	0.2286032	0	2.2940178	0.230203	0	0.0635029	0.2668765	1.093425
0.776862	0.4184598	0	1.9520353	0.1544536	0.1880211	0.7947693	0.1223414	2.7500705
0.3700527	0.4238471	0.2263852	1.1462642	0.3312466	0.2773897	0.417488	0.3819478	0.9275931
0.6482814	0.1952216	0	1.7073923	0.6637545	0	0.2040155	0	1.6641187
1.3607014	0.6788832	0.0927487	2.8028756	0.1386834	0.169027	0.275007	0.0558898	0.5269702
0.9515132	0.4473147	0.1248575	2.7953098	0.1264443	0.3795101	0.3357119	0.281075	3.0844045
1.5774893	0.5128842	0	1.4160831	0.5163178	0.2878269	0	0.3249258	1.7234712
1.2459215	0.1807844	0.0315364	2.0463164	0.4183519	0.1512094	0.2538683	0.1096949	0.639973
0.5324666	0.8253786	0.0332292	0.2624332	0.2776277	0.3678183	0.1979912	0.1150332	0.2072682
0.9284272	0.422233	0.0900404	1.2090166	0.574925	0.0287106	0.4262111	0.0806577	3.3559184
1.3295829	0.6240541	0.0364682	2.6746188	0.9524081	0.533962	0.2661569	0.4499575	0.4226636
0.4693651	0	0	1.0819531	0.3970362	0.1764504	0.4108838	0.3196179	2.6136259
0.8917302	0	0.0970714	2.0975771	0.5088343	0.2309408	0.4310345	0.3199647	1.395995
0.5455731	0.7015047	1.3836628	1.6892544	0.2113861	0.175812	0.0579701	0.1680001	0.4271771
1.3423272	0.4774701	0	3.1981012	0.178619	0.4056657	1.1594354	0.072586	2.1736711
0.444455	0.3170725	0.0576929	1.4576468	0	0.0716254	0	0.0683269	0.2169825
1.8371494	1.632718	0.6257692	4.8694964	2.190362	0.6964984	2.1756098	0.6529683	3.0622311
0.7366482	0.3693827	0.090176	2.5456225	1.0554733	0.1645289	0.6886727	0.1571731	3.3270262
0.1607911	0.5675454	0	3.2574934	0	0	0.3959402	0.1310626	1.0433445
0.2744108	0.2147465	0	0.6157929	0.075738	0.1514692	0.1892869	0.2509616	1.8633042
0.7006173	0.2418402	0	1.1998137	0.2436691	0.1050075	0.1318528	0	0
1.9852089	0.3070787	0.1091599	2.012068	0.5636463	0.1981169	0.8293625	0.458592	3.3264511
1.5340118	0.9999279	0.0585243	1.5627187	0.8121285	0.2081426	0.7269184	0.1356662	2.1232027
0.2249046	0.4273916	0.1059462	2.944315	0.3006509	0.067088	0.0843364	0.2947825	0.4903648
0.1982427	0	0.2013829	2.4577256	0.9338747	0.0871913	0.5973166	0.3750672	3.1080393
0.8663146	0.9237957	0.2000021	1.827819	0.1544536	0.0654344	0.5845777	0.3891262	3.3409752
0.556503	0	0.1457427	2.215927	0.2814312	0.4370146	0.0786098	0.0596322	1.6832473
0.5310695	0.3331947	0.0903115	1.2539288	0.2586998	0.1118326	0.3475523	0.3582837	0.848638
1.3780121	1.3972553	0.4061012	1.7322258	0	0.0943712	0.3781787	0	0.7819052
0.5109619	0.4301782	0.4887207	1.3110962	0.9071983	0.3645724	1.3231971	0	4.0860449

0.757706	0.4889263	0.757962	2.4569901	0	0.0395596	0	0.0745054	0.1991226
2.319194	1.7010019	0.6052731	5.4236039	1.548601	0.8445731	1.3959768	0.8260298	1.6945682
0.555718	0.1737671	0	1.1263121	0.3312466	0.0745054	0.4354152	0.1054099	0.905967
1.4338675	0.7977186	1.2530816	5.5703202	1.3743718	0.0603933	0.5407217	0.8061591	2.0562887
0.4692608	0.754802	0.0462815	2.1665226	0.2605075	0.2173548	0.3005338	0.3894567	2.4355753
0.6927843	0.1243281	0.4729036	2.1547128	0	0	0.1300083	0.3200803	1.7724347
1.0500489	0.6630258	0.0681893	2.038998	0.1978654	0	0.1566555	0	2.2203919
0.1509495	0	0.1040682	0.7144873	0	0	0.1611783	0.1228715	0.3760681
0.3568199	0.4265331	0.1558787	1.8511594	0.3000651	0.1920679	0.0427844	0.063779	0.9703383
0.0926134	0	0	1.2311866	0	0.0787464	0	0	0.503247
0.3621051	0.2561653	0	2.0621908	0	0	0.4930324	0.2529	0.1741507
1.3766238	0.1401242	0.0943712	1.8514792	0	0.116897	0.2144979	0.1644001	0.1823109
0.778545	0.2995963	0.0363275	1.4593267	0.4727997	0.0451634	0.1647863	0.0432045	1.8513593
0.3408471	0.3474389	0.063779	1.6456094	0.185486	0.0790196	0.1466552	0.0754642	1.9989898
1.2770921	0.1959775	0.067914	1.7111425	0	0.0843364	0.1059462	0.0805212	0.2535053
1.7568525	0.3450557	0.1237986	2.3126652	0.5398288	0.1528973	0.5807244	0	1.3257318
0.7096435	0.6383056	0.0861044	1.4403144	0.2473199	0.1067503	0	0.3289513	2.0078055
0.9711481	0.3166092	0	1.4560702	0.1681285	0.2667566	0.0455828	0.0681893	0.1479579
0.5361524	0.2295879	0.0272956	2.4791271	0.300768	0.0339339	0.1047392	0.0640551	1.9199121
1.5587085	1.4893888	0.1108977	2.9410875	0	0	0.2115107	0.2510828	3.1373396
0.4966157	0.5056877	0	1.4803685	0.884637	0.4760187	1.0127117	0.2210728	2.4668881
1.3549035	0.5751187	0.2503553	2.8610422	0.2198345	0.1828194	1.0641931	0.0457225	2.3798704
0.41792	0.1856129	0.0641931	2.4567799	0.3526455	0	0.1003049	0.0761486	0.2404975
1.730966	0.3961595	0.0494914	1.6304055	0.1456122	0	0.0392788	0.0588014	1.4414304
0.60862	0.5729867	0.0458623	1.9152532	0.4782988	0.4469972	0.4138107	0.2065182	0.5153088
0.9981955	0.2677156	0	2.23091	0.2067682	0.1718473	0.0932897	0.0287106	2.267356
0.1620811	0	0	1.3200803	0.1131671	0.2235461	0	0.3646845	1.3411888
0.2590615	1.0742999	0.0931545	2.0996319	0.2661569	0.0588014	0.3962691	0.2123826	1.8699898
1.3390233	1.0301242	0	2.8911275	0.2327835	0.587941	0.4479494	0.1852323	1.3817264
0.3774014	0	0.0713508	1.3334237	0.3873625	0	0.0565835	0	0
0.8820561	0.1318528	0.2894804	2.1816751	0	0.2606279	0	0.249506	0.5073126
1.61664	0.8302553	0	2.3525043	0.2916036	0.1269729	0.2677156	0.3369689	2.4726177
1.1044709	0.0938306	0.1523781	1.2242873	0	0.0395596	0	0.482745	0.3403914
1.3372544	0.1110313	0.0746424	1.9421203	0.1118326	0.3006509	0.347779	0.3248106	0.4723838
0.385928	0.5559143	0.7886857	0.5315686	0	0.2565276	0.2194628	0.086512	0.4997821
0.6829328	0.419431	0	2.7214602	0.154972	0	0.6140973	0.0626744	0.801407
0.4325319	0.2370742	0	1.3353117	0	0	0.0659858	1.0890913	1.1240634
0.7441611	0.1011122	0	1.514703	0	0.2018847	0	0	0.045443
0.7729836	0.7385517	0.0522768	3.0777557	0.2923106	0.1868808	0.081885	0.2336427	2.7630291
0.5574836	1.1631122	0.6723801	1.4655044	0	0	0.0511633	0.1487389	0.1651723
1.102255	0.3576083	0	2.1147668	0.130272	0.2558028	0.6001746	0.5298709	2.1593386
0.6014113	0.2341334	0.2800061	2.2747388	0	0.1015156	0.4855299	0.6160753	0.3317052
0.5199444	0.7567671	0.0912598	1.9262272	0.1365852	0.3156822	0.445197	0.1591125	1.7028791

1.082362	0.343351	1.4528063	1.5407217	0	0.1521185	1.064469	0.5098478	2.3450841
0.2672361	0	0	0.6699338	0	0	0	0	0
0.083792	0	1.0088454	1.3572705	0.1674864	0.2655569	0	0.1326426	0.0756011
0.9342523	0.3897871	0.0486547	1.6802343	0	0.1742786	0.3764015	0.4511188	0.5857317
0.4692608	0.3941848	0.0492125	1.2263852	0.2121335	0.1199537	0.0582472	0.0583858	0.6354294
0.7646859	0.8429386	0.1212807	1.7682479	0.2789363	0.2856981	0.0588014	0.2734562	0.1285574
0.8661563	0.6597419	0	1.6136259	0.3031086	0.2820246	0.1056781	0.1263121	0.3280322
0.5962673	0	0	1.8485578	0.3374257	0	0.4968202	0.072586	2.2626737
0.8159852	0	0.4592218	1.3821139	0	0.0820213	0.7368213	0.1525079	0.804384
0.7771146	0.353662	0.0650208	1.1910572	0.1890338	0.4274989	0.1729995	0.1500397	1.1525079
0.2988929	0.1604039	0.4130524	1.0020904	0.0831112	0	0.1478277	0.4307135	0.3021728
0.0866479	0.5905302	0	0.92486	0.5942619	0.0735461	0.971516	0.0702519	0.4157587
0.913033	1.0585243	0.6216182	2.6749803	0	1.0324535	0.0666748	0.4392508	0.3981314
1.2844549	0.8201585	0.0597706	3.0450585	0.8250529	0.2764371	1.0727232	0.2028878	3.4444418
0.5192399	0.3963787	0.5052812	0.344374	0.1731274	0.1090262	0	0.2624332	0.4456208
0.7959331	1.3245802	0.2340107	2.3996633	0.3420996	0.2865263	1.229711	0.4319973	5.2646903
0.4915966	0.4240621	0.046002	1.1691554	0.5772475	0.1388145	0.8112244	0.0275787	0.5585615
0.9078136	1.0052563	0.1997509	2.7835614	0.1541943	0.1277653	0.1973622	0.1793837	1.3655246
0.5492256	0.2901885	0.4141355	1.8530375	0	0.0965317	0	0.260387	0.0690147
1.0401209	0.3650206	0	1.0163537	1.3761237	0.162339	0.8109777	0.1552311	3.3754565
1.1296787	0.7894371	0.0754642	1.5061957	0.3157981	0.2234226	0.1728715	0.1319845	1.6333382
1.1024566	0.7131679	0.0985543	1.4944156	0.5156115	0.3386811	0	0.1164978	0
1.4096896	0.5091384	1.2078304	2.4884636	0.1918153	0.43328	0	0.1522483	0.1688987
1.1342867	0.1344838	0.046002	2.204798	0.1355349	0.1651723	0	0	0.516116
1.2663368	0.5169229	0.1926992	1.5044679	0.7855924	0.1620811	1.0902437	0.0795659	4.8957728
0.879941	1.021764	0.0538064	3.0570516	0.1575612	0.191689	0.5656947	0.063779	2.594668
0.3148705	0.2218152	0.0773797	1.6441793	0.7397617	0.6323613	0.4770556	0.091666	1.3837734

NFYC-AS1	AL121845.4	SH3BP5-AS	AC004908.3	AC018529.1	AC011825.2	AL353801.3	AC012020.1	AC106845.1
2.8095379	2.1745662	3.0979984	2.0688771	0.2783417	1.4151637	1.093425	0.3654686	0.0394192
1.1744064	1.6224619	2.0867838	1.431088	0.2579761	0.3283769	0.605684	0.2036397	0.0540844
1.3413027	1.6846841	1.4765372	1.0595629	0.2273714	1.2917803	0.1553607	0.556503	0.1662013
1.5620348	0.7608596	2.2114172	0.2265085	0.3722835	0.6567252	0.4772628	2.1481206	0.5071096
2.3344539	0.8067777	2.5363762	0.8682917	0	0.5003942	0.8844807	0.5085301	1.3847682
3.1193728	1.8563085	3.8497791	2.1113986	0.1772162	1.0169954	0.9689386	1.2228664	0.5365503
1.9005287	0.9431337	1.6733755	2.4639624	0	0.5950262	1.1476323	0.2033891	0.4926223
0.3279173	0	0.6082415	0.3315906	0.533364	0.133958	0	0.0611541	0.3465315
1.1714631	0.8453495	2.2538078	1.6827081	0.2928995	0.6670292	0.2015084	0.6869711	2.5439412
1.4569113	0.9066597	3.0062262	1.878333	1.3119681	1.4297499	0.1680001	0.8608436	0.6697525
1.2909551	1.4543865	1.9139904	1.4352019	0.3171883	0.2147465	0.3168409	0.5731806	0.6632991
0.5574836	0	0.5158134	0.4475263	0	0.8656813	0.2746493	0.1059462	0.4310345
0.5786006	0.662479	1.9458699	1.8731439	0.8666311	0.1473067	1.1417597	0.1933302	0.3664761
1.2174169	0.803723	1.8979761	0.8503994	0.4638317	0.22033	0.4186757	0.1265765	1.5232608
2.0574849	1.9253539	2.4896971	1.2449479	0.4487953	0.8748363	0.9798059	0.8364897	0.2070182
0.8778228	1.7263081	1.5553744	0.5098478	0.7940207	1.1875144	0.217727	0.033793	0.1523781
1.2761989	1.5655972	1.7597524	1.3120843	0	0.5782142	0.3805078	0.6574571	0.4679055
1.0871233	1.1248575	1.3578898	1.0084152	1.1706942	0.5754092	1	0.2986583	0.3338817
1.7184822	1.5303704	2.0824642	1.4200242	0.1376347	0.6670292	0.9001809	0.5217543	0.1171631
1.494518	1.2564672	2.0071237	0.706199	0.2242873	0.3499314	0.1531568	0.1183597	0.1138338
1.2761393	1.4552286	1.9075445	1.4031586	0.6110779	0.6330126	0.6433945	0.2182232	0.4690524
1.7887692	1.0072673	1.7995018	0.9185389	0.4249221	1.227433	0.6510402	0.1367164	0.1231364
0.7363884	0.8373782	1.8110599	1.1080895	0.5306701	0.3675947	0.5302705	0.1333005	0.1764504
2.0032424	1.0744369	3.587713	1.1928254	0.9541218	0.7557422	1.0104934	0.5839041	1.3703875
1.1842803	0.9299425	1.9409184	0.8283066	0	0.9080443	0.328262	0.6386763	0.8764875
0.5722106	0.2236697	0.899717	0.8648894	0.3609822	0.1280294	0	0.2869994	0.4540706
1.9846623	1.6447331	4.1178363	4.9254393	3.1719434	1.8009517	0.4857359	1.6868367	1.4435536
2.383884	0.5377433	2.2746493	2.1100959	0.6547105	1.2477453	0.1731274	0.4836739	0.4966157
1.0765591	1.7015934	1.7247812	0.8381854	0	0.4394636	0.4472089	0.4113178	0.0253123
2.6304637	2.673726	3.2749102	4.9232229	2.2807337	4.0401823	2.6792999	3.549971	3.988412
0.9929859	1.4701984	1.5216538	0.7226409	0.1700532	0.5752155	0.5023306	0.2843957	0.2174789
0.7025689	0	0.5810137	0.2487777	0.7238643	0	0	0.0666748	0.0889557
1.1089593	0.6047356	1.1743425	0.7113187	0.7329204	0.3652446	0.2874723	0.2918393	0.1191569
2.3489122	2.1285904	3.1076042	2.445197	1.0701145	1.3669236	1.2130673	1.3098165	0.7043408
1.5729867	0.5260688	1.1109645	0.4615286	0.2467121	0.6302191	0.3200803	0.2558028	0.2848694
1.1146335	0.1593708	0.9332705	0.5434959	0	0.255682	0	0.0871913	0.063641
2.2871472	0.7766936	1.0777215	0.6076736	0	1.5720165	0.4881036	0.1214133	0.1352722
0.7932717	0.5350576	0.6775308	0.2866446	0	0	0.325962	0.0775165	0.1683853
1.5960764	0	1.6678467	1.3704433	0.6870607	0.4835707	0.4919044	0.2885358	0.826762
2.0949116	1.6584629	3.4603494	2.2460736	0.9979788	0.1486088	0.5288713	1.0037461	1.0943036
1.2310022	1.2144357	2.3686007	1.0918014	0.5055861	0.6304055	0.5518851	0.585924	0.7031007

1.430981	0.380397	1.9731704	0.5291713	0.3284918	1.2236697	1.1323794	0.5314688	0.158854
1.7368646	0	2.2329984	1.4773147	0.4472089	0.6718368	0.4468914	0.5605193	0.6482814
2.2846918	0.6643918	2.675274	2.4433149	0.5809173	1.7073923	1.3138843	0.5654022	0.8911081
0.9793669	0.1526377	1.6115029	1.0272248	0.2499914	0.1680001	0.3242346	0.313362	0.1674864
2.0552651	0.9565028	1.9275552	1.3199647	0.2621926	0.7229905	0.6144743	0.640991	0.5653047
1.4137565	1.102255	1.6445024	0.8062828	0	0.2767944	1.447156	0.6164518	0.116764
1.1044038	0.5086315	2.0324535	2.1757801	0.8766446	0.8935175	0.7105254	0.3201958	0.4572265
2.1158989	1.6497074	3.1483159	1.8545942	2.765174	1.5901948	0.6811344	0.954643	1.0265876
0.1663298	0.153546	1.1662013	0.632082	0.4653999	0.169027	0.1722315	0.0839282	0.5005981
2.6288202	1.6845944	1.9643247	0.8387501	0	1.2245343	0.7500919	0.2806	0.0316775
1.9880484	1.7960162	2.6606777	2.3231395	0	1.3666439	0.7174735	0.4340277	0.5369481
1.9999639	1.4792306	2.4685312	1.2764967	0.250234	1.3127233	0.9132628	0.0261627	0.0176369
2.3841051	1.7545027	2.8247068	2.0890235	0	0.8385081	0	1.6517291	0.9184626
3.4304815	2.7938959	3.2704093	2.6292944	2.0769467	3.1872135	2.4816864	2.2344911	2.8114984
2.2371048	0.8632249	2.9686253	1.6502591	0.3002995	0.9309265	0.5484366	0.6620232	0.5672534
1.2913089	0	1.9851361	0.217603	0	0.7957669	0.2481705	0.6657566	0.2428159
0.7921057	0	1.2521128	0	0	0.5324666	0	0.1365852	0.3156822
1.3451125	0.978635	2.8398991	1.0490731	0.4971269	0.9051198	0.4249221	0.6110779	1.6009833
1.6106056	1.8531973	2.8306001	2.1412693	0.4532278	1.2174789	0.797096	1.2908372	0.2092662
1.5854433	0	2.7185699	2.7608384	1.061638	0.6487415	1.2038902	0.4186757	0.6598332
1.3757345	1.102255	1.6523259	0.6429326	0.4050121	0.509037	0	0.5093411	0.1986199
2.3671753	1.8589358	3.7318566	2.6467387	0	1.6390932	0.7435581	1.2984237	0.2600257
2.432425	1.5185855	2.8775087	1.8119642	0.8403625	1.7320955	1.6949248	0.8117998	0.9452331
2.1848939	1.5326993	2.965735	2.4294106	1.464738	3.5957304	1.9946521	1.7576065	2.0614076
1.9991702	1.8090028	3.311009	1.2317396	0.8867452	1.2543522	1.4310345	0.4646683	0.3041605
1.0610849	0.942233	1.944821	0.9278206	2.0442542	0	0.3335382	0.154972	0.3839393
1.2904244	1.5815923	1.8524384	1.5614483	1.0882773	0.3162616	0.3219281	0.229711	0.3976935
1.3231395	1.3411318	2.1634987	1.7666376	0	0.4215869	0.6834719	0.2184712	0.082975
0.8154935	0.7034551	1.3488556	0.2691529	0	0.9465063	0.3062622	0.1638851	0.7195777
0.6700245	0.7956007	1.8797842	1.795102	0.2365845	0.3018217	0.2363395	0.2135028	0.0574156
1.7571086	0.4093638	2.5036286	1.6266728	0.6379348	0.787516	0.7997505	0.7069946	0.7234275
0.6895675	0.6444101	1.4349884	1.250052	0.1315895	0	0.4662356	0.1151665	0.0528332
0.3881344	0.3139424	0.92448	0.6737373	0.4979444	0.8537562	0	0.2381754	0.1984942
1.1283594	2.2208871	2.8854572	1.9325526	0.2888901	1.1095612	0.7976771	1.1936456	0.3163775
1.0639171	0.7134319	1.3814495	0.3930867	0	0.359746	0.5903385	0.3241193	0.5788904
0.8617172	1.1078218	1.7078781	0.886277	0	0.9221217	0.1768334	0.2036397	0.1891604
1.8124982	0.5189379	2.1593063	1.9088513	1.0709388	0.9694544	0.3154503	0.5991273	0.6750933
0.7031893	0.8727106	1.4136482	0.9630302	0.769264	0.3858175	0.5550308	0.2479275	0.1365852
2.9911182	3.1474044	3.6913443	2.0618108	0.9996393	1.5642806	0.6366362	0.7734056	0.4059924
0.6858056	1.2278027	2.1011122	1.1689629	0.423632	0.9193019	0.7500919	0.5153088	0.9776829
0.2409859	0	0.5320676	0.2846326	0.6474527	0	0	0.045443	0.1828194
2.2514161	2.0586628	3.585251	2.1408441	0	1.4137565	0.8515192	0.7104373	0.7714631
1.3832204	0.5649147	1.7470866	2.4172449	0.6865229	0.9473295	0.3455099	0.0959919	0.5742469

2.8410674	2.2645364	2.9028068	2.9460758	1.4436597	2.6307549	0.6533352	0.5334637	0.7530054
2.2885948	1.5039593	2.1149333	2.721132	0.23008	1.1647219	0.656176	0.8506394	0.1390765
2.224349	1.3219281	2.6180507	2.1059462	0.2772707	1.2529606	1.1828829	1.5497185	0.9540474
0.5913922	0.4099068	1.2116975	0.487795	0.6389543	0.241108	0.2454957	0.5105569	0.7323995
0.7437304	0	1.4002647	1.5782625	0.6536104	0.458487	0	0.2559237	0.3556929
1.5155106	1.8327685	2.2255217	0.7023916	1.06067	0.2353594	0.7851739	0.3365119	0.0253123
1.0769011	0.7171225	1.0626053	0.3952822	0	0.6176746	0.2409859	0.2606279	0.4938522
1.5342609	1.5703658	2.0361165	2.1171298	0.5980793	1.1202192	0.8236679	0.9125732	0.7599228
1.1329716	1.1591771	2.7198844	1.2068307	0.7629015	0.9334971	0.158337	0.7508637	0.5166203
1.2423281	0.2886539	2.3915203	1.2687937	0	0.3162616	0.5850587	0.0640551	0.1183597
2.1091599	1.2928995	4.497689	1.7102168	0.797096	1.8985566	0.5764738	1.3751785	1.6954594
1.3998273	1.1538053	2.2008496	1.3642362	0.1760674	0.5096452	0.8303365	0.593306	0.3450557
1.0898371	0.4086032	1.380009	1.3802862	0.9792205	0.6262989	1.2548963	0.1818023	0.2613503
0.25181	0	0.1378969	0.2877087	0.4667576	0.1151665	0	0.026446	0.1036655
0.9390761	1.4597464	0.8026482	0.7369079	1.8631455	0.2813125	1.1427402	0.2332745	0.0899049
1.2691529	0.473735	1.2636955	1.0989585	0.4108838	0.7183507	0.5249663	0.3817264	0.3302142
2.0784732	1.5726957	3.4458194	2.3591838	0	1.0653655	1.2125693	0.6343145	0.1619522
2.4334402	1.2490812	2.976272	0.9462817	0.3693827	1.2800655	0.6626613	0.3266524	0.743041
1.8966981	0.9132628	2.3962143	1.1999393	0	1.3022898	0.5837116	0.724301	0.068602
0.3759569	0	0.9935657	0	0.4828483	0	0.1793837	0.2420842	0.2711866
1.1122998	0.7003509	2.1755885	1.1530919	0	0.5183337	0.9784153	0.3727293	0.3896769
1.9631412	1.5839523	2.3220724	1.9761803	0.2620723	0.7784609	1.514905	0.7188767	0.8086734
0.5353563	0	1.1927623	1.0551957	0.3025238	0	0	0.2532632	1.0335816
1.2349304	1.2369518	1.3446581	1.2195248	0.3646845	1.61584	0	0.2567691	1.1957255
2.3434931	1.8409667	3.0505539	1.7118913	0.523863	1.4408459	1.4592218	0.9342523	0.650305
2.7118913	0.8198317	3.8375094	2.5534342	0.2168584	1.5903385	1.0565835	0.4108838	0.5706572
1.8206896	2.072929	2.4146497	1.2543522	0.2140004	1.0313952	0.7129039	0.6602898	0.5842891
1.260387	1.0184917	2.4096896	1.8459918	0.3672592	0.8130321	0.6591939	0.9609565	0.4316764
2.1570114	1.9971479	2.7170348	1.1901723	1.0769695	2.2716647	1.1119661	1.0144267	0.4001007
1.8311476	0.4148932	1.5903385	0.5210507	0.2488991	0.7969299	1.3246378	0.374177	0.5572875
1.9697491	1.6072474	2.7069283	1.6541147	0.3193867	0.5708515	1.4064278	0.417704	0.5374452
0.5360529	0	0.4956952	0.7604339	0	0	0.4856329	0.1587248	0.3039268
1.1401242	0.4529117	1.0124256	0.8579014	0.7005285	0.7775354	0.6045458	0.0640551	0.0289934
1.4689482	1.3921525	1.8670266	1.0661236	0.2292187	1.231801	1.3814495	0.4592218	0.1237986
2.7152564	1.124659	1.9064673	1.959696	0.4152178	1.3357691	0.7368213	0.1751733	0.2573728
2.5931864	1.4805753	2.9322313	3.1161984	0	1.4509605	0.325041	0.4894402	0.7367347
2.2552288	1.9097731	2.5042899	2.6781395	1.0443242	2.0185274	1.0923428	0.9830939	0.5283713
0.8514392	0.8729469	0.8491987	0.6254575	0.7695179	0.385928	0	0.0797024	0.064607
1.3005338	0.4034859	1.8184007	1.1100291	0.348799	0.7265697	0.5110632	0.2345013	0.2649567
2.1236662	0.8930515	2.9240999	2.0666403	0.7878503	0.8145914	1.3495917	1.4925198	0.6128714
2.0900404	1.5428523	3.0796512	1.486354	0.5840966	0.8596516	1.1133671	1.0015861	1.2751859
1.0553345	2.1809117	1.2892443	0.3056787	1.5392829	1.3284344	0.1252543	0.41792	0.1106305
0.7137838	0.245739	0.7249994	0.7886857	0	0.1411712	0.2746493	0.1461338	0.0720372

1.3397075	1.3067289	2.6539543	0.78375	0.2952528	1.5497185	0.6130601	0.4922121	0.1224739
0.5417131	0.6286336	1.5649634	0.3418719	0.1277653	0.0846086	0.2448871	0.1237986	0.0843364
2.4295624	1.8294437	3.0806918	1.1701815	0	0.7355222	1.6019817	0.3178829	0.2814312
1.7445056	1.2974849	2.1216454	0.9648052	0.4977401	0.5342609	0.5432979	0.6087146	0.0127832
1.0562367	0.748633	2.1997509	0.662479	0.8990209	0.730053	0.6562675	0.5254676	0.1897929
1.6104166	0.6742799	1.9791839	0.9069675	0	0.9921158	1.1225402	0.3709454	0.2791741
1.7608596	1.8615981	3.6797278	2.8969306	0.5326661	0.9065828	0.673466	1.708717	0.4923147
0.8246457	0.2781037	1.6188964	0.7895206	0.9266826	0.8666311	0	0.1662013	0.1598875
2.0135695	1.7953098	2.4258621	1.5270203	0.5512945	0	1.0600474	0.6953703	0.2033891
1.3917127	1.1562672	2.5311693	0.8949147	0.6737373	0.9792205	0.9199121	0.5180315	0.9480027
1.0007933	0	1.2743511	0.3787336	0	0.4214792	0	0.0533894	0.1393385
1.6985297	1.0477478	3.0702176	2.0839962	0	0.8693187	1.217665	0.6336637	0.3456235
1.0325946	1.7118032	1.1991855	0.6728326	0.4707191	0.5885168	0.7529198	0.2544731	0.0475384
1.4382396	0.9353089	1.1134338	0.9765103	0.5997939	0.5895718	0.3305584	0.2994791	0.012068
0.5157125	0.9617715	1.7399777	0.9470302	0	0.8372167	0	0.5993178	0.5301706
1.1418905	0.5152078	1.7396321	0.705226	0.241108	0.776104	0.4473147	0.1676148	0.0666748
1.2599654	0.7472585	1.8547138	0.7350022	0.2530211	1.0009374	0.3281471	0.2679552	0.1207501
1.6721538	0.4873834	2.5736896	1.831026	1.0157116	0.7373406	0.2950177	0.7015934	0.092884
0.458382	0.4262111	1.1023222	0	0	0.251204	0	0.1723595	0.1054099
0.4627853	1.3157401	1.2009437	0.1963552	0	0.2200823	0	0.2576141	0.3366262
2.4003193	1.4249758	3.2426635	2.8782937	0	0.8790784	1.3880793	0.6024569	0.0265876
1.4424393	0.3544522	1.321755	0.8625109	0.5579737	0.9447085	0.7039866	0.0163537	0.1067503
1.3122586	1.1751094	2.0623981	1.2545336	1.0471195	0.6060631	0.2358496	0.5629628	0.0968016
1.1515341	0.794603	0.4373343	0.5054845	0.500904	0.4209405	1.0745054	0.0568609	0.0289934
1.9705592	1.7091141	2.5180567	2.0815442	0.7651952	1.1737031	0.6621143	0.509037	0.1554902
0.4614238	0.8119642	0.6209617	0.6368218	0.2142492	0	0.1461338	0.054779	0.0443242
2.4688179	1.9580631	2.6371697	2.7129479	0.2179751	1.2532027	1.0607392	0.4128357	0.6237732
0.7166837	0.4793341	1.5066527	0.254594	0	0	0.1522483	0.3410749	0.2836848
1.640297	0.4369081	2.7265915	1.0279325	0.3782897	2.2384506	0.2626737	1.1961034	0.8319583
2.0156403	1.5448316	3.2290495	1.9024209	0.9621418	1.9035397	1.1196218	1.7733634	0.9776096
4.2867185	3.5833265	3.2159038	3.1853829	2.1914047	4.9934072	2.2007397	1.5618149	2.284603
1.1682569	0.338567	0.8292813	0.6021718	0.5344601	0.5251668	0	0.0309716	0.0617071
0.6154163	1.6102276	1.6863885	1.0036742	0.582267	0.4055568	0.5817851	0.4316764	0.1122331
0.4896457	1.0799754	0.5239633	0.3478924	0.2108875	0.1410403	0.9585829	0.010923	0.0578315
1.5651097	0.7322258	2.0144624	1.6591025	1.5201959	0.4498519	0.5827487	0.6668475	1.0492125
0.2558028	0.6180507	1.1618877	0.1774714	0	0.1991226	0.2028878	0.0769695	0.0827026
1.044534	1.3381677	1.8278597	0.8059528	1.0562367	0.5087329	0.3646845	0.449535	0.6432097
1.3746778	1.4628899	1.9147176	0.7388975	0.4124022	0.7207162	0	1.1844707	0.0903115
0.6059684	0.3352546	1.1929516	0.3290662	0	0.1950956	0.1027253	0.1116991	0.1575612
1.0717627	0.4562805	0.8699503	0.3823906	0.3953919	0.5658896	0.0976108	0.7099082	0.5971259
2.1820884	2.2195248	3.9257146	1.1884009	0.1986199	1.8869013	0.7555713	1.0703893	0.1448295
1.8178279	0.7825763	3.0032064	2.1402551	1.2776277	1.5158638	0.3460775	0.9671686	0.6004601
1.5855395	2.2257685	2.6470381	1.3222166	0.6036916	1.0125687	0.7588148	1.2295264	0.455965

1.0327356	0.9161709	1.1139671	0.4067543	0	0.8945268	0	0.3273425	0.0688771
1.3915478	2.2417182	1.2693923	0.9358369	2.1626934	0.4984551	0	0.2798873	0.4542812
1.872159	0.82546	2.0627089	0.8122928	0	0.8909525	0.7257848	0.7355222	1.9456077
1.168642	1.0710075	3.0400683	1.5951217	0	1.0468403	0.697329	0.2532632	0.105544
1.3109217	0.6757256	1.4431292	0.8318772	0.4196468	0.7322258	0.5357544	0.4167317	0.2733368
1.5744891	0.9186152	1.5709	1.2730384	0.4618428	0.3176514	0.587557	0.5869809	0.7755142
1.9108478	0	2.6476829	1.4090922	1.0531113	0.5536554	0.5629628	0.3726178	0.2179751
1.169412	1.4445081	2.0230069	1.4254056	0	0	0.2562861	0.7825763	0.8908747
2.0565835	0.9573203	3.0164785	1.0669502	0.6768992	0.7794699	0.7916057	0.2950177	1.0592168
1.5086822	0.8590949	1.3546214	0.9973285	0	0.9266067	1.5049254	0.3223897	0.2653169
1.4188916	0.3375398	2.2825584	1.3859832	0.5330649	0.6631169	0.7396753	0.4112093	0.5221562
2.2171066	1.0043216	3.3645304	2.2681948	0.2788174	0.1880211	1.0948441	0.7313571	0.2559237
2.0853907	1.9930221	2.7246939	1.9155974	0.590051	1.3128975	1.3302716	0.1820566	0.3367404
2.3159429	0.4431823	2.1967959	2.8405237	0.9369677	1.1335635	1.2757818	0.2708279	0.16221
1.9783421	1.8416914	3.0652104	1.1044709	0.261952	0.9335726	0.6140973	0.6581886	0.1422174
1.3727293	0.6196478	1.5330649	0.1211481	0.6838312	0.2605075	0.4894402	0.3630028	0.198997
0.7318783	0.3825013	0.7560839	1.028852	0.8253786	0.5887086	0.2282338	0.5588554	0.3799536
2.3316192	2.5423076	3.2053925	1.8760159	0.2360946	1.1116323	1.2757818	0.7629015	0.7799741
1.6980406	1.2220008	2.6867246	1.3169567	0.5695883	1.1789377	0.6204926	1.2803031	0.3119681
1.7096877	0.7404527	1.5703172	0.4096896	0.6493855	0.5799525	1.0070519	0.1505597	0.0352016
0.9358369	1.3436921	1.9791839	0.9696755	0.1615653	0.3011194	0.9614752	0.3765127	0.7926888
1.4785059	0.7949357	2.369271	1.5574346	0.698663	0.6502131	0.1150332	0.4708232	0.1015156
1.3918777	0.7759355	2.0088096	1.3752897	0.2295879	0.9687912	1.3511195	1.6201641	0.0939658
3.4306867	1.093425	3.1802274	1.8547138	0.1856129	1.1729355	1.5001902	0.2133784	0.8509594
0.6205864	0.729879	0.6248028	0	1.4192692	0.4482667	0.2459824	0.1123665	0.3056787
2.4708492	0.6653928	1.8793137	0.2811938	0.5817851	0.3140584	0.8694766	0.6787931	0.1856129
1.1693478	0.7275284	1.6053995	0.9732806	0.874915	1.0626053	0.2450088	0.5432979	0.3255015
1.4536493	1.2446435	2.7684808	0.955164	0.7626465	1.4951323	0.6947465	0.344374	0.1050075
0.6843699	0.8256228	2.0962619	1.093763	0.5224576	0.8911858	0.3681537	0.2121335	0.2784606
2.7355583	3.0400917	2.7853901	2.4803168	1.7464418	3.4619869	2.2643963	2.7941247	2.3056496
1.8722378	1.2787579	3.6052098	1.0685332	0.4280352	1.4874349	0.9413317	1.229834	0.7720546
1.6770346	1.3302716	2.059113	0.9918257	0	1.420132	0.891497	0.6017916	0.8313909
0.7051375	0.8356007	1.4675925	1.5731806	0.4646683	0	0.8150835	0.3357119	0.5494227
0.9272138	1.5419113	2.4881551	0.953079	0	1.0411729	0.2820246	0.1904252	0.6107001
1.287945	1.5787938	2.9933483	1.6731493	0.7063758	1.0701145	0.6424705	1.0775165	0.7873489
1.6924718	2.7570659	2.0515809	1.1902356	2.5564539	1.3750116	0.6702058	1.4369081	0.1576905
1.4576993	0	1.1857397	0.6683915	0	0.3407332	1.061638	0.1096949	0.4419084
1.3145805	0	1.2733965	2.1862787	0.3417581	0.9049657	0.9185389	0.1249898	1.3391944
2.3716982	1.525668	2.958453	2.1160986	0	0.7223786	0.8445463	1.101919	0.4046852
2.1008095	1.4808338	3.5955037	2.900606	1.7937294	1.1627901	0.9144114	0.7559131	1.0176369
0.5303704	0.626953	0.8762517	0.6777112	0	0	0.2062681	0.1812934	0.4751887
1.9471051	2.5340865	2.4774701	2.1215791	0.6601985	1.7917307	0.4714476	0.82546	1.7152345
2.2110745	1.4953882	2.1845659	1.0488639	0.6932305	1.6847739	1.5104556	1.3261346	0.1640139

1.4115889	1.1810389	1.3133039	1.584722	0.7792177	1.0093472	0.3983504	0.1275012	0.1589833
3.1952951	2.4868087	3.5980555	2.7254721	1.5267032	3.5727361	2.1210043	2.5600954	2.7107605
1.9158268	0.487795	2.5070588	1.401412	0.5407217	0.7380328	0.6135317	0.5315686	0.3063789
2.8669772	2.6565993	3.1352804	2.6043916	1.9332516	4.6669497	1.0401911	1.8631059	2.4868431
1.5375446	1.1598229	2.4065639	2.1779497	0.2319239	1.7121996	0.8583789	0.4376539	0.2543522
1.5767156	1.2137516	2.7137618	1.1372412	0.5649147	0.7011498	0.8885387	0.5649147	0.4141355
1.5002412	1.3378252	3.2700205	2.2448262	1.1984313	1.4279815	0.4276062	0.9744559	0.6989296
0.6778014	0.9594734	1.7457536	0.5484366	0	0.3349115	0	0.5315686	0.1765781
1.2331518	1.1382247	1.332221	1.269871	0	0.846072	0.6252705	0.235482	0.2930173
0.2701103	0.6484654	0.9423081	0.6375639	0.5665716	0	0	0.0816123	0.1900459
0.7027462	1.2778063	1.181357	0.9974731	0	1.0882773	0.2984237	0.0705267	0.1388145
0.97717	1.3191555	1.8032684	1.722772	0.625925	0.6699338	0.4455148	0.3761792	0.1298765
1.4919557	0.5691022	1.7421356	0.9015717	0.4955928	0.2368293	0.6285403	0.3975839	0.1918153
0.3900073	0.6512239	1.0290641	0.7640064	0.9760703	0.3958306	0	0.1135672	0.305562
2.1281284	0.9366662	2.7946446	1.3172462	0.8277377	1.2319853	0.8954577	0.80719	0.3996633
1.201822	1.5293212	1.1917521	0.7194901	0.8476763	1.2995377	0.6158871	0.5193406	0.4912887
2.1203851	1.5572875	2.3813664	0.9644356	2.6485115	0.976437	1.5726472	1.1344181	0.2149951
2.2128183	2.2793821	3.1888282	1.529721	0.725523	1.2761989	0	0.7796379	0.6736469
1.1239972	1.2170446	1.8772731	0.9585829	0.5949307	0.7932717	1.2796496	0.3183458	0.083792
1.6771248	1.5943097	3.6467157	1.8949535	0	1.7547593	1.0977456	0.4679055	3.2336887
1.8449078	1.0699771	1.5055353	2.0051125	0	0.790772	1.3860936	0.3758457	0.6616584
1.9109629	1.8339425	2.2132228	0.6654837	0.6597419	1.0945739	1.2336427	0.5488312	0.0922074
0.7470866	0.6557182	2.2008182	0.5085301	0.3148705	0.8447874	0.2171066	0.399007	0.1518588
1.8325255	1.467436	2.4114805	1.2657369	0.458487	1.3714473	0.458067	0.8157394	0.3696061
2.2113238	1.2808376	2.4254056	1.6781621	0.6035017	0.4212637	1.3865351	0.3709454	0.6954594
1.4143521	0.5129853	2.6414998	1.3607014	1.0607392	0.6697525	0.2397646	0.784504	0.7508637
0.3527585	0.7474304	0.5315686	0	0	0	0	0.0100637	0.1290851
1.422556	0.7063758	2.17728	0.6947465	0	0.4325319	0.1620811	0.3225051	0.3806186
2.2713659	0.7888527	3.2890672	1.8766053	0	1.1427402	0.269512	0.7539468	1.0588706
1.258941	0.7137838	0.7514637	0.5559143	0.8590154	0.4374408	1.3929768	0.1094274	0.0984196
1.316725	0.6794238	2.3131878	1.0563754	0.4221253	0.6366362	0.2944296	0.4714476	0.3011194
1.5570914	1.1137672	2.133432	1.6625701	0	1.2732772	0.7287477	1.8237494	0.1410403
0.4675925	1.0878023	1.6338497	0.186247	0.7786291	1.2162996	0	0.3330802	0.1487389
1.7790075	0.2235461	1.9103873	0.4960021	0.7742494	1.8833471	0.3605328	0.2949001	0.9510655
0.3759569	0.409581	1.0773114	0.4021764	1.2577951	0	0	0.0571383	0.217727
1.8678174	1.4056657	0.7587296	0.7589001	0.2626737	0.9356106	0.8465535	0.041243	0.2568899
1.0129977	0.8060353	1.6235392	1.1905517	0.3974744	0.2715452	1.1811662	1.0311128	0.0866479
1.3159719	0.9354597	1.9085439	1.8504794	0.1749177	1.177854	0.7543745	0.1042024	0.1923204
1.5385878	2.0122826	1.9967503	1.7161129	1.914488	0.9291092	0.3374257	0.8124572	0.1244605
1.7905218	1.8465134	2.4302853	1.6408985	0.5744406	0.9692334	0.723777	0.8515991	0.1727435
2.1323136	2.1811026	2.2997136	1.3975839	0.2221863	0.5207491	0.6363578	1.6775759	0.3207735
2.1636919	1.5084794	2.5590267	2.1481857	0.3926471	1.1973622	1.3378823	0.1842168	0.520045
1.160533	1.514703	1.8475159	1.3309599	0.60862	0.9362893	0.4325319	0.5177292	0.4114262

1.1203519	0.6441332	1.2417792	1.1634987	0.9665781	0.8307421	0.3981314	0.5813995	0.1487389
0.3961595	0	1.3396505	0	0	0.5675454	0.3171883	0.0753273	0.0676387
0.1880211	0.5960764	0.9079674	0.4601659	0.2830921	0.1909309	0.5192399	0.0593553	0.0982848
1.8610422	0.5193406	2.073786	1.1154994	0.6331057	1.6030269	1.1525728	0.2788174	0.3769571
1.2223718	1.2786985	2.1173626	0.6697525	1.4189455	0.7895206	0.7495772	0.3751785	0.2553194
1.3493653	1.582556	2.1497146	1.6892544	0.1294809	0.9411063	0.2481705	0.4183519	0.4825385
1.1307332	1.4966669	1.628307	0.7260465	1.2877087	1.1439158	0	0.0632268	0.1467855
1.2575538	0.6301259	1.584241	0.61946	0.3011194	0.2035144	0	0.3945141	0.9419326
0.9355352	0.6707497	1.2203919	0.5207491	0	0.7269184	0.4159749	0.4350952	0.1977396
1.4270161	1.8554715	2.0244615	1.2777468	1.0771746	0.4880008	0.5780209	0.7739963	0.1638851
0.7206287	0.6923379	1.9241759	1.1804025	0.2735756	0.4230941	0.1878944	0.6882251	0.2593026
1.0064058	2.3141164	2.0061544	1.6800993	0.2921928	0.1973622	0.8054576	0.296428	0.140386
2.536028	2.1382575	2.0690147	2.3942123	0	1.8950699	1.0476082	0.3375398	0.2224336
2.3699689	1.8768803	3.816999	2.9883211	0.7482036	1.6184736	1.1422174	1.3542265	0.9800984
1.1064823	1.3726178	0.8679755	0.405121	0	0.8529577	0.201132	0.371057	0.5604215
1.2904244	2.3961869	2.3364262	1.1832006	0.7036323	1.6096604	1.0332997	0.8467139	0.2184712
1.4063734	0.6901936	1.9492735	0.7662983	0.1199537	0.7476022	0.6036916	0.4881036	0.246104
1.5000882	1.8395162	4.1026413	2.259393	0.995376	2.3281471	0.3387952	1.0342862	0.5889005
0.3019388	0.1592417	0.5934016	0.3624418	0	0.2554403	0.2601461	0.0137838	0.0635029
0.7748398	0.9294123	1.6100386	1.1249236	0.3283769	0.4154342	0.8883828	0.4681141	0.7209788
1.3081278	0	1.6274668	1.219215	0.1937087	0.7283124	0.7399345	0.2226809	0.3353689
1.3893465	1.2261386	1.3045694	0.7275284	0	0.9914629	0.3243498	0.101919	0
1.6026469	0.9157886	2.0181712	0.5201456	0	0.5758932	0.7376002	0.2409859	0.1768334
1.5509991	0.8628283	2.5198186	2.2652568	1.3568199	0.5390347	0.5481407	0.6667566	0.4083858
1.5410192	0	2.4305529	1.217665	1.4777809	0.9992063	0	0.3879139	0.3012364
1.7989629	1.7648132	3.1200699	1.4653476	0	1.374344	1.5339121	0.6053047	0.4820222
1.7382058	0	2.1144337	1.606537	0.6691175	0.2541102	0.9874664	0.5429019	0.488515

RAB30-DT	IRF1-AS1	AL031666.1	AC092809.2	LINC02518	TMEM30A-	LINC00592	AC005837.1	AL355075.2
2.0271894	2.5225078	0.9830939	0.1595	0.2717842	0.2172307	0.0483757	0.4413772	4.0095622
2.219153	1.2246577	0.7349155	0	0.363115	0.7680784	1.0467006	0.5389354	1.6183796
1.5496199	1.0673634	0.5287713	0.0661236	0.4142438	0.371057	0.6289135	0.1000357	1.2736949
2.0418037	0.717649	0.7191396	0	1.4440839	0.7725614	1.1230702	0.5192399	2.6634585
1.8789607	1.1844707	1.4722278	0.8361665	0	1.0178507	0.3267674	0.3029916	1.2863489
2.3051534	2.1594031	2.5928995	0.8397984	0	0.6725611	0.4389315	0.4406333	2.5449552
1.8124572	1.2053925	3.4442032	0.0997665	0	0.6393248	0.4156505	0.0769695	1.5699285
0.9912452	0.7201033	0.5323668	0.576764	1.5661819	0.2960756	0.2229282	0.2486563	0.153935
1.4241159	1.2600257	0.5352567	0.2454957	0	0.3795101	0.051024	0.3063789	1.8953414
1.971332	2.0168885	1.3100492	0.3846025	0.1247252	0.4890291	0.5311693	0.2090166	1.9780492
1.8297278	1.4411116	1.8283879	1.3333092	0.0839282	0.2765562	0.2352369	0.1419559	1.6053047
1.0082717	1.3058538	0.3941848	0.1196882	0	0.1792562	0.1385524	0.0924781	0.5613017
1.9942176	1.3791219	3.3919739	0.3969267	0.1112985	0.6758159	1.2454348	0.4288928	0.1691554
2.0431345	1.0161397	0.8934399	0.2729787	0	0.3615438	0.590626	0.4281424	1.2242873
2.3719212	2.4386921	1.1145003	1.2651968	0.1228715	0.4312485	0.2330291	0.2531422	2.4355219
2.257584	1.1423481	0.4756037	0.1819294	0.1624679	0.7178245	1.3598023	0.3302142	1.8425766
1.8420134	1.8026068	0.8827604	1.3655246	0.1981169	0.9859375	0.1646576	0.0451634	2.2863489
1.2443391	1.2643563	1.7211539	2.9897748	0.1659441	0.5063988	0.263395	0.4523846	1.0934926
2.4138919	2.6021243	0.8942163	0.077243	0.4235244	0.4748773	0.3106891	0.348799	1.6396026
2.6500522	1.5897156	0.4883093	0.2446435	1.3999367	0.2697513	0.0382957	0.4369081	1.1828829
1.3330802	0.8020691	0.7246503	0.2663968	0.2067682	0.3673711	0.1611783	0.092884	1.7268312
2.8730848	1.5486832	0.5303704	0.1904252	0.115566	0.3352546	0	0.3242346	2.3613473
1.5345597	1.3035762	0.6157929	0.7414023	0.1483485	0.3175356	1.2981304	0.1892869	2.2814015
1.2415962	2.100103	2.1421193	0.6594679	0.1560082	0.7753456	0.248049	0.3736204	2.5307949
1.2075181	1.467436	0.7230779	0.962438	0	0.337654	0.0859685	0.2157407	1.4533332
1.4734752	0.5961719	1.6187555	0.209391	0.1871343	0.4127273	0.2413521	0.2740529	0.5155106
2.2722621	3.1565099	1.4216407	0.9527808	1.1184262	1.0662614	0.2144979	0.1166309	2.9925872
2.2846326	1.5309197	0.8188506	0.8390727	0	0.4800583	0.2056427	0	1.6638911
2.4713436	1.3324502	1.1358632	0.1042024	1.2196487	0.3923174	0.9713688	1.1023222	1.5129853
3.3462478	3.2549719	3.0211155	0.765662	0.2276179	1.5264695	0.1437199	0.4836223	2.6550197
1.1775351	0.6171104	2.7204536	0.5996987	0.0852887	0.2111368	0.3337672	0.2113861	0.8876033
1.2470768	0.3402774	0.2570106	0.2375637	0.2125071	0.1531568	0.1431322	0.1852323	0
2.7077015	1.2258302	0.7123757	0.1651723	0.0756011	0.450591	0.2981891	0.158337	0.9385493
1.9190731	2.2590615	1.1844073	1.2568295	0	0.7270056	0.6767187	0.502025	2.6203283
1.4863025	0.5044679	1.1314578	0.2992447	0	0.4469972	0.2376861	0.1348781	1.0885487
1.345226	1.1528973	0.7583885	0.1839628	0.4197546	0.5018213	0.2319239	0.3216972	1.230203
2.7996261	1.0928163	0.0844725	0.1150332	0	0.9947969	0	2.0181	2.4161101
1.2598449	1.546413	0.2961931	0.143524	2.9746761	0.1012467	0.6864333	0.1110313	0.6552602
1.7408412	1.6346862	0.5723076	0.810731	0	0.7842527	0	0.597412	1.6196478
2.5016684	2.6591254	1.9825099	1.6324544	0.2164859	0.6692989	0.2784606	0.3556929	2.290159
2.8636412	1.9411439	0.8333354	0.9715895	0	0.534958	0.5340616	0.6405284	1.9850267

1.463884	1.2252749	0.4406333	0.2759606	0	0.3923174	0.2686739	0.5693939	1.643533
1.858737	2.4047669	1.1821838	0.1372412	0.1223414	1.0045373	1.0540844	0.6181447	2.4307135
2.8647309	2.0324888	1.0961269	1.870345	0	1.2292803	0.162339	0.3341106	2.1881793
1.4679576	1.1742146	0.7641763	0.5016175	0.8346303	0.4140273	0.7754299	0.0836559	0.9476288
1.7995847	1.696528	0.9371937	0.1499097	0	0.587269	0.4340277	0.1708224	1.9486382
1.7617533	0.803723	1.5558162	0.3415304	0.559345	0.3047447	0.6029319	0.2683146	1.7855087
1.707569	1.4828999	1.1631122	0.2983064	0	0.6040713	0.1566555	0.4612142	1.6204457
1.957246	2.4921608	1.6140502	0.9230351	0.5274708	0.5899552	0.0813396	0.1059462	2.4635963
1.6221807	0.8802545	3.1815797	0.242572	0	0.700706	1.0892269	0.4224483	0.2817873
2.3020265	1.2162996	1.1543888	0.0661236	0	0.4140273	0.3171883	0.9772433	3.7686819
2.8330925	1.9641029	0.8514392	0.1224739	0	0.3248106	0.072586	0.0946415	1.9716631
2.3793714	0.8435015	0.6480972	0.0731347	0	0.4606901	0.7890197	0.9969672	2.5867888
2.6351043	1.8534767	1.5095945	0.7341351	0	0.8719225	0.0900404	0.7458827	1.9013786
3.8682587	3.141411	3.3989249	0.3761792	0.2926247	1.1576258	0.174449	0.5983017	3.2644413
1.6226961	1.4872805	2.2452218	0.9185389	0.2933705	0.3190398	0.4152178	0.6634813	1.0347792
1.5409696	1.7411002	0.5360529	0.3013535	0	0.3438058	0.1832006	0.5598344	1.6455172
2.5330151	0.8474357	0.1844707	0	1.1149666	0.5169229	1.9226926	0.3645724	0.8274939
1.6659385	1.4238471	0.4317834	0.6199294	0.0705267	0.8615584	0.1573025	0.5401265	1.3524196
1.9252019	1.9394523	0.5280712	0.2042659	0	0.2683146	0.2711866	0.0549179	1.9213982
1.9444462	1.9861196	0.8675012	0.8292001	0	0.4840866	0.1432628	0.064469	2.1534487
1.0521376	1.7574927	0.6016014	0.6988408	0.2115107	0.3474389	0.0729976	0.0950467	1.4399954
3.3254584	1.8923133	0.6835617	0.3540007	0.2597847	0.7221162	0.2167342	0.5953127	3.3028601
2.3354833	1.9459073	3.1771365	0.3465315	0	1.066537	0.0885487	0.3682655	2.8909719
4.0564708	2.7550229	3.0025765	0.1801479	0	1.1029268	0.3880609	0.5448151	4.0361721
2.8438231	1.7463558	0.8175414	0.9508416	0	0.2565276	0.2263852	0.2905424	2.2771218
1.5368984	1.158337	0.2090166	0.402613	0	0.1432628	0.1700532	0.4097982	0.668936
1.7054029	1.2630945	1.6311509	1.1619522	0	0.3394795	0.0842004	0.5201456	0.7729836
1.8256228	1.0127832	1.668573	0.2801249	0.5293712	0.2002532	0.8406042	0.4955928	1.5186358
1.3407332	1.1837088	1.3949531	0.1343524	0	0.6441332	0.1553607	0.2468337	0.3427823
1.6782973	1.23008	0.9969672	0.0690147	0.0612923	0.3474389	0.5425057	0.0267293	0.7424374
1.7717588	1.3666999	0.7363018	0.2051422	0.1833277	0.5738592	0.6988408	0.3032255	1.7268748
1.230449	1.4943132	0.4456208	0.0373119	0.5044679	0.2371966	1.98167	0.4868688	0.1004395
1.0025225	0.8319583	0.6932305	0.0794293	0.3798427	0.6549854	0.6115501	0.1199537	0.8304176
2.5598589	0.8726318	2.1825334	0.0852887	0	0.8242383	0.3200803	0.2467121	2.7782927
1.5890444	0.9483018	0.4055568	1.0513721	0	0.4527009	0.353662	0.7518921	0.7270056
1.5922059	1.5506545	0.8172958	0.1119661	0.1314578	0.2742915	0	0.2945472	1.1264443
2.3367975	2.0130692	1.3434647	0.3811726	2.9286356	0.6158871	3.2326147	0.6229304	2.4419349
0.820567	1.1674222	0.8639385	0.2937236	0	0.1357975	0.1296127	0.229957	0.3402774
3.1607911	2.0475384	1.705049	0.9026525	0	0.9477784	0.6900147	0.3563692	4.1786509
1.3873625	1.8070251	1.0316069	0.0444641	0.0777899	0.5248661	0.0766959	0.1934564	1.4427047
0.8102374	0.7084963	0.646347	0.5780209	0	0.1244605	0.1051417	0.0833836	0.3619929
1.8904856	1.3241193	1.4402612	0.102591	0	0.6869711	0.1471764	0.1171631	2.8443254
1.4984041	0.5898593	0.7563402	0.0783366	0	0.4237395	0.0908534	0.329181	1.3415304

2.6252003	1.4871261	0.2803625	0	0	0.0581086	0.0487942	0.2397646	2.243334
2.6837863	1.9566515	1.8010759	0.2510828	0.116764	0.57415	0.7108781	0.5936884	1.6275135
2.5667665	3.0236989	0.631989	1.4575943	0	0.2851063	0.2260152	0.3417581	3.1180108
1.1573671	0.8026482	0.3221589	0.342441	0.0947766	0.3879139	0.5541468	0.1971105	0.7059337
1.2600859	1.3029916	1.6128243	0.3059122	0	0.246104	0.1265765	0.3115031	0.8996397
2.0090605	1.4016851	0.7691793	0.9221978	0	0.6510402	0.1202192	0.3622174	1.4006472
0.8324445	0.4801617	0.5754092	1.1371756	0	0.2606279	0.2322924	0	0.8674221
1.7231216	1.1963552	1.5845777	0.4731115	0.0875986	0.4571215	0.8329305	0.3448285	1.9671686
1.6358008	1.8656813	1.0059748	0.4169478	0	0.3252713	0.223299	0.1972364	2.144438
2.339451	0.7447639	0.4978423	0.1415636	0.1263121	0.5518851	0.7580473	0.3519675	2.1840581
2.0216219	1.7883516	0.6226493	0.2661569	0	0.4301782	0.2108875	0.3896769	2.9419138
1.646347	2.1153995	0.3616561	0.7153663	0.2505978	0.6139088	0.0588014	0.2521734	2.5122775
2.261952	1.3992805	2.1842168	0.3413027	0	0.6913555	0.0930192	0.3026408	2.5670586
0.7073481	1.0613615	0.3555801	0.5751187	0	0.1210154	0.5345597	0.111432	0.2537473
1.7045622	1.7648557	0.374177	0.6680283	0	0.3949531	0.178874	0.4294285	1.166844
1.6928289	1.2741125	0.8912636	0.3466449	0.2147465	0.1050075	0.0742314	0.2723816	0.996678
2.376596	1.6714293	2.0453032	0.3228511	0.3746222	0.4315695	0.1017845	0.3287216	2.7292265
3.2018847	1.0243197	2.3016754	0.3568199	0.0990932	0.5429019	0.458067	0.6901936	2.3122295
1.7025689	1.3140584	1.0844045	0.269871	0.4485838	0.410124	0.5650122	0.1092937	1.2711866
1.0408924	1.1676148	0.5223571	1.0267293	0.1333005	0.1654296	0.0889557	0	0.6784325
1.6692082	2.1696364	1.0590091	0.1495195	0	0.2839218	0.059909	0.0395596	1.7657045
2.3632552	2.0099562	0.9066597	0.5511961	0	0.4288928	0.4674882	0.3941848	1.4088205
1.6997293	0.8381854	0.5048746	0.2537473	0	0.3213509	0.1531568	0.1350094	0.9114998
2.1536757	1.175812	3.2380837	0.5597365	0.1891604	2.131787	0.9065828	0.3810618	0.2835662
1.6367754	2.1729035	2.2366763	1.4563331	0.1461338	0.3515153	0.3955016	0.546166	2.7626252
2.9896295	1.2387257	2.3531821	0.5753123	0	0.7441611	1.3101074	0.8073549	3.4488217
2.83289	1.2811344	2.4594316	0.0619835	0	0.4440309	0.8835426	0.556503	2.2824398
1.5711428	1.0041058	1.6640732	0.996678	0	0.3015876	0.0654344	0.2789363	2.1707263
2.1103298	2.0418387	1.13888	1.6678921	0	0.8761731	0.0714881	0.2630344	2.7794909
2.0304773	0.4726957	0.5554235	0.1078218	0.0647449	1.5298209	0.293959	0.5014137	1.188844
1.3321064	2.0863082	1.283507	1.0631578	0	0.4829515	0.2125071	0.2730981	2.8052305
1.466862	0.7955176	1.136782	1.3607014	0	0.4143521	0.7426961	0.7062874	0.7489764
2.4142167	1.410721	2.2456782	0.1747899	1.1981169	0.3500446	0.2930173	0.6095659	0.4373343
2.7724558	1.905736	0.4979444	0	1.1750455	0.6765382	2.4058018	0.3258469	1.8992917
2.8602477	1.2321081	0.736042	0	1.2015711	0.9294123	0.279293	1.3060872	1.3284918
2.8498192	2.4793341	2.4707451	0.7905218	0	0.9575432	0.2414741	0.3095255	2.688158
1.8532372	1.6992407	1.0427144	0.43328	0	0.5723076	0.2266318	0.2371966	2.8912442
2.4261305	0.8634628	0.3576083	0.090176	0.2973675	0.2159892	0.2934882	0.5790836	1.0457924
1.4317834	1.005903	1.5591002	0.3225051	0.0626744	0.969086	0.1781091	0.1319845	1.6482353
2.3209756	1.7069062	0.5781175	0.3997727	0.5806279	0.6213369	0.4317834	0.3846025	1.9460196
2.4668359	1.8189323	2.1574318	1.1373724	0.3140584	0.8523185	0.4218023	0.5320676	2.5139707
0.8201585	1.7462698	0.5479433	0.2474415	0	0.1083572	0.1206174	0.3632272	1.2246577
1.445091	1.3058538	1.3265949	0.3328511	0	0.1245928	0.5397296	0.3761792	1.2065807

1.9682382	1.0875986	3.2304182	0.5228593	0	0.6127771	0.5223571	1.621712	2.9429836
1.121546	0.8783722	0.1754288	0	0.3455099	0.7383787	2.5908894	0.1593708	0.640991
2.7012163	1.7106577	2.1802752	0.232538	0	0.2564069	0.1664584	0.4870747	1.807561
2.8528179	2.7685655	1.1590479	0.4618428	0.093425	0.230326	0.7307486	0.3331947	2.5025343
1.684325	1.8043014	1.431409	0.4812989	0.2758414	0.3632272	0.1575612	0.3131297	1.8958455
1.8824865	1.753091	0.7701948	0.0968016	0.3173041	0.3004166	0.1651723	0.563451	2.0183849
2.7330506	2.329669	1.6502131	0.3166092	0	0.6441332	0.3628906	0.5553254	2.9533025
1.388961	1.3699968	0.8115532	0.3751785	0	0.4844991	0.4287856	0.3393655	1.320138
1.9880848	2.6930297	0.354565	0.8214653	0	0.3309025	0.2464689	0.0688771	1.636497
1.8536763	0.8617172	0.7510352	0.8620347	0.1009776	0.373509	0.162339	0.8335783	1.7958916
2.5231856	1.015997	0.0729976	0.2800061	0	0.5327658	0	0.7274413	1.8284691
2.0755669	1.2116353	0.7842527	0.6292867	0.3384529	0.5826523	0.1582077	0.3406192	2.1428055
2.2062681	1.9192257	0.1410403	0.4364817	0.4603756	0.1796384	0.4107753	0.1829465	0.8294437
1.5175781	1.5472524	0.6468999	0.6145685	0.5871729	0.2944296	0.3427823	0.4354152	2.1074535
1.2817873	0.9718102	0.6050202	1.2836255	0	0.2249046	0.2149951	0.1721034	0.8141812
1.4320508	1.6002222	0.6271399	0.3784006	0.1224739	0.1964811	0.1208827	0.1063483	0.9721044
2.0147481	1.4817639	1.4033768	0.2758414	0.4579619	0.2865263	0.821547	0.4032677	1.729792
1.946918	2.0870554	0.561595	0.4588019	0	0.65306	0.1493894	0.0997665	1.9373444
0.9530044	0.9645095	0.5509991	0.1110313	0.1914363	0.3912178	0.7249994	0.3854863	1.070458
0.963844	1.2206395	0.4358419	0.5710457	0.1673579	0.2890081	0	0.1456122	1.223299
2.5634754	1.5479927	2.5189883	0.0554733	0.0970714	0.4494293	0.0326651	0.5660845	2.0455828
1.9427209	0.4520682	0.8105665	0.2565276	0	0.1830736	0.2949001	0.5328655	1.5662794
2.2671762	1.7503921	0.0747794	0.101919	0.1761951	0.3618806	0.3771793	0.3564819	2.0072673
1.831026	1.0339339	0	0.1558787	1.3316479	0.7051375	1.6214776	0.5003942	0.7701102
2.5145515	2.0226874	0.3940751	0.1942132	0.3283769	0.6301259	0.1163648	0.1509495	2.8757997
0.9076598	0.3818371	0.533364	0.1216786	0	0.077243	0.4148932	0.1823109	0
2.1444054	1.8242791	1.1612428	0.1819294	0.1102296	0.5529672	0.0371713	0.3103401	2.4740985
1.1695402	0.5988416	0.0472592	0.2431816	0	0.5703658	0.3111543	0	0.1714631
1.8554715	1.8315125	0.729618	0.1142337	0.1967329	0.486251	0.3103401	0	1.3702201
2.480963	1.9912815	0.673285	1.0299829	0.0922074	0.7554004	0.406319	0.4241696	2.5380911
4.5559664	2.9488998	4.5268763	0.2319239	0.7020369	1.3598866	0.0435545	0.4788165	4.6553347
1.117961	1.1031283	0.0630887	0.1676148	0.6286336	0.6775308	4.3966938	0.0663992	1.0114241
1.6763577	1.7882263	0.8024828	0.2689134	0.164915	0.5604215	0.354565	0.4498519	0.8081791
1.2136272	0.4145685	0.3940751	0.23008	0	1.2884176	0	0.3760681	0.6729231
1.6542064	1.9881939	1.2506585	1.124659	0.24123	0.4996801	0	0.2105136	1.8236679
2.1079891	0.5234616	0.3968171	0	0.527771	0.8312287	2.2218771	0.2516888	0.7525773
1.2421452	1.3484591	1.4454619	0.3747335	0	0.4378669	0.0963968	0	1.2044538
2.1819294	1.576764	0.9194545	0.3985693	0.9157886	0.3467584	0.6623878	0.5724046	1.3919327
1.7266133	0.6823935	0.2077679	0.1261799	0.0758749	0.1833277	1.2749474	0.2467121	0
1.7612002	1.1357975	1.7164203	0.65764	0.0720372	0.2972501	0.3817264	0.483158	0.5619859
2.0758406	1.9443712	0.5857317	0.5721136	0	0.4682184	0.6262989	0.2463473	2.5620836
2.0936954	1.703898	1.5994606	1.643533	0	0.5290713	0.3341106	0.1184926	1.8529976
2.1513393	2.6798967	0.5004961	0.8360049	0.3255015	0.769264	0.0588014	0.5229597	2.6090457

1.3166671	1.1779816	0.248049	0.3894567	0	0.1844707	0	0.1096949	0.5118728
1.9111547	0.7983409	0.3955016	0.3341106	0	0.4962067	0.2659169	0.413919	1.2824398
1.6333382	1.1148334	2.6226024	0	0	0.4328525	0.3355976	0.4985572	0.9916806
1.9864473	1.2851654	1.8320799	0.3292958	0	0.2582174	2.7945614	0.1979912	1.7935214
1.2821432	2.1047728	0.907506	0.6806844	0.4103411	0.3529845	0.0384362	0.2784606	1.7201909
1.6853123	1.6962164	0.6107945	0.7432133	0.1268407	0.2908962	0.539134	0.2609891	1.6495695
2.8915942	1.5887086	0.5150059	0.1354035	0.2321695	0.537644	0.9819987	0.5380415	2.9058707
1.9029225	1.139011	0.1589833	0	0.1918153	0.379621	0.3565946	0.3860384	2.3752341
1.6033118	1.092884	1.2105136	0.4102325	0.1963552	0.8110599	0.4514353	0.2995963	1.7381193
1.3156242	0.9614752	0.9221217	0.6494775	1.3291236	0.3374257	0.8705817	0.1009776	0.8331735
1.6950584	1.5273206	2.3638719	0.3519675	0.1491293	0.372395	0.3223897	0.3844919	1.3328511
2.1384541	2.6894781	1.6250834	1.4631516	0.7882681	0.9833858	0.1845977	0.2917214	2.233612
2.7806671	1.9658766	0.8125393	0	0	1.5172253	0.0571383	0.2133784	2.2679253
1.5499156	1.9237957	1.3339389	1.373676	0	0.516822	0.1343524	0	1.3849339
2.4404739	2.2197416	0.7446778	0.1497796	0	0.3142905	0.1317212	0.461319	2.3393655
1.7149269	1.2567088	0.5094425	0.4592218	1.075738	0.3603081	0.2263852	0.3639	1.5017703
0.7524061	1.4379202	0.7814856	0.9403547	0	0.328262	0.2702299	0.2826177	0.6626613
2.3344253	2.5965297	0.7126398	1.213565	0	0.5743438	0.0405418	0.1534162	3.0910058
3.0499792	2.6470612	1.0888879	1.3004166	0.2106382	0.5174269	0.1418251	0.346418	1.6191313
2.6374248	0.9577661	0.8433407	0.1428708	0	0.4834676	0.0850167	0.63951	1.9494976
2.0644	1.0114241	0.5116704	0.0909889	0.1576905	0.5566011	0.2038902	0.458172	1.3846577
1.1745343	1.9581003	1.1815479	0	0	0.1984942	0.8463128	0.2429378	0.6458861
2.0187054	1.7752613	0.2708279	0.8525582	0	0.2117598	0.3361691	0.6266728	1.7971375
2.6216651	0.7767778	1.7701948	0	0.093425	0.3713916	0.2336427	0.1944653	2.3926197
1.559296	0.8459115	0.2229282	0.6901042	0	0.2532632	0.0630887	0.0822938	0.7069062
2.4545181	1.3017632	0.738984	0.4597464	0.2406196	0.6673018	0.4622618	0.9752633	1.5238128
1.3497616	1.4005926	0.8291189	0.9383987	0.6245221	0.6042611	0.2363395	0.2330291	0.2748878
2.2345626	2.7834567	0.8381047	0.1732554	0.1545832	0.3604205	0	0.3151025	1.461319
0.9976176	1.3713358	0.7252613	1.0969365	0.1457427	0.1805298	0	0.0647449	0.9918982
3.9110236	3.7455099	3.0780803	0.6998773	0	1.0142124	0.0753729	0.9822422	3.0463746
2.6204691	1.8667102	0.7266569	1.2194628	0.3246954	0.666393	0.8044666	0.2843957	2.4764595
1.8389518	1.8045492	0.5129853	0.3046279	0	0.2385423	0.3493653	0.4435006	1.7058895
2.35225	0.9753367	3.6470727	0.9362893	0.3002995	0.1770886	0.1460035	0.4218023	0.6544355
2.0495611	1.0748479	2.1253866	0.43957	0	0.6950139	0.0729976	0.2683146	0.7943535
2.5973643	2.1899827	1.4671229	0.0810669	0	0.2834477	0.3056787	0.6558098	2.3601957
2.0846426	2.3648245	0.4839834	0.4497462	0	0.2258919	0.4003193	0.065848	2.0061185
1.9855733	0.9553128	0.6129658	0.2242873	0	0.2754839	0.6672109	0.0606008	1.3455099
2.4894916	1.6835617	2.6510632	2.955443	0.1765781	0.5950262	0.7886857	0.7913557	1.3868661
2.1978339	1.6921593	3.1091767	0.9070445	0.1335635	0.3577209	0.2525368	0.3233124	2.4623665
2.5315686	2.1170966	1.9269482	2.2804516	0.1276333	0.7681631	0.3827226	0.484396	2.3663922
1.4092008	1.116764	0.9758503	0.1722315	0	0.2662768	0.2218152	0.3953919	0.3349115
2.9115765	1.162339	2.7005507	0.1106305	0	0.9241759	0.2458607	0.3149865	3.0789855
2.2135961	1.9362893	2.1528	0.7466567	0	0.2202061	0.4802651	0.3327366	2.5587329

1.8433407	0.7803941	0.1926992	0	0	0.6167341	0.2752455	0.23352	2.2314939
3.8999876	3.2457644	2.8692924	0.9886544	0.054779	1.1638207	0.1663298	0.7103491	3.4559387
2.3561156	1.2810157	1.1685137	0.4915966	0.9571717	0.3586213	0.3687125	0.5401265	2.4725917
4.2248313	3.4020468	2.9600761	0.8418121	0	1.0573116	0.1411712	0.5731806	4.8247704
2.1390438	2.3253576	1.0313246	0.067501	0.3275724	0.40021	0	0.1973622	2.1876411
2.6050676	1.5782625	1.7343953	0.1784916	0	0.561595	0.4415897	0.3046279	1.7191834
1.8991756	1.9855733	0.4992719	0.5831339	0	0.8880711	0.4132691	0.5216538	2.8203832
1.0876665	0.996678	0.7815695	0.7310963	0	0.2430597	0.0896337	0.1166309	1.1436546
1.9682751	1.0517201	0.82546	0.2237932	0	0.3993352	0.1346152	0.1742786	1.9138755
1.1964182	0.6681191	0.3651326	0	0	0.186247	0.2539893	0.2657969	0
1.5299708	0.8223631	2.3975566	0.2504766	0	0.2199584	0	0.1009776	1.0302654
1.4804202	1.0007933	0.482745	0	0	0.3755121	0.5208497	0.2045164	2.6313604
1.7480747	1.6919361	1.2573124	2.2490205	0.0930192	0.3986787	0.285343	0.2984237	1.2273714
1.3524196	0.9101954	1.0094905	1.644087	0.3052118	0.3837181	0.3458505	0	0.7911056
1.9553872	2.3099329	0.9849903	0.3604205	0.3238888	0.3849339	0.0583858	0.5204474	2.1735112
2.5695883	0.775767	0.5005981	0.2865263	0	0.5440897	0.5212518	0.8800978	0.5686159
1.9548663	2.631081	0.4796445	0.3465315	0.1112985	0.2721427	0.2760797	0.230326	2.3517979
1.9131479	2.2007554	1.3589026	0.1631122	0.1454818	0.5680321	0.5735684	0.5439907	2.5287713
2.8520388	1.5518359	1.1386834	0.2585792	0	0.2187192	0.1133004	0.2804813	2.6241243
2.7881428	1.5029925	1.6485115	0.3717261	0	0.5677401	0.1852323	0.1826923	2.9611417
1.6301725	1.9446711	3.8394356	0.4419084	0.4469972	0.9290335	0.1236662	0.8028136	1.4271234
2.9720493	2.8803721	0.5487325	0.6018866	0.5096452	0.3774014	0.0970714	0.349705	2.3048031
1.8262739	1.9281239	1.7796379	0.5548344	0	0.212009	0.209391	0.0721744	2.1697647
1.9604005	1.1104301	0.2007554	0.5484366	0.1257833	0.3864799	0.5647196	0.1092937	2.1799887
2.1454166	1.9240999	2.5622791	1.3071953	0.2249046	0.338567	0.1152996	0.6622055	2.260658
1.1838993	1.6144743	1.9460945	0.1037997	0.1219437	0.4899539	0.1582077	0.2279875	1.3628345
1.7643462	0.8571052	1.8643347	0	0.3564819	0.440527	0.0328061	0	0.9838236
1.2562257	0.8544745	1.0236102	0.4774701	0.605684	1.3720606	0.4557546	0.3365119	1.3882447
2.8649488	2.2139693	1.3440899	0.1172961	0	0.4239546	0.1357975	0.539928	2.9110779
2.361628	0.4354152	1.7578767	0.291132	0.1791288	0.1557492	0.5633534	0.6511321	0.8635421
1.4407396	1.2630344	0.9120366	0.5527705	0.1147668	0.6033118	0.0387171	0.0996319	1.3448853
1.9385116	2.0297002	1.1511444	0.6249899	0	0.3718376	0.2504766	0.1692837	1.8211796
0.6822137	0.941482	1.5614483	0.3358262	0	0.1673579	0.275007	0.1704379	1.6606549
1.5832784	1.166137	2.5755544	0.0551957	0	0.6404358	0.0641931	0.3090597	1.74859
1.3187507	0.7138717	0.6877774	0	0	0.2396424	0.1231364	0.2332745	0.5061957
2.4224752	0.9403547	0.7116711	0.0771062	0	0.6735564	0.045443	0.417812	2.0592168
1.962586	1.8390324	0.7075248	0.231801	0	0.2110122	0.4918018	0	0.7812338
2.2410164	0.7884351	1.9469554	0.0501882	0	0.4005379	0.1416944	0.1484786	2.7947486
2.2000335	2.7686501	0.4800583	0.148869	0	0.4703026	0.3618806	0.2220626	2.771294
1.2551986	1.9682382	0.1343524	0.4886178	0	0.5156115	0.20989	0.1411712	1.7658742
2.2611095	1.4682184	0.791439	0.4006472	0	0.3268824	0.4003193	0.0496308	2.8400402
1.9239858	1.4830031	1.8483575	0.3309025	0	0.5866927	0.0705267	0.0919367	3.001478
1.8476763	2.3272562	0.4319973	0.4693651	0.117961	0.1978654	0.1531568	0.2435472	1.8115943

1.7934798	1.0129262	0.7367347	0.3358262	0	0.3278023	0.8186052	0.2637556	1.2875905
1.2113861	0.7409707	0.3726178	0.1393385	0.9253918	0.2767944	3.3532245	0	1.2594232
1.1779178	1.2775087	0.230326	0.0835197	0.1448295	0.5415148	1.2349304	0	1.1018517
2.1912468	1.1680001	0.9923334	0.0709388	0	0.8531973	0.1219437	0.1580785	1.968017
1.5995558	1.6757256	1.0540149	0.4944668	0.3468718	0.4079509	0.1814206	0.1851054	0.7674853
1.4837255	1.5601769	0.2704691	0.2077679	0	0.2898345	0.1245928	0.0558898	1.9134926
1.8226894	1.2365232	0.7290089	0.2606279	0.0706641	0.5460672	0.6570912	0.4746697	2.2660369
1.8094968	1.0302654	1.7092465	0.1732554	0	0.4934424	0.1033969	0.4240621	1.6340356
1.8706606	0.9836777	0.7662983	0	0.9838236	1.058455	1.4041402	0.0740944	1.4357353
2.6621143	1.6175336	0.1027253	0	0.3106891	0.4059924	0.7408844	0.4467856	1.9928772
1.6690721	1.1508846	2.1633377	1.0635029	0	1.0035303	1.2144357	0.2601461	1.0730661
2.0666748	1.1804025	2.1537405	0.0863762	0	1.1766419	0.23842	0.5577777	1.1288873
2.6608146	2.3811449	0.8728682	0.2340107	0	0.8245642	0.3294107	0.6837414	2.7672522
2.9099843	2.5895718	1.8747576	0.4578569	0	0.5011079	0.2403754	0.6522341	1.7635815
0.8617172	0.8678174	0.3178829	0.0437644	0.679604	0.1300083	0.1709506	0.1609202	0.8858867
2.8961943	2.2983357	0.4276062	0.9949417	0	0.3918777	0.0708015	0.0468403	2.7176929
1.6903724	1.2605677	0.164915	0.2224336	0.2251515	0.3199647	1.1955996	0.4288928	1.3250985
2.0880737	2.3630309	0.8740494	0.6285403	0.1333005	0.6845495	0.7654499	0.4604805	2.5735684
1.5043153	0.5080229	0.3295255	0.0387171	0.6135317	1.1918153	1.8080555	0.1152996	0.979001
2.0052563	1.0174231	0.2678354	0.1895399	0	0.5639391	0.316725	0.1473067	1.7294875
1.6352436	1.3931965	1.3818924	1.4527536	0.4330663	0.5685186	0	0.380951	2.0895659
2.8195864	1.2989515	0.2028878	0.272501	0.6351507	0.3336527	0.3817264	0.5630605	2.4784283
1.7662135	0.7149269	0.7223786	0.2708279	0.5735684	0.3205424	1.3356548	0.2758414	1.3296977
1.7779141	1.863661	0.7882681	0.415326	0.5084287	0.4270698	0.3218127	0.1964811	1.5680808
2.6899924	1.8864331	2.4403144	0.6420084	0	0.4127273	0.4939547	0.2803625	1.5489298
2.514602	2.9906282	0.4912887	0.6909981	0	1.3868109	0.2174789	0.4695734	2.3632832
2.2869994	1.0968016	0.7204536	0.1125	0.1938348	0.6756353	0.1911836	0.4564908	1.2247195

AF230666.1	AP4B1-AS1	AL031600.2	AC006017.1	AC009961.1	AC010542.5	AC008937.3	AP000346.1	AC018845.3
0.7427823	1.835722	0.3221589	1.7149269	0.6710216	4.5057703	0.9288061	0.579856	1.3153923
0.1313261	1.0985543	0	1.3047447	0.9124966	1.9857554	0.5433969	0.1764504	0.3493653
0.5430009	0.4003193	0	0.5436938	0.3968171	1.9037324	0.2838033	0.2947825	0.7793858
0.5865006	0.166844	0.4287856	1.1783641	2.135502	2.206737	0.169925	0.57415	0.2701103
0.2073931	0.9203695	0	1.7223349	0.7060221	2.9265119	0.8283066	0.144699	0.9173936
0.4992719	1.8308232	0.5469562	1.3852654	0.6521423	4.4179673	1.3683772	0.429964	2.1908045
0.08882	1.0603933	0.3858175	3.0481664	0.4281424	2.8397782	0.4724878	0.119821	1.2981304
0.2406196	0.0440443	0	0.3102238	0.6535187	0.8839336	0.4311415	0.0697022	0.1431322
0.410124	1.1187584	0.3387952	2.006693	0.5917751	3.1140671	0.4671751	0.1040682	0.5582676
1.0894982	1.7122436	0.725872	1.5305702	0.6000794	2.9692518	0.6693897	0.2445218	0.4740466
0.3440331	0.5466599	0.5197432	1.4406865	0.779806	2.340904	0.6060631	0.1667154	1.3494219
0	0.2590615	0	0	0.1701815	0.3758457	0.4160831	0	0
0.1638851	0.0486547	0	0.4364817	0.5656947	2.8530176	0.097476	0.219215	1.320658
0.2444	0.2115107	0.1998765	0.346191	1.2571917	1.9949779	0.7407117	0	0.1222089
0.6575485	1.0973411	1.3230241	1.611314	0.7249994	3.1589509	0.9485261	0.3131297	1.5433969
0.3759569	0.3272275	0.6556266	0.47892	1.7651104	1.5106582	0.8312287	0	0.4247071
0.1979912	0.8867452	0.2358496	0.9193019	0.4439249	1.2793524	0.7428686	0.0706641	1.0402612
0.2420842	0.6536104	0	0.4882065	1.0891591	1.5319678	1.044604	0	0.7657893
0.449218	0.9540474	0.3048615	0.8519988	0.9069675	2.9649161	0.6345003	0	1.2630344
0.3169567	0.7952682	0.4808855	0.581303	1.231801	2.4879493	0.5502112	0.1886541	0.9570974
0.3296403	0.4576468	0.9016489	0.972178	0.4908781	1.6748222	0.599413	0.1441769	0.6692989
0.8747576	0.4773665	0.4882065	0.8692397	0.3474389	2.8048177	0.6280737	0	0.6771699
0.9411063	0.3535491	0.8352773	1.0518593	0.3053285	3.0255427	0.359746	0.2886539	1.0626053
0.4847053	0.7718856	0	0.323543	0.6262055	2.1482508	0.9416322	0	0.9882666
0.4080596	0.5253673	0	0.8663937	0.7967639	1.8228525	0.5341612	0.0890913	1.1426095
0.0965317	0.082975	0	0.3840498	0.4212637	1.0529723	0.6814942	0	0.2617113
0.7919391	1.5451282	0.771125	1.963881	0.9076598	3.5824958	1.3398785	0.1802752	0.7661287
0.2465905	0.7802261	0	1.4021764	0.9168588	2.1496821	0.5731806	0	0.7395889
0.3398215	0.2952528	0	0.5249663	0.9288061	2.2829142	0.4302853	0	1.1009776
2.2605827	2.0232908	1.7971583	3.6099499	2.2837144	3.6172867	2.0629851	0.4324784	1.6220401
0.5121763	0.6533352	0	0.3430098	0.7481177	1.8947208	0.58938	0.169412	0.6892992
0.3078948	0.1834548	0	0	0.720366	0.9438088	0.0965317	0	0.2961931
0.3733976	0.4271771	0	0.5592471	0.9746027	1.4712395	0.4345615	0.0518593	0.549127
0.2798873	1.5246655	0.6014113	1.5298209	0.972178	3.7292591	1.7173858	0.8507194	1.0545012
0.0640551	0.5129853	0.6305919	0.9636221	0.4806787	2.2055176	0.1619522	0.0440443	0.5426048
0.2543522	0.3189242	0.158854	0.213254	0.4924172	1.0209822	0.3955016	0.1782366	0.5703658
0.5275708	0.8115532	0.4387187	0.2167342	1.0576929	1.7159812	0.5353563	0.138028	0.2767944
0.3538878	0.4815056	0	0.268075	0.0709388	2.8308232	0.4896457	0	0.3406192
0.1032626	0.397803	0.4421208	0.8623522	0.7545455	2.8759569	0.829606	0	1.1892236
0.6096604	1.3348543	0.9834588	1.8057877	0.7508637	3.7618278	0.6129658	0.5276709	0.9487503
0.6590112	0.4934424	0.5787938	1.138028	1.7058452	3.0250288	0.801407	0.1281614	0.9846987

0.3908878	0.7588148	0	1.3269975	1.2360333	1.5524754	0.4068632	0.2262619	0.441271
0.2349917	0.9320612	0	0.5727927	0.3929768	2.8444057	0.5490284	0.240009	0.4661311
0.4458326	1.9405802	1.1150332	1.9231492	1.3284344	4.1014064	0.7063758	0	1.3566509
0.4280352	0.3733976	0	0.491494	1.0020904	1.5688104	0.5077185	0.1708224	0.4824353
0.5227588	1.1666512	0.30381	0.5136929	0.7315308	2.8511793	0.5514914	0	0.982656
0.3063789	0.8008274	0	1.0306186	0.2806	2.2249355	0.1859934	0.2811938	0.7485471
0.2672361	0.697418	0	1.148804	0.3968171	3.9173267	0.5674481	0.2609891	0.8090028
0.6538855	1.1666512	0	1.5092905	0.6119278	3.4728776	0.6920701	0.679694	0.9164002
0.7809819	0.1363227	0.2914857	0.643025	0.3674829	0.2386646	0.2655569	0	0.7363018
1.245374	1.1883376	0.2638757	1.1899827	0.7077015	3.500904	0.5568953	0.2947825	0.4416959
0.3501577	1.1647219	0	2.0996992	0.2276179	2.5916316	0.349252	0.6186146	0.9277448
0.1272371	0.9012627	0.9192257	0.6867918	0.7249122	2.405121	1.1936456	0	2.0173518
0.3717261	0.9423831	0	1.928503	0.7137838	3.8770276	0.3292958	0	0
1.3889793	1.4119866	0.993155	2.5735442	1.9714301	3.4393705	2.3574488	0.4895429	2.0866253
0.7451082	0.7438165	0.626953	1.9468805	0.7172981	2.0978804	1.0574156	0.1068843	1.297074
0.0957219	0.6661203	0.4129441	1.8958067	0.8354391	2.4667576	0.3777346	0	0.2596642
0.2220626	0	0	1.0705954	0.7086728	1.8418524	0.5928278	0.1551016	0.9684226
0.3789555	0.5356549	0.8847933	1.6304987	1.6061105	2.0391384	0.8190959	0.3092926	0.4432884
0.344374	1.7080106	0.9015717	1.6225087	0.8141812	4.3600552	0.7701948	0.166844	0.6007455
0.7554858	1.2651368	1.1881477	1.7864291	0.4890291	3.4685703	0.3528715	0	0.935686
0.3064956	0.1825652	0	0.6054943	0.5155106	3.1137505	0.5672534	0.147437	1.3712801
1.3095255	1.8193003	0.5618882	1.9753734	0.5153088	3.9580538	1.7504779	0.418136	1.938474
0.5190386	1.3347399	0.7630716	1.8254193	0.5312692	4.5023179	1.1210154	0.3362834	1.8026482
2.620563	2.4570689	2.0476082	3.0740829	1.6826931	4.0995365	2.2995866	0.3693083	1.832701
0.6009358	1.217603	0.3214664	1.5536063	0.6176746	4.3963034	0.7451082	0.5088343	1.4760706
0	0.5732776	0	0.7035437	0.3902274	2.4407396	0.2214441	0	0
0.064469	0.5547362	0.2877087	0.6817641	0.376846	3.0483757	0.4839834	0	0.838266
0.3615438	0.5985558	0	0.9308509	0.2503553	2.5337627	0.2878269	1.0856966	1.4950811
0.428464	0.4537546	0	1.1622745	0.4107753	1.5913922	0.4214792	0.305562	0.3200803
0.2832106	0.5312692	0.1437852	0.9678326	0.3992258	2.731596	0.2268784	0.082975	0.3914378
0.4205094	0.6050202	0	1.0314658	0.634872	2.3050366	0.7220288	0.2445218	0.663208
0.1882743	0.1624679	0.153546	0.4952858	0.4641455	1.7422218	0.3374257	0.0450235	0.5533605
0.2638757	0.1746621	0	0.2881814	1.2588204	2.4018489	0.5237627	0	0.1943392
0.4623665	1.5090877	0	2.8917886	0.8187688	3.4392641	0.3049782	0.9687912	0.2080178
0.2764371	0.2668765	0.3276874	0.859413	0.587845	2.0973748	0.6347791	0.1479579	0.3823906
0.3084774	0.7421787	0.2990101	0.8381854	0.2721427	2.1452862	0.3451693	0.0461418	0.4249221
0.5326661	0.688046	0	0.6693897	1.1156992	3.220624	0.9523336	0	2.199688
0.0406821	0.3435784	0.7572793	0.5268701	0.4247071	2.7109882	0.2624332	0	0.4964112
0.5720165	1.6553976	0.2580968	2.6488796	0.878843	4.0100816	0.8866672	0	1.7358687
0.1515991	0.6922486	0.3419857	0.9375704	0.3117356	3.279189	0.2838033	0.2496274	0.3987881
0.0648829	0.2106382	0	0.4377604	0.4198625	1.6917575	0.4445611	0	0.4816089
0.7812338	0.7641763	0.2113861	2.6613392	0.9176991	2.0601166	1.153935	0.0629506	0.6445947
0.1360601	0.3263072	0	1.0632958	0.8964656	1.8827213	0.3320491	0.3447149	0.3609822

1.5541468	1.8101552	0.3251561	2.6827531	1.2435472	2.9834406	1.377457	0.3624418	1.3244074
0.5899552	1.0058311	0	1.3128394	0.8859648	2.7831633	0.52677	0.0805212	1.0627434
0.8268434	1.6807294	0.9976176	1.8984019	0.6431173	4.4872098	1.1763228	0.1900459	1.8156984
0.1834548	0.2666366	0	0.2917214	0.81295	2.0562714	0.4998841	0.0652966	0.2568899
0.3550163	0.7789655	0	0.3867006	0.905659	1.8906023	1.0246034	0	0.486354
0.1791288	0.5382402	0	1.0139267	0.6226493	1.7263953	0.7084963	0.3453964	1.0973411
0.1800205	0.4232017	0	0.3703875	1.1567849	2.6815617	0.7114068	0.1251221	0.8185234
0.3577209	1.1396659	0.2028878	0.8137709	0.6895675	2.4570426	0.5496199	0.4687397	0.5353563
0.3271125	0.9850632	0.2689134	1.6116918	0.6361722	3.232231	0.6369146	0.429964	0.7919391
0.064469	0.2574934	0	0.2647166	0.8448677	2.1278313	0.4423331	0.2473199	0.1779816
0.9690123	1.63157	1.1086248	2.3840498	0.3709454	3.4273514	0.8343876	0.5752155	1.1401242
0.4302853	0.7677395	0	0.8761731	0.7844202	2.3702759	0.6333847	0.4273916	0.5400273
0.3829439	0.5482393	0.4075159	0.920903	0.7955176	1.5691022	0.7963486	0.4033768	0.5709486
0.0440443	0.0377336	0	0	0.6848187	0.1638851	0.3142905	0	0.4397827
0.1640139	0.4976379	0.255682	1.0435545	0.9557592	1.7386382	0.3951725	0	0.6569082
0.6429326	0.3498182	0	0.9136458	0.7268312	2.790063	0.7652801	0	1.1061473
0.768756	1.3695502	0	1.7514637	0.4033768	3.8161594	0.7191396	0.2655569	1.6457478
0.5476472	1.186247	1.2463473	1.1712709	0.6421932	3.1693478	0.5808208	0.2555612	1.3967075
0.541614	1.1170301	0	1.3820585	1.064469	2.2902474	0.4828483	0.3203114	1.362554
0.0681893	0.2713061	0	0.4003193	0.4528063	1.3234277	0.3733976	0	0
0.5049763	0.6590112	0.2087669	1.3972006	0.4152178	2.2867925	0.2249046	0.0621217	1.1998137
0.6168282	0.7343953	0	1.5739077	0.3228511	2.1339251	0.1992483	0.0469799	0.8768018
0.2953704	0.4736311	0	0.3222743	0.579856	1.5773926	0.1998765	0	0.2180992
0.3561438	0.2382977	0	0	1.2690331	2.9623084	1.1111649	0.2521734	0.4878979
0.7131679	1.0018743	0.3304437	1.1963552	0.4916992	2.9297531	0.982729	0.3682655	1.5805797
0.3519675	0.8561491	0.817787	2.5535571	0.9278965	2.6346165	0.6648469	0.2814312	1.2395813
0.5908176	1.5514422	0.8091675	2.619906	1.0863082	3.1715591	0.860049	1.0442542	0.640991
0.4731115	0.9968949	0.2270017	1.0076261	0.4487953	3.903983	0.3527585	0.3111543	1.009992
0.3881344	1.6069633	0.640806	1.8623125	0.5060941	5.0004102	0.7426099	0.144438	1.6355687
0.0958569	0.393526	0	0.6833821	0.6543439	0.9844071	0.3782897	0.3924273	0.729966
0.3118518	1.1288213	0	1.3000651	0.8648101	2.1741827	0.9219694	0.2198345	1.1465249
0.1017845	0.1700532	0	0.2154922	0.9378717	1.7097759	0.0892269	0	0.2752455
0.2043912	0.2572521	0.2434254	0.8110599	1.1109645	3.2127094	1.2792336	0	1.8134426
0.2235461	0.6864333	0.6846392	1.2540498	0.2693923	2.9695465	0.3287216	0.3627785	1.769264
0.4915966	0.5024324	0.4773665	0.9218933	0.7716321	2.0538064	0.5809173	0.5291713	0.5525738
1.0114956	1.7184822	0.5322671	1.2778658	0.7689253	2.5012353	0.8526381	0	1.2186572
0.8699503	1.8519988	0.9996393	1.5546871	0.6445024	4.3701085	1.5559634	0.3587338	1.0289934
0.3627785	0.2577348	0.3518544	0.704872	0.6127771	1.8733801	0.1372412	0	2.2065182
0.4383993	0.3614315	0.514804	0.9480027	0.5688104	1.4902107	0.7463988	0.2033891	0.7668919
0.2776277	0.5694911	0.2276179	0.8313909	1.0009374	2.6679148	1.2561049	0.82546	1.1504297
0.9796596	0.5534588	0.7937711	1.3444877	0.8161491	2.6835393	0.8858867	0.2716647	0.9053509
0.3393655	0.4527009	0.2142492	0.6639366	0.325041	2.2424806	0.2659169	0	0.7371676
0.342896	0.1777265	0.2450088	0.4198625	0.7012385	1.4778845	0.4160831	0	0.6329196

0.2882995	0.9602893	0.3415304	1.4031586	1.1499747	2.5433969	0.5201456	0.1545832	0.6380275
0.0321008	0.2068932	0	0.3199647	0.744075	2.4025312	0.2824991	0.0436245	0.2561653
0.0440443	0.9470302	0.2012575	1.7023029	0.4905701	2.9522031	0.6236796	0	0.9855004
0.5863084	1.1379625	1.0910566	0.4523846	1.0394894	3.2380378	0.5234616	0.3489122	1.2875314
0.2331518	0.7313571	0	0.4715517	1.0626744	3.652865	0.6331057	0.1935825	0.9317587
0.3865903	1.47892	0.3750672	0.9621418	0.5543433	2.8488983	0.3426685	0	0.6135317
1.3953919	2.4739168	0	2.2727996	0.7233401	4.2904686	0.6498454	0.1994996	1.3573268
0.3862591	0.7762725	0	0	0.7992531	1.9615122	0.6890307	0.1631122	0.8125393
0.5392333	1.2067057	0.3490255	1.458172	0.486354	4.6165988	0.7586443	0.2073931	1.2032012
0.2836848	0.3547906	0	1.0822938	1.0733404	2.0334054	0.5903385	0.0695647	0.9537495
0.5908176	0.2826177	0.384713	0.1882743	0.6332917	1.3066122	0.412619	0	1.2954879
0.3876934	0.9573203	0	1.2380531	0.4685312	2.9064481	0.5470549	0.2391536	1.403595
0.1701815	0.6922486	0	0.8701082	0.5957901	1.9241759	0.8193412	0.1182268	1.3388522
0.2102642	1.0330882	0.2035144	1.0745739	1.0538064	1.4992209	0.2529	0.1184926	1.1777903
0.0688771	0.8094144	0.3057955	0.5169229	0.7125518	1.7937294	0.8561491	0	0.7676548
0.6959048	0.7499204	0	0.7510352	1.2027625	1.6148983	1.6075315	0.1645289	0.5936884
0.1286893	0.7811499	0.5369481	0.8660771	0.7536045	2.4672012	0.3153344	0.0890913	0.342896
0.660381	0.9426083	0.8488783	0.7915224	0.7460547	2.1830736	0.4454089	0.1545832	2.0349904
0.0988238	0.313362	0	0	0.4302853	0.6328266	0.3190398	0	0.6884042
0.1672295	0.2753647	0	0	0.4521737	0.7788814	0.6193661	0	0.6134374
0.3142905	0.9199883	0.417812	1.1543888	1.5881329	4.7063869	0.1651723	0.3070787	1.0698396
0.6015064	0.1352722	0.3534362	0.1719754	0.7011498	1.8787253	1.4084401	0.1088924	0.7317915
0.474462	0.7836662	0.9564284	1.0007212	0.4364817	2.8676198	0.2945472	0.1223414	1.317362
0.0710762	0.2822619	0	0.2900705	0.2541102	1.3052118	0.1219437	0	1.206018
0.2784606	1.275007	0.269871	2.2486866	0.6804144	2.9281239	0.8616378	0.1585956	0.1665869
0	0.0931545	0	0.2287264	0.2262619	1.4934936	0.3470987	0.0747794	0.8351965
1.1050075	1.1071522	0	1.7861363	0.7018595	3.0158008	0.5701714	0.2172307	0.5423076
0	0.1878944	0	0.779722	1.5269202	2.7405175	0.3997727	0.9583602	0
0.5239633	0.9990619	0	1.770829	0.9365155	2.3923724	1.5091384	0.3770682	0.2747686
0.8410875	1.2349304	0.9676851	2.1891604	0.516822	3.2401311	1.2858756	0.7046949	1.0957219
3.2540876	2.488335	2.6274434	3.9695788	1.5988892	4.5787093	2.5481407	0.7987556	1.4652693
0.0766959	0.2469553	0	0.78375	1.2962518	1.5394814	0.362554	0.1036655	0.5564049
0.0847446	0.8288753	0	0.6167341	0.7864291	2.4498783	0.1448295	0.2204538	0.6057788
0.2056427	0.1352722	0.2450088	0.2250281	0.2226809	0.673466	0.3418719	0	0
0.3987881	0.8301742	0.2865263	0.2635152	0.7385517	2.571337	0.6372856	0.3198491	0.725523
0.1510795	0.1910572	0.6164518	0.1655583	1.3056204	0.7159812	0.1326426	0	0
0.3953919	0.2385423	0	0.7612002	0.7542034	1.2799467	0.7550585	0.0999011	0.2030132
0.4462562	0.9486008	0	0.529771	0.9903014	2.2486259	0.4714476	0.4694693	0.5489298
0.4623665	0.2995963	0	0.5021269	0.9006447	0.8782937	0.3322783	0.1982427	0.2080178
0.1067503	0.3854863	0	0.645056	0.7386382	1.7586443	0.7775354	0.0494914	0.4517518
0.7196654	1.0747794	0	1.5363016	0.683921	3.6846392	0.834873	0.4256741	1.5667178
0.4816089	1.156073	0.5642318	1.064538	0.7891867	3.459366	0.8985566	0.4910835	1.5113162
0.8145914	1.1307991	0.3846025	0.1881477	1.4980466	2.5019231	0.6841904	0.2295879	0.9325904

0.3500446	0.5165195	0	0.6825733	0.6304987	1.0467704	0.7125518	0.4590119	0.7290959
0.2065182	0.3371973	0	1.892663	1.033793	2.1969532	0.555718	0	0.5283713
0.6393248	0.3476657	0.4696776	2.1159987	1.0290641	2.0329119	0.8748363	0.148869	1.1007086
0.2953704	0.3689359	0	1.0882773	0.3886855	2.4930837	0.3754009	0.2078929	0.8608436
0.2696317	0.8263553	0.2612299	0.3466449	0.5774409	2.1729995	0.2378084	0	0.7728147
0.186247	0.5571895	0	0.8556708	0.7225535	2.0111736	0.263395	0	1.0398403
0.5221562	0.533663	0	0.6568167	0.4140273	3.078883	0.5425057	0.5616927	1.0019463
0.4385058	0.3827226	0	1.6744155	1.1620811	2.9297721	0.4561753	0	0
0.3690476	0.2722621	0	1.0121396	0.7090259	1.5290213	0.70982	0.2211966	0.7639214
0.5886127	0.1935825	0.4911861	0.9450083	1.0366792	2.8467942	0.4502743	0.1565261	0.3118518
0.4936473	0.5280712	0	0.9701174	0.5740531	1.5364011	0.8722378	0.1522483	0.3925372
0.7878503	2.1860568	0.8090851	0.9371937	0.9291092	4.4160358	0.9300939	0.1910572	1.7597524
1.3434647	1.3061456	0	1.2359108	1.2260769	2.9421203	1.0139267	0.3238888	1.2714854
0.8008274	1.1413674	0.4420146	0.7260465	1.091666	3.225676	0.9307752	0.3826119	0
0.705049	1.5801937	0	2.1779497	1.0020904	2.8119231	0.3261922	0.2611095	0.1881477
0.4137024	0.5897635	0.2367069	0.4922121	0.548042	3.1504784	0.3670355	0.2028878	0.886355
0.2484134	0.2149951	0	0.6342215	0.595981	1.5584636	0.6790634	0.1183597	0.2387869
0.7218538	1.3532668	0	0.8181143	0.5101518	3.5757238	1.0928163	0.2353594	0.9037324
0.804384	1.7156738	0.4641455	0.6833821	0.6961719	3.8346809	1.5623279	0.2151194	0.537644
0.2442782	0.3518544	0.2901885	0.8585381	0.8509594	0.9225023	0.4459385	0	1.1330374
0.7342219	0.6022668	0	0.9183862	1.455965	1.9679064	0.2028878	0.1092937	0.7340484
0.0430645	0.7658742	0	0.4159749	0.4986594	3.4912246	0.4547023	0.1147668	0.6070107
0.628447	0.951364	0.4912887	1.9344033	0.8480771	2.6361258	0.5618882	0.5442875	0.6811344
0.6490176	0.8482373	0	1.3896219	0.5764738	2.5035268	0.4636225	0.2963106	1.1060803
0.0947766	0.3014706	0.4094724	0.2012575	0.636729	1.6368682	0.3743996	0.1278974	0
0	0.6495695	0.6635723	0.4850146	0.5137939	2.5388113	0.4533332	0.0582472	1.2035771
0.3456235	0.658737	0	0.533364	0.7015047	2.0538759	1.4074615	0.1273691	0.6630258
0.3590713	1.0450934	0.6286336	0.6983963	1.8905634	2.8803525	0.3738431	0.1071522	0.9815604
0.2133784	0.347779	0	0.1596292	0.4904674	1.6145214	0.1877677	0.1952216	0.5439907
2.9911	1.8571184	1.3203884	3.6331948	2.4099611	4.0926698	2.4378935	1.106594	1.1667797
0.9129564	1.6255043	0	2.1764504	1.100574	3.5573121	1.0955194	0.6004601	0.681854
0.7004397	0.6991962	0.588037	1.1027925	1.0149265	2.3345683	0.7102609	0.191689	1.6302657
0.3533233	0.82489	0.4170559	1.0498398	0.8627489	3.2250281	0.6460705	0.3606452	0.7917724
0.3064956	0.8009103	0.4658178	0.7616257	0.4715517	2.1273361	0.6328266	0	0.2947825
0.4423331	1.6543897	0.5803385	2.1336293	1.1374379	4.0634425	1.3077199	0.097476	0.5279711
0.6231177	1.0118534	0	2.6411067	0.3719491	3.6718821	0.5101518	0.102591	0.5516883
0.2003788	0.3275724	0	0.4096896	1.0578315	0.9739418	0.7598376	0	0
0.0909889	0.6383056	0.3942946	0.3637878	0.6148512	1.5950262	0.6486495	0	1.4239546
0.6165459	1.2436082	0.5541468	1.547203	0.5863084	4.1494626	0.6707497	0	1.6909981
0.4537546	1.4533332	0.2906603	0.7761882	0.5387368	3.7022475	0.7869309	0.4627853	1.9712216
0.2246577	0.1942132	0	0.455439	0.6182387	1.3978577	0.6446869	0	0.5696855
0.6461627	1.3277448	0	1.9844071	0.6213369	3.0709217	0.9897203	0.9008765	1.0107082
0.3788445	1.484396	0.446468	3.4928658	1.2067057	3.100759	1.2874723	0.4953882	1.6589655

0.3680419	0.4571215	0.1895399	1.0175656	0.6933197	2.6557869	0.6856262	0.1100959	1.8435417
2.3313995	2.6403433	2.0912259	3.209812	2.2517898	4.2724363	2.0572307	0.9425333	2.4999947
0.3524196	0.7739963	0.7384652	1.6197886	0.7850902	2.9774265	0.5446832	0.249506	1.2819059
2.372381	2.522106	2.179734	3.0604798	1.8003098	6.1810051	2.2590314	1.1069848	2.6703418
1.0134265	1.7943119	0.2690331	1.6124469	1.1079556	3.1009945	0.7368213	0.3005338	1.0689459
0.7321389	0.8466337	0	1.0800436	1.1140338	1.8944104	0.776104	0	0.2910141
0	1.2641761	0.9362893	2.161275	0.8938281	3.3985282	0.5801455	0.3311319	0.6262989
0	0.4151096	0	1.1944023	0.5103543	1.5319179	0.5946441	0.1801479	0
0.3193867	0.7470006	0.3096419	1.275007	0.5447821	2.219184	1.1724875	0	0.1921942
0.4954905	0	0	0.3311319	0.2905424	0.5406225	0.2681948	0.1108977	0
0.9751899	0.3643483	0	0.3534362	0.3754009	0.9841883	0.1504297	0	0.568324
0.4357353	0.9007219	0.2784606	2.1995624	0.4687397	2.7325731	0.4286784	0.0842004	0.915865
0.3777346	0.7261337	0	0.7308356	0.7707868	2.6832248	0.9689386	0.1840898	0.2524157
0	0.4354152	0.3609822	0.1759397	0.899253	0.753091	0.5977934	0	0
0.4617381	1.0155689	0	1.3357119	1.0434145	2.828266	0.5248661	0.3303289	0.9286546
0.4743582	0.1804025	0.6453328	0.4255667	1.1288213	2.5449304	0.4931349	0.0746424	0.9221978
0.3568199	0.8931292	0	1.1614363	0.7075248	3.0557336	0.7350889	0.348799	0.5468574
0.5628652	0.9651748	0.3289513	1.1166309	0.7093789	2.2343174	0.9098883	0.1007086	1.5001902
0.5578757	0.7114949	0.1634987	0.6801443	0.6980406	2.5888525	0.4061012	0.0946415	0.7774513
0.8329305	1.6084308	1.8140171	2.224349	0.9739418	3.0139267	0.7498346	0.2788174	0.8061178
0.0640551	0.158854	1.2109499	0.9257716	1.2824991	1.717956	0.3968171	0	1.6855365
0.9391513	1.2001277	0.2270017	1.5576797	0.8987114	2.921284	0.798009	0.4699901	1.3340533
0.6175806	0.3836075	0	0.9378717	0.5385382	2.1561053	1.2620121	0	0.2272482
0.5857317	0.7990044	0.2867629	2.6820788	1.5155611	1.4686354	0.7443333	0.0869196	0.9380976
0.3729521	0.623586	0.8594925	1.0803166	0.6955485	3.1194226	1.0211243	0.3645724	1.7231216
0.0313952	0.6111724	0.5116704	1.2964868	0.9437338	2.1990912	0.5087329	0.2391536	0.5292712
0.2751263	0.23842	0	0.3004166	0.4459385	0.7482036	0.5441886	0	0.1382902
0.1202192	0.4944668	0	1.1035983	0.3868109	3.1307991	0.5408208	0	0.9061979
0.3788445	0.9251639	0	1.4955928	0.6527848	3.2406654	0.3357119	0.4953882	0.7201033
0.3381106	0.5378427	0.3994446	1.1137672	0.5532622	1.3438058	0.2991274	0	0.6502131
0.4124022	0.9141818	0.8474357	0.9347807	0.5138949	2.9829115	0.5191393	0.0791562	0.9643617
0.6567252	1.3568762	0.3011194	1.3717819	0.9177754	3.1730475	0.5469562	0.5447821	0
0.4312485	0.2311866	0	0.7134319	0.6927843	1.598651	0.1392075	0.1099623	0.2226809
0.5695883	0.529671	0	1.2914857	0.8405237	1.5258183	0.5082258	0.305562	0.5819778
0	0.1582077	0	0.376846	0.7021256	1.5062973	0.4993739	0	0.2566484
0.2564069	0.1695402	0	0.4020673	0.7999991	1.2148708	0.7472585	0	0.5050779
0.3882447	0.5481407	0	0.422556	0.2752455	1.8866282	0.1823109	0	0.2891262
0.3231971	0.3731749	0.6838312	1.1665226	0.1407787	2.6412454	0.1851054	0.0604625	1.3914928
0.567156	1.7954761	0.7637515	1.782744	0.3946239	3.6544241	1.0059748	0.0918014	1.1671652
0.5025343	1.1844073	0.3645724	1.7488905	0.8561491	3.4511056	0.3329657	0.7222037	0.8917302
0.7902716	1.6705684	0.2579761	2.2206705	1.057277	3.1736392	0.741316	0.3520805	1.3299847
0.3552418	1.1617587	0.4518573	2.8607443	0.7459687	1.2506585	0.7913557	0.1426095	0.7902716
0.2784606	0.8489584	0.269871	0.7284865	0.963844	3.1051417	0.531868	0	0.8927407

0.4312485	0.6743703	0.3567073	0.5962673	0.6498454	2.1313919	0.7733212	0	0.5856356
0.23842	0.4691566	0	1.1296787	0.4249221	0.7635815	0	0	0.3314759
0.0742314	0.4447731	0	0.1587248	0.6014113	1.8733408	0.4523846	0.1940871	0.6841904
1.1125	0.7554004	0	1.1256511	0.9077367	1.7779983	0.628447	0.2424501	0.7157177
0.2401311	0.6606549	0.1496496	0.4321042	0.7676548	2.2829438	0.839879	0.3185772	1.0745054
0.3755121	0.9257716	0.1512094	1.8958067	0.6759062	2.7593689	0.544881	0.1696685	0.4099068
0.7456246	0.5361524	0	0.5820742	0.5764738	2.0740601	0.5450788	0.2692726	0.518233
0.6462548	0.3673711	0.628447	1.166844	0.7977601	1.675274	0.4277134	0	0.857583
0.242694	0.6062527	0	0.6213369	1.6297063	0.5599323	0.8038883	0.22231	0.767231
0.5363514	1.0374525	0.6599246	0.5489298	0.6069159	2.6954148	0.2744108	0.1672295	0.6815842
0.2362171	0.2838033	0.167101	0.7416611	1.6337567	1.8340639	0.4844991	0.0492125	0.2858165
0.1496496	0.6849085	0.3379965	1.4254593	0.7002621	2.6563362	0.25181	0.6058736	0.5569934
1.0100637	1.0005049	0	1.614757	0.6342215	3.4585264	1.3967623	0.1458731	2.2663668
0.82546	1.5294212	0.8473556	1.7784189	0.7575354	3.7228704	0.6127771	0.2936059	1.4560176
0.2190911	0.3567073	0.3379965	0.6800542	0.7649406	1.487332	0.4155424	0	0.1090262
0.6542522	1.8808031	0.9441088	1.74859	0.9383987	4.5771629	0.9518862	0.3929768	0.9104257
0.144699	0.6740086	0	1.0491428	1.4081684	2.4607164	0.372395	0.1939609	0.5104556
0.703898	1.93104	0	2.6376798	0.6351507	3.1517939	1.153027	0.2606279	1.2571917
0.2246577	0.3422134	0.1587248	0.2776277	0.6807744	0.9521099	0.440527	0.0918014	0.6356151
0.0870554	0.9294123	0	1.1595	1.2764967	1.1424788	0.4645637	0.1175621	0.7783768
0.7352622	0.4425455	0	0.8244827	0.7035437	3.8251852	1.0483757	0.1315895	1.2675957
0.127105	0.9004901	0	1.3917677	1.0022344	2.4980466	0.8508794	0	0.6130601
0.1660727	0.6541605	0.3725064	0.1819294	0.9231872	1.5329652	0.5115692	0	0.7663831
0.3258469	0.9553128	0	0.8027309	0.5457707	2.3500163	0.7338749	0.0807941	0.8871353
0.2463473	0.399007	0	1.2436691	1.0340044	2.6558326	0.2172307	0.4209405	0
0.3189242	1.6000318	0.7800581	1.9797693	1.3423841	4.3776999	0.9632522	0.1826923	1.1890338
0.2815499	0.4527009	0	0.8410875	0.9255438	2.0240714	0.5246655	0.2582174	0.8684497

KDM4A-A!	RPARP-AS	AC012409.4	AL354989.1	AL137009.1	AC024060.2
2.0020544	3.3456518	0	3.1866907	0.9186152	4.1658878
1.5414653	2.245161	0.3605328	1.3552418	0.3034593	3.4985572
1.2290341	1.7867218	0	1.5560124	0.0482362	2.3896494
1.5056369	3.681899	0	2.0043935	0.9547919	3.6071291
1.1615653	3.7870876	0	1.0588706	1.753904	2.5824837
1.8990209	3.5698313	0.8137709	1.587797	1.587221	4.4411315
1.2532027	3.5037431	0	0.5306701	0.0729976	4.7500276
0.1064823	1.7100846	0	0.3268824	0.2365845	2.1655904
0.9156356	2.5100251	0	1.9794035	1.866275	3.0708015
1.722772	2.9493108	0	1.9819987	1.8713311	3.9152054
0.8893957	2.3865351	0	1.2190291	2.0306892	3.0490731
0.4898512	1.803723	0	0	0.2487777	2.3622735
0.3263072	3.3958032	0.3089433	0.1290851	1.058455	4.3824321
0.6736469	3.9448022	0	0.5171245	0.3240041	2.4171369
1.5771025	2.949703	0	2.2744108	1.3373115	3.2326915
0.8105665	2.4859162	1.045443	1.7502635	0.7080548	3.9355823
0.9588798	2.980921	0.2856981	1.13888	0.9949417	2.9713872
0.9257716	3.636729	0.7870981	0.8902521	1.8648101	2.9227687
0.7747554	2.5525492	0.7868472	0.295723	0.6581886	3.9723434
1.8790392	2.6368218	0.3148705	0.9323636	1.2370742	3.5998296
0.5284713	3.6689247	0	0.8792352	0.9126499	2.9602337
0.9976898	3.0987564	0	0.7497488	1.0168528	4.0889811
1.124262	2.4057746	0	1.0958569	0.5725987	3.026446
1.4411116	3.349804	0.2263852	1.2624332	1.1915627	3.369299
1.1791288	2.3224762	0.6380275	1.1371756	0.6883147	2.9501698
0.3422134	2.5128084	0	0.4041402	0.3913278	1.8046318
2.0549179	3.4886178	0.9039638	1.3105728	2.6571369	4.3028381
1.5846258	4.1276745	0.3533233	1.9371937	0.4257815	3.7793123
0.9163238	1.9960995	0	0.696261	0.3467584	3.1000525
2.6653473	3.2671762	2.8524783	3.2782896	3.0247718	4.7472021
0.8244827	2.7541392	0	1.4738389	0.6072001	2.5011334
0.1526377	2.5197432	0.5580717	0	0.3329657	1.8554715
0.8949147	2.1837723	0.2141248	1.2124448	0.8932069	2.1593386
1.6622511	3.1663941	0	1.8582198	1.6539313	4.1727835
0.676809	1.5313191	0.1829465	1.4865084	0.7387246	2.6429788
0.8656813	2.7312267	0	1.0094905	0.520045	2.8525582
0.9689386	3.503972	0	1.139404	0.928503	3.642251
0.217727	2.2403143	0	0	0.4637271	1.0790196
0.6061579	4.0076799	0	0.9006447	2.1183265	3.8452592
1.6932305	3.806654	0.3115031	1.7493626	1.931494	3.8439838
1.2125693	2.8195456	0	0.5629628	0.3546778	3.6733529

1.1962923	2.9418012	0	0.6612936	0.860049	3.4218427
0.9038867	2.6090457	0.3374257	0.6958157	0.9570231	3.7243119
2.2369212	4.0609553	0	2.2216606	1.6433021	4.3873488
1.0029545	2.5430999	0.349705	0.7647708	0.8712128	2.2214441
1.252234	3.2484741	0.6580058	1.3063206	0.8433407	2.9984663
0.6622967	2.4989657	0	0.4525954	0.403595	2.9759053
0.4841897	2.7881845	0.230449	0.9128031	1.0666748	2.8473756
1.4608998	3.1452536	0.3365119	1.5130864	1.7734478	4.2685167
0.3981314	3.9182717	0.3516283	0.3455099	2.3002702	2.6838536
1.6133902	3.155474	0	1.0476082	0.8606848	4.4805107
1.044534	2.77178	0	1.3024653	0.7158055	3.8273414
1.7861363	3.4289463	0	0.9776829	2.1114988	3.3611507
1.4988125	3.8358534	0.6631169	2.4188376	1.2406807	3.889824
2.389943	3.1296677	3.0595456	2.5964184	2.4005561	4.5913124
0.8578218	3.9465437	0	0.8395565	1.0429945	3.2152748
0.9921883	2.154486	0	2.0159257	1.4246534	2.9242709
0.402613	2.0375579	0.5808208	0.9795132	0.1842168	1.9061979
1.1529622	2.4536229	0.2005044	1.3988976	1.2465297	3.5262441
1.2761989	2.6677105	0.3420996	1.3039268	1.2614105	4.0335287
1.4096896	3.8006825	0	1.9424207	1.2014457	4.4888749
0.8065303	2.5325414	0	0.2439128	0.9770234	3.4208192
2.0303714	3.3682236	0.666393	1.955164	2.9814143	4.390537
1.3349115	3.4991953	0	1.9712216	0.8057877	4.1262543
2.061396	2.4973058	1.0000962	2.785432	3.0397233	4.4877543
1.2841587	2.9915355	0	1.9165913	0.9488251	4.223577
0.679694	1.3797318	1.0925457	1.0773797	0.6982185	2.8060765
0.73014	2.7508423	0.8610819	0.6169222	1.7305313	2.89664
0.9930584	2.3925098	0	1.5096958	0.1756843	3.5986034
0.5718224	2.2871768	0.3304437	0.6831125	0.5104556	1.4258352
0.8240754	1.8710551	0.175301	0.6389543	0.0254541	3.4967435
1.4529644	2.6877102	0.4886178	2.6724027	0.7353489	3.1903462
0.8355199	1.9832399	0.3525326	0.4645637	0.0538064	3.6125058
0.4913914	1.6988852	0	0.6652108	0.5377433	2.340961
1.9648791	2.7644311	0	2.0534589	0.8790784	3.236462
0.6894781	2.3645164	0	0.8491186	0.3812834	2.3785116
0.6352436	2.0472243	0	0.1522483	0.4131608	2.5870769
1.1333005	3.3077491	0	1.6256913	1.0167816	3.1335306
0.587365	2.1212807	0.4226636	0.3414165	0.1596292	3.1103632
2.9821813	3.9565957	0	1.5795181	0.9726191	3.5942261
0.6727421	2.0840983	0	1.2376249	0.3470987	3.5308074
0.1650436	2.4990167	0.1851054	0	0.2936059	1.6815392
2.0522072	3.4863282	0.8304176	1.0482362	1.2890081	3.7707551
0.7617959	3.2334586	0	0.65764	0.165301	3.2645965

2.0586628	3.4992081	0	2.1449274	0.7892702	3.9204362
1.8795882	3.1248079	0	1.1241958	0.8139351	3.3893603
2.1048734	4.3919052	0.3864799	3.3270118	1.6567709	3.5465982
0.7685019	1.4142438	0.2653169	0.7762725	0.4735272	1.9062364
0.3993352	3.2550928	0	0.9836047	0.4249221	3.5197558
0.5507037	2.7904384	0	1.0568609	1.4775737	2.7973658
0.8244827	1.9193782	0	1.9809393	0.98448	2.803723
1.0775848	2.9262082	0	1.9439588	0.2990101	3.9144306
1.0932221	2.1002376	0	1.8774694	1.7184822	3.309249
1.3187507	2.643279	0	0.8904856	0.4971269	3.1406642
1.8602875	3.3017047	0	0.9843341	1.0204844	4.8503694
0.9593992	2.4582245	0	0.9703383	1.276318	3.7671357
0.6400656	3.391094	0	1.1777903	0.7580473	2.9624935
0.2584586	2.2737546	0	0.1950956	0.2358496	1.4195389
0.7341351	3.0061544	0	1.0222613	0.1781091	3.2378696
1.1823109	1.5419113	0.9675375	0.6433945	0.2593026	2.6548021
1.6904171	2.9658581	0.2875905	1.7379463	1.1653653	4.248975
1.4910321	3.5234365	0	1.317825	1.2775087	4.2247658
1.3069038	3.2262619	0	1.8293625	1.3170725	3.2859792
0.3279173	1.51783	0	0.6460705	0.5216538	1.9105408
0.8610025	3.0883112	0	1.1889705	1.4959509	3.5175277
0.948227	3.792366	0.1945914	1.7072155	1.1701173	3.8547537
0.5842891	2.0701489	0.4202937	0.614003	0.4064278	2.3956661
0.988921	3.9297721	0	0.861876	0.3633393	2.6923156
1.5417131	3.5049635	0.3976935	0.8046318	1.7833309	3.8989049
2.1513718	4.7929386	0.9562797	2.1410403	1.5707544	3.9390291
1.409581	3.212009	0.3008851	2.3986787	1.4936986	3.7428901
0.930321	1.7706599	0	1.4490066	0.3659165	3.0544318
1.5627187	3.0932052	0.2984237	1.1773438	0.9847716	3.5993892
1.1112985	3.6034186	0	0.514097	0.2927817	3.0516679
0.9353089	3.2780145	0	1.5717253	1.3110962	3.7545669
0.6703872	2.8188506	0	0.7511209	0.3096419	2.0261981
1.0498398	3.2268476	0	0.4387187	0.3911078	2.6964613
1.2362171	2.8769196	0.9987732	1.2450088	0.3498182	3.4513298
2.1273691	3.6408407	0.5691022	1.2186572	0.2621926	3.7734056
1.6525553	3.7967639	1.0705954	2.9824004	1.4131066	4.7285029
1.5589533	2.3066997	0.6925165	2.0913952	0.5092398	4.3401991
0.7516351	2.1510795	0	0.6174866	0.4088205	2.49439
0.9359877	2.7930011	0	1.198997	1.0380849	3.0695819
1.2437909	2.8097437	0.8817429	2.3117647	0.549127	3.928664
1.2103265	3.6525783	0.2398868	1.8293219	0.7622212	3.6841455
0.7749241	1.8645725	0	1.0046093	0.1129003	2.6029557
0.3595212	2.3442888	0	0.9221978	0.3229665	2.2270325

1.8463128	3.436988	0.2198345	1.2362171	0.5159143	3.9172981
0.5456719	2.1593708	0	0.3959402	0.1027253	1.9417449
1.1369788	3.6134256	0	1.5272205	0.8575033	3.9296111
0.9299425	2.6652791	0	1.900065	0.7750927	3.0223323
0.9077367	2.7354139	0.7021256	0.727267	1.0839962	3.2527638
0.8821343	2.4552023	0	1.0035303	0.8111422	3.1889389
1.7363451	4.323788	1.1984313	2.2654669	1.5201959	5.1534608
0.6933197	2.4281692	0.834873	0.4953882	0.2381754	2.487615
1.5022287	3.0285868	1.0087737	1.7576207	0.6805044	4.0121932
0.9390008	2.87401	0.2817873	1.181993	0.9179282	3.0315364
0.8308232	2.623703	0	1.0245325	1.6187555	2.6153928
1.1121663	2.1628545	0.3360548	1.1598875	0.7215039	3.6744494
0.7600932	2.2281414	0	0.8513593	0.8226078	2.0026305
0.7707022	2.6764028	0	0.852718	0.1070183	2.1949696
0.7696025	1.8855744	0	0.296663	0.3562565	2.8108132
0.4250295	2.5273957	0	0.949124	0.4072984	2.6893663
1.1994996	2.5795422	0	1.5124797	0.5060941	3.0009735
1.2975436	3.0332997	0	0.8274939	1.9702279	3.038998
0.484396	1.7972621	0.5083272	0.4127273	0.6602898	2.4517518
0.3572705	2.3791219	0	0	1.0400508	2.4886692
1.7589001	2.8898823	0	1.299655	0.2959581	5.0372285
0.754631	4.1791846	0	0.7407981	0	3.1597422
0.9865202	2.2760499	0	0.6837414	0.4560702	3.2879155
0.6976849	2.615275	0.202261	0.3056787	0.0585243	2.0912936
1.5951217	2.5198438	0	1.3169567	1.0567222	3.9368169
0.2860531	1.8588961	0	0	0.0453032	2.015105
1.4419615	2.9445586	0	1.9877938	0.6934981	4.473092
1.1569143	2.0539107	0	0.9632522	0.1372412	2.1827241
0.9632522	2.809291	1.2042033	2.4414304	3.0818168	3.4115347
1.7803941	3.7130359	0.2586998	2.133695	1.1469158	4.5769151
3.3463542	3.2567616	0.6371929	2.9083614	2.7656196	4.3174271
1.0924781	1.7164203	0.7225535	1.6037866	0.1234013	2.0401911
0.8030617	1.9792571	0	0.6464392	0.6729231	2.2675358
0.6837414	3.4735791	0	0.6199294	0.4274989	1.7225097
1.0169241	2.8252972	0.3458505	0.5107594	2.3488839	3.0656585
0.6440408	2.1245928	0	0.5997939	0.6247092	1.868015
0.6191782	2.9949236	0	0.986957	0.8617966	2.9606044
1.562914	2.5995796	0.3102238	1.5266197	0.8408459	2.8165997
0.5786972	2.1994682	0	1.0506758	0.3642362	1.8450685
0.9528554	3.4146903	0.3838287	0.5636463	0.8612408	2.7571086
1.4955928	3.178635	0.715542	2.007662	1.1979283	4.1118576
1.3702759	3.4316764	0.3729521	1.1842803	1.6547105	3.8611316
1.2092038	2.5081751	0.8100729	2.1364211	0.9105792	3.3912041

0.5839041	1.6696164	0	0.7147511	0.1536757	2.1927307
1.1666512	3.1283264	0	2.4096353	0.5269702	2.4301247
0.9652487	2.9929315	0	1.773279	0.2577348	3.0309716
0.9989176	3.1935037	0.4202937	1.2180372	0.681854	3.8547936
0.5905302	2.4803427	0	0.3650206	0.6523259	2.9375892
1.0912598	2.8574436	0	0.8080143	1.2113861	2.7636028
1.3927021	1.9071983	0.6035967	1.0132836	0.5846739	3.5846739
0.9444837	2.757962	0	1.2187812	0.859254	2.8872913
1.2532027	3.0868517	0	1.204579	0.3765127	4.1093606
0.8693977	1.6705684	0	0.258338	0.632082	2.9646574
1.0964643	3.7465493	0.2166101	1.18301	0.9851361	2.8973953
1.6059684	3.8610124	0.3885753	1.4946716	1.2893033	4.3989591
1.6513158	2.7569806	0	1.5196929	1.2382366	3.9275836
1.4448791	2.487795	0	0.7597524	0.6846392	4.0718399
1.2720232	3.021302	0	1.6030269	1.1310626	3.5207994
0.4608998	1.8913025	0	0.4272843	0.3122005	2.8487581
0.4118601	2.2170446	0	0.9089666	0.822934	1.5262692
1.6537479	3.6287503	0	2.1496821	1.4141897	4.4457399
0.975997	4.0947934	0.5537537	1.3559184	1.924898	3.7035991
1.0041778	2.6134845	0	1.4456208	0.4220177	2.4413507
0.6112668	2.9913903	0	1.1012467	0.6035967	3.7425345
1.3304437	1.6606549	0.444985	0.647729	0.5574836	3.8915456
1.3127233	2.5264445	0	2.2704392	0.6793337	3.22033
1.2118221	3.0229891	0.4834676	1.2183472	0.6037866	4.636787
0.7995847	1.4679576	0.8553519	1.0776532	0.3538878	2.4559387
1.124262	3.1304532	0.4436067	1.0905147	0.4810922	2.667597
0.8551924	2.5852029	0.488515	1.1790014	0.9153297	2.9069098
1.427338	3.096245	0.4185678	0.9441088	0.3533233	4.2468337
0.5156115	1.772688	0.3965979	0.8026482	0.9999279	2.3180276
2.3432941	2.4798514	1.194066	3.8630398	3.4467326	4.6790465
1.860645	3.1079389	0	1.4685312	0.6327335	3.4388916
1.0697022	2.3036931	0	0.5720165	1.3894567	2.5086061
1.0634339	2.9897567	0	0.8985566	0.6488335	3.0514417
0.979001	2.4194041	0	0.634872	1.2753051	2.6347327
1.4773665	3.4700422	0	2.3755955	0.6665748	4.2597395
1.2319853	2.6145921	0	1.5939274	0.9801716	3.3103547
0.8368129	2.5556444	0.6705684	0.4308205	0.3613192	2.3716982
0.6100386	4.295914	0	0.9349316	3.5225329	2.8582198
1.6306384	4.0047261	0	1.4911348	1.2797684	4.5578328
1.7112746	3.9912543	0.3506104	1.7542889	2.5736169	4.5354123
0.520548	2.5173009	0	1.2492026	0.0961269	2.2353901
1.4793858	2.7866382	0	1.6527389	0.808591	3.4824869
1.5496692	3.4730725	0	1.4612666	1.4439249	3.1149666

0.8860428	2.6054232	0	1.1515341	0.2797684	3.919464
2.7899935	2.4975783	2.2210006	2.6916012	3.0741629	4.4143363
1.703898	2.721657	0.4109923	1.5866447	0.8781368	3.5299084
2.8745609	3.6660521	1.3977208	3.0251706	2.9773257	4.5913982
1.0320303	3.2232063	0	1.6237264	0.466862	4.6040001
1.2600257	2.9455515	0.549127	1.668936	0.8445463	3.609755
1.5393822	2.224349	0.4599561	2.0328061	0.6485575	4.0294706
0.425137	2.1534487	0	1.3747335	0.1107641	2.3493087
0.7482895	2.0779266	0	0.5490284	0.7909388	2.8872523
0.4107753	0.8507194	0.4315695	0.7515494	0.3098747	3.3166381
0.3896769	2.6755676	0	0.6676651	0.4982509	3.2589862
1.4355753	2.7229905	0	1.2253366	1.0828388	3.9219314
0.631989	3.0687396	0.2607483	1.1144337	1.8960781	3.2907488
0.6016014	2.2233299	0	0.632082	0.4701984	2.1806571
1.1765143	2.7092906	0.4589069	1.2150573	1.6837863	3.3032255
0.8431799	2.544881	0	0.8625109	0.6539772	2.8230564
1.2348079	3.2381143	0	1.7401504	1.2498094	3.7176161
1.8372974	3.5227337	0.3959402	1.4508549	1.5831821	3.7112636
0.878843	2.809826	0	1.3599709	0.4064278	3.5897635
1.9545313	2.4892346	0	2.6394406	1.7046064	4.7775091
0.9210554	3.286822	0.3450557	0.6133431	0.6387689	4.8196273
1.670795	2.9147367	0	1.6519127	0.6038815	3.7548127
0.7561693	2.6025757	0	0.6365434	0.7062874	3.3090306
1.2969566	2.1475998	0	3.0032424	0.8930515	3.2850767
1.328779	3.3751368	0.5868848	0.6695711	0.7860945	4.4596742
1.1582077	2.7213945	0	1.3467584	2.8375396	3.3305584
1.0070519	2.0453032	0	0.6529683	0.297837	2.5959094
0.7679937	2.0231844	0.3320491	0.7742494	0.2360946	3.7004397
1.324753	2.5690536	0	1.5502604	1.6834719	4.2672062
0.5038575	2.1486088	0	0.8246457	0.2167342	2.4226098
1.1891604	3.5674968	0.7983409	1.3481191	1.193204	3.7726458
1.2643563	2.4802393	0.3630028	2.4854011	0.9587313	3.6314652
0.9705592	1.8168045	0	1.1941501	0.6290068	2.8970274
1.5466599	2.4669925	0	0.9775364	0.4815056	4.8386342
1.2519311	1.6033118	0	0	0.2890081	2.45249
0.3489122	2.4732934	0	0.6486495	0.3088268	2.6448484
0.5475485	2.4093909	0	0.444243	0.2504766	2.1624679
1.1466552	2.1073866	0	0.9103489	0.1742786	4.080777
1.0356943	1.8790392	0.3637878	0.9258475	0.9310022	2.5645245
1.2894804	2.3378538	0	1.2571313	0.791439	4.4650537
2.3516849	3.8908066	0	1.9047731	1.0666059	4.3026334
1.9538612	3.8955547	0	1.5344601	0.6802343	3.3745944
1.0657102	2.2548359	0.5919666	0.922274	0.5732776	3.2578102

0.5555217	2.1861202	0	2.1343195	0.1309309	2.5981746
0.3610946	1.4145685	0	0	0.2875905	1.777325
0.6892992	1.9995671	0	0.3178829	0.0610158	2.1653975
0.8988662	2.7495343	0	0.791439	0.4878979	3.6485
0.6967951	2.5390595	0	0.8843244	0.3745109	2.4008658
1.1044038	3.5683848	0	2.2766753	0.7591558	4.3281327
0.6932305	2.1561377	0.2007554	0.7170348	0.8146735	2.7153443
0.8116354	2.1017509	0	2.0127117	0.1873877	2.1898246
0.6598332	2.4668098	0	1.439038	0.2629142	2.277955
0.9239478	2.0023065	0.6195539	1.1775351	0.6686638	3.114367
1.0821576	2.8622927	0.2033891	0.8726318	0.7716321	2.5751671
0.8841681	2.5102531	0	0.1732554	1.7720546	3.7338966
1.2290341	2.9837871	0	1.1859934	0.7122876	4.1396087
1.487332	3.5436815	0.4097982	2.9683304	1.0272248	3.945205
0.6212431	2.4784283	0.4065367	0.9211316	0.2912499	2.8386493
1.5478446	3.4167452	0	2.6070107	1.2869994	3.4277939
1.0276495	2.1418578	0.3234277	2.2006927	0.3519675	2.8493988
1.9668365	3.1129503	0	1.6010309	2.4102054	4.291935
0.5431989	2.7855087	0	0.2249046	0.055751	2.6499143
1.3148125	2.9011469	0	1.2944884	0.8186052	1.777325
0.9431337	2.5896916	0.5025343	1.2564069	1.2300185	4.0297532
1.2392147	2.7393297	0	1.1959145	0.1048734	3.4895943
1.1380936	2.1913416	0.7877668	0.8910303	0.3778456	3.2908372
1.3969815	3.4571871	0	1.4203477	1.0744369	3.5420351
1.3002409	2.7935422	0.4538599	1.5284713	0.7750927	3.6501327
1.5170741	2.9836777	0.3726178	2.0827366	1.1473718	4.0912513
0.854634	3.016104	0.8918858	1.743515	0.9995671	3.0954012
