# Supplementary material for: Establishment of a prognostic signature for lung adenocarcinoma using cuproptosis-related lncRNAs
Source: BMC Bioinformatics. 2023 Mar 6;24:81. doi: 10.1186/s12859-023-05192-5 (PMC9990240; doi:10.1186/s12859-023-05192-5)
Supplement: Supplementary file 3 — Additional file 3. The detail result of GO enrichment analysis. [file 12859_2023_5192_MOESM3_ESM.pdf]

Table S3 the detail result of GO enrichment analysis

ONTOLOGY	ID	Description	GeneRatio	BgRatio	pvalue	p.adjust	qvalue
BP	GO:0008544	epidermis deve	17/160	355/18800	1.00E-08	2.54E-05	2.31E-05
BP	GO:0050829	defense respon	7/160	87/18800	9.36E-06	0.0082818	0.0075405
BP	GO:0019730	antimicrobial	8/160	122/18800	9.78E-06	0.0082818	0.0075405
BP	GO:0045109	intermediate fi	6/160	68/18800	2.45E-05	0.0155511	0.014159
BP	GO:0043588	skin developm	11/160	296/18800	4.76E-05	0.0197501	0.0179822
BP	GO:0042303	molting cycle	7/160	114/18800	5.44E-05	0.0197501	0.0179822
BP	GO:0042633	hair cycle	7/160	114/18800	5.44E-05	0.0197501	0.0179822
BP	GO:0031424	keratinization	6/160	85/18800	8.69E-05	0.0228231	0.02078
BP	GO:0042634	regulation of h	4/160	28/18800	8.82E-05	0.0228231	0.02078
BP	GO:0045104	intermediate fi	6/160	88/18800	0.0001054	0.0228231	0.02078
BP	GO:0045103	intermediate fi	6/160	89/18800	0.0001123	0.0228231	0.02078
BP	GO:0051873	killing by host	4/160	30/18800	0.0001165	0.0228231	0.02078
BP	GO:1903532	positive regula	10/160	274/18800	0.0001226	0.0228231	0.02078
BP	GO:0051798	positive regula	3/160	12/18800	0.0001258	0.0228231	0.02078
BP	GO:0051384	response to glu	7/160	139/18800	0.0001891	0.0288277	0.0262472
BP	GO:0002475	antigen proces	3/160	14/18800	0.0002055	0.0288277	0.0262472
BP	GO:0045861	negative regul	11/160	350/18800	0.0002091	0.0288277	0.0262472
BP	GO:0009410	response to xer	12/160	411/18800	0.0002131	0.0288277	0.0262472
BP	GO:0055123	digestive syster	7/160	142/18800	0.0002156	0.0288277	0.0262472
BP	GO:0051047	positive regula	10/160	300/18800	0.0002552	0.0324153	0.0295136
BP	GO:0002062	chondrocyte di	6/160	110/18800	0.0003574	0.043229	0.0393594
BP	GO:0031960	response to cor	7/160	157/18800	0.0003975	0.0458874	0.0417798
BP	GO:0010466	negative regul	9/160	262/18800	0.0004183	0.0461965	0.0420612
BP	GO:0051797	regulation of h	3/160	19/18800	0.0005303	0.0528518	0.0481208
BP	GO:0061844	antimicrobial	5/160	79/18800	0.0005691	0.0528518	0.0481208
BP	GO:0030216	keratinocyte di	7/160	167/18800	0.0005753	0.0528518	0.0481208
BP	GO:0032496	response to lip	10/160	333/18800	0.0005797	0.0528518	0.0481208
BP	GO:0035902	response to im	3/160	20/18800	0.00062	0.0528518	0.0481208
BP	GO:0046887	positive regula	6/160	122/18800	0.0006205	0.0528518	0.0481208
BP	GO:0035987	endodermal ce	4/160	46/18800	0.0006242	0.0528518	0.0481208
BP	GO:0001704	formation of p	6/160	123/18800	0.0006477	0.0530736	0.0483227
CC	GO:0005921	gap junction	4/161	32/19594	0.000132	0.0314098	0.0291732
MF	GO:0071723	lipopeptide bir	4/154	10/18410	9.51E-07	0.00029	0.0002532
MF	GO:0030280	structural cons	5/154	37/18410	1.35E-05	0.0020554	0.0017947
MF	GO:0048018	receptor liganc	14/154	489/18410	6.39E-05	0.0052355	0.0045715
MF	GO:0030546	signaling recep	14/154	496/18410	7.43E-05	0.0052355	0.0045715
MF	GO:0008201	heparin bindin	8/154	168/18410	8.58E-05	0.0052355	0.0045715
MF	GO:0004867	serine-type end	6/154	98/18410	0.0001736	0.0088249	0.0077056
MF	GO:1901681	sulfur compou	9/154	267/18410	0.0004217	0.0164624	0.0143745
MF	GO:0008083	growth factor	7/154	162/18410	0.0004318	0.0164624	0.0143745
MF	GO:0005179	hormone activ	6/154	122/18410	0.0005657	0.018951	0.0165474

MF	GO:0004252	serine-type end	7/154	174/18410	0.0006613	0.018951	0.0165474
MF	GO:0005243	gap junction cl	3/154	21/18410	0.0006835	0.018951	0.0165474
MF	GO:0005539	glycosaminogly	8/154	234/18410	0.0008036	0.0204248	0.0178342
MF	GO:0030414	peptidase inhil	7/154	187/18410	0.0010096	0.0232124	0.0202683
MF	GO:0005544	calcium-depen	4/154	54/18410	0.0010759	0.0232124	0.0202683
MF	GO:0008236	serine-type pep	7/154	191/18410	0.0011416	0.0232124	0.0202683
MF	GO:0017171	serine hydrolas	7/154	195/18410	0.0012868	0.02453	0.0214188
MF	GO:0017147	Wnt-protein bi	3/154	30/18410	0.0019746	0.0334584	0.0292147
MF	GO:0022829	wide pore char	3/154	30/18410	0.0019746	0.0334584	0.0292147
MF	GO:0001530	lipopolysaccha	3/154	34/18410	0.0028401	0.0455915	0.039809
MF	GO:0061134	peptidase regu	7/154	230/18410	0.0032645	0.0486215	0.0424547
MF	GO:0071855	neuropeptide r	3/154	36/18410	0.0033477	0.0486215	0.0424547
MF	GO:0004866	endopeptidase	6/154	180/18410	0.0040964	0.0567914	0.0495884

geneID	Count
COL17A1/	17
CAMP/AZ	7
CAMP/AZ	8
KRT16/K	6
KRT16/K	11
KRT16/G	7
KRT16/G	7
KRT16/K	6
GAL/EPS	4
KRT16/K	6
KRT16/K	6
CAMP/AZ	4
NTSR1/P	10
GAL/FST/	3
PCSK1/PI	7
CD1E/CD	3
SERPINB7	11
PCSK1/SL	12
CLMP/PD	7
NTSR1/P	10
PTHLH/H	6
PCSK1/PI	7
SERPINB7	9
GAL/FST/	3
CAMP/EL	5
KRT16/K	7
CAMP/PC	10
GAL/UCN	3
PDX1/GA	6
HMGA2/	4
TBXT/TR	6
GJB3/GJB	4
CD1E/CD	4
KRT81/K	5
GAL/UCN	14
GAL/UCN	14
CEL/AZU	8
SERPINB7	6
CEL/AZU	9
INHA/IG	7
GAL/UCN	6

PCSK1/KI	7
GJB3/GJB	3
CEL/AZU	8
SERPINB7	7
PLA2G4F/	4
PCSK1/KI	7
PCSK1/KI	7
APCDD1L	3
GJB3/GJB	3
CAMP/AI	3
SERPINB7	7
GAL/UCN	3
SERPINB7	6
