# Supplementary material for: Establishment of a prognostic signature for lung adenocarcinoma using cuproptosis-related lncRNAs
Source: BMC Bioinformatics. 2023 Mar 6;24:81. doi: 10.1186/s12859-023-05192-5 (PMC9990240; doi:10.1186/s12859-023-05192-5)
Supplement: Supplementary file 4 — Additional file 4. The detail result of KEGG enrichment analysis. [file 12859_2023_5192_MOESM4_ESM.pdf]

Table S4 the detail result of KEGG enrichment analysis

ID	Description	GeneRatio	BgRatio	pvalue	p.adjust	qvalue	geneID	Count
hsa05146	Amoebiasis	8/75	102/8170	3.79E-06	0.0005036	0.0004823	SERPINB4,	8
hsa04310	Wnt signali	7/75	170/8170	0.0009016	0.0599546	0.057416	APCDD1L,	7
hsa04640	Hematopoi	5/75	99/8170	0.0020841	0.0923944	0.0884822	CD1E/CD1	5
hsa05204	Chemical c	4/75	69/8170	0.003609	0.1119077	0.1071692	GSTA2/CY	4
hsa00982	Drug metal	4/75	72/8170	0.0042071	0.1119077	0.1071692	GSTA2/CY	4
hsa00980	Metabolism	4/75	78/8170	0.0055974	0.1163087	0.1113839	GSTA2/CY	4
hsa00983	Drug metal	4/75	80/8170	0.0061215	0.1163087	0.1113839	GSTA2/CY	4
hsa04060	Cytokine-cy	7/75	295/8170	0.0181833	0.3022975	0.2894974	INHA/IL3	7
hsa00592	alpha-Lino	2/75	26/8170	0.023441	0.3117649	0.298564	PLA2G3/P	2
hsa04950	Maturity or	2/75	26/8170	0.023441	0.3117649	0.298564	PDX1/HN	2
hsa00591	Linoleic aci	2/75	30/8170	0.0306463	0.3530033	0.3380562	PLA2G3/P	2
hsa05202	Transcripti	5/75	193/8170	0.0318499	0.3530033	0.3380562	HOXA10/I	5
