# Supplementary material for: Establishment of a prognostic signature for lung adenocarcinoma using cuproptosis-related lncRNAs
Source: BMC Bioinformatics. 2023 Mar 6;24:81. doi: 10.1186/s12859-023-05192-5 (PMC9990240; doi:10.1186/s12859-023-05192-5)
Supplement: Supplementary file 5 — Additional file 5. The detail data of nomogram and calibration. [file 12859_2023_5192_MOESM5_ESM.pdf]

Table S5 the detail data of nomogram and calibration

| ID | futime | fustat | NIFK-AS1 | AC026355.2 | SEPSECS-A | AL360270.1 | AC010999.2 | ABCA9-AS1 |
|---|---|---|---|---|---|---|---|---|
| TCGA-62-A | 3.4136986 | 0 | 1.6458861 | 2.0037821 | 1.5109113 | 0.2747686 | 0 | 0.6912661 |
| TCGA-L9-A | 0.1589041 | 1 | 1.7285301 | 1.1073531 | 0.8864331 | 1.7777038 | 0 | 0.1142337 |
| TCGA-78-8 | 9.2082192 | 1 | 2.2767051 | 2.1504297 | 0.8904856 | 0.0635029 | 0 | 0.5479433 |
| TCGA-62-A | 1.6273973 | 1 | 1.6230709 | 0.5665716 | 1.2525973 | 1.0546401 | 0 | 0 |
| TCGA-38-4 | 3.1424658 | 1 | 1.8482373 | 0.9387751 | 0.6263924 | 0.6300326 | 0 | 0.0257376 |
| TCGA-86-A | 1.1369863 | 0 | 1.926417 | 2.3189242 | 0.3170725 | 0.8433407 | 0 | 0 |
| TCGA-97-8 | 1.3671233 | 0 | 1.8217102 | 2.0355535 | 0.230449 | 0.8094144 | 0.4565959 | 0.3074285 |
| TCGA-50-6 | 0.5178082 | 1 | 1.5531147 | 0.4722798 | 0.5809173 | 1.4765891 | 0 | 0 |
| TCGA-78-7 | 4.1863014 | 1 | 2.7582819 | 0.8914192 | 1.0702519 | 0.3674829 | 0.2055176 | 0.6598332 |
| TCGA-97-A | 1.6821918 | 0 | 1.6142858 | 0.7426961 | 1.2848102 | 1.1379625 | 0 | 0 |
| TCGA-78-7 | 2.2109589 | 1 | 2.2157097 | 0.9280481 | 1.8459918 | 2.5246404 | 0.3743996 | 0.9154062 |
| TCGA-50-8 | 3.0821918 | 0 | 1.9480027 | 1.4671751 | 1.400374 | 0.7987556 | 0.2424501 | 0 |
| TCGA-69-8 | 0.3534247 | 0 | 1.8317962 | 0.8284691 | 1.3331375 | 1.8219143 | 0.2675957 | 0.1261799 |
| TCGA-86-A | 0.3178082 | 1 | 1.8528378 | 0.2757223 | 1.6521423 | 0.0736832 | 0.2719037 | 0 |
| TCGA-78-8 | 19.347945 | 0 | 1.8997943 | 3.4568456 | 0.651775 | 2.2863489 | 0.1823109 | 0 |
| TCGA-97-8 | 0.4493151 | 1 | 2.4749551 | 1.2444 | 1.4936473 | 0.795102 | 0.3605328 | 0.059909 |
| TCGA-44-7 | 2.4356164 | 0 | 1.6638455 | 0.6084308 | 0.751378 | 0.5211513 | 0 | 0 |
| TCGA-78-7 | 0.490411 | 1 | 2.2463169 | 1.7959331 | 1.1412366 | 0.9758503 | 0 | 0 |
| TCGA-44-4 | 2.2136986 | 1 | 2.2070338 | 6.2884084 | 2.1200699 | 2.3212354 | 1.073786 | 1.0924781 |
| TCGA-71-6 | 0.7013699 | 0 | 1.3074285 | 3.4390247 | 0.988921 | 1.7126398 | 0.1054099 | 0 |
| TCGA-05-5 | 0.169863 | 0 | 1.4943644 | 1.13888 | 0.7441611 | 1.1352065 | 0 | 0 |
| TCGA-05-4 | 2.0849315 | 0 | 2.3566509 | 0.7472585 | 0.902035 | 1.4537019 | 0.0932897 | 0.0565835 |
| TCGA-78-7 | 13.591781 | 1 | 1.5716283 | 1.3148705 | 1.6388616 | 1.8580208 | 0.4850146 | 0 |
| TCGA-73-4 | 1.2794521 | 0 | 1.4670186 | 0.2979544 | 0.3188085 | 1.5837598 | 0.154324 | 0.0321008 |
| TCGA-38-4 | 8.1452055 | 0 | 2.5611061 | 0.6304055 | 0.8901743 | 0.5254676 | 0.1633699 | 0 |
| TCGA-67-3 | 1.169863 | 0 | 2.298629 | 3.801376 | 1.4158668 | 0.9159415 | 0 | 0 |
| TCGA-97-A | 1.709589 | 0 | 1.8169683 | 2.5622302 | 1.5087836 | 0.6410836 | 0.1384213 | 0.111432 |
| TCGA-78-7 | 2.1671233 | 0 | 2.3810341 | 1.2489598 | 1.5018722 | 0.4018489 | 0.1172961 | 0.2251515 |
| TCGA-67-3 | 1.6712329 | 0 | 2.2606881 | 2.8660178 | 0.6911768 | 1.8722378 | 0.2077679 | 0.0863762 |
| TCGA-49-4 | 1.0547945 | 1 | 2.3033716 | 0.9974731 | 1.3241769 | 0.8426973 | 0 | 0.0553345 |
| TCGA-55-8 | 1.6383562 | 1 | 1.8163539 | 1.4661833 | 0.6991962 | 1.1653653 | 0.1949696 | 0 |
| TCGA-99-A | 1.8027397 | 0 | 2.1718793 | 1.3562002 | 1.6678467 | 0.5840966 | 0 | 0 |
| TCGA-49-4 | 6.3506849 | 1 | 1.9117683 | 2.9064866 | 1.073409 | 0.5083272 | 0.0809305 | 0.0164963 |
| TCGA-44-2 | 0.2657534 | 1 | 2.2703495 | 2.9008443 | 1.4187477 | 1.5097634 | 0.9962682 | 0.9535757 |
| TCGA-62-8 | 3.3315068 | 0 | 2.1639173 | 1.8326875 | 1.4057201 | 1.8278597 | 0 | 0.0784732 |
| TCGA-05-4 | 0.0027397 | 0 | 1.6275601 | 0.7598376 | 0.9165531 | 1.4690524 | 0.2273714 | 0 |
| TCGA-55-6 | 0.3232877 | 1 | 1.7196654 | 0.7055799 | 0.5672534 | 1.7233401 | 0.2715452 | 0 |
| TCGA-73-4 | 6.890411 | 0 | 2.0787806 | 1.0085586 | 1.5945008 | 1.0164963 | 0.2471984 | 0.0526941 |
| TCGA-44-A | 0.9616438 | 0 | 1.5695883 | 1.5507529 | 0.8443053 | 0.9710745 | 0.1528973 | 0.0318186 |
| TCGA-NJ-A | 6.1945205 | 0 | 1.7037652 | 1.381228 | 1.3090597 | 0.4500631 | 0.1784916 | 0 |
| TCGA-05-4 | 2 | 0 | 1.833133 | 0.4839834 | 0.78668 | 1.0641931 | 0 | 0 |
| TCGA-NJ-A | 1.690411 | 0 | 2.4687397 | 2.5512453 | 1.4946204 | 0.5693939 | 0.3096419 | 0 |

| | | | | | | | | |
|---|---|---|---|---|---|---|---|---|
| TCGA-44-6 | 1.8027397 | 0 | 1.9414444 | 1.8648101 | 0.7482036 | 0.6209617 | 0 | 0.1909309 |
| TCGA-50-8 | 2.2712329 | 0 | 2.6739182 | 1.027791 | 1.8027722 | 0.4577519 | 0 | 0.032383 |
| TCGA-78-7 | 0.7068493 | 1 | 1.2649567 | 0.4684269 | 0.9544197 | 0.7263953 | 0.1478277 | 0 |
| TCGA-05-4 | 2.1671233 | 0 | 1.9730234 | 1.3443172 | 1.1956626 | 1.3879139 | 0 | 0 |
| TCGA-49-4 | 0.7342466 | 1 | 1.7248685 | 0.9985566 | 0.7623063 | 1.2379308 | 0.1578198 | 0.1272371 |
| TCGA-78-7 | 1.7150685 | 1 | 1.772308 | 0.5916794 | 0.4422269 | 1.3116775 | 0.1696685 | 0.3769571 |
| TCGA-55-7 | 1.8356164 | 0 | 1.8586176 | 0.5526722 | 0.4806787 | 1.0206978 | 0.2594232 | 0.1774714 |
| TCGA-55-6 | 3.3945205 | 0 | 1.6006979 | 0.3797318 | 1.1434587 | 0.6909981 | 0.3070787 | 0.0336521 |
| TCGA-78-7 | 3.2794521 | 1 | 1.572065 | 0.3660284 | 1.0361868 | 1.8428582 | 0.0990932 | 0 |
| TCGA-99-8 | 3.0630137 | 0 | 1.6382129 | 0.8234233 | 0.6979517 | 0.6388616 | 0.186247 | 0 |
| TCGA-05-4 | 1.5835616 | 0 | 1.3711127 | 0.3677065 | 0.7355222 | 0.8436623 | 0 | 0 |
| TCGA-78-7 | 7.3452055 | 1 | 2.9411814 | 3.1706622 | 1.7710827 | 1.285343 | 0.1127668 | 1.1044709 |
| TCGA-78-7 | 1.6054795 | 1 | 2.3720606 | 0.3995539 | 2.0441843 | 1.3717819 | 0.1437852 | 0.2735756 |
| TCGA-97-A | 1.7863014 | 0 | 2.1608557 | 1.429857 | 1.3278598 | 0.8304987 | 0 | 0.0364682 |
| TCGA-64-1 | 6.8164384 | 0 | 1.629986 | 1.192131 | 0.8076022 | 1.7601358 | 0.116897 | 0.047678 |
| TCGA-64-1 | 3.0849315 | 0 | 2.2335813 | 2.8055814 | 0.9096579 | 1.0976782 | 0 | 0 |
| TCGA-86-8 | 2.569863 | 0 | 1.8432201 | 1.3234277 | 1.2047042 | 0.5835191 | 0 | 0.1777265 |
| TCGA-78-7 | 2.2630137 | 1 | 1.2555007 | 1.7439458 | 0.9963887 | 1.5870769 | 0 | 0 |
| TCGA-99-8 | 2.9041096 | 0 | 2.3296977 | 0.1096949 | 0.574925 | 1.7646434 | 0.2086421 | 0 |
| TCGA-L9-A | 1.8191781 | 0 | 1.5395807 | 1.1926992 | 0.398022 | 0.9309265 | 0 | 0 |
| TCGA-55-8 | 1.4821918 | 0 | 1.6663476 | 1.0278618 | 1.4797997 | 2.1805935 | 0.4234168 | 0.3725064 |
| TCGA-86-A | 2.4547945 | 0 | 1.5999366 | 1.8958842 | 1.4172179 | 1.2582174 | 0 | 0.0832474 |
| TCGA-44-3 | 2.8109589 | 1 | 1.9068136 | 1.8295655 | 0.9040409 | 0.875151 | 0 | 0.0662614 |
| TCGA-69-7 | 0.630137 | 0 | 2.2128494 | 0.5965536 | 0.9237957 | 1.1021878 | 0.1164978 | 0.2026371 |
| TCGA-35-4 | 0.6164384 | 0 | 1.3888508 | 0.7017708 | 0.4311415 | 1.0234683 | 0.3002995 | 0 |
| TCGA-35-4 | 0.4986301 | 0 | 1.9149854 | 1.0100637 | 0.4955928 | 0.7494914 | 0.3780676 | 0 |
| TCGA-55-6 | 2.7260274 | 1 | 1.3021728 | 2.3938555 | 0.7968469 | 1.5688104 | 0 | 0 |
| TCGA-86-8 | 2.6 | 0 | 2.6038578 | 1.6152279 | 1.96857 | 1.5786972 | 0.3392514 | 0 |
| TCGA-05-4 | 0.830137 | 1 | 1.8861209 | 1.1050746 | 1.2975436 | 1.0222613 | 0 | 0.9958824 |
| TCGA-05-4 | 1 | 0 | 1.710305 | 1.5953604 | 1.5354558 | 0.2971327 | 0.5682267 | 0.8038057 |
| TCGA-05-4 | 1.2465753 | 0 | 1.3309599 | 0.3640121 | 0.6059684 | 1.2367681 | 0 | 0 |
| TCGA-MP- | 6.1589041 | 0 | 2.3208601 | 4.3677345 | 2.7864709 | 1.4128357 | 0.1351408 | 0.0554733 |
| TCGA-97-8 | 1.509589 | 0 | 1.5070081 | 2.325041 | 0.6121166 | 0.151729 | 0 | 0.0250288 |
| TCGA-97-A | 1.7232877 | 0 | 1.7589001 | 1.0513025 | 0.7054029 | 0.4731115 | 0.1322478 | 0.0272956 |
| TCGA-05-4 | 2.4986301 | 0 | 1.8126214 | 0.6050202 | 0.6574571 | 1.3938555 | 0.3618806 | 0.0483757 |
| TCGA-38-4 | 3.7178082 | 1 | 1.6224619 | 1.6856711 | 0.6531518 | 0.974823 | 0.628447 | 0 |
| TCGA-78-7 | 19.857534 | 0 | 2.5607883 | 1.5471043 | 1.4518573 | 0.3640121 | 0.2501127 | 0 |
| TCGA-MP- | 2.6027397 | 1 | 2.7832471 | 2.0251351 | 1.4768483 | 2.1369788 | 0.5029416 | 0.3450557 |
| TCGA-50-5 | 1.1890411 | 1 | 3.0737346 | 0.2691529 | 1.2688535 | 1.5046204 | 0.4896457 | 1.061638 |
| TCGA-MP- | 4.1123288 | 1 | 1.7973866 | 2.8881685 | 0.6768992 | 0.7228157 | 0 | 0.0721744 |
| TCGA-78-7 | 1.6246575 | 1 | 1.7887275 | 1.1081564 | 1.0770379 | 0.655535 | 0 | 0.5676428 |
| TCGA-73-4 | 1.9479452 | 1 | 1.6257381 | 1.423363 | 0.5981746 | 0.4166236 | 0.287945 | 0.015783 |
| TCGA-69-7 | 1.1260274 | 0 | 2.6375407 | 1.3329084 | 2.2825288 | 0.8304176 | 0.6925165 | 0.6428402 |

| | | | | | | | | |
|---|---|---|---|---|---|---|---|---|
| TCGA-73-7 | 3.2575342 | 0 | 2.5056369 | 3.0379796 | 1.0243906 | 0.8342258 | 0.3810618 | 0.0216219 |
| TCGA-78-7 | 0.8794521 | 1 | 1.5897156 | 0.5045695 | 1.0193461 | 1.6248028 | 0 | 0 |
| TCGA-05-4 | 0.3315068 | 1 | 1.9805006 | 0.8274939 | 0.6782522 | 0.9194545 | 0 | 0.0538064 |
| TCGA-38-4 | 2.939726 | 1 | 2.910771 | 0.0973411 | 1.3837181 | 0.4670708 | 0 | 0.0581086 |
| TCGA-05-4 | 0.7506849 | 1 | 1.21456 | 0.4239546 | 0.6628435 | 1.0345679 | 0.1534162 | 0.0319597 |
| TCGA-55-8 | 2.4767123 | 0 | 2.1519562 | 1.70982 | 1.3631711 | 0.9670948 | 0.3879139 | 0.3350258 |
| TCGA-55-7 | 2.4356164 | 0 | 1.6830227 | 4.9093363 | 0.8902521 | 0.8127036 | 0.5017194 | 0.2618317 |
| TCGA-55-6 | 0.6493151 | 1 | 1.5132886 | 2.5776101 | 0.5668639 | 1.5134402 | 0.2053925 | 0 |
| TCGA-62-A | 2.4931507 | 0 | 2.0760802 | 3.3309742 | 1.1025238 | 1.3238888 | 1.0579008 | 0.1476974 |
| TCGA-62-8 | 1.2164384 | 1 | 1.7126398 | 1.9564656 | 0.8410875 | 1.7650679 | 0.1493894 | 0.0614306 |
| TCGA-MP- | 1.1753425 | 1 | 1.7083638 | 2.239123 | 0.6586457 | 0.7759355 | 0 | 0.078883 |
| TCGA-44-8 | 0.7123288 | 0 | 2.9218362 | 1.1307332 | 1.1749177 | 1.0497702 | 0.2391536 | 0 |
| TCGA-73-4 | 2.1917808 | 0 | 1.8062828 | 1.4118601 | 0.7774513 | 0.9150237 | 0.1082233 | 0.0221902 |
| TCGA-78-7 | 0.6684932 | 1 | 2.1740868 | 1.4138107 | 1.1765143 | 1.4826934 | 0.108491 | 0 |
| TCGA-55-7 | 2.3890411 | 0 | 1.851759 | 2.0312187 | 0.9375704 | 1.411155 | 0 | 0 |
| TCGA-62-8 | 0.3808219 | 1 | 1.7466567 | 0.6686638 | 0.9776096 | 1.2503553 | 0.3013535 | 0 |
| TCGA-86-8 | 1.1589041 | 0 | 2.3610946 | 0.2744108 | 1.0710762 | 1.4979955 | 0.2705887 | 0 |
| TCGA-50-5 | 1.260274 | 1 | 1.4259963 | 0.7796379 | 0.8061178 | 1.842295 | 0 | 0 |
| TCGA-38-7 | 2.1917808 | 1 | 1.5662307 | 1.0613615 | 0.5462648 | 1.0308304 | 0 | 0 |
| TCGA-05-4 | 0.0027397 | 1 | 1.9119216 | 2.213565 | 0.5247658 | 1.5071096 | 0.2492633 | 1.5630605 |
| TCGA-49-4 | 4.6575342 | 0 | 1.5301706 | 0.7564256 | 1.0061185 | 0.5988416 | 0 | 0.1074201 |
| TCGA-62-A | 4.5287671 | 1 | 1.6932305 | 1.2653769 | 0.9901562 | 0.5101518 | 0.2600257 | 0.0281448 |
| TCGA-44-3 | 3.2410959 | 0 | 2.3708896 | 5.0359406 | 1.7080989 | 2.587509 | 1.7122876 | 0.597698 |
| TCGA-05-4 | 0.2493151 | 1 | 1.7066853 | 0.3514022 | 0.7063758 | 2.4935961 | 0 | 0 |
| TCGA-44-6 | 1.630137 | 0 | 1.7376435 | 1.7195339 | 0.9919707 | 0.5797594 | 0 | 0 |
| TCGA-78-7 | 1.8246575 | 1 | 1.8387905 | 0.504061 | 1.3343394 | 2.35834 | 0.1856129 | 0.2529 |
| TCGA-69-7 | 1.890411 | 0 | 1.8263146 | 1.6436253 | 0.6030269 | 1.0174231 | 0 | 0 |
| TCGA-95-7 | 1.3068493 | 0 | 1.6869711 | 0.3842709 | 1.2785201 | 1.403595 | 0.2635152 | 0 |
| TCGA-86-7 | 2.8657534 | 1 | 1.6314768 | 0.9668734 | 0.8694766 | 2.0313599 | 0.0938306 | 0 |
| TCGA-49-4 | 3.8931507 | 1 | 2.4113449 | 1.761966 | 0.6310577 | 0.6293799 | 0 | 0.0182069 |
| TCGA-91-6 | 0.2164384 | 0 | 2.5512453 | 2.273307 | 0.175301 | 1.3050366 | 0 | 0.044604 |
| TCGA-50-6 | 3.5287671 | 1 | 1.8273313 | 0.8554316 | 0.5378427 | 1.1428708 | 0 | 0 |
| TCGA-50-5 | 1.309589 | 1 | 1.7035437 | 2.7525131 | 0.8880711 | 0.9997114 | 0 | 0 |
| TCGA-91-6 | 1.0191781 | 0 | 2.1673258 | 0.2873541 | 0.8494389 | 0.7763567 | 0 | 0 |
| TCGA-49-4 | 2.4547945 | 1 | 2.4302317 | 1.0308304 | 0.8823692 | 0.4013028 | 0.1646576 | 0.0343566 |
| TCGA-35-3 | 0.0383562 | 0 | 1.9443337 | 4.7970234 | 0.8890062 | 1.2120712 | 0.4205094 | 0 |
| TCGA-97-A | 1.5561644 | 0 | 2.082975 | 2.3025238 | 1.7816115 | 0.4072984 | 0.1751733 | 0.1072861 |
| TCGA-53-7 | 1.1616438 | 0 | 1.8211387 | 1.0619144 | 0.8777442 | 0.5112656 | 0.5450788 | 0 |
| TCGA-78-8 | 6.4657534 | 0 | 1.9972563 | 3.0222258 | 1.0434145 | 0.683921 | 0.4534386 | 0 |
| TCGA-86-8 | 0.339726 | 1 | 2.0259147 | 1.7756827 | 0.5383395 | 1.1716552 | 0 | 0.0771062 |
| TCGA-49-6 | 1.430137 | 0 | 1.4276062 | 0.5605193 | 0.5550308 | 1.7154541 | 0 | 0 |
| TCGA-MP- | 2.030137 | 0 | 2.146753 | 2.7413375 | 1.0941009 | 0.4938522 | 0.516822 | 0 |
| TCGA-99-7 | 2.0465753 | 0 | 2.1324452 | 0.581303 | 2.153935 | 1.0611541 | 0.3709454 | 0.0278618 |

| | | | | | | | | |
|---|---|---|---|---|---|---|---|---|
| TCGA-55-1 | 3.2273973 | 0 | 2.3415019 | 0.4325319 | 1.6915788 | 1.3757345 | 0.4269624 | 0.2143735 |
| TCGA-95-7 | 1.0328767 | 0 | 1.762774 | 0.7567671 | 0.2584586 | 1.4815573 | 0.2028878 | 0.0427844 |
| TCGA-05-4 | 3.9205479 | 0 | 1.4616333 | 1.6666657 | 0.514905 | 1.2140626 | 0.1433934 | 0.0149265 |
| TCGA-78-7 | 0.7972603 | 1 | 2.0398753 | 1.4268551 | 0.7409707 | 0.6864333 | 0.1152996 | 0.0926134 |
| TCGA-50-5 | 6.5561644 | 1 | 1.4302317 | 1.2467121 | 1.3502709 | 0.9871753 | 0 | 0 |
| TCGA-78-7 | 1.909589 | 1 | 1.7881428 | 1.5262692 | 1.3467584 | 1.5639879 | 0 | 0.047678 |
| TCGA-62-8 | 4.1041096 | 1 | 2.1884326 | 0.7773671 | 1.4190534 | 0.8042188 | 0.1897929 | 0.1164978 |
| TCGA-78-8 | 3.3123288 | 1 | 1.336569 | 0.4881036 | 0 | 0.1377658 | 0.3383388 | 0 |
| TCGA-44-3 | 2.8383562 | 0 | 2.3632739 | 5.1001984 | 1.6948208 | 2.3353975 | 1.4157226 | 0.8759897 |
| TCGA-55-A | 1.4958904 | 0 | 2.0604279 | 1.6917575 | 1.1669082 | 2.1144337 | 0.2740529 | 0 |
| TCGA-78-7 | 2.6 | 1 | 2.3312753 | 1.8125393 | 1.6075315 | 1.417974 | 0.0806577 | 0 |
| TCGA-55-A | 0.0767123 | 0 | 1.4540179 | 1.1560082 | 0.5089357 | 1.4678533 | 0.1819294 | 0.0381552 |
| TCGA-50-5 | 3.3835616 | 1 | 2.0451284 | 0.3230818 | 1.2030132 | 1.1693478 | 0.1681285 | 0.1028597 |
| TCGA-05-5 | 0.4136986 | 0 | 1.857583 | 3.0980152 | 1.5496199 | 0.7616257 | 0 | 0.0458623 |
| TCGA-62-A | 1.1342466 | 1 | 2.1198541 | 0.5751187 | 0.7311832 | 1.3698294 | 0.1642714 | 0.0342157 |
| TCGA-97-A | 1.6465753 | 0 | 2.6322449 | 0.951364 | 1.4489538 | 1.0324535 | 0.1570437 | 0.2428159 |
| TCGA-95-7 | 1.5561644 | 0 | 1.8538759 | 2.2292187 | 0.6004601 | 1.0906502 | 0.097476 | 0.0199154 |
| TCGA-55-7 | 2.8493151 | 0 | 2.0620526 | 1.0500489 | 0.6125884 | 1.6870607 | 0.4345615 | 0.2752455 |
| TCGA-95-8 | 0.230137 | 0 | 1.8360049 | 1.6260185 | 0.8212204 | 0.6474527 | 0.458067 | 0 |
| TCGA-55-1 | 1.9205479 | 1 | 2.6495005 | 0.9327415 | 1.1957885 | 1.9589911 | 0.3509498 | 0.2701103 |
| TCGA-44-2 | 3.5643836 | 0 | 2.7483539 | 5.2143152 | 2.8668486 | 1.6344306 | 1.7849855 | 0.9145263 |
| TCGA-78-7 | 3.3287671 | 1 | 1.5189883 | 2.1528 | 0.3395935 | 1.2313095 | 0 | 0 |
| TCGA-64-5 | 0.169863 | 1 | 1.357552 | 0.198997 | 0.5443865 | 1.1228052 | 0.3688242 | 0 |
| TCGA-78-7 | 9.9589041 | 0 | 1.4807304 | 0.5103543 | 0.9587313 | 0.4812989 | 0.2738142 | 0 |
| TCGA-MP- | 0.4410959 | 1 | 1.1471113 | 3.0422241 | 0.705049 | 1.2708877 | 0 | 0.031254 |
| TCGA-44-2 | 3.1753425 | 0 | 1.7868891 | 0.6673926 | 0.8511194 | 0.6837414 | 0.220949 | 0.0236102 |
| TCGA-73-4 | 4.3835616 | 1 | 1.4639363 | 0.2000021 | 0.2635152 | 1.6703418 | 0.1971105 | 0 |
| TCGA-44-7 | 2.4164384 | 0 | 3.3186928 | 0.3729521 | 0.9368923 | 1.1997509 | 0.133432 | 0.0275787 |
| TCGA-55-8 | 1.6410959 | 0 | 1.763794 | 0.3828332 | 0.8302553 | 0.7270927 | 0.2624332 | 0.0563061 |
| TCGA-55-8 | 1.6630137 | 1 | 2.0860364 | 1.4549655 | 1.278639 | 0.8745216 | 0.1787465 | 0.0374525 |
| TCGA-55-8 | 1.4986301 | 0 | 2.0705954 | 0.2488991 | 0.7457106 | 0.3922075 | 0.3188085 | 0.0176369 |
| TCGA-44-A | 0.7863014 | 0 | 1.8415706 | 2.0559938 | 0.915865 | 1.3255591 | 0.175812 | 0 |
| TCGA-64-5 | 3.5753425 | 0 | 2.7036766 | 1.1768334 | 1.6325474 | 0.6770797 | 1.0901082 | 0 |
| TCGA-86-7 | 1.6575342 | 0 | 1.9698596 | 2.2993326 | 1.9301696 | 0.7359554 | 0.2333973 | 0.1890338 |
| TCGA-62-8 | 7.3863014 | 0 | 1.83289 | 5.1265393 | 1.7534334 | 2.9390761 | 0.1548424 | 0.0322419 |
| TCGA-95-7 | 0.2383562 | 1 | 1.6429788 | 0.4371212 | 0.6636634 | 1.1154994 | 0.231801 | 0.0492125 |
| TCGA-69-7 | 0.5534247 | 0 | 1.3072536 | 2.622743 | 0.6971511 | 0.3170725 | 0.1480881 | 0.0308304 |
| TCGA-55-7 | 2.7260274 | 1 | 1.8181961 | 1.2189051 | 1.0341453 | 1.1551663 | 0 | 0 |
| TCGA-MP- | 0.4575342 | 1 | 1.859254 | 2.0840302 | 0.9795132 | 1.1005068 | 0.5990321 | 0.1674864 |
| TCGA-95-A | 1.5150685 | 0 | 1.3953919 | 2.2516888 | 0.782073 | 0.4138107 | 0.1628545 | 0 |
| TCGA-86-7 | 3.169863 | 0 | 2.499476 | 0.432425 | 0.4978423 | 1.0310422 | 0 | 0.0957219 |
| TCGA-50-5 | 1.709589 | 1 | 1.212009 | 0.8919635 | 0.3524196 | 0.1733833 | 0 | 0 |
| TCGA-64-5 | 7.3315068 | 0 | 2.5934016 | 0.8951475 | 0.921284 | 1.0061903 | 0.4466797 | 0.3116194 |

| | | | | | | | | |
|---|---|---|---|---|---|---|---|---|
| TCGA-4B-A | 0.8219178 | 1 | 1.5191896 | 0.9046575 | 0.9159415 | 0.4592218 | 0.3036931 | 0.8678174 |
| TCGA-83-5 | 2.2575342 | 0 | 1.6560844 | 1.3683214 | 0.7417474 | 1.8250529 | 0.1632411 | 0.0673634 |
| TCGA-44-A | 1.1369863 | 0 | 1.4833128 | 2.7146192 | 1.3902274 | 1.5402257 | 0 | 0.5581697 |
| TCGA-L4-A | 1.5835616 | 0 | 1.5265196 | 1.6076262 | 1.1286233 | 1.877705 | 0 | 0 |
| TCGA-97-8 | 1.5561644 | 0 | 1.963844 | 1.1071522 | 0.8646517 | 0.5005981 | 0.088413 | 0 |
| TCGA-78-7 | 0.4739726 | 1 | 1.3972553 | 2.6843475 | 0.9772433 | 0.9355352 | 0.3662523 | 1.6730136 |
| TCGA-50-5 | 0.7726027 | 1 | 1.8611614 | 0.5401265 | 0.9622899 | 1.165301 | 0.291132 | 0.1231364 |
| TCGA-64-5 | 4.2712329 | 0 | 1.3827779 | 2.359999 | 0.605115 | 1.2185952 | 0 | 0 |
| TCGA-NJ-A | 0.1369863 | 0 | 1.7774513 | 0.70982 | 0.5062973 | 2.5112909 | 0 | 0.032383 |
| TCGA-44-8 | 0.7808219 | 0 | 2.1713349 | 0.4645637 | 0.6205864 | 0.7731524 | 0.3915478 | 0.0354832 |
| TCGA-44-2 | 3.6273973 | 0 | 2.1659119 | 4.3316049 | 0.8448677 | 0.580435 | 0.088413 | 0 |
| TCGA-86-8 | 2.3616438 | 0 | 2.2454044 | 3.1489666 | 1.1796384 | 1.1615008 | 0 | 0.3765127 |
| TCGA-MP- | 0.9205479 | 1 | 2.3037515 | 2.3048906 | 1.9012241 | 2.3558902 | 0 | 1.3708338 |
| TCGA-05-4 | 0.0027397 | 0 | 1.9289198 | 1.6444101 | 1.2591218 | 0.8621141 | 0.2514464 | 0 |
| TCGA-05-4 | 1.6630137 | 0 | 2.0128547 | 3.7405175 | 1.0194884 | 1.23444 | 0.5058909 | 0 |
| TCGA-73-4 | 0.3589041 | 0 | 1.6999957 | 1.0243906 | 0.3171883 | 1.4544918 | 0.3779566 | 0.0170667 |
| TCGA-44-3 | 3.1863014 | 0 | 1.6972845 | 3.0439569 | 0.85591 | 0.4811956 | 0.5212518 | 0.1200865 |
| TCGA-73-A | 0.9315068 | 1 | 2.0909889 | 0.9492735 | 1.0025945 | 2.1239641 | 0.5443865 | 0 |
| TCGA-97-A | 1.7369863 | 0 | 1.8467942 | 0.4005379 | 1.1140338 | 0.9279723 | 0.1441769 | 0.1973622 |
| TCGA-62-A | 5.6630137 | 0 | 2.051024 | 2.5852029 | 1.9626601 | 1.0627434 | 0.3176514 | 0 |
| TCGA-35-5 | 0.7232877 | 0 | 1.866908 | 1.3654126 | 0.7573647 | 0.1350094 | 0 | 0 |
| TCGA-MP- | 7.169863 | 1 | 1.4068087 | 1.0800436 | 0.6156988 | 0.812375 | 0.2624332 | 0 |
| TCGA-05-4 | 2.0849315 | 0 | 1.8956904 | 1.503654 | 0.7438165 | 1.3777901 | 0 | 0 |
| TCGA-05-4 | 2.5013699 | 0 | 2.2174169 | 2.8187892 | 0.9396027 | 1.038998 | 0 | 0.1998765 |
| TCGA-86-8 | 2.0273973 | 0 | 1.4115889 | 2.750778 | 1.2854614 | 3.4628507 | 0 | 0.0729976 |
| TCGA-55-6 | 2.0821918 | 1 | 1.3761792 | 0.3959402 | 0.4898512 | 1.0433445 | 0 | 0 |
| TCGA-69-7 | 1.1342466 | 0 | 2.334082 | 0.8539158 | 1.6372856 | 1.0033144 | 0.2128806 | 0.0886844 |
| TCGA-55-8 | 1.0273973 | 1 | 3.0109588 | 0.1988713 | 1.5714341 | 0.8257042 | 0.7890197 | 0 |
| TCGA-53-7 | 2.8575342 | 1 | 2.1445359 | 0.1914363 | 1.6068686 | 2.436535 | 0.1886541 | 0.2570106 |
| TCGA-62-A | 4.7260274 | 1 | 1.8856134 | 0.1601457 | 1.2290957 | 1.7755563 | 0.4294285 | 0 |
| TCGA-78-7 | 2.6739726 | 1 | 2.1075874 | 4.1559597 | 1.7538184 | 1.3726736 | 0.5462648 | 1.0920044 |
| TCGA-NJ-A | 3.9232877 | 0 | 1.4764335 | 2.5258434 | 1.2750667 | 2.4916479 | 0.2906603 | 0 |
| TCGA-50-5 | 0.6849315 | 1 | 2.0199509 | 1.4294821 | 1.0649518 | 1.4729556 | 0.162339 | 1.0975434 |
| TCGA-55-6 | 5.8547945 | 0 | 1.2973088 | 2.9288061 | 0.5768607 | 1.0032424 | 0.1319845 | 0 |
| TCGA-44-2 | 3.5068493 | 0 | 2.2156682 | 5.6207438 | 2.1087475 | 1.7441898 | 1.2164859 | 1.587941 |
| TCGA-44-2 | 3.9150685 | 0 | 2.0842117 | 4.7293352 | 1.469313 | 2.3526173 | 1.2053091 | 0.9030126 |
| TCGA-64-5 | 2.3726027 | 0 | 1.4120227 | 1.6689814 | 0.458277 | 0.9900109 | 0 | 0 |
| TCGA-95-7 | 1.3041096 | 0 | 2.8283472 | 3.1076544 | 1.7055799 | 0.6995516 | 0.8914192 | 0 |
| TCGA-86-6 | 0.490411 | 0 | 1.5068558 | 0 | 1.1868808 | 0.9344033 | 0.0802483 | 0.0163537 |
| TCGA-78-7 | 4.4438356 | 1 | 1.9583601 | 1.2559841 | 1.0924104 | 1.2520523 | 0.1003049 | 0.0204844 |
| TCGA-05-4 | 1.2520548 | 1 | 1.8428984 | 1.063641 | 0.9851361 | 0.9177754 | 0.1625968 | 0 |
| TCGA-05-5 | 2.4164384 | 0 | 1.7055356 | 0.9689386 | 0.5648171 | 0.6586457 | 0 | 0.0382957 |
| TCGA-50-5 | 4.0383562 | 0 | 1.5433969 | 0.5576797 | 1.0719686 | 0.7359554 | 0.2077679 | 0 |

| | | | | | | | | |
|---|---|---|---|---|---|---|---|---|
| TCGA-05-5 | 0.7534247 | 1 | 1.4339743 | 0.7923556 | 0.6927843 | 0.7895206 | 0.2390314 | 0 |
| TCGA-86-A | 2.0191781 | 1 | 1.5334637 | 2.7914807 | 0.6244285 | 0.5070081 | 0.2730981 | 0 |
| TCGA-MP- | 0.2027397 | 1 | 1.5061449 | 2.8489183 | 0.7775354 | 0.9979066 | 0.3272275 | 0.0714881 |
| TCGA-64-1 | 3.1972603 | 1 | 2.1686099 | 0.9787082 | 1.6993295 | 1.2125693 | 0 | 0 |
| TCGA-05-4 | 4.1726027 | 0 | 2.0243197 | 1.2329063 | 0.6994628 | 0.8607642 | 0 | 0 |
| TCGA-50-5 | 8.4767123 | 0 | 1.4864055 | 1.8676593 | 0.6057788 | 0.7524061 | 0 | 0 |
| TCGA-86-8 | 0.0520548 | 1 | 1.5710943 | 0.6042611 | 1.1009776 | 1.7167715 | 0 | 0.3083609 |
| TCGA-50-5 | 3.9506849 | 0 | 1.5977219 | 1.7270709 | 1.1881477 | 0.9951227 | 0.0462815 | 0.0370307 |
| TCGA-86-8 | 2.2082192 | 0 | 1.8211796 | 4.7771619 | 1.3028747 | 1.3726178 | 0.1928254 | 0.1557492 |
| TCGA-50-5 | 5.060274 | 0 | 2.593306 | 0.3846025 | 1.1471113 | 1.2440346 | 0 | 0 |
| TCGA-69-A | 1.6191781 | 0 | 1.9712216 | 2.3466449 | 0.8356816 | 0.898634 | 0 | 0.1378969 |
| TCGA-50-6 | 2.1287671 | 1 | 2.0330529 | 0.4460444 | 1.4888235 | 0.8085086 | 0.3078948 | 0.3612069 |
| TCGA-49-4 | 2.9616438 | 1 | 1.2514464 | 1.0738202 | 0.0858325 | 0.5706572 | 0 | 0 |
| TCGA-44-2 | 2.0849315 | 1 | 1.8466203 | 3.0655838 | 1.2696516 | 1.3267866 | 0.3329275 | 0.4730769 |
| TCGA-50-6 | 0.060274 | 1 | 2.1492594 | 0.5014137 | 0.7891867 | 1.3315906 | 0 | 0 |
| TCGA-50-6 | 0.3260274 | 1 | 1.9972924 | 0.2617113 | 1.2913678 | 0.9191494 | 0.2580968 | 0.108491 |
| TCGA-49-4 | 2.3424658 | 1 | 1.587221 | 0.7411434 | 1.2371354 | 0.8760945 | 0.2207014 | 0.0920721 |
| TCGA-73-4 | 0.769863 | 1 | 0.979879 | 3.6998847 | 0.8316341 | 0.6104166 | 0.3538878 | 0 |
| TCGA-NJ-A | 5.9205479 | 0 | 1.4122396 | 2.0003606 | 0.955164 | 0.6660294 | 0.276318 | 0 |
| TCGA-44-7 | 1.969863 | 0 | 1.3741214 | 1.4591168 | 0.7418337 | 0.4718638 | 0.0892269 | 0 |
| TCGA-64-1 | 1.7205479 | 1 | 1.7593689 | 3.7171445 | 0.6848187 | 0.2571313 | 0 | 0.0545012 |
| TCGA-55-6 | 5.7780822 | 0 | 1.9192638 | 1.874797 | 0.5649147 | 0.7816535 | 0.2855798 | 0 |
| TCGA-86-7 | 1.7123288 | 1 | 2.0197731 | 1.2372578 | 1.0174944 | 1.1033298 | 0.3418719 | 0 |
| TCGA-86-8 | 1.9013699 | 1 | 1.8934787 | 1.8551127 | 1.0980152 | 1.2956055 | 0 | 0.1411712 |
| TCGA-MP- | 4.8712329 | 1 | 1.724301 | 1.9676113 | 0.9234915 | 0.7642612 | 0.2687937 | 0.0291348 |
| TCGA-86-8 | 2.2986301 | 0 | 1.9595476 | 1.5229597 | 1.1779178 | 0.7650255 | 0 | 0.0739573 |
| TCGA-67-3 | 1.569863 | 0 | 2.0897354 | 2.092681 | 0.380397 | 0.7625614 | 0.1268407 | 0.1264443 |
| TCGA-86-8 | 1.7890411 | 0 | 2.408413 | 0.2832106 | 2.0554733 | 0.6723801 | 0.8910303 | 0.328262 |
| TCGA-78-7 | 8.6821918 | 1 | 2.3099038 | 1.0158543 | 1.187261 | 0.7990044 | 0.1263121 | 0.3694944 |
| TCGA- MN | 2.2657534 | 0 | 1.5253172 | 1.0287106 | 0.6655747 | 1.6939441 | 0.4568061 | 0 |
| TCGA-49-4 | 2.7369863 | 1 | 0.9830209 | 0.2913678 | 0.1642714 | 1.1896664 | 0 | 0 |
| TCGA-62-A | 3.9835616 | 1 | 2.3156822 | 0.374177 | 2.1478928 | 2.315885 | 0.7900213 | 0.8357624 |
| TCGA-86-8 | 0.0657534 | 0 | 1.8671848 | 2.2276179 | 1.1641426 | 2.460297 | 0.1095612 | 0.0224743 |
| TCGA-86-7 | 2.5945205 | 0 | 1.8113477 | 0.514501 | 1.0684645 | 1.4644069 | 0 | 0 |
| TCGA-67-3 | 1.6712329 | 0 | 1.7749662 | 3.2508252 | 0.988412 | 0.3246954 | 0 | 0.1104969 |
| TCGA-38-4 | 2.3671233 | 1 | 1.2105759 | 0.1792562 | 0.5582676 | 0.729879 | 0.1767057 | 0 |
| TCGA-55-6 | 4.4712329 | 1 | 2.5541959 | 3.4506306 | 1.2808376 | 0.5354558 | 0.2895984 | 0.8813514 |
| TCGA-86-8 | 0.9671233 | 0 | 1.7137398 | 3.8957389 | 1.142152 | 1.9626231 | 0.3719491 | 0 |
| TCGA-05-4 | 3.0849315 | 0 | 1.5489791 | 0.5832302 | 0.5111644 | 0.8690818 | 0.2185952 | 0 |
| TCGA-67-6 | 1.1561644 | 0 | 1.8168045 | 2.9160753 | 0.9569488 | 1.6656202 | 0 | 0.1561377 |
| TCGA- MN | 3.2191781 | 0 | 1.4682184 | 3.5530533 | 0.548338 | 2.4422004 | 0 | 0.148869 |
| TCGA-L4-A | 1.1917808 | 0 | 1.7035437 | 1.1983056 | 1.1095612 | 0.5253673 | 0.1582077 | 0.187261 |
| TCGA-05-4 | 3.7506849 | 0 | 1.9063134 | 1.9088897 | 1.2372578 | 0.4480552 | 0.4213714 | 0.5085301 |

| | | | | | | | | |
|---|---|---|---|---|---|---|---|---|
| TCGA-55-8 | 1.8465753 | 0 | 1.6076262 | 1.7163764 | 1.1585309 | 0.6321751 | 0.2017593 | 0 |
| TCGA-55-6 | 3.5424658 | 1 | 2.1508846 | 0.1858665 | 0.9176991 | 1.7337448 | 0.4918018 | 0 |
| TCGA-55-8 | 1.9232877 | 1 | 1.8915358 | 0 | 0.623586 | 2.0469799 | 0 | 0.0652966 |
| TCGA-86-7 | 2.7315068 | 0 | 1.6176746 | 1.8547537 | 1.0971388 | 2.3094964 | 0.2609891 | 0.3745109 |
| TCGA-38-4 | 4.0876712 | 1 | 1.4845506 | 0.5582676 | 1.0199865 | 1.0295589 | 0.092884 | 0 |
| TCGA-55-A | 1.7260274 | 0 | 1.3325074 | 3.6687772 | 0.5637439 | 1.0496308 | 0 | 0.0301242 |
| TCGA-55-8 | 2.4328767 | 0 | 2.3600552 | 0.2200823 | 1.4615286 | 1.0891591 | 0.3142905 | 0.0683269 |
| TCGA-44-7 | 1.5260274 | 1 | 1.7539468 | 1.3288365 | 0.9540474 | 1.2956055 | 0.1365852 | 0.0282862 |
| TCGA-55-8 | 0.1315068 | 0 | 2.1030612 | 1.8128678 | 0.6180507 | 0.9668734 | 0 | 0 |
| TCGA-97-A | 1.4794521 | 0 | 1.878843 | 2.091666 | 0.5579737 | 0.7324863 | 0.16221 | 0 |
| TCGA-MP- | 7.1780822 | 1 | 1.9715895 | 2.3728685 | 0.9091971 | 0.7941871 | 0.164915 | 0.0680516 |
| TCGA-86-7 | 2.9369863 | 0 | 2.0425393 | 1.1763228 | 1.8981309 | 1.8738132 | 0.5512945 | 0 |
| TCGA-86-8 | 0.3808219 | 0 | 2.0939995 | 2.0315364 | 1.234256 | 1.486045 | 0 | 0 |
| TCGA-05-4 | 1.6712329 | 0 | 1.9181954 | 1.3225627 | 1.3172462 | 1.9787814 | 0 | 0 |
| TCGA-86-6 | 1.030137 | 1 | 1.5921102 | 0.5684213 | 0.7420062 | 1.7838757 | 0 | 0 |
| TCGA-67-6 | 0.3863014 | 0 | 1.5804832 | 2.1687704 | 0.372395 | 1.0107798 | 0 | 0 |
| TCGA-73-4 | 2.5260274 | 1 | 2.0090963 | 0.4404207 | 1.0279325 | 0.494262 | 0.1897929 | 0.0398403 |
| TCGA-64-1 | 4.7342466 | 0 | 2.6168987 | 3.0935602 | 0.8443857 | 0.7005285 | 0.6946574 | 0 |
| TCGA-55-6 | 3.7780822 | 1 | 1.2462256 | 1.4207249 | 0.6461627 | 0.7910222 | 0.2708279 | 0 |
| TCGA-L9-A | 1.5479452 | 0 | 2.2095469 | 0.251204 | 1.2220008 | 1.2889491 | 0.1292171 | 0.0267293 |
| TCGA-55-1 | 4.0520548 | 0 | 1.9398284 | 1.7389407 | 1.499425 | 0.4111008 | 0.6203987 | 0.2053925 |
| TCGA-55-6 | 8.9342466 | 0 | 1.8042188 | 1.5808208 | 0.8519988 | 0.8815863 | 0.258338 | 0 |
| TCGA-86-8 | 1.9205479 | 0 | 1.9466934 | 1.8627093 | 1.3131297 | 0.7278769 | 0.5497185 | 0.0524159 |
| TCGA-91-6 | 0.8849315 | 0 | 1.7633691 | 0.3234277 | 1.0590783 | 0.5851548 | 0 | 0 |
| TCGA-55-A | 1.8876712 | 0 | 2.3723392 | 0.3353689 | 1.5730351 | 1.3761237 | 0.826518 | 0 |
| TCGA-05-5 | 1.2520548 | 0 | 1.3905576 | 1.2634551 | 0.8622728 | 0.5506052 | 0.1570437 | 0.4506966 |
| TCGA-05-4 | 1.1671233 | 0 | 2.2492936 | 4.5497246 | 1.2385423 | 0.7633266 | 0.4520682 | 0.1505597 |
| TCGA- MN | 0.230137 | 0 | 1.7369944 | 2.0037821 | 1.1486738 | 2.1800205 | 0 | 0.0301242 |
| TCGA-95-A | 1.7835616 | 0 | 2.7350455 | 0.1781091 | 1.7614129 | 1.5012608 | 0.3320491 | 0.0367495 |
| TCGA-49-4 | 1.1726027 | 1 | 1.6898806 | 1.0394894 | 0.7494914 | 1.20989 | 0.1497796 | 0 |
| TCGA-05-4 | 2.0027397 | 1 | 2.5726957 | 1.8821735 | 1.8080967 | 0.0832474 | 0.8663937 | 0.0167816 |
| TCGA-86-8 | 1.2164384 | 1 | 1.5029925 | 0.5381408 | 0.3609822 | 1.8124572 | 0.1996252 | 0.198997 |
| TCGA-44-3 | 3.0958904 | 0 | 1.3197913 | 1.2092038 | 0.6254575 | 0.7542889 | 0 | 0.0254541 |
| TCGA-62-8 | 3.5315068 | 0 | 1.2441564 | 0.9007219 | 1.057069 | 0.6720179 | 0 | 0 |
| TCGA-78-7 | 10.794521 | 0 | 2.5740773 | 3.0372944 | 1.2371354 | 1.4958998 | 0.1432628 | 0.0873271 |
| TCGA-78-8 | 0.8794521 | 1 | 2.2282646 | 1.2024491 | 0.6280737 | 1.2863489 | 0.5131875 | 0.0404015 |
| TCGA-38-4 | 0.969863 | 1 | 1.3237736 | 0.9281239 | 0.7737432 | 1.3840498 | 0.199374 | 0.4688439 |
| TCGA-73-7 | 4.1945205 | 1 | 2.0141767 | 1.8699109 | 1.8002476 | 0.9266826 | 0.4815056 | 0.0668125 |
| TCGA-50-6 | 0.9205479 | 1 | 1.6578686 | 0.6009358 | 1.2013829 | 0.9581374 | 0.5934972 | 0 |
| TCGA-67-3 | 1.0547945 | 0 | 2.0831793 | 1.2368905 | 1.3925372 | 0.7117592 | 0 | 0 |
| TCGA-71-8 | 0.5753425 | 1 | 2.602148 | 1.2739932 | 1.0836559 | 1.440527 | 0 | 0.1104969 |
| TCGA-50-5 | 1.7890411 | 1 | 2.2624632 | 1.6271399 | 1.6804144 | 0.933346 | 0.228357 | 0 |
| TCGA-78-7 | 3.2082192 | 1 | 2.0268709 | 0.2611095 | 2.385072 | 1.1911836 | 0 | 0 |

| | | | | | | | | | |
|---|---|---|---|---|---|---|---|---|---|
| TCGA-44-5 | 2.7753425 | 0 | 2.3331375 | 2.8740887 | 0.6053995 | 1.0047531 | 0.4291071 | 0.6663021 |
| TCGA-44-2 | 3.7452055 | 0 | 1.4382396 | 1.8471551 | 0.8085086 | 1.0943712 | 0.247563 | 0.0354832 |
| TCGA-86-8 | 2.7205479 | 0 | 1.7166837 | 1.7546738 | 1.0592168 | 1.2110745 | 0.2027625 | 0.0214797 |
| TCGA-S2-A | 1.4054795 | 0 | 1.3938006 | 0.9920433 | 0.7108781 | 0.703898 | 0.1482183 | 0 |
| TCGA-MP- | 0.8410959 | 1 | 1.3981314 | 2.4735791 | 0.865998 | 0.614003 | 0 | 0.5043662 |
| TCGA-50-7 | 0.8438356 | 1 | 1.7775354 | 1.5944052 | 1.0515113 | 1.0248161 | 0.1596292 | 0.6413611 |
| TCGA-05-5 | 1.8356164 | 0 | 2.3353403 | 2.2410775 | 0.6928735 | 1.206018 | 0.2226809 | 0.0472592 |
| TCGA-44-8 | 1.0547945 | 0 | 1.6428864 | 1.7995018 | 0.7755142 | 1.8274532 | 0 | 0.0224743 |
| TCGA-05-4 | 1.8328767 | 0 | 2.2715751 | 0.229957 | 0.7838338 | 0.9918257 | 0.5949307 | 0 |
| TCGA-55-1 | 5.6575342 | 0 | 1.8434613 | 3.597984 | 1.6282603 | 0.9216648 | 0.2152437 | 0.0455828 |
| TCGA-99-8 | 0.1205479 | 0 | 1.5760383 | 0.5304703 | 0.5840004 | 0.8119642 | 0.1964811 | 0.1206174 |
| TCGA-67-6 | 0.4767123 | 0 | 1.9225785 | 2.3595493 | 0.9141052 | 1.2576745 | 0 | 0 |
| TCGA-97-7 | 2.1232877 | 0 | 1.6104166 | 0.8509594 | 0.8251343 | 1.4724358 | 0 | 0.0281448 |
| TCGA-05-4 | 2.1671233 | 0 | 2.0016581 | 1.1972993 | 0.7974281 | 1.1200201 | 0.6082415 | 0.0425043 |
| TCGA-50-6 | 3.4739726 | 1 | 1.8877202 | 0.2168584 | 0.4326388 | 1.3411888 | 0 | 0 |
| TCGA-50-6 | 1.0136986 | 1 | 2.0246034 | 1.2797684 | 1.0123541 | 0.8577422 | 0 | 1.287945 |
| TCGA-53-7 | 2.5452055 | 1 | 2.4218831 | 3.0441843 | 0.9831669 | 1.3946239 | 0.5465611 | 0 |
| TCGA-62-A | 6.0246575 | 0 | 2.5370227 | 3.8245438 | 1.4722278 | 0.9164002 | 0.1828194 | 0 |
| TCGA-50-5 | 5.0136986 | 1 | 1.8536364 | 1.1604685 | 0.7363018 | 0.5251668 | 0 | 0 |

| AC032011.1 | AL162632.3 | LINC02518 | LINC00592 | AL031600.2 | AP000346.1 | AC012409.4 | riskScore | risk |
|---|---|---|---|---|---|---|---|---|
| 0.064331 | 0.0614306 | 0.363115 | 1.0467006 | 0 | 0.1764504 | 0.3605328 | 1.837029 | high |
| 0.0563061 | 0 | 0.4142438 | 0.6289135 | 0 | 0.2947825 | 0 | 1.4779859 | high |
| 0.2699906 | 0.6071054 | 1.4440839 | 1.1230702 | 0.4287856 | 0.57415 | 0 | 2.4338245 | high |
| 0.2415962 | 0.0812033 | 0 | 0.4156505 | 0.3858175 | 0.119821 | 0 | 1.004778 | low |
| 0.3925372 | 0.1367164 | 1.5661819 | 0.2229282 | 0 | 0.0697022 | 0 | 3.7342447 | high |
| 0.3811726 | 0.1680001 | 0.1247252 | 0.5311693 | 0.725872 | 0.2445218 | 0 | 0.7724869 | low |
| 0.193204 | 0.2189671 | 0.0839282 | 0.2352369 | 0.5197432 | 0.1667154 | 0 | 0.7521256 | low |
| 0 | 0.1889073 | 0 | 0.1385524 | 0 | 0 | 0 | 2.1287923 | high |
| 0 | 0.4188916 | 0 | 0.590626 | 0.1998765 | 0 | 0 | 1.2250488 | high |
| 0.117695 | 0.5319678 | 0.1228715 | 0.2330291 | 1.3230241 | 0.3131297 | 0 | 0.4811575 | low |
| 0.0799754 | 0.0762854 | 0.1624679 | 1.3598023 | 0.6556266 | 0 | 1.045443 | 4.0909035 | high |
| 0.09815 | 0 | 0.1981169 | 0.1646576 | 0.2358496 | 0.0706641 | 0.2856981 | 0.4322334 | low |
| 0.1088924 | 0.0529723 | 1.3999367 | 0.0382957 | 0.4808855 | 0.1886541 | 0.3148705 | 1.1046307 | high |
| 0.1107641 | 0.4248146 | 0.115566 | 0 | 0.4882065 | 0 | 0 | 0.5089751 | low |
| 0.2716647 | 0 | 0.1483484 | 1.2981304 | 0.8352773 | 0.2886539 | 0 | 0.4476429 | low |
| 0.2851063 | 0.108491 | 0.1560082 | 0.248049 | 0 | 0 | 0.2263852 | 0.6715002 | low |
| 0 | 0.2503553 | 0.1871343 | 0.2413521 | 0 | 0 | 0 | 1.5964998 | high |
| 0.3920976 | 0.2266318 | 0 | 0.2056427 | 0 | 0 | 0.3533233 | 1.594431 | high |
| 0.2683146 | 0.6592852 | 0.2276179 | 0.1437199 | 1.7971583 | 0.4324784 | 2.8524783 | 0.6197737 | low |
| 0.232538 | 0.2895984 | 0.0852887 | 0.3337672 | 0 | 0.169412 | 0 | 0.9626496 | low |
| 0 | 0 | 0.2125071 | 0.1431322 | 0 | 0 | 0.5580717 | 1.9866165 | high |
| 0.1071522 | 0 | 0.0756011 | 0.2981891 | 0 | 0.0518593 | 0.2141248 | 1.1805251 | high |
| 0.329181 | 0.2574934 | 0 | 0.6767187 | 0.6014113 | 0.8507194 | 0 | 0.3508829 | low |
| 0.0309716 | 0.2211965 | 0 | 0.2376861 | 0.6305919 | 0.0440443 | 0.1829465 | 1.8796794 | high |
| 0.1273691 | 0 | 0.4197546 | 0.2319239 | 0.158854 | 0.1782366 | 0 | 0.5230586 | low |
| 0.3609822 | 0 | 0 | 0 | 0.4421208 | 0 | 0 | 0.1601595 | low |
| 0.3460775 | 0.0522768 | 0.2164859 | 0.2784606 | 0.9834588 | 0.5276709 | 0.3115031 | 0.1870868 | low |
| 0.2178511 | 0.2467121 | 0 | 0.5340616 | 0.5787938 | 0.1281614 | 0 | 0.4459347 | low |
| 0.0835197 | 0.0797024 | 0 | 0.2686739 | 0 | 0.2262619 | 0 | 0.4720201 | low |
| 0.1050075 | 0.2821432 | 0.559345 | 0.6029319 | 0 | 0.2811938 | 0 | 1.0729096 | high |
| 0.1880211 | 0.2131295 | 0 | 0.1566555 | 0 | 0.2609891 | 0.230449 | 1.2679868 | high |
| 0.1170301 | 0.0569996 | 0.5274708 | 0.0813396 | 0 | 0.679694 | 0.3365119 | 0.3723819 | low |
| 0.0316775 | 0.5718224 | 0 | 1.0892269 | 0.2914857 | 0 | 0.3516283 | 1.1817552 | high |
| 0.9602646 | 0.879758 | 0.2926247 | 0.174449 | 0.993155 | 0.4895429 | 3.0595456 | 30.012552 | high |
| 0.075738 | 0.4435006 | 0.2933705 | 0.4152178 | 0.626953 | 0.1068843 | 0 | 0.6801715 | low |
| 0.0918014 | 0 | 0 | 0.1832006 | 0.4129441 | 0 | 0 | 0.7046534 | low |
| 0 | 0.2040155 | 1.1149666 | 1.9226926 | 0 | 0.1551016 | 0.5808208 | 9.6009645 | high |
| 0.067501 | 0.2697513 | 0.0705267 | 0.1573025 | 0.8847933 | 0.3092926 | 0.2005044 | 0.2506054 | low |
| 0.1190241 | 0.1673579 | 0 | 0.2711866 | 0.9015717 | 0.166844 | 0.3420996 | 0.5663867 | low |
| 0.0713508 | 0.3122005 | 0 | 0.1432628 | 1.1881477 | 0 | 0 | 0.2234173 | low |
| 0.2028878 | 0.1003049 | 0.2115107 | 0.0729976 | 0 | 0.147437 | 0 | 1.5846758 | high |
| 0.1867541 | 0.2862898 | 0 | 0.0885487 | 0.7630716 | 0.3362834 | 0 | 0.0992063 | low |

| | | | | | | | | |
|---|---|---|---|---|---|---|---|---|
| 0.24123 | 0 | 0 | 0.1700532 | 0 | 0 | 1.0925457 | 2.9702002 | high |
| 0 | 0.1700532 | 0 | 0.0842004 | 0.2877087 | 0 | 0.8610819 | 0.563515 | low |
| 0.169412 | 0.187261 | 0.0612923 | 0.5425057 | 0.1437852 | 0.082975 | 0.175301 | 2.1612698 | high |
| 0.0905825 | 0.1680001 | 0.1833277 | 0.6988408 | 0 | 0.2445218 | 0.4886178 | 1.7101053 | high |
| 0.0931545 | 0.531369 | 0.5044679 | 1.98167 | 0.153546 | 0.0450235 | 0.3525326 | 9.6653901 | high |
| 0.0676387 | 0.1264443 | 0.3798427 | 0.6115501 | 0 | 0 | 0 | 3.6134237 | high |
| 0.0360461 | 0 | 0 | 0.353662 | 0.3276874 | 0.1479579 | 0 | 0.8746885 | low |
| 0.064331 | 0 | 0.1314578 | 0 | 0.2990101 | 0.0461418 | 0 | 0.5318282 | low |
| 0.439038 | 0 | 0 | 0.1296127 | 0.7572793 | 0 | 0.4226636 | 1.8417255 | high |
| 0.0377336 | 0.1389455 | 0.0777899 | 0.0766959 | 0.3419857 | 0.2496274 | 0 | 0.6020551 | low |
| 0 | 0.0299829 | 0 | 0.1051417 | 0 | 0 | 0.1851054 | 1.6960858 | high |
| 0.0444641 | 0.4724878 | 0 | 0.1471764 | 0.2113861 | 0.0629506 | 0.8304176 | 1.3374245 | high |
| 0.2156165 | 0.2062681 | 0.116764 | 0.7108781 | 0 | 0.0805212 | 0 | 1.1951917 | high |
| 0 | 0.5566011 | 0 | 0.2260152 | 0.9976176 | 0.1900459 | 0.3864799 | 0.4780244 | low |
| 0.1340895 | 0.0439044 | 0.0947766 | 0.5541468 | 0 | 0.0652966 | 0.2653169 | 2.1910747 | high |
| 0 | 0.0890913 | 0 | 0.1265765 | 0 | 0 | 0 | 0.3760539 | low |
| 0 | 0.1642714 | 0 | 0.1202192 | 0 | 0.3453964 | 0 | 0.5643762 | low |
| 0.0889557 | 0.3132459 | 0 | 0.2322924 | 0 | 0.1251221 | 0 | 1.5694991 | high |
| 0.3441467 | 0.1184926 | 0.0875986 | 0.8329305 | 0.2028878 | 0.4687397 | 0 | 1.8695615 | high |
| 0.0574156 | 0.054779 | 0 | 0.223299 | 0.2689134 | 0.429964 | 0 | 0.7570053 | low |
| 0.0617071 | 0.115566 | 0.1263121 | 0.7580473 | 0 | 0.2473199 | 0 | 1.2734821 | high |
| 0.0803848 | 0.0766959 | 0 | 0.2108875 | 1.1086248 | 0.5752155 | 0 | 0.1402272 | low |
| 0.0429244 | 0.0809305 | 0.2505978 | 0.0588014 | 0 | 0.4273916 | 0 | 0.4984167 | low |
| 0.1335635 | 0.4212637 | 0 | 0.0930192 | 0.4075159 | 0.4033768 | 0 | 0.9133367 | low |
| 0 | 0.1547128 | 0 | 0.5345597 | 0 | 0 | 0 | 1.807309 | high |
| 0.0543623 | 0.3292958 | 0 | 0.178874 | 0.255682 | 0 | 0 | 0.9190599 | low |
| 0.2061431 | 0.6650289 | 0.2147465 | 0.0742314 | 0 | 0 | 0.9675375 | 8.3443878 | high |
| 0.422233 | 0.2950177 | 0.0990932 | 0.458067 | 1.2463473 | 0.2555612 | 0 | 0.1605141 | low |
| 0.3353689 | 0.1152996 | 0.4485838 | 0.5650122 | 0 | 0.3203114 | 0 | 3.7265309 | high |
| 0.0654344 | 0.4135941 | 0 | 0.4674882 | 0 | 0.0469799 | 0.1945914 | 1.409458 | high |
| 0 | 0.1423481 | 0 | 0.1531568 | 0 | 0 | 0.4202937 | 3.2798424 | high |
| 0.2497487 | 0.2389091 | 0 | 1.3101074 | 0.817787 | 0.2814312 | 0.9562797 | 0.1906702 | low |
| 0.2662768 | 0.1330374 | 0 | 0.0654344 | 0.2270017 | 0.3111543 | 0 | 0.5943335 | low |
| 0.0522768 | 0.0499095 | 0 | 0.0714881 | 0.640806 | 0.144438 | 0.2984237 | 0.5090026 | low |
| 0 | 0.2231754 | 0.0647449 | 0.293959 | 0 | 0.3924273 | 0 | 1.0134427 | low |
| 0.1890338 | 0.1807844 | 0 | 0.7426961 | 0 | 0 | 0 | 1.0898535 | high |
| 0.0515809 | 0.0492125 | 1.1981169 | 0.2930173 | 0.2434254 | 0 | 0 | 0.3207518 | low |
| 0.1112985 | 0.582267 | 1.1750455 | 2.4058018 | 0.6846392 | 0.3627785 | 0.9987732 | 4.3437158 | high |
| 0.3905576 | 0.1031283 | 1.2015711 | 0.279293 | 0.4773665 | 0.5291713 | 0.5691022 | 2.5292552 | high |
| 0.2607483 | 0.3056787 | 0 | 0.2266318 | 0.9996393 | 0.3587338 | 0.6925165 | 0.6203181 | low |
| 0.2856981 | 0.2734562 | 0.2973675 | 0.2934882 | 0.3518544 | 0 | 0 | 2.3383884 | high |
| 0.0600474 | 0.1392075 | 0.0626744 | 0.1781091 | 0.514804 | 0.2033891 | 0 | 0.4244175 | low |
| 0.2256451 | 0.0458623 | 0.5806279 | 0.4317834 | 0.2276179 | 0.82546 | 0.8817429 | 0.381726 | low |

| | | | | | | | | |
|---|---|---|---|---|---|---|---|---|
| 0.0413832 | 0.3529845 | 0.3140584 | 0.4218023 | 0.7937711 | 0.2716647 | 0.2398868 | 0.1754234 | low |
| 0 | 0.4785059 | 0 | 0.1206174 | 0.2142492 | 0 | 0 | 2.0082881 | high |
| 0.0519985 | 0.0976108 | 0 | 0.5397296 | 0.2450088 | 0 | 0 | 1.1509943 | high |
| 0.2126316 | 0.7054914 | 0 | 0.5223571 | 0.3415304 | 0.1545832 | 0.2198345 | 1.6677184 | high |
| 0 | 0.1678717 | 0.3455099 | 2.5908894 | 0 | 0.0436245 | 0 | 7.5140829 | high |
| 0.0832474 | 0.2606279 | 0 | 0.1664584 | 0.2012575 | 0 | 0 | 0.5225816 | low |
| 0 | 0.3149865 | 0.093425 | 0.7307486 | 1.0910566 | 0.3489122 | 0 | 0.0840711 | low |
| 0 | 0.0787464 | 0.3173041 | 0.1651723 | 0.3750672 | 0 | 0 | 0.5442044 | low |
| 0.1428708 | 0.5811102 | 0 | 0.3628906 | 0 | 0.1994996 | 1.1984313 | 1.1611362 | high |
| 0.1163648 | 0.3105728 | 0 | 0.4287856 | 0 | 0.1631122 | 0.834873 | 3.2326901 | high |
| 0.2174789 | 0.3317052 | 0 | 0.2464689 | 0.3490255 | 0.2073931 | 1.0087737 | 2.5528186 | high |
| 0.4662356 | 0.0923428 | 0.1009776 | 0.162339 | 0 | 0.0695647 | 0.2817873 | 0.9519871 | low |
| 0.0839282 | 0.2970153 | 0.4603756 | 0.4107753 | 0 | 0.1182268 | 0 | 1.4996628 | high |
| 0.1244605 | 0.5721136 | 0.5871729 | 0.3427823 | 0.2035144 | 0.1184926 | 0 | 1.4830676 | high |
| 0.1895399 | 0.1232689 | 0 | 0.2149951 | 0.3057955 | 0 | 0 | 0.7382568 | low |
| 0 | 0.635708 | 0.4579619 | 0.821547 | 0.5369481 | 0.0890913 | 0 | 1.7730975 | high |
| 0.2126316 | 0.2033891 | 0 | 0.1493894 | 0.8488783 | 0.1545832 | 0 | 0.4682511 | low |
| 0.2674759 | 0.7200157 | 0.1914363 | 0.7249994 | 0 | 0 | 0.5083272 | 16.580807 | high |
| 0 | 0.2244108 | 0.1673579 | 0 | 0 | 0 | 0 | 1.5774327 | high |
| 0.1011122 | 0.403595 | 1.3316479 | 1.6214776 | 0 | 0 | 0.202261 | 28.000335 | high |
| 0.0528332 | 0.4006472 | 0 | 0.4148932 | 0 | 0.0747794 | 0 | 2.0411438 | high |
| 0.1056781 | 0.1952216 | 0.1102296 | 0.0371713 | 0 | 0.2172307 | 0 | 0.6582044 | low |
| 0.6164048 | 0.8292813 | 0.7020369 | 0.0435545 | 2.6274434 | 0.7987556 | 0.6371929 | 0.0371357 | low |
| 0.0735461 | 0.4838803 | 0.6286336 | 4.3966938 | 0 | 0.1036655 | 0.7225535 | 105.54487 | high |
| 0.2311866 | 0.1512094 | 0.164915 | 0.354565 | 0 | 0.2204538 | 0 | 0.9447688 | low |
| 0.2765562 | 0.1384213 | 0.527771 | 2.2218771 | 0.6164518 | 0 | 0 | 4.6211114 | high |
| 0 | 0.0676387 | 0 | 0.0963968 | 0 | 0.0999011 | 0 | 0.7529104 | low |
| 0.3447149 | 0.3718376 | 0.9157886 | 0.6623878 | 0 | 0.4694693 | 0.3102238 | 3.8974465 | high |
| 0.2078929 | 0 | 0.0758749 | 1.2749474 | 0 | 0.1982427 | 0 | 2.5178169 | high |
| 0.03492 | 0.5528689 | 0.0720372 | 0.3817264 | 0 | 0.0494914 | 0.3838287 | 1.9356001 | high |
| 0.0847446 | 0.230326 | 0.3255015 | 0.0588014 | 0.3846025 | 0.2295879 | 0.8100729 | 1.3892502 | high |
| 0.0618453 | 0.369941 | 0 | 0 | 0 | 0.4590119 | 0 | 1.3258626 | high |
| 0 | 0.2085173 | 0 | 2.7945614 | 0 | 0.2078929 | 0.4202937 | 3.1179505 | high |
| 0.1156992 | 0.4833644 | 0.2321695 | 0.9819987 | 0 | 0.5616927 | 0.6035967 | 4.0725541 | high |
| 0.7029235 | 0.0625362 | 0.1963552 | 0.4514353 | 0 | 0.2211965 | 0 | 1.5726712 | high |
| 0.1430015 | 0.2311866 | 0.1491293 | 0.3223897 | 0 | 0.1522483 | 0.2166101 | 0.2872711 | low |
| 0.1367164 | 0.0668125 | 0.7882681 | 0.1845977 | 0.8090851 | 0.1910572 | 0.3885753 | 0.2031448 | low |
| 0.0825664 | 0.153546 | 0 | 0.0571383 | 0 | 0.3238888 | 0 | 0.4247189 | low |
| 0 | 0.2668765 | 0 | 0.1343524 | 0.4420146 | 0.3826119 | 0 | 0.1240232 | low |
| 0.2773897 | 0.3819478 | 1.075738 | 0.2263852 | 0.2367069 | 0.2028878 | 0 | 2.2633043 | high |
| 0 | 0 | 0 | 0.2702299 | 0 | 0.1183597 | 0 | 1.7368935 | high |
| 0.169027 | 0.0558898 | 0 | 0.0405418 | 0 | 0.2353594 | 0 | 0.1781068 | low |
| 0.3795101 | 0.281075 | 0.2106382 | 0.1418251 | 0.4641455 | 0.2151194 | 0.5537537 | 0.7370721 | low |

| | | | | | | | | |
|---|---|---|---|---|---|---|---|---|
| 0.2878269 | 0.3249258 | 0 | 0.0850167 | 0.2901885 | 0 | 0 | 0.811815 | low |
| 0.3678183 | 0.1150332 | 0 | 0.8463128 | 0 | 0.1147668 | 0.444985 | 6.1754803 | high |
| 0.0287106 | 0.0806577 | 0 | 0.3361691 | 0.4912887 | 0.5442875 | 0 | 0.4480249 | low |
| 0.533962 | 0.4499575 | 0.093425 | 0.2336427 | 0 | 0.2963106 | 0.4834676 | 3.909751 | high |
| 0.1764504 | 0.3196179 | 0 | 0.0630887 | 0.4094724 | 0.1278974 | 0.8553519 | 1.9421919 | high |
| 0.175812 | 0.1680001 | 0.6245221 | 0.2363395 | 0 | 0.1273691 | 0.488515 | 2.0850652 | high |
| 0.4056657 | 0.072586 | 0.1545832 | 0 | 0.6286336 | 0.1071522 | 0.4185678 | 0.7279752 | low |
| 0.0716254 | 0.0683269 | 0.1457427 | 0 | 0 | 0.1952216 | 0.3965979 | 1.9495729 | high |
| 0.6964984 | 0.6529683 | 0 | 0.0753729 | 1.3203884 | 1.106594 | 1.194066 | 0.1983789 | low |
| 0.1645289 | 0.1571731 | 0.3246954 | 0.8044666 | 0 | 0.6004601 | 0 | 0.7678022 | low |
| 0.1514692 | 0.2509616 | 0.3002995 | 0.1460035 | 0.4170559 | 0.3606452 | 0 | 0.3001405 | low |
| 0.2081426 | 0.1356662 | 0 | 0.4003193 | 0 | 0.1025909 | 0 | 2.1464827 | high |
| 0.067088 | 0.2947825 | 0 | 0.6672109 | 0 | 0 | 0.6705684 | 3.4664463 | high |
| 0.0871912 | 0.3750672 | 0.1765781 | 0.7886857 | 0.3942946 | 0 | 0 | 0.4646364 | low |
| 0.0654344 | 0.3891262 | 0.1335635 | 0.2525368 | 0.5541468 | 0 | 0 | 1.1588502 | high |
| 0.4370146 | 0.0596322 | 0.1276333 | 0.3827226 | 0.2906603 | 0.4627853 | 0.3506104 | 0.7932162 | low |
| 0.1118326 | 0.3582837 | 0 | 0.2218152 | 0 | 0 | 0 | 1.232784 | high |
| 0.0943712 | 0 | 0 | 0.2458607 | 0 | 0.9008765 | 0 | 0.4993893 | low |
| 0.3645724 | 0 | 0 | 0.4802651 | 0.446468 | 0.4953882 | 0 | 0.3440886 | low |
| 0.0745054 | 0.1054099 | 0.9571717 | 0.3687125 | 0.7384652 | 0.249506 | 0.4109923 | 0.708763 | low |
| 0.0603933 | 0.8061591 | 0 | 0.1411712 | 2.179734 | 1.1069848 | 1.3977208 | 0.010428 | low |
| 0 | 0.1228715 | 0 | 0.0896337 | 0 | 0.1801479 | 0 | 0.9251738 | low |
| 0.0787464 | 0 | 0 | 0.2539893 | 0 | 0.1108977 | 0.4315695 | 2.1763285 | high |
| 0 | 0.2529 | 0 | 0 | 0 | 0 | 0 | 0.9995176 | low |
| 0.116897 | 0.1644001 | 0 | 0.5208497 | 0.2784606 | 0.0842004 | 0 | 0.8772279 | low |
| 0.0451634 | 0.0432045 | 0.0930192 | 0.285343 | 0 | 0.1840898 | 0.2607483 | 1.0305324 | high |
| 0.0790196 | 0.0754642 | 0.3052118 | 0.3458505 | 0.3609822 | 0 | 0 | 2.2585649 | high |
| 0.1528973 | 0 | 0 | 0.5212518 | 0.6453328 | 0.0746424 | 0 | 0.3441084 | low |
| 0.1067503 | 0.3289513 | 0.1112985 | 0.2760797 | 0 | 0.348799 | 0 | 1.3421758 | high |
| 0.2667566 | 0.0681893 | 0.1454818 | 0.5735684 | 0.3289513 | 0.1007086 | 0.3959402 | 0.872786 | low |
| 0.0339339 | 0.0640551 | 0 | 0.1133004 | 0.1634987 | 0.0946415 | 0 | 0.5874559 | low |
| 0 | 0.2510828 | 0 | 0.1852323 | 1.8140171 | 0.2788174 | 0 | 0.0937125 | low |
| 0.4760187 | 0.2210728 | 0.4469972 | 0.1236662 | 1.2109499 | 0 | 0.3450557 | 0.147471 | low |
| 0.1828194 | 0.0457225 | 0.5096452 | 0.0970714 | 0.2270017 | 0.4699901 | 0 | 0.1860516 | low |
| 0 | 0.0588014 | 0.1257833 | 0.5647196 | 0.2867629 | 0.0869196 | 0 | 0.1489708 | low |
| 0.2235461 | 0.3646845 | 0.3564819 | 0.0328061 | 0 | 0 | 0 | 3.0280899 | high |
| 0.0588014 | 0.2123826 | 0.605684 | 0.4557546 | 0 | 0 | 0.3320491 | 1.4858034 | high |
| 0.587941 | 0.1852323 | 0 | 0.1357975 | 0 | 0.4953882 | 0 | 1.5624743 | high |
| 0.2606279 | 0.249506 | 0.1147668 | 0.0387171 | 0.8474357 | 0.0791562 | 0.7983409 | 0.6876395 | low |
| 0.1269729 | 0.3369689 | 0 | 0.2504766 | 0.3011194 | 0.5447821 | 0.3630028 | 0.6825065 | low |
| 0.3006509 | 0.3248106 | 0 | 0.0641931 | 0 | 0.305562 | 0 | 1.9864506 | high |
| 0.2565276 | 0.086512 | 0 | 0.1231364 | 0 | 0 | 0 | 2.1907652 | high |
| 0 | 0.0626744 | 0 | 0.045443 | 0 | 0 | 0 | 0.5271719 | low |

| | | | | | | | | |
|---|---|---|---|---|---|---|---|---|
| 0.2018847 | 0 | 0 | 0.1416944 | 0.6838312 | 0.0604625 | 0 | 1.0092571 | low |
| 0.1868808 | 0.2336427 | 0 | 0.3618806 | 0.7637515 | 0.0918014 | 0.3637878 | 1.3420215 | high |
| 0 | 0.1487389 | 0 | 0.20989 | 0.3645724 | 0.7222037 | 0 | 0.3530572 | low |
| 0.2558028 | 0.5298709 | 0 | 0.4003193 | 0.2579761 | 0.3520805 | 0 | 1.8093232 | high |
| 0.1015156 | 0.6160753 | 0 | 0.0705267 | 0.4518573 | 0.1426095 | 0 | 0.938814 | low |
| 0.1521185 | 0.5098478 | 0 | 0.8186052 | 0.3567073 | 0 | 0 | 5.4466391 | high |
| 0 | 0 | 0.9253918 | 3.3532245 | 0 | 0 | 0 | 6.4471136 | high |
| 0.2655569 | 0.1326426 | 0.1448295 | 1.2349304 | 0 | 0.1940871 | 0 | 2.3068418 | high |
| 0.2856981 | 0.2734562 | 0 | 0.1245928 | 0.1512094 | 0.1696685 | 0 | 3.4076586 | high |
| 0.2820246 | 0.1263121 | 0.0706641 | 0.6570912 | 0 | 0.2692726 | 0.2007554 | 1.6574667 | high |
| 0 | 0.4307135 | 0 | 1.2144357 | 0.167101 | 0.0492125 | 0.2033891 | 0.5299237 | low |
| 0.0735461 | 0.0702519 | 0 | 0.23842 | 0.3379965 | 0.6058736 | 0 | 0.1987646 | low |
| 1.0324535 | 0.4392508 | 0 | 0.3294107 | 0 | 0.1458731 | 0 | 12.455056 | high |
| 0.2865263 | 0.4319973 | 0 | 0.0708015 | 0.9441088 | 0.3929768 | 0 | 0.287927 | low |
| 0.1388145 | 0.0275787 | 0.2251515 | 1.1955996 | 0 | 0.1939609 | 0.3234277 | 0.4561428 | low |
| 0.0965317 | 0.260387 | 0.6135317 | 1.8080555 | 0.1587248 | 0.0918014 | 0 | 4.2528562 | high |
| 0.2234225 | 0.1319845 | 0.4330663 | 0 | 0 | 0.1315895 | 0.5025343 | 0.6812703 | low |
| 0.3386811 | 0.1164978 | 0.6351507 | 0.3817264 | 0 | 0 | 0 | 1.6899033 | high |
| 0.1651723 | 0 | 0.5084287 | 0.3218127 | 0 | 0.0807941 | 0 | 1.4828032 | high |
| 0.191689 | 0.063779 | 0 | 0.2174789 | 0.7800581 | 0.1826923 | 0.3726178 | 0.1308307 | low |
| 0.103934 | 0.0992279 | 0.2092662 | 0.3871419 | 0 | 0.1458731 | 0 | 0.8590923 | low |
| 0.2990101 | 0.2862898 | 0.1112985 | 0.2118844 | 0.4722798 | 0 | 0 | 1.4598453 | high |
| 0.1307991 | 0.1248575 | 0.0923428 | 0.2041407 | 0 | 0 | 0.2590615 | 1.6446825 | high |
| 0.0805212 | 0.1497796 | 0 | 0.055751 | 0 | 0.4081684 | 0 | 0.3724493 | low |
| 0.6178627 | 0.0451634 | 0.1886541 | 0.3248106 | 0.4192153 | 0.1925729 | 0.2723816 | 2.2561514 | high |
| 0 | 0.1558787 | 0 | 0.0581086 | 0 | 0 | 0 | 2.0394978 | high |
| 0.0856966 | 0.6088092 | 0.3286067 | 0.548338 | 0 | 0 | 0.4653999 | 2.3641274 | high |
| 0.2240403 | 0.0750534 | 0 | 0.0545012 | 1.2709475 | 0.3997727 | 0 | 0.0526554 | low |
| 0.2157407 | 0.2063932 | 4.1856525 | 3.6802456 | 0 | 0.1064823 | 0.4162993 | 87.563515 | high |
| 0 | 0.6332917 | 0 | 0.450591 | 0.5348585 | 0 | 1.2529 | 4.7094164 | high |
| 0 | 1.0878701 | 0.5788904 | 0.7215039 | 0 | 0 | 0 | 2.1571233 | high |
| 0.3306731 | 0.2187192 | 1.230449 | 1.124262 | 0.5193406 | 0.1665869 | 0.3415304 | 2.3381597 | high |
| 0.1858665 | 0.9718102 | 1.340961 | 4.0897693 | 0 | 0.1770886 | 0.891808 | 289.3748 | high |
| 0.1513393 | 0.0980152 | 0 | 0.235482 | 0 | 0.0374525 | 0 | 0.824336 | low |
| 0.9632275 | 0.1865006 | 0.6797841 | 0.3479302 | 3.3505727 | 0.3124328 | 2.5281545 | 0.3420444 | low |
| 0.5856997 | 0.7476882 | 0 | 0.642563 | 2.6886504 | 1.0571152 | 2.135305 | 0.3501442 | low |
| 0.293135 | 0 | 0 | 0.072586 | 0 | 0 | 0 | 1.5849235 | high |
| 0.4103411 | 0.1882743 | 0 | 0.2023864 | 0 | 0.554638 | 0 | 0.0810846 | low |
| 0 | 0.1440464 | 0.0648829 | 0.0430645 | 0.4151096 | 0.1298765 | 0 | 0.7559167 | low |
| 0.1870076 | 0.1099623 | 0 | 0.251204 | 0 | 0 | 0 | 1.017707 | high |
| 0.2149951 | 0.5454742 | 0.0676387 | 1.9164766 | 0.1580785 | 0.1773438 | 0.6518668 | 7.4057109 | high |
| 0.1423481 | 0.0695647 | 0 | 0.4393572 | 0 | 0 | 0 | 1.6537072 | high |
| 0.1624679 | 0.0797024 | 0 | 0.0578315 | 0 | 0.117695 | 0 | 0.9430651 | low |

| | | | | | | | | |
|---|---|---|---|---|---|---|---|---|
| 0 | 0.0923428 | 0.1953476 | 0 | 0 | 0 | 0 | 1.047872 | high |
| 0 | 0.1565261 | 0 | 0.0773797 | 0 | 0.1560082 | 0 | 0.3970622 | low |
| 0.1978654 | 0 | 0.1406478 | 0.047678 | 0.3186928 | 0 | 0.9367416 | 1.0420465 | high |
| 0.1811662 | 0.2905424 | 0 | 0.8139351 | 0.4195389 | 0.1928254 | 0.2726205 | 0.9306333 | low |
| 0 | 0.0972062 | 0.1062143 | 0 | 0.2441564 | 0.2730981 | 0 | 0.4954564 | low |
| 0.2072682 | 0.0521376 | 0.1119661 | 0.1104969 | 0 | 0 | 0 | 1.1970234 | high |
| 0.0918014 | 0.3930867 | 0 | 0.8935175 | 0.4131608 | 0 | 0.4944668 | 5.4680533 | high |
| 0.1026581 | 0.2886539 | 0.0736832 | 0.800786 | 0.325444 | 0.6191782 | 0 | 0.6183704 | low |
| 0.077243 | 0.2107629 | 0 | 0.154972 | 0.3534362 | 0 | 0 | 0.174767 | low |
| 0 | 0.3515153 | 0.4468914 | 0.1103632 | 0 | 0 | 0 | 1.2438267 | high |
| 0.1584663 | 0.1514692 | 0 | 0.3401634 | 0 | 0.0775165 | 0 | 0.8710251 | low |
| 0.1263121 | 0.1207501 | 0.2526579 | 0.6177686 | 0.2997136 | 0.0911243 | 0 | 0.8927401 | low |
| 0.0350608 | 0.3314759 | 0.8670266 | 0.0945063 | 0 | 0.0978804 | 0 | 3.4123649 | high |
| 0.1800205 | 0.1197767 | 0 | 0.6419775 | 2.5752881 | 0.2383385 | 1.0616149 | 0.1424998 | low |
| 0.3362834 | 0 | 0 | 0.2826177 | 0 | 0.1040682 | 0 | 1.6572621 | high |
| 0.8105665 | 0.1937087 | 0 | 0 | 0 | 0.1471764 | 0.5548344 | 4.6568578 | high |
| 0.1727435 | 0.5104556 | 0 | 0.3850444 | 0 | 0 | 0.4809889 | 3.6808608 | high |
| 0 | 0.1401242 | 0 | 0.4513298 | 0 | 0.2046416 | 0 | 0.3538543 | low |
| 0.1126334 | 0.054779 | 0 | 0.3231971 | 0 | 0.3666999 | 0 | 0.4685912 | low |
| 0.0691522 | 0.2188432 | 0.0721744 | 0.0942361 | 0 | 0.0496308 | 0 | 1.1002038 | high |
| 0.1997509 | 0.2778658 | 0 | 0.0717627 | 0.4591168 | 0.2770326 | 0 | 0.2843823 | low |
| 0.1166309 | 0.2148708 | 0 | 0.429964 | 0 | 0 | 0 | 0.9491242 | low |
| 0.141302 | 0.1350094 | 0 | 0 | 0 | 0.1973622 | 0 | 0.5499977 | low |
| 0.0469799 | 0.1304038 | 1.5536063 | 0.4229865 | 0.2225573 | 0.2491419 | 0.269871 | 1.4480873 | high |
| 0.3061456 | 0.1044709 | 0.1142337 | 0.1830736 | 0.8441446 | 0.153546 | 0 | 0.331601 | low |
| 0.0713508 | 0.1953476 | 0.1453514 | 0.0972062 | 0 | 0.3663642 | 0.9609565 | 1.5250116 | high |
| 0 | 0.1824381 | 0 | 0.5231605 | 0.2368293 | 0 | 0.2867629 | 1.0372248 | high |
| 0.3179986 | 0 | 0.4262111 | 0.0792928 | 0 | 0 | 0.5957901 | 0.7146649 | low |
| 0.1448295 | 0.4533332 | 0 | 0.4465739 | 0 | 0.138028 | 0 | 1.6455522 | high |
| 0.9498711 | 0.0485152 | 0.1042024 | 0.5535571 | 0.2397646 | 0.6895675 | 0 | 2.4105153 | high |
| 0.1174291 | 0.1389455 | 0.288772 | 0.3359405 | 0 | 0.0846086 | 0.3377682 | 6.7175232 | high |
| 0.2244108 | 0.0751903 | 1.4643546 | 3.4672273 | 0.3596336 | 0.782073 | 0.7639214 | 7.3785761 | high |
| 0.3135941 | 0.6047356 | 0 | 0.2732175 | 0.2055176 | 0 | 0.249506 | 2.7623105 | high |
| 0 | 0.3012364 | 0 | 0.4755 | 0 | 0.1579492 | 0 | 1.63257 | high |
| 0.2062681 | 0.3708338 | 0 | 1.4829515 | 0 | 0 | 0 | 1.4937499 | high |
| 0 | 0.1628545 | 0 | 0.4814023 | 0 | 0.0506062 | 0 | 2.1532441 | high |
| 0.6769895 | 0.8603669 | 0 | 1.4662356 | 0 | 0 | 0 | 6.2066835 | high |
| 0.1547128 | 0.5174269 | 0 | 1.08882 | 0.3623296 | 0.6456094 | 0 | 0.4058906 | low |
| 0 | 0.1634987 | 0 | 0.0610158 | 0 | 0 | 0 | 1.3424542 | high |
| 0.2875905 | 0.0739573 | 0.1574318 | 0.4634132 | 0.3541136 | 0.8365705 | 0 | 0.3963637 | low |
| 0.417488 | 0.0565835 | 0.4338141 | 0.3653566 | 0.7092906 | 0.5042644 | 1.2046416 | 2.4958187 | high |
| 0.0629506 | 0 | 0.1285574 | 0 | 0.7442472 | 0.3981314 | 0.3533233 | 0.3220103 | low |
| 0.0462815 | 0.2085173 | 0.5630605 | 0.1532865 | 0 | 0 | 0 | 0.7766535 | low |

| | | | | | | | | |
|---|---|---|---|---|---|---|---|---|
| 0.2998308 | 0.0391384 | 0.0846086 | 0.2125071 | 0.1962293 | 0.0582472 | 0 | 0.6170099 | low |
| 0.1430015 | 0.3761792 | 0 | 0.1470461 | 0.3366262 | 0.455439 | 0 | 0.7646485 | low |
| 0.1235338 | 0.5407217 | 0.0871912 | 0.4089292 | 0 | 0.0600474 | 0 | 6.2436883 | high |
| 0.2048919 | 0.1012467 | 0.1106305 | 0.1091599 | 0.4697818 | 0.4651909 | 0 | 0.6632291 | low |
| 0.0364682 | 0.2273714 | 0 | 0.3173041 | 0 | 0 | 0 | 1.5875679 | high |
| 0.1130337 | 0.3903375 | 0 | 0.5337627 | 0 | 0 | 0 | 1.28754 | high |
| 0.0873271 | 0.16221 | 0.6060631 | 0.0899049 | 0.5583656 | 0.6172044 | 0 | 0.2782878 | low |
| 0.2051422 | 0.1961034 | 0.1108977 | 0.177854 | 0.4704067 | 0.1491293 | 0 | 0.7662477 | low |
| 0 | 0.1933302 | 0 | 0.4989657 | 0 | 0.1926992 | 0 | 0.8220398 | low |
| 0.064607 | 0.1774714 | 0.1318528 | 0.0880059 | 0.2998308 | 0 | 0.8911081 | 1.3853512 | high |
| 0.0657102 | 0.6574571 | 0.1340895 | 0.3651326 | 0.5559143 | 0.1798932 | 0 | 0.6564584 | low |
| 0.2006299 | 0.4539653 | 0.3213509 | 0.8475961 | 0.3797318 | 0.117828 | 0 | 0.7568185 | low |
| 0.3702759 | 0.231801 | 0 | 0.0706641 | 0.4532278 | 0 | 0.295723 | 0.9394457 | low |
| 0.3610946 | 0 | 0 | 0.1972364 | 0 | 0.4908781 | 0.5284713 | 1.4049306 | high |
| 0 | 0.3017047 | 0.711583 | 0.1160986 | 0 | 0.2313095 | 0 | 2.4845708 | high |
| 0 | 0.1443075 | 0 | 0.0536674 | 0.354565 | 0.2107629 | 0 | 0.5654839 | low |
| 0.1830736 | 0.107554 | 0 | 0.6219932 | 0.6286336 | 0.0546401 | 0.2244108 | 0.8164719 | low |
| 0 | 0.1840898 | 0 | 0.1350094 | 0 | 0 | 0 | 0.1552151 | low |
| 0 | 0 | 0.2216915 | 0.2848694 | 0 | 0 | 0 | 0.9273461 | low |
| 0.2390314 | 0.2703495 | 0 | 0.5316684 | 0.2409859 | 2.0648484 | 0 | 0.2352977 | low |
| 0.1987456 | 0.1170301 | 0 | 0.2169825 | 0.2003788 | 0 | 0.4514353 | 0.4965322 | low |
| 0 | 0.1939609 | 0 | 0 | 0 | 0 | 0 | 0.6292348 | low |
| 0.0994972 | 0.1840898 | 0 | 0.3720606 | 0.2389091 | 0.3841604 | 0 | 0.2346419 | low |
| 0 | 0.3474389 | 0.261952 | 0.0913952 | 0.971516 | 0.1835818 | 0.6712028 | 0.991138 | low |
| 0.0698396 | 0.3603081 | 0.1423481 | 0.3476657 | 0.5854433 | 0 | 0.945308 | 0.864843 | low |
| 0 | 0 | 0 | 0.1654296 | 0.2906603 | 0.0881416 | 0.3507235 | 1.1158405 | high |
| 0 | 0.3448285 | 0.1991226 | 0.790355 | 0 | 0 | 0.5265697 | 0.4118455 | low |
| 0.5342609 | 0.3469852 | 0 | 0.1889073 | 0 | 0.606537 | 0 | 1.7066152 | high |
| 0.2620723 | 0.4643546 | 0.1428708 | 1.5094425 | 0 | 0.1914363 | 0.3893465 | 2.9333888 | high |
| 0.0594937 | 0.0843364 | 0.178874 | 0.0411028 | 0 | 0 | 0 | 1.0977883 | high |
| 0.1545832 | 0.2027625 | 1.3828886 | 0.7649406 | 0.296663 | 0.5049763 | 0 | 0.1445762 | low |
| 0 | 0.6769895 | 0.1627257 | 0.3049782 | 0 | 0.1129003 | 0 | 6.0977929 | high |
| 0.1634987 | 0.0233263 | 0 | 0.1298765 | 0 | 0.0347792 | 0.1462642 | 1.6521428 | high |
| 0 | 0 | 0 | 0.0791562 | 0.5001902 | 0 | 0 | 0.5388708 | low |
| 0.0568609 | 0.2056427 | 0 | 1.4964623 | 0 | 0 | 0 | 0.6816882 | low |
| 0.5783108 | 0 | 0 | 0.154324 | 0 | 0 | 0 | 1.3111753 | high |
| 0 | 0.0764223 | 0.1624679 | 1.0681205 | 0.6557182 | 0.2172307 | 0 | 1.4651252 | high |
| 0.2790552 | 0.7385517 | 0.3991164 | 0.2991274 | 0.5474498 | 0 | 0 | 0.6840195 | low |
| 0 | 0.2468337 | 0 | 0.4421208 | 0 | 0.1282934 | 0 | 0.7183699 | low |
| 0 | 0.1491293 | 0 | 0.2584586 | 0.3654686 | 0.1129003 | 0.7746711 | 0.7822347 | low |
| 0.2990101 | 0.1971105 | 0 | 0.0740944 | 0 | 0.4094724 | 0 | 0.8027096 | low |
| 0.3378823 | 0.3944044 | 0.3514022 | 0.3469852 | 0 | 0 | 0 | 0.9615961 | low |
| 0.104605 | 0 | 0.2105136 | 0 | 0 | 0.2801249 | 0 | 0.3560182 | low |

| | | | | | | | | |
|---|---|---|---|---|---|---|---|---|
| 0.0930192 | 0.2516888 | 0.1881477 | 1.0051844 | 0 | 0.1309309 | 0 | 1.251842 | high |
| 0.0342157 | 0 | 0 | 0.0468403 | 0.1650436 | 0.2278643 | 0.200881 | 0.5397735 | low |
| 0.041243 | 0.1150332 | 0.0850167 | 0.13711 | 0 | 0.1146335 | 0 | 0.6080782 | low |
| 0 | 0.0561673 | 0.1204847 | 0.0406821 | 0.2751263 | 0.1618232 | 0.3323929 | 0.899584 | low |
| 0 | 0.0496308 | 0 | 0.1713349 | 0 | 0.0735461 | 0.5429019 | 1.5447145 | high |
| 0.1243281 | 0.1187584 | 0 | 0.3542265 | 0 | 0 | 0 | 1.6360139 | high |
| 0.0897693 | 0.2431816 | 0 | 0.2343787 | 0.7206287 | 0 | 0 | 0.340563 | low |
| 0.0850167 | 0.1948435 | 0.0886844 | 0.4594316 | 0.3852654 | 0.2822619 | 0.6470842 | 1.7734398 | high |
| 0.3355976 | 0.3917677 | 0 | 0.8555114 | 0 | 0 | 0 | 2.4164574 | high |
| 0 | 0.4988636 | 1.0252415 | 3.2422366 | 0 | 0.6128714 | 0 | 1.4308464 | high |
| 0.1894134 | 0.1811662 | 0.0822938 | 0.0275787 | 0.359746 | 0.2141248 | 0 | 1.1986703 | high |
| 0 | 0.0820213 | 0 | 0.144699 | 0.3893465 | 0.2326608 | 0 | 0.3012426 | low |
| 0.1058122 | 0.1955996 | 0 | 0.0735461 | 0.2535053 | 0 | 0 | 1.4121117 | high |
| 0.2993619 | 0.380951 | 0 | 0.5991273 | 0.1958515 | 0.269512 | 0.2379308 | 1.3525246 | high |
| 0 | 0.0821576 | 0 | 0.276318 | 0 | 0 | 0 | 1.9156918 | high |
| 0.1074201 | 1.0226874 | 0 | 0.6637545 | 0 | 0.1508196 | 0 | 14.133876 | high |
| 0.5869809 | 0.1170301 | 0.3184615 | 0.1393385 | 0 | 0 | 0.2433035 | 0.7738428 | low |
| 0.272501 | 0.5319678 | 0 | 0.6188025 | 0.3360548 | 0.2894804 | 0 | 0.2623888 | low |
| 0 | 0 | 0 | 0.2983064 | 0.744075 | 0 | 0.5018213 | 0.6180478 | low |

| Nomogram |
| ---: |
| 2.5375271 |
| 7.2203844 |
| 1.4527984 |
| 0.5476954 |
| 1.8620509 |
| 0.5693557 |
| 0.5753727 |
| 3.4426351 |
| 1.3541416 |
| 0.5251421 |
| 3.6379794 |
| 0.4040199 |
| 0.9508621 |
| 0.5873975 |
| 0.7340556 |
| 0.5587862 |
| 1.3648604 |
| 4.1970559 |
| 0.5738625 |
| 0.5922493 |
| 1.3929742 |
| 2.5242241 |
| 0.570854 |
| 2.6102956 |
| 0.5648843 |
| 0.5387837 |
| 0.3876295 |
| 0.5658174 |
| 0.3894245 |
| 7.657816 |
| 0.928635 |
| 0.3966551 |
| 0.9483664 |
| 1.4423063 |
| 1.15102 |
| 0.5520308 |
| 2.5576133 |
| 0.4019018 |
| 0.3935399 |
| 0.5476954 |
| 1.3017968 |
| 2.0916792 |

2.5612686
0.3795645
2.4075918
2.6171649
8.8767067
2.4912709
0.5637602
0.5667313
1.4040005
0.4180621
1.3224874
2.1155644
2.6034444
0.5738625
2.5478411
1.2829958
0.3896723
4.9635096
7.1332481
1.0426393
2.2651786
0.3884024
0.395614
0.6016625
0.9558733
0.4172302
2.5226186
1.0704047
4.8426739
1.3505874
1.4604549
0.5502317
0.5814533
0.3795645
0.5833545
4.2044397
0.5549401
0.9730021
1.3711808
1.0290279
6.8980374
1.3678945
0.3966551

0.4093641
1.3938607
8.7378285
1.3711808
6.9526396
0.3896723
0.3935399
1.0697239
2.8401596
3.4995779
2.484732
0.5578647
1.6578373
3.373154
0.3966551
4.1313919
0.4061491
1.2915731
0.980704
3.8813186
0.9628269
0.5363002
0.6160635
4.0800525
0.4050831
2.5710922
0.3845852
0.9609109
2.4978269
1.4003154
0.9577792
1.3820345
3.7446873
1.0147897
2.7152548
0.5593328
0.4235921
1.1869733
0.3894245
2.4394381
3.2598534
0.3966551
1.1173492

1.3607233
0.9508621
2.44E-07
2.944733
3.9223366
3.7570642
1.3887518
3.0311068
0.407218
0.3863662
0.577254
1.4489852
2.4652183
1.0182668
3.4790171
0.4158704
2.5576133
0.6042423
1.0129285
0.5476954
1.0810288
0.5476954
2.7068179
0.5663709
1.3607233
0.9884669
1.3532803
0.783407
1.4026284
1.2035409
0.3976989
0.5279097
0.5549401
0.5619229
1.3823505
2.4912709
2.8178545
1.4113997
0.8848366
1.0613229
1.3779365
5.0607232
0.5549401

0.4158704
1.0147897
0.3805634
1.0445551
1.2237146
3.4698857
3.5739363
1.4451821
1.4085912
2.8178545
0.4019018
0.5534835
1.4705767
1.2596082
0.5433941
2.2341852
0.3894245
3.0650892
0.921342
1.0365136
1.3859883
2.5966111
1.430069
1.1427073
1.3865534
2.5762187
0.9409185
1.144918
2.4111241
1.4489852
4.6753395
0.9884669
3.3291182
0.3765837
0.5476954
0.5564004
2.4717058
0.6016625
0.7316633
1.3364635
4.7716434
2.5109908
1.456028

7.1190534
1.1855353
2.3438191
0.4061491
0.5448241
1.3435069
2.9234661
0.5377116
1.0592109
0.9936763
1.2131394
0.5575099
6.8079849
0.5682227
0.9502574
2.2696952
0.980704
1.0732216
0.4180621
1.0336317
1.3742289
0.397952
1.0170456
1.4040005
1.0508925
2.5694569
1.3461857
0.5787731
0.9409185
2.5643439
2.5966111
1.4076952
1.8718641
2.1776174
0.9858725
2.8551276
1.3364635
0.5593328
1.4338324
1.0310796
0.9864999
0.3866121
0.3835758

0.3935399
0.629941
0.989096
0.3976989
2.6171649
1.3815627
0.397952
0.560448
0.5768869
0.9884669
0.5448241
0.5520308
1.1501228
1.3747892
2.6188306
0.4104413
3.583124
0.3958657
1.0386556
1.0076182
0.392507
0.5531315
0.5979234
0.3835758
0.7511474
3.390931
1.3678945
0.9710659
3.4426351
2.6448233
1.0182668
7.2703177
3.4426351
1.1816716
1.0890587
2.5044002
1.3820345
0.5430485
1.0496322
0.5545871
0.5738625
0.3803214
0.5433941

3.5180212
0.7472095
0.412867
0.3987455
3.3554702
0.9787525
0.7379242
1.4489852
2.2283212
2.5982637
0.9761835
0.5860558
3.3621607
1.3399806
1.3568416
7.003217
0.7259172
0.5473471
0.7202162
